# The First Asynchronous Online Evidence-Based Medicine Course for Syrian Health Workforce: Effectiveness and Feasibility Pilot Study

**DOI:** 10.2196/36782

**Published:** 2022-10-25

**Authors:** Yazan Kenjrawi, Mayssoon Dashash

**Affiliations:** 1 Medical Education Program Syrian Virtual University Damascus Syrian Arab Republic; 2 Faculty of Dentistry Tishreen University Latakia Syrian Arab Republic; 3 Pediatric Dentistry Department Faculty of Dentistry Damascus University Damascus Syrian Arab Republic

**Keywords:** evidence-based medicine, continuous medical education, web-based learning, Syria, medical graduates, medical education, web-based course

## Abstract

**Background:**

Evidence-based medicine (EBM) is critical for providing the best scientifically proven patient health care, and it is implemented worldwide in order to improve the quality of the delivered health care. However, not all Syrian health professionals are knowledgeable about the importance, methodology, and implementation of EBM. Providing web-based learning courses on EBM might be effective in improving the EBM knowledge of health care professionals.

**Objective:**

This study was performed to test the effectiveness and the feasibility of an asynchronous web-based course on EBM in improving the competencies of Syrian health care professionals in terms of EBM.

**Methods:**

A web-based course on EBM was developed in Arabic and uploaded onto the Syrian Virtual University platform. An electronic registration form was designed and distributed to medical groups on social media for registration to this web-based course. Both the pretest and posttest had the same 3 sections to measure the impact of this web-based EBM program on the knowledge, skills, and attitudes of the Syrian health care professionals. The posttest had an additional section for measuring the efficacy and ease of use of this program. Student paired 1-tailed *t* test was used to analyze the differences in the different assessment sections among the participants.

**Results:**

Nineteen participants filled the electronic registration form, but 8 participants did not meet the inclusion criteria. Therefore, the pretest was sent to the remaining 11 participants (7 men and 4 women) who graduated from Syrian universities. Ten of them completed the pretest, while 7 of them completed the posttest. The web-based course was found to be effective in improving the participants’ EBM knowledge, skills, and attitudes at *P*>.05. Further, the web-based EBM course was feasible and easy-to-use.

**Conclusions:**

In order for EBM to be implemented in Syria, continuous medical education training programs should be designed for clinical practitioners. Our study shows that asynchronous web-based medical education is an effective and a feasible means for introducing the concept of EBM, improving practitioners’ skills, and promoting the positive attitudes of Syrian clinical practitioners toward EBM.

## Introduction

Evidence-based medicine (EBM) is the integration of well-designed research with clinical experience and patients’ beliefs that aim at developing decision-making through the utilization of the best available scientific evidence [1]. The practice of EBM requires formulating a question based on the clinical problem faced, searching the available literature to find evidences, and critically appraising the evidence in order to find the best application on the patient [2]. This process is followed by the evaluation of each of the last 4 steps of EBM (explained in the subsequent section) to make sure that the goal of EBM in improving patient care has been achieved [2].

EBM promotes a patient-centered approach by making patients’ preferences and values an important component of the treatment process [3]. EBM is also a cost-effective approach since it promotes what has already been proven to be effective [4]. This will be reflected not only on the health care provider but also on the patient, which makes its implementation of special importance in limited-resource countries. EBM provides practitioners with an effective continuous medical education strategy by offering the required tools needed to keep up with the most recent scientific evidence [5] and benefits patients as it will provide them with the best scientifically approved health care.

EBM was first introduced in the medical programs in 1990 at McMaster University [6]. Since then, it has been a part of the medical programs offered in universities worldwide [7]. In limited-income countries, however, teaching EBM is faced with major barriers such as the lack of educational resources and the language barrier [5]. These barriers are more obvious in Syria, as EBM was still not part of the undergraduate medical program in public universities until 2017 [8]. Moreover, medical education in Syria is delivered in Arabic, with the vast majority of students having the desire to improve their English skills to be able to raise their scientific standards, but the English language is still a significant barrier to learning [9]. This barrier has resulted in producing doctors who are not familiar with EBM and who lack the skills needed to learn about this concept on their own, since educational resources on EBM are rare in Arabic. Another barrier is that clinical practitioners usually lack the needed time to master EBM skills [6].

e-Learning is as an approach that utilizes the internet and digital technologies to deliver mediated, well-designed, learner-centered, and interactive learning to anyone at any place and anytime according to instructional design principles [10]. Several studies have shown that e-learning is as effective as the traditional methods of learning EBM [11-13]. In fact, asynchronous web-based learning is superior to face-to-face learning, because it offers the opportunity for learners to learn based on their availability without worrying about a specific time or place [14], which makes it the perfect option for busy clinical practitioners. Additionally, social distancing procedures due to the COVID-19 pandemic make web-based learning the safest option, specifically for clinical practitioners [15]. Web-based education facilitates learning from different geographical places [14], which is particularly useful in war-torn Syria, where moving across the country is not an option for many Syrians [16]. In addition, web-based learning in medical education has already been proven to be successful in Syria during the ongoing crisis and is suggested to be further implemented to cover the other health topics [17]. Regarding EBM, peer-taught web-based courses should be implemented in limited-income countries as this is found to be an effective approach [18].

In light of the above reasons, we developed an asynchronous web-based EBM course in Arabic that would be delivered by a peer of the targeted study population in order to investigate its effects on Syrian medical graduates’ competencies and to study the course’s efficacy and feasibility.

## Methods

### Electronic Registration Form and Participant Selection Criteria

An electronic registration form was designed using Google forms. This form included an introduction about the web-based course and the reason for choosing the internet to deliver this course. The form also included the learning objectives of the web-based course in addition to how those objectives are connected to the learning contents, the specified duration of each video in the asynchronous web-based session, and a detailed description of the expected time for the whole web-based course. This form worked as an advertising tool in addition to its use as a registration form. The e-form was distributed to medical groups on both Telegram and WhatsApp. To be accepted in this web-based course, the participant had to be a clinical practitioner who had graduated from a Syrian medical faculty.

### Web-Based EBM Course Design

The design of the web-based course followed the objective-based approach. The learning objectives were set to meet the need of medical practitioners who were currently working in clinical settings and who did not have the opportunity to learn about EBM in their undergraduate medical program. The web-based EBM course had 3 main learning objectives. The first objective was to define and introduce the concept of EBM and to explain its 5 steps. The second objective focused on improving the knowledge and skills about the methodology and how to apply EBM while approaching clinical problems in clinical settings. The last learning objective was related to the clinical practice guidelines and its importance in clinical settings.

After setting those objectives, YK, who was a peer of the targeted study population, developed a screening process for the medical literature to design the material of the web-based course. Considering the time problem and the language barrier that deprive clinical practitioners from learning this concept, 11 presentations were designed in Arabic using the PowerPoint 2016 software. Those were turned into 11 video lessons by YK who explained the presentation screens in Arabic by using 2 free video recording software, namely, Loom and iFun Screen Recorder. For editing purposes, OpenShot Video Editor, which is an open-source software, was used to remove the noise, silence, and errors clips from the videos. On average, a lesson lasted for 6 minutes.

The lessons were categorized under 4 learning units. The first learning unit was based on the first learning objective. At the start of the second unit, participants were introduced to a clinical problem, and they were asked to turn this problem into the PICO (Patient, Intervention, Comparison, and Outcome) format question and to search for an answer in one of the medical search engines (eg, Cochrane, PubMed). This was a voluntary task and participants were able to find the best solution for that PICO task problem in the last lesson of the second unit. In the same unit, there was also a practical session where a clinical problem was presented, turned into a PICO question, and then a medical search engine (PubMed) was searched for an answer to that PICO question. The third learning unit was also related to the second learning objective. It focused on the last 3 steps of the EBM implementation. It started by introducing the scientific evidence pyramid and then moving onto defining the concept of validity and reliability. This unit also dealt with the application of the evidence and the evaluation process of each step of EBM. The fourth learning unit covered the last learning objective, wherein the clinical practice guidelines were defined, and the components, databases, and the benefits of following the recommendations of clinical practice guidelines were listed. Participants needed approximately 70 minutes to finish all the materials of the web-based EBM course. Table 1 shows the web-based course map (Multimedia Appendices 1-Multimedia Appendices 11).

**Table 1:** The web-based course map of evidence-based medicine in clinical settings (Multimedia Appendices 1-Multimedia Appendices 11).

Objective, learning unit and objective, lesson title				Duration		PowerPoint Presentation
**Learning objective 1^a^**						
	**First learning unit: Introduction to evidence-based medicine**					
		Defining evidence-based medicine	5 min		Multimedia Appendix 1	
		The importance of evidence-based medicine	8 min		Multimedia Appendix 2	
		Explanation of the 5 steps of evidence-based medicine	5 min		Multimedia Appendix 3	
**Learning objective 2^b^**						
	**Second learning unit: Clinical problem solving**					
		Patient, Intervention, Comparison, and Outcome format	8 min		Multimedia Appendix 4	
		How to search in medical search engines	7 min		Multimedia Appendix 5	
		Practical training	5 min		Multimedia Appendix 6	
	**Third learning unit: Appraise, apply, and evaluate our approach to solving a clinical problem^c^**					
		Critical appraising 1	8 min		Multimedia Appendix 7	
		Critical appraising 2	7 min		Multimedia Appendix 8	
		Application guide and process calendar	4 min		Multimedia Appendix 9	
**Learning objective 3^d^**						
	**Fourth learning unit: Clinical guidelines**					
		Clinical practice guidelines	6 min		Multimedia Appendix 10	
		The importance of clinical practice guideline	4 min		Multimedia Appendix 11	

^a^Define the concept of evidence-based medicine, summarizing its benefits, and explaining its 5 steps.

^b^Apply the 5 steps of evidence-based medicine to solve clinical problems encountered during clinical work. Step 1: convert your clinical problem into an answerable question. Step 2: Searching medical search engines for evidence using the Patient, Intervention, Comparison, and Outcome format.

^c^Apply the 5 steps of evidence-based medicine to solve clinical problems encountered during clinical work. Step 3: critically evaluate studies. Step 4: application of the practical guide. Step 5: defining the process of evaluating the steps of evidence-based medicine.

^d^Define the clinical practice guideline and explain its components and importance for health care.

### Knowledge, Skills, and Attitudes of Participants Regarding EBM

The EBM knowledge, skills, and attitudes were defined in the context of this study as follows.

#### EBM Knowledge

This involves the understanding of the EBM triad and pointing out its importance, in addition to being able to explain the 5 steps of EBM and the evidence pyramid, and defining the clinical practice guideline, its components, and importance.

#### EBM Skills

This involves effectively being able to turn a clinical problem into a PICO format and to search for an answer in a web-based medical database and applying the critical appraisal skills and implementing the evidence and the evaluation of the entire process.

#### Attitude of Participants Toward EBM

This involves the willingness to learn and improve knowledge and skill with regard to EBM and support the implementation of EBM on a large scale in the Syrian community.

### Measurements

The pretests and the posttests were drafted in Arabic and designed using Google forms. The first section of the pretest focused on the demographic information of the participants. The other 3 sections were designed to test the participants’ knowledge, skills, and attitudes regarding EBM in the context of this study. All 3 sections were designed based on the learning objectives of the web-based EBM course. The knowledge section included 20 questions: 12 multiple choice questions and 8 true or false questions. For the skills section, a clinical problem was introduced followed by 8 multiple choice questions about its PICO elements in addition to the best study type to answer that type of PICO question. The last section consisted of 20 statements on the Likert scale to test the attitudes of the participants regarding EBM. The posttest differed from the pretest in that it had an extra section that measured the usefulness and ease of use of the web-based course. This section included 14 statements evaluated on the Likert scale [19]. The assessment was checked for content and face validity by the supervisor MD, an expert in the field of EBM and a member of the Cochrane Collaboration. However, no criterion or construct validity of the questions was attempted.

### Study Design and Procedures

The contents of the course were validated by the supervisor MD according to the essential information presented by Cochrane Collaboration and all related international publications. After that, the course videos were uploaded to the learning management system of the Syrian Virtual University. The web-based EBM course was designed as a 1-group pretest and posttest study. Replies to the electronic registration form expired after 72 hours. Then, participants were asked to answer the pretest. After that, they were provided with the login data to access the web-based course on the learning management system. On the first day, all asynchronous sessions were available for the participants so that they could attend the web-based course according to their own pace and schedule. On the second day, reading materials were provided to the participants, which included the presentations and the scientific resources used to develop these presentations. The reading materials provided more in-depth information, and participants were informed as such. On the third day, an encouraging message and a reminder of the deadline (ie, the fourth day) was sent to participants. On the fourth and last day, the posttest assessment was shared with the participants along with an encouraging message to fill it even if they did not attend all the sessions. Participants were kept motivated during these 4 days by daily messaging, explaining the significance of this web-based course and its benefits to their practice and the health care system. Further, they were constantly encouraged to share their opinions and suggestions about the web-based EBM course. The degree of commitment of the participants and interest in the EBM course were monitored by the researchers (YK and MD) by observing and counting the number of downloads, videos watched, and duration of access to the course through a specific tracking system related to the Syrian Virtual University platform in collaboration with the technical support staff in the university without violation of private data and information of participants.

### Statistical Analysis

All data obtained from the pretests and posttests were retrieved and put into Excel 2016. Paired 1-tailed *t* test was used to analyze the differences between the findings of the participants in the pretest and posttest. *P*<.05 was used to determine the statistical significance threshold. 

### Ethics Approval

This study was reviewed and approved by the ethics approval committee of the Syrian Virtual University on November 15, 2021 (ID 2083/0). Informed consent was obtained from all potential participants through the electronic registration form, wherein they were informed that the data shared for the purpose of this research would be kept confidential and would solely be used for this research.

## Results

### Characteristics of the Participants

Nineteen potential participants filled out the electronic registration form. Eight potential participants did not meet the inclusion criteria, and the pretest was sent to the remaining 11 participants (7 men and 4 women). Eight of them were dentists, 2 of them were physicians, and 1 was a pharmacist. The majority of the participants (9/11, 82%) graduated from Tishreen University, 1 graduated from Aleppo University, and 1 graduated from the International Private University for Science and Technology in Damascus. All of them were studying for their master’s degree in their fields. Table 2 shows the demographic characteristics of the participants in detail. Ten participants filled out the pretest, while 7 completed the final test.

**Table 2:** Demographic characteristics of the participants.

Characteristic		Participants (n)
**Gender**		
	Female	4
	Male	7
**Age**		
	25 years	1
	26 years	6
	27 years	2
	31 years	1
	40 years	1
**Graduation** **year**		
	2021	1
	2020	1
	2019	2
	2018	4
	2017	1
	2014	1
	2004	1
**Medical specialty**		
	Dentistry	8
	Medicine	2
	Pharmacy	1
**Master’s study**		
	Medical education	4
	Quality assurance	1
	Cosmetic dentistry	1
	Prosthodontics	1
	Oral maxillofacial surgery	1
	Otolaryngology	1
	General surgery	1
	Microbiology, Hematology, and Immunology	1
**Participants’ self-perceived expertise in evidence-based medicine (scale of 1-10; 1: novice, 10: expert)**		
	1	1
	3	2
	4	1
	5	2
	7	2
	8	3

### Efficiency and Ease of Use of the Web-Based Course

Seven participants filled out the posttest. The majority of the participants agreed on the positive statements regarding the effectiveness and ease of use of the web-based course. Table 3 shows the statements and the number of participants who agreed to them.

The overall satisfaction with the web-based course was high, as 4 participants rated their satisfaction as 9 out of 10, while the other 3 participants rated their satisfaction as 10. Most of the participants attended all the asynchronous web-based sessions, while only 2 could not attend the last one. Most of them tried to solve the PICO task, and only 1 participant did not attempt to do so. Further, all participants agreed on receiving future communications in case another web-based course was developed in the future. The practical training session and the PICO format session in the second learning unit were the most interesting and useful for 4 participants. Two of them found that the entire training program was useful and interesting, while 1 participant liked the topics of the third and fourth educational units. As for the least interesting and useful aspect, 3 participants answered “nothing” on the web-based course being not interesting or useful. Two of them commented that having many definitions and terms affected their enjoyment and clarity of the learning process. Only 1 participant commented that the first lesson of the third unit was the least interesting and useful. Further, the web-based course was feasible, as the design of its materials and presenting them in asynchronous web-based sessions were free of charge.

**Table 3:** Participants who agreed with the statements used to test the efficacy and ease of use of the web-based course.

Statement	Agree/totally agree (n=7)
The objective of the web-based course was clear.	7
The content of the web-based course was understandable.	7
The web-based course met my expectations.	7
The web-based course offered me sufficient information for an introductory course.	7
The content of the web-based course matched my educational level.	7
I will be able to use what I learned in this web-based course.	7
The content of the web-based course fits to my work setting.	5
This web-based course will help me to better practice evidence-based medicine.	7
This web-based course will help me to improve the quality of my work.	7
The difficulty level of the web-based course was appropriate.	6
The web-based course was presented in a clear logical manner.	7
The web-based format was a good way for me to learn evidence-based medicine.	7
I enjoyed taking the web-based course.	7
I would like to take more of this kind of web-based courses.	7

### Knowledge, Skills, and Attitudes of the Participants Regarding EBM

Only the results of the participants who completed both the pretest and posttest assessment were included. The data were exported from the Google form in Excel format. Paired 1-tailed Student *t* test was performed 3 times to each group of the data representing each of the 3 sections of the assessment. The results showed that the web-based course is effective in terms of improving EBM competencies. The average score of the participants in the EBM knowledge section was clearly improved from 8.4 to 15 after attending the web-based course—an improvement that was statically significant (*P*=.002). Although the increase in the average score of the participants for EBM skills between the pretest and the posttest was quite small (from 3.1 to 4.4), it was still a statistically significant improvement (*P*=.03). The average score of the participants’ attitudes toward EBM was improved from 66.5 to 79.5 among medical graduates who did not receive EBM training during their undergraduate years, which was also statistically significant (*P*=.001). The standard deviation, however, was always greater in the posttest scores when compared with that in the pretest scores, with the knowledge section having the greatest difference between the pretest and posttest (Table 4).

**Table 4:** Participants’ scores in the pretest and posttest.

Section			X^a^_1_		X_2_		X_3_		X_4_		X_5_		X_6_		X_7_		Mean (SD)		*P* value	
**Knowledge section (x^b^/20)**																				.002
	Pretest	7.00		6.00		8.00		10.00		9.00		9.00		10.00		8.4 (1.5)				
	Posttest	18.00		14.00		14.00		18.00		15.00		7.00		19.00		15 (4)				
**Skills section (x/8)**																				.03
	Pretest	3.00		1.00		3.00		4.00		3.00		3.00		5.00		3.1 (1.2)				
	Posttest	6.00		3.00		3.00		7.00		4.00		2.00		6.00		4.4 (1.9)				
**Attitudes section (x/100)**																				<.001
	Pretest	66		68		65		63		74		60		70		66.5 (4.6)				
	Posttest	83		80		79		75		77		71		92		79.5 (6.6)				

^a^X indicates participant number.

^b^x indicates participant’s score.

## Discussion

### Main Findings

Web-based medical education can provide many learning and training opportunities for medical students. Several web-based learning modalities have shown to improve clinical training, such as simulation technology, synchronous learning delivery, and web‑based or videoconferencing for standardized patient‑based training [20]. However, in Syria, despite the growing demand for web-based medical learning resources, e-learning is still in its early stage [21]. Moreover, since English is a language barrier in Syria, Syrian clinical practitioners are unlikely to benefit from the large number of already existing English web-based learning resources [9]. Furthermore, the current unstable political situation in Syria makes it hard to organize a traditional course [17].

In this study, our web-based EBM course is the first one in Syria, to the best of our knowledge, to rely entirely on asynchronous web-based sessions and to be designed and delivered in Arabic by a peer of the targeted study population. Additionally, this is the first web-based EBM learning course in Syria that targeted clinical practitioners who did not have the opportunity to be introduced to EBM during their undergraduate studies, and our study reveals the opportunities that e-learning offers in a low-resource and an unstable environment. The goal of our study was to test the feasibility of this web-based course and to develop an effective method to promote EBM practice in Syria. This goal could be achieved because a significant improvement in EBM learning was observed after the clinicians attended our web-based course that was designed using open-sourced software.

Kirkpatrick’s model for the evaluation of training programs represent one of the most comprehensive strategies for evaluating organizational training [22]. Our web-based EBM course was found to be successful when evaluated on the basis of the adaptation of Kirkpatrick’s model to the e-learning environment [22]. Course assessment showed a clear satisfaction, enjoyment, improvement in participants’ learning, and willingness to apply what they learned after attending this course. Our web-based EBM course was not only feasible but also extremely useful during the COVID-19 pandemic, wherein social distancing regulations had to be followed. Further, asynchronous web-based learning was found to be advantageous for busy clinical practitioners with crowded schedules, as they were able to attend approximately all sessions in just 4 days.

This web-based course focused on practitioners acquiring the needed skills to practice EBM in clinical settings. Given the fact that there is preappraised evidence available online, this web-based course focused on the first 2 sets of EBM skills in formatting a clinical question and searching the literature online. For that purpose, the PICO task was designed and presented to be solved voluntarily by the participants followed by general feedback on that task. In addition to designing a practical session, the process of searching a web-based search engine was presented. Moreover, a population of clinical practitioners was targeted to try to make this web-based EBM course clinically integrated, as it was found that EBM skills can be best fostered during ward rotation [23]. These were found to be effective techniques, as EBM skills were significantly improved in this 70-minute web-based course, which was provided over a period of 4 days. Further, the electronic registration form with its content worked as an effective advertising tool for the web-based course, as more than half of the potential participants rated their self-perceived expertise regarding EBM as 5 or more on the scale of 1-10.

### Limitations and Suggestions

This was a pilot study conducted with only a few medical graduates. Thus, the effectiveness of this web-based learning method could not be determined among older clinical practitioners who often have technophobia and will therefore find web-based learning to be a challenging task [24]. Further, it is assumed that only participants who were interested in learning the concept of EBM had joined, which could raise bias in the results. However, future studies will be performed on a more diverse sample, and critical appraisal tasks will be designed with general feedback to be delivered in the same technique as done for the PICO task to reveal more benefits of the e-learning in terms of EBM.

In the future, it is possible to adopt a peer-review strategy to communicate feedback provided by participants to their peers’ work before introducing the general feedback and studying the effectiveness of such an approach and its reflection on the participants’ EBM skills. Specifically, the peer’s role was found to be effective in improving EBM skills, which can overcome the barrier of the limited number of EBM experts in Syria [18]. The effect of including a test after each asynchronous session is also worth studying, as it enhances the retention rates to make the participants feel more engaged [25]. Since this web-based course was found to be effective for the introduction of EBM, it can be disseminated further as part of a larger continuous medical education program to make all clinical practitioners familiar with the concept of EBM. This web-based EBM course can also be presented at the undergraduate level as an introductory session before proceeding to the newly developed official curriculum.

### Comparison With Prior Work

The results obtained in our study are consistent with the previously obtained findings [11,12,26] that e-medical education is effective and feasible for developing EBM knowledge and improving practitioners’ attitudes toward EBM. Previous studies [11,12,26] have focused on comparisons between e-learning and lecture-based methods, but they have not shown the effects of e-learning on EBM skills. A study showed that e-learning was found to be effective for developing EBM skills; however, in the 8-week-long e-module in that study, a tutor with experience in teaching EBM was responsible for providing feedback on the multiple exercises and assignments incorporated in that e-module [13]. Another study found that e-learning can improve EBM skills. However, that e-module contained summative, formative, and individual assessments [27]. In this study, EBM skills were improved by providing general feedback on a given task. Another study showed that a 90-minute introductory EBM e-module, which consists of set of screens without explanation for those screens, failed to improve EBM skills but succeeded in improving EBM knowledge and practitioners’ attitudes [19]. Our web-based course improved EBM skills significantly in 70-minute video recordings of explanations for a set of presentations. In Syria, 2 other studies [8,18] were found to be associated with EBM teaching: the first one was based on traditional teaching methods by experienced EBM instructors for the undergraduate population [8], and the second one relied on both asynchronous and synchronous sessions of web-based learning and the web-based course was delivered over 6 weeks [18].

### Conclusions

Transforming health care in Syria from relying on expert opinion to relying on the latest scientific evidence starts by teaching the concept of EBM at the undergraduate level and continuing the medical education trainings throughout the study program. Our study showed that asynchronous web-based medical education introduces the concept of EBM effectively, provides adequate EBM skill training, and promotes the positive attitudes of Syrian clinical practitioners toward EBM. Further, this web-based course was designed with simple and accessible means, and it was highly feasible and well-received by the participants.

## Appendix

Multimedia Appendix 1 First lesson in the first learning unit.

Multimedia Appendix 2 Second lesson in the first learning unit.

Multimedia Appendix 3 Third lesson in the first learning unit.

Multimedia Appendix 4 First lesson in the second learning unit.

Multimedia Appendix 5 Second lesson in the second learning unit.

Multimedia Appendix 6 Third lesson in the second learning unit.

Multimedia Appendix 7 First lesson in the third learning unit.

Multimedia Appendix 8 Second lesson in the third learning unit.

Multimedia Appendix 9 Third lesson in the third learning unit.

Multimedia Appendix 10 First lesson in the fourth learning unit.

Multimedia Appendix 11 Second lesson in the fourth learning unit.

## Abbreviations

EBM: evidence-based medicine
PICO: Patient, Intervention, Comparison, and Outcome
