# Supplementary material for: The First Asynchronous Online Evidence-Based Medicine Course for Syrian Health Workforce: Effectiveness and Feasibility Pilot Study
Source: JMIR Form Res. 2022 Oct 25;6(10):e36782. doi: 10.2196/36782 (PMC9644249; doi:10.2196/36782)
Supplement: Multimedia Appendix 1 [file formative_v6i10e36782_app1.pptx]

## Slide 1
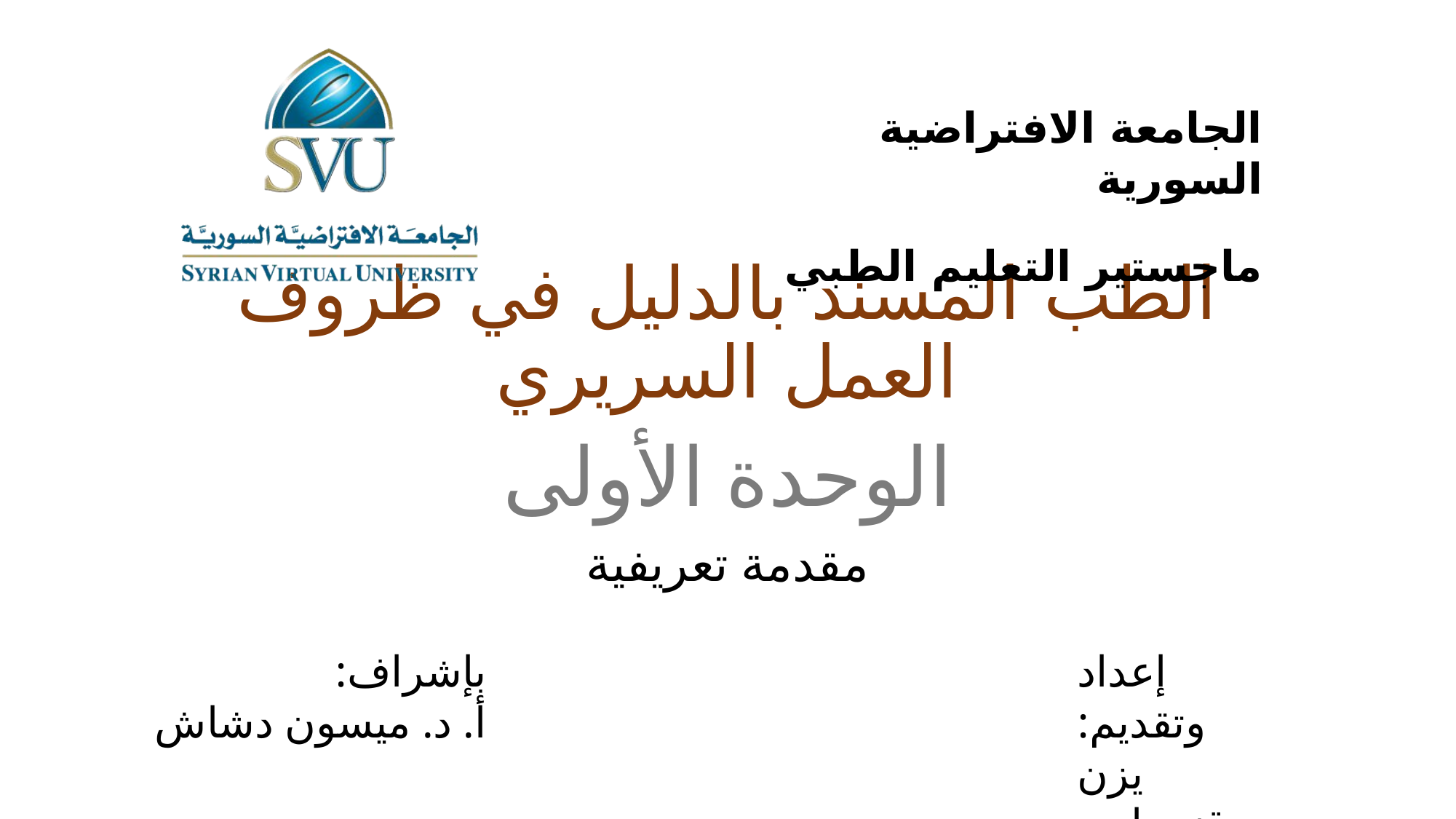

الجامعة الافتراضية السورية
ماجستير التعليم الطبي
# الطب المسند بالدليل في ظروف العمل السريري
الوحدة الأولى
مقدمة تعريفية
إعداد وتقديم:
يزن قنجراوي
بإشراف:
أ. د. ميسون دشاش

## Slide 2
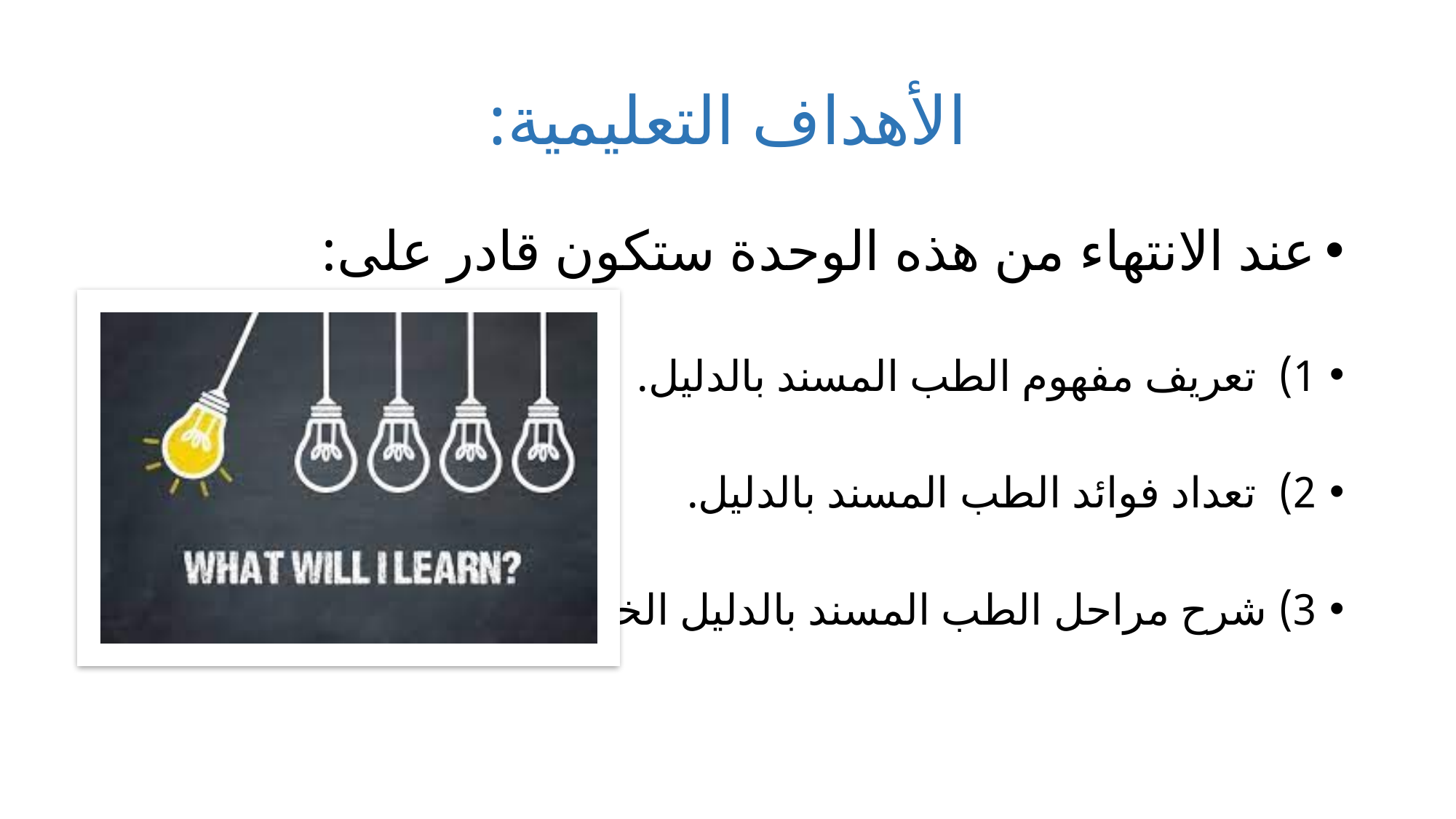

# الأهداف التعليمية:
عند الانتهاء من هذه الوحدة ستكون قادر على:
1) تعريف مفهوم الطب المسند بالدليل.
2) تعداد فوائد الطب المسند بالدليل.
3) شرح مراحل الطب المسند بالدليل الخمسة.

## Slide 3
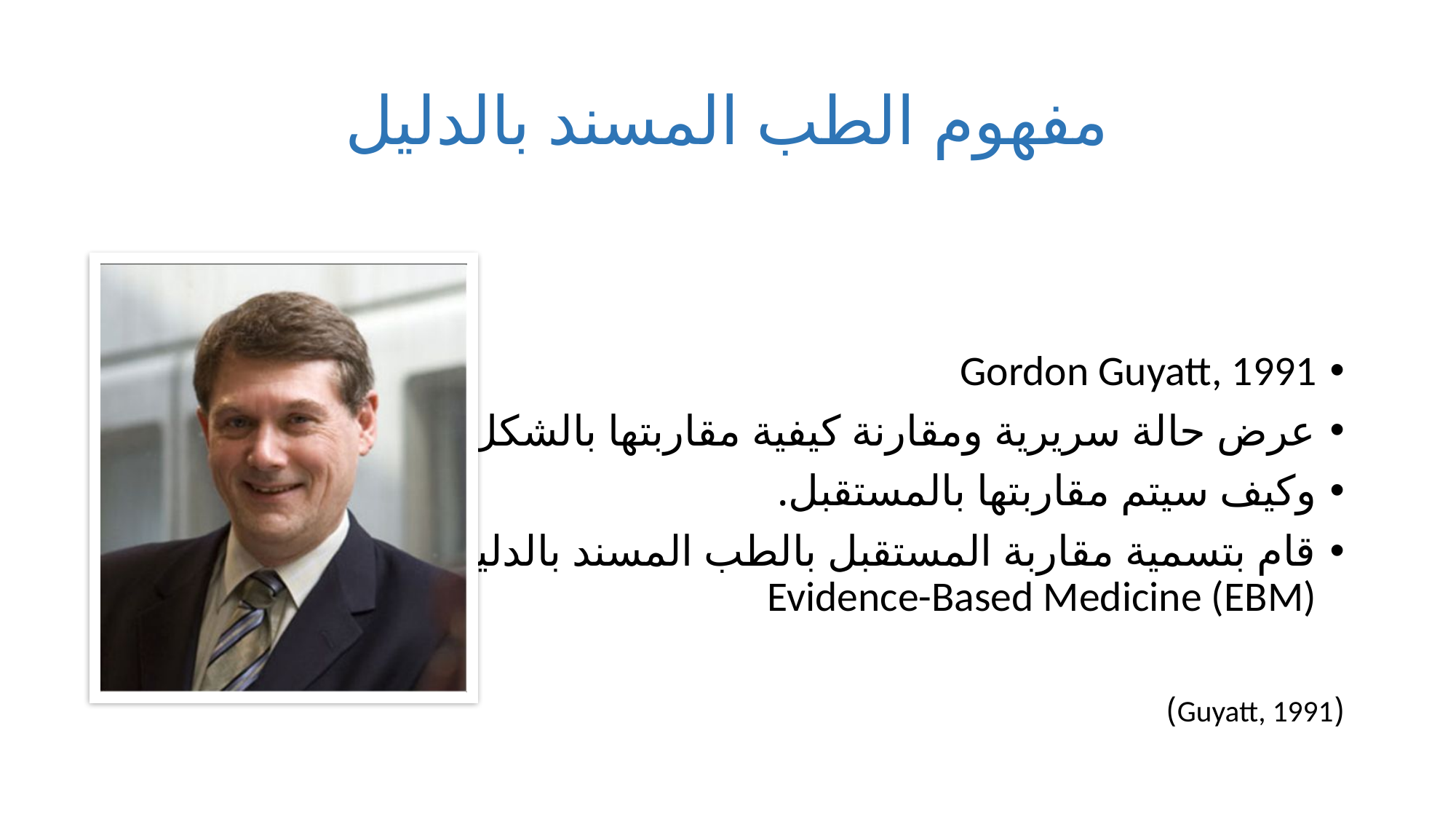

# مفهوم الطب المسند بالدليل
Gordon Guyatt, 1991
عرض حالة سريرية ومقارنة كيفية مقاربتها بالشكل التقليدي
وكيف سيتم مقاربتها بالمستقبل.
قام بتسمية مقاربة المستقبل بالطب المسند بالدليل
Evidence-Based Medicine (EBM)
(Guyatt, 1991)

## Slide 4
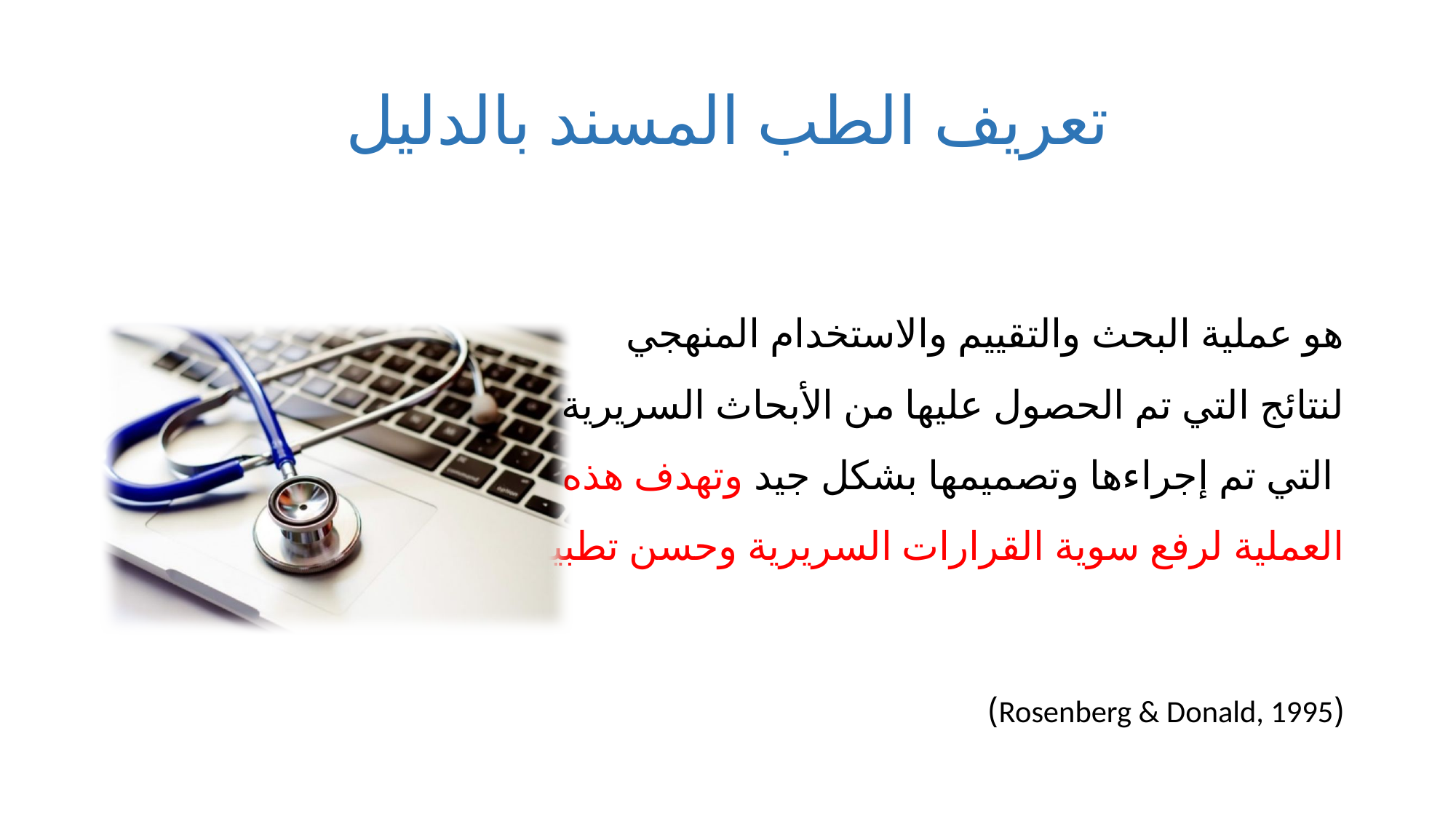

# تعريف الطب المسند بالدليل
هو عملية البحث والتقييم والاستخدام المنهجي
لنتائج التي تم الحصول عليها من الأبحاث السريرية
 التي تم إجراءها وتصميمها بشكل جيد وتهدف هذه
العملية لرفع سوية القرارات السريرية وحسن تطبيقها.
(Rosenberg & Donald, 1995)

## Slide 5
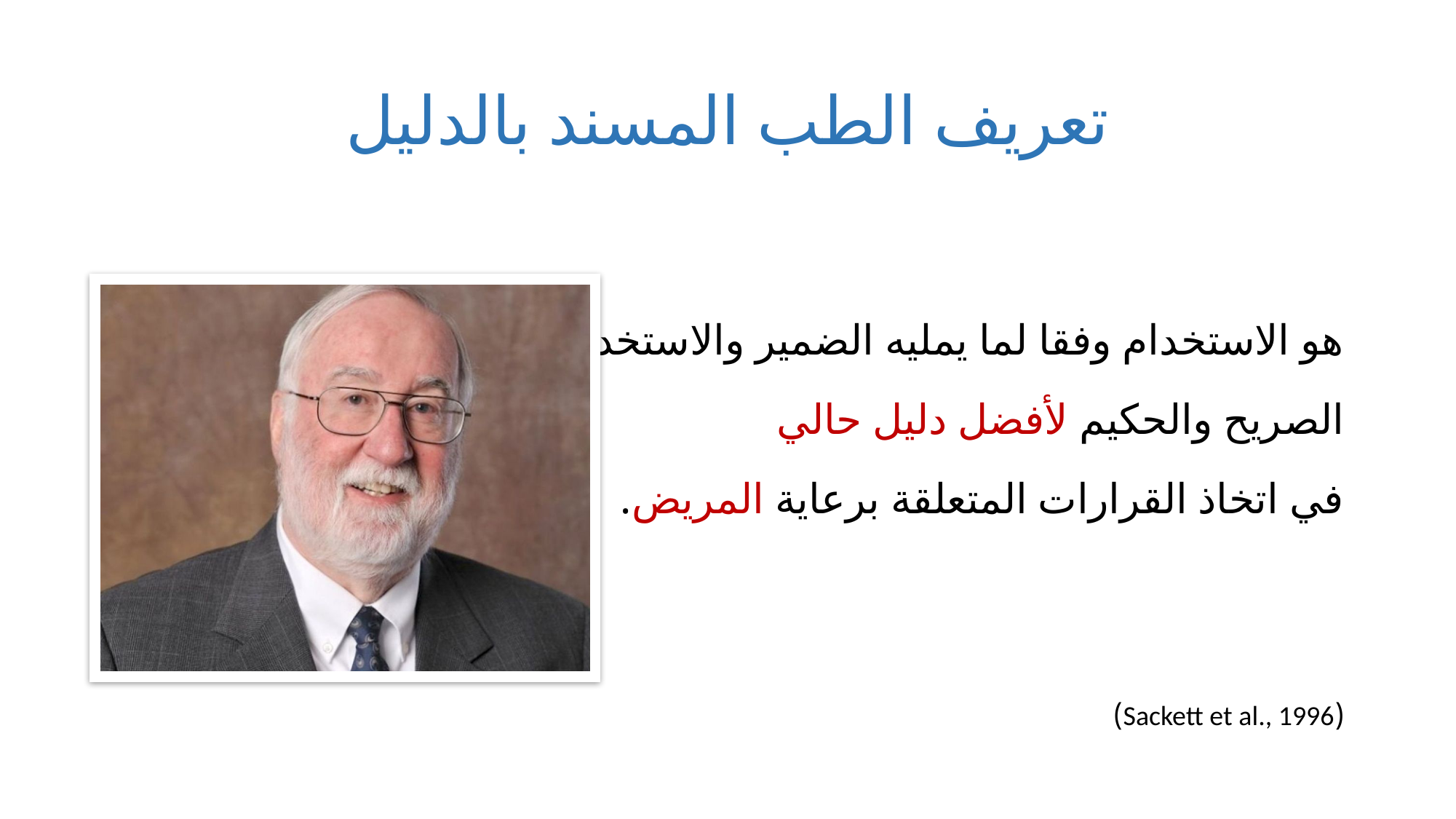

# تعريف الطب المسند بالدليل
هو الاستخدام وفقا لما يمليه الضمير والاستخدام
الصريح والحكيم لأفضل دليل حالي
في اتخاذ القرارات المتعلقة برعاية المريض.
(Sackett et al., 1996)

## Slide 6
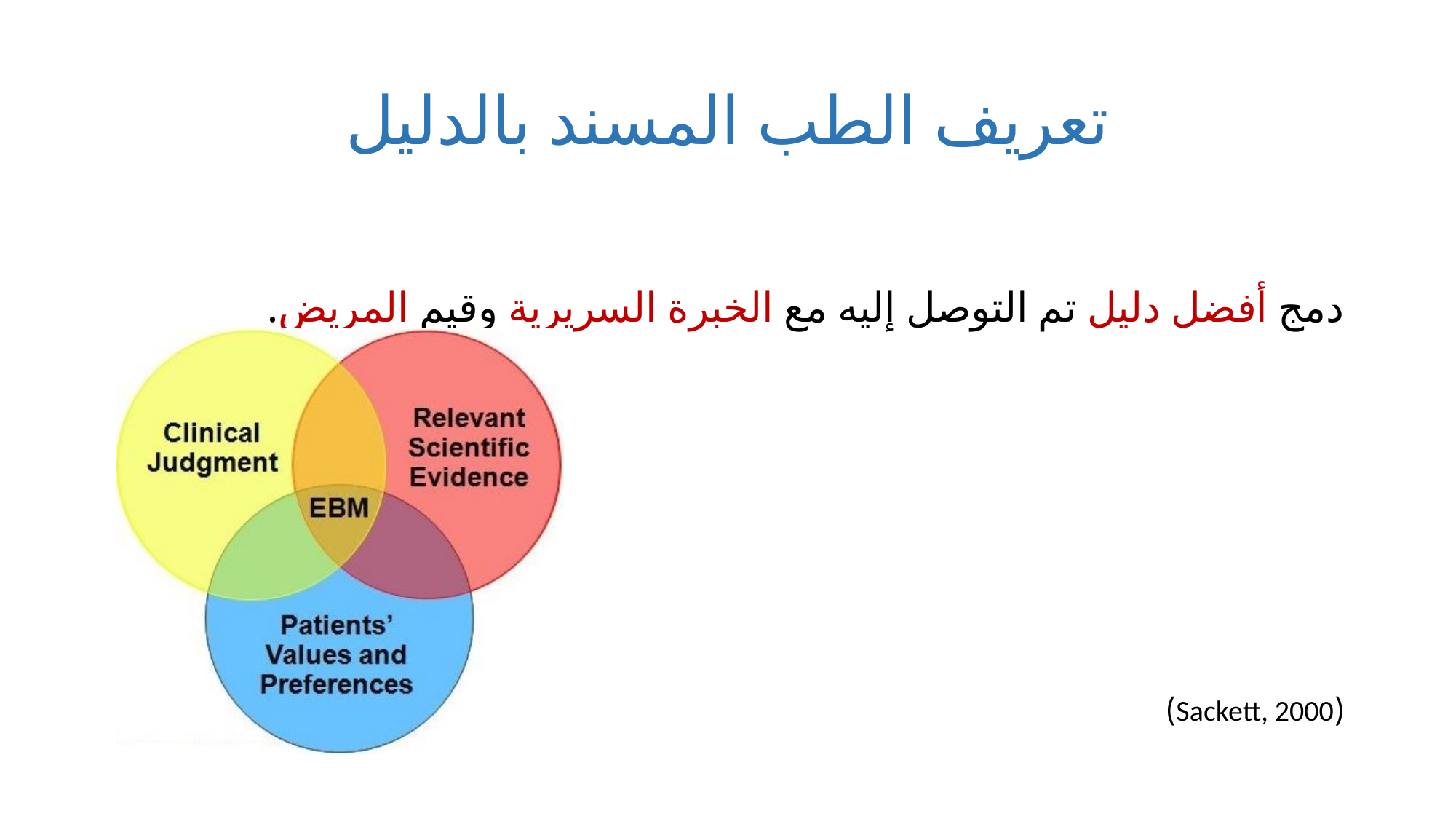

# تعريف الطب المسند بالدليل
دمج أفضل دليل تم التوصل إليه مع الخبرة السريرية وقيم المريض.
(Sackett, 2000)

## Slide 7
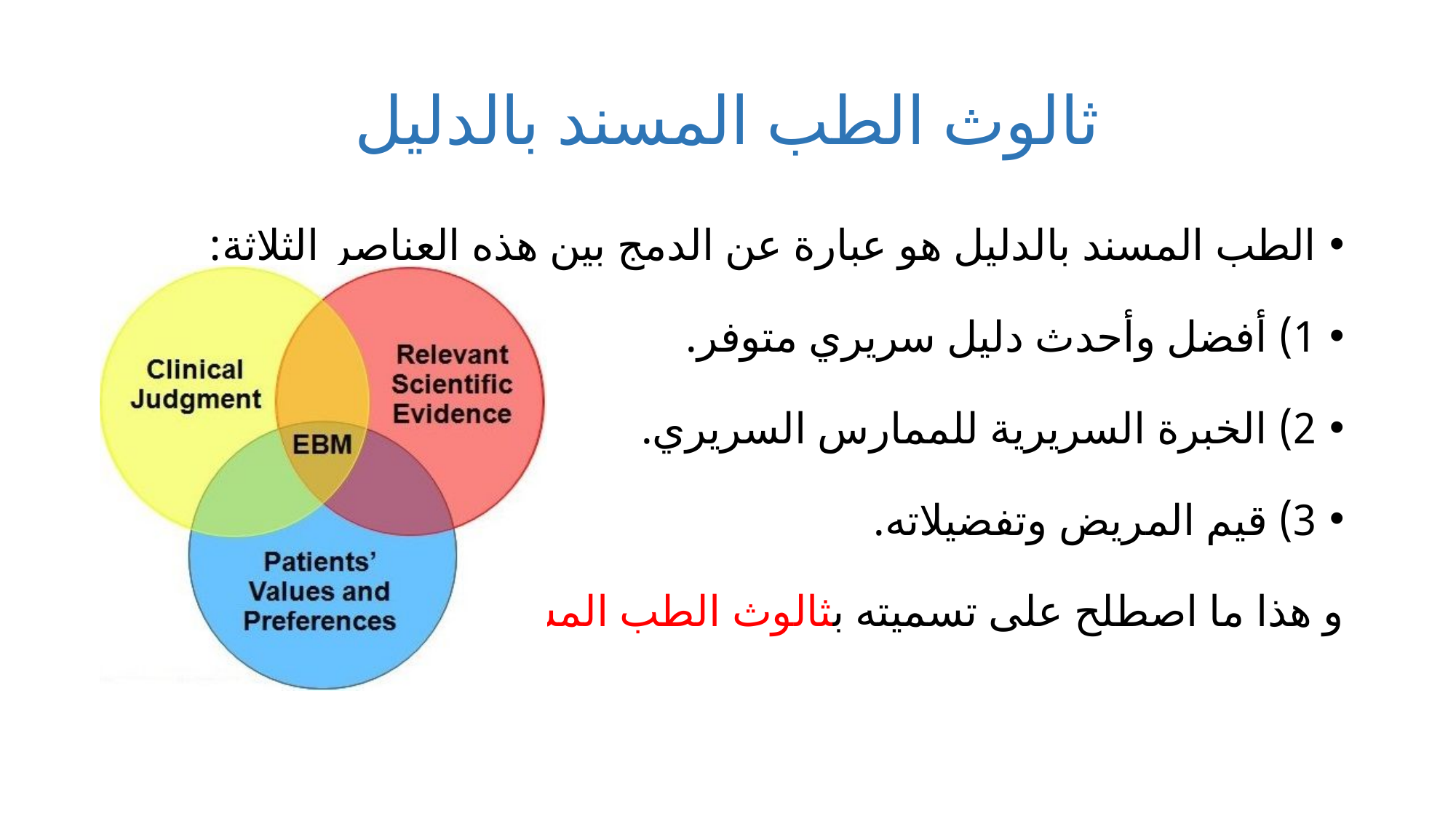

# ثالوث الطب المسند بالدليل
الطب المسند بالدليل هو عبارة عن الدمج بين هذه العناصر الثلاثة:
1) أفضل وأحدث دليل سريري متوفر.
2) الخبرة السريرية للممارس السريري.
3) قيم المريض وتفضيلاته.
و هذا ما اصطلح على تسميته بثالوث الطب المسند بالدليل.

## Slide 8
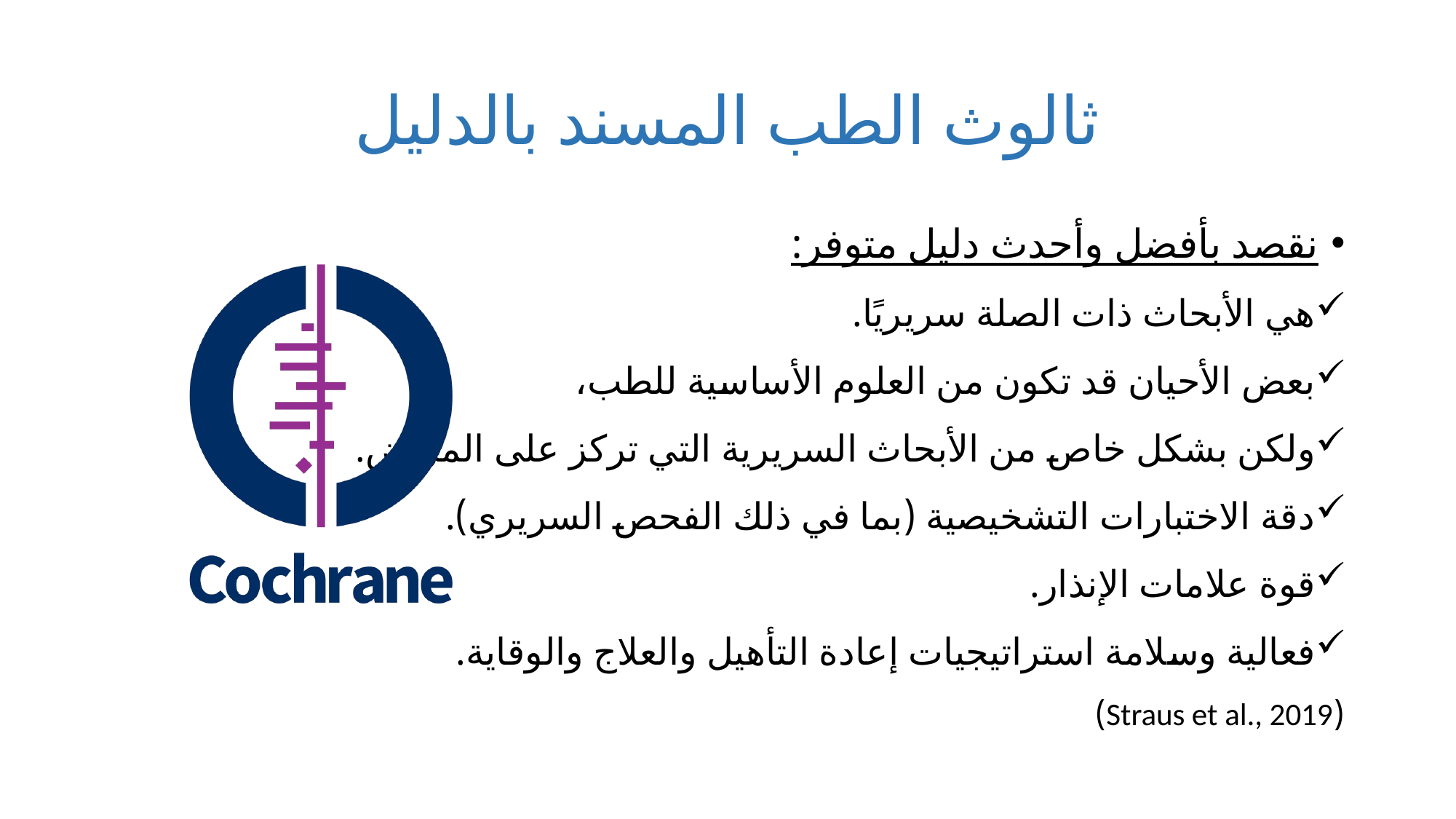

# ثالوث الطب المسند بالدليل
نقصد بأفضل وأحدث دليل متوفر:
هي الأبحاث ذات الصلة سريريًا.
بعض الأحيان قد تكون من العلوم الأساسية للطب،
ولكن بشكل خاص من الأبحاث السريرية التي تركز على المريض.
دقة الاختبارات التشخيصية (بما في ذلك الفحص السريري).
قوة علامات الإنذار.
فعالية وسلامة استراتيجيات إعادة التأهيل والعلاج والوقاية.
(Straus et al., 2019)

## Slide 9
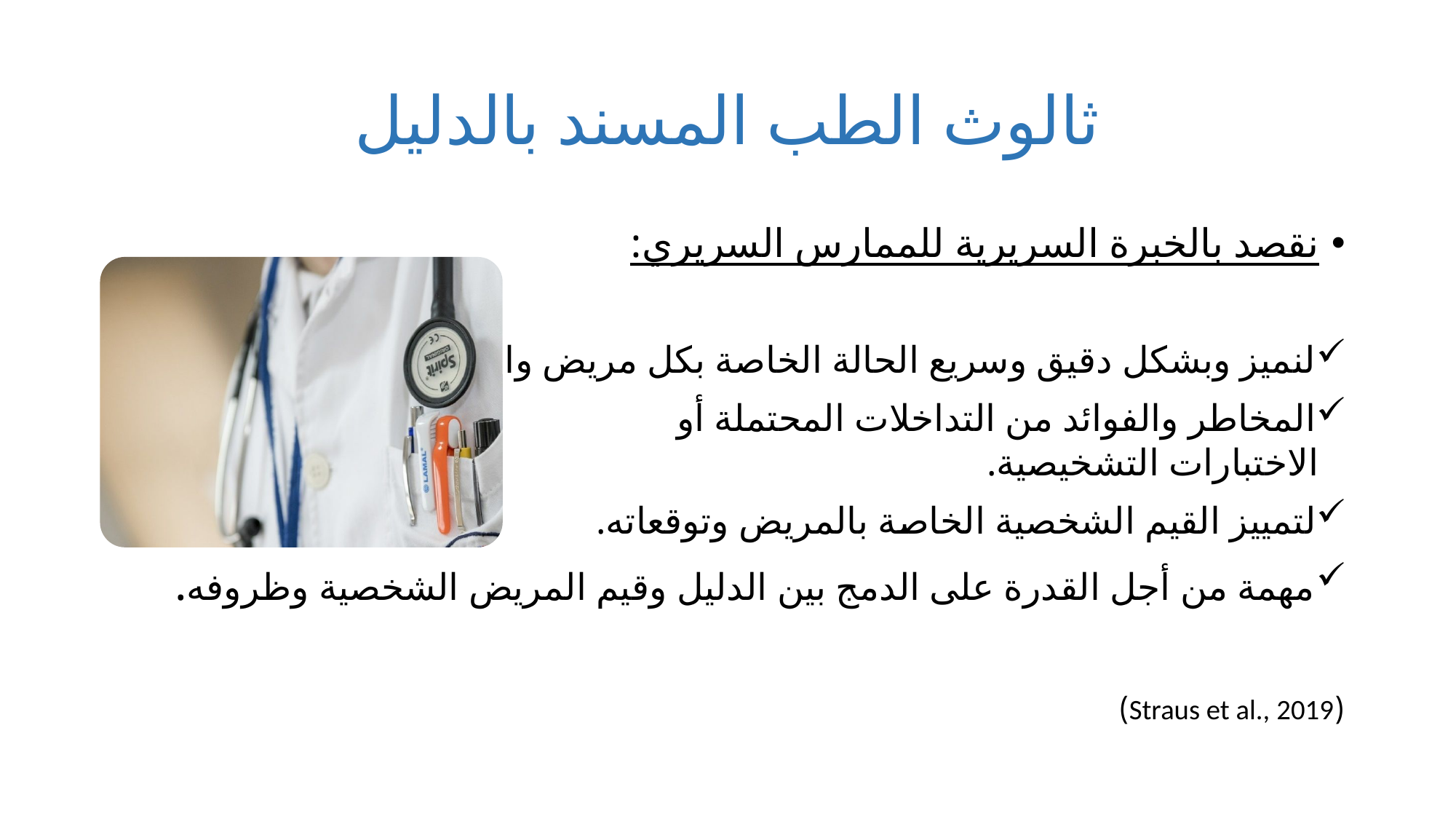

# ثالوث الطب المسند بالدليل
نقصد بالخبرة السريرية للممارس السريري:
لنميز وبشكل دقيق وسريع الحالة الخاصة بكل مريض والتشخيص.
المخاطر والفوائد من التداخلات المحتملة أو
الاختبارات التشخيصية.
لتمييز القيم الشخصية الخاصة بالمريض وتوقعاته.
مهمة من أجل القدرة على الدمج بين الدليل وقيم المريض الشخصية وظروفه.
(Straus et al., 2019)

## Slide 10
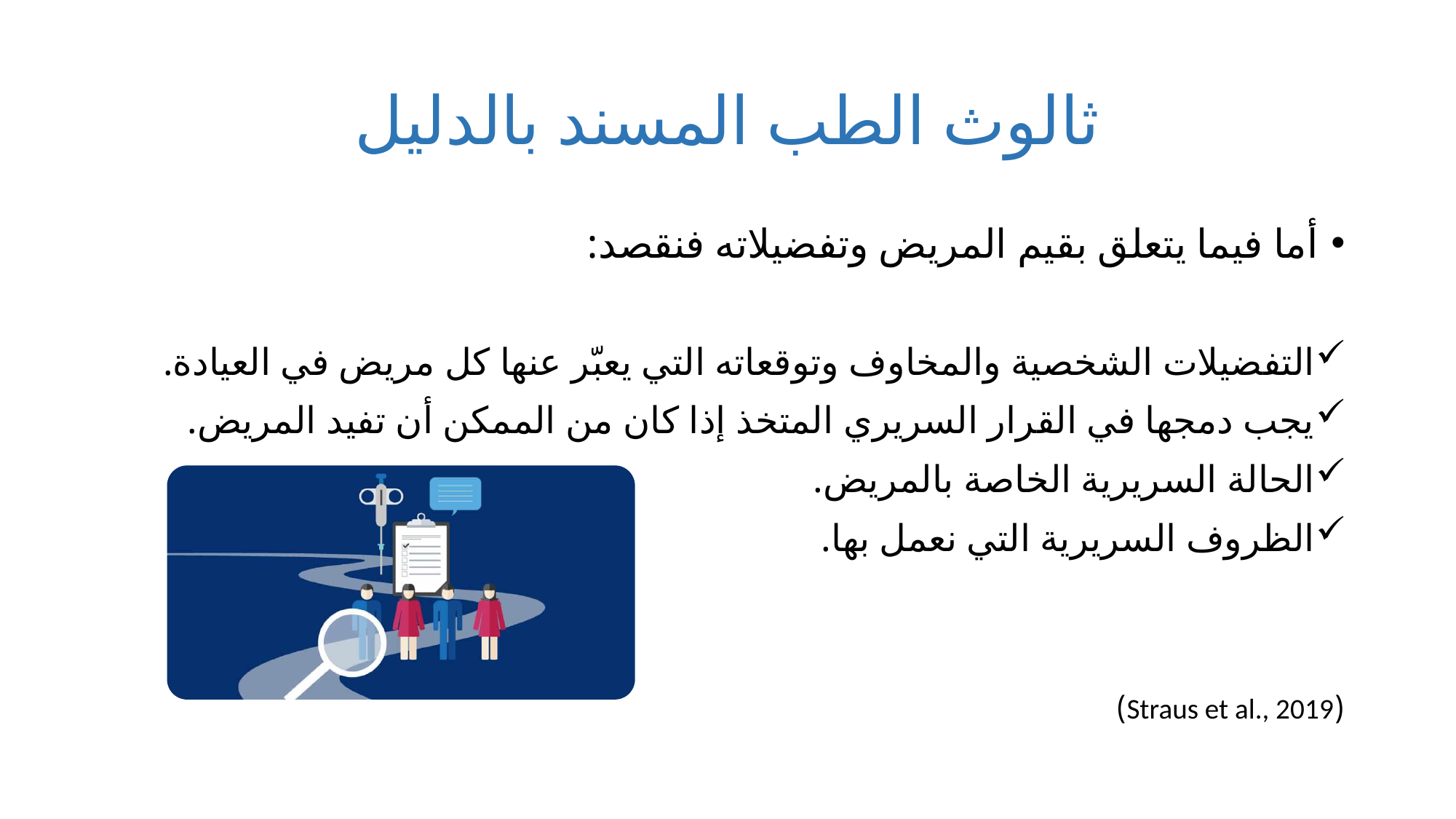

# ثالوث الطب المسند بالدليل
أما فيما يتعلق بقيم المريض وتفضيلاته فنقصد:
التفضيلات الشخصية والمخاوف وتوقعاته التي يعبّر عنها كل مريض في العيادة.
يجب دمجها في القرار السريري المتخذ إذا كان من الممكن أن تفيد المريض.
الحالة السريرية الخاصة بالمريض.
الظروف السريرية التي نعمل بها.
(Straus et al., 2019)

## Slide 11
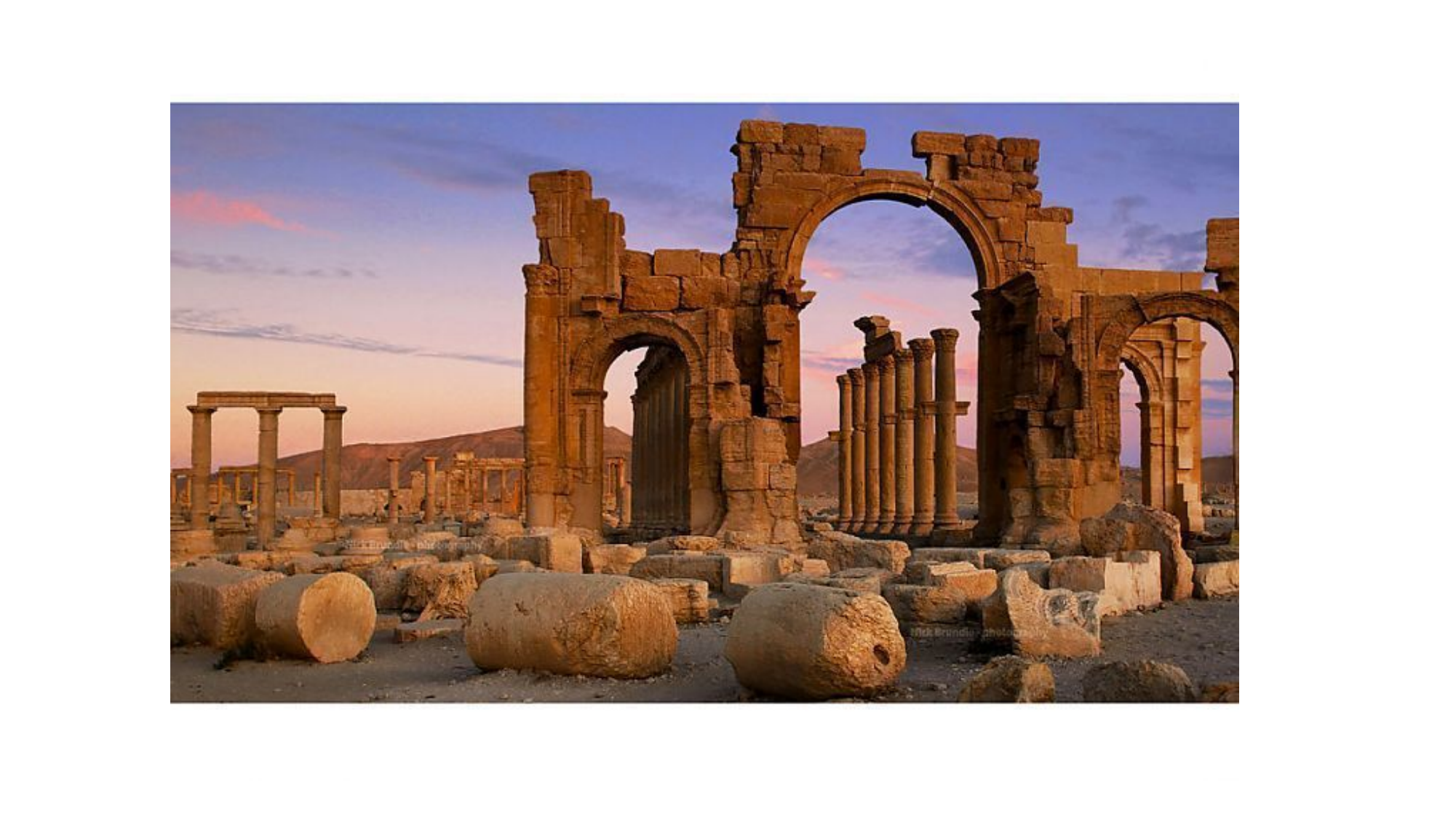

## Slide 12
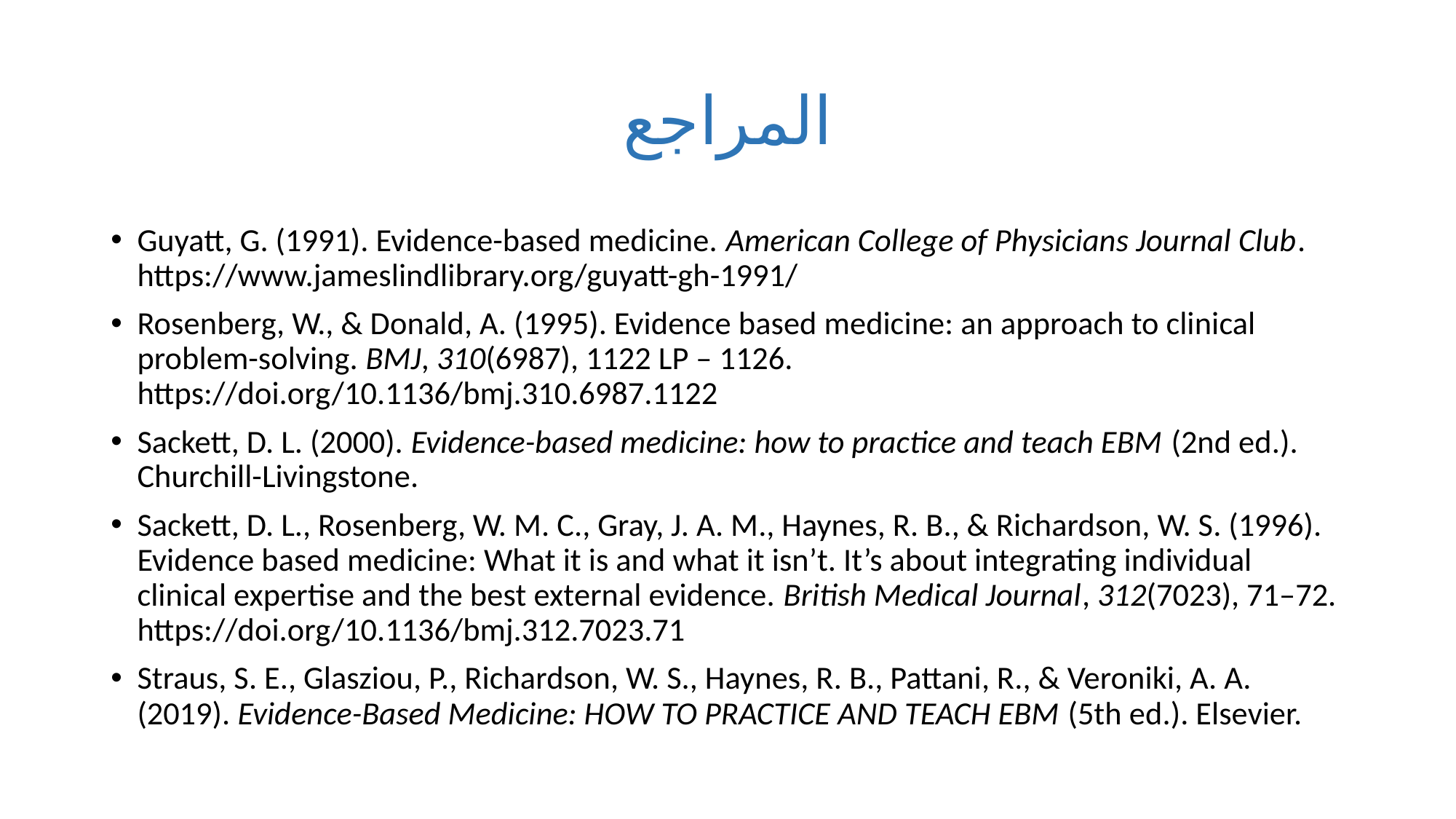

# المراجع
Guyatt, G. (1991). Evidence-based medicine. American College of Physicians Journal Club. https://www.jameslindlibrary.org/guyatt-gh-1991/
Rosenberg, W., & Donald, A. (1995). Evidence based medicine: an approach to clinical problem-solving. BMJ, 310(6987), 1122 LP – 1126. https://doi.org/10.1136/bmj.310.6987.1122
Sackett, D. L. (2000). Evidence-based medicine: how to practice and teach EBM (2nd ed.). Churchill-Livingstone.
Sackett, D. L., Rosenberg, W. M. C., Gray, J. A. M., Haynes, R. B., & Richardson, W. S. (1996). Evidence based medicine: What it is and what it isn’t. It’s about integrating individual clinical expertise and the best external evidence. British Medical Journal, 312(7023), 71–72. https://doi.org/10.1136/bmj.312.7023.71
Straus, S. E., Glasziou, P., Richardson, W. S., Haynes, R. B., Pattani, R., & Veroniki, A. A. (2019). Evidence-Based Medicine: HOW TO PRACTICE AND TEACH EBM (5th ed.). Elsevier.
