# Supplementary material for: The First Asynchronous Online Evidence-Based Medicine Course for Syrian Health Workforce: Effectiveness and Feasibility Pilot Study
Source: JMIR Form Res. 2022 Oct 25;6(10):e36782. doi: 10.2196/36782 (PMC9644249; doi:10.2196/36782)
Supplement: Multimedia Appendix 2 [file formative_v6i10e36782_app2.pptx]

## Slide 1
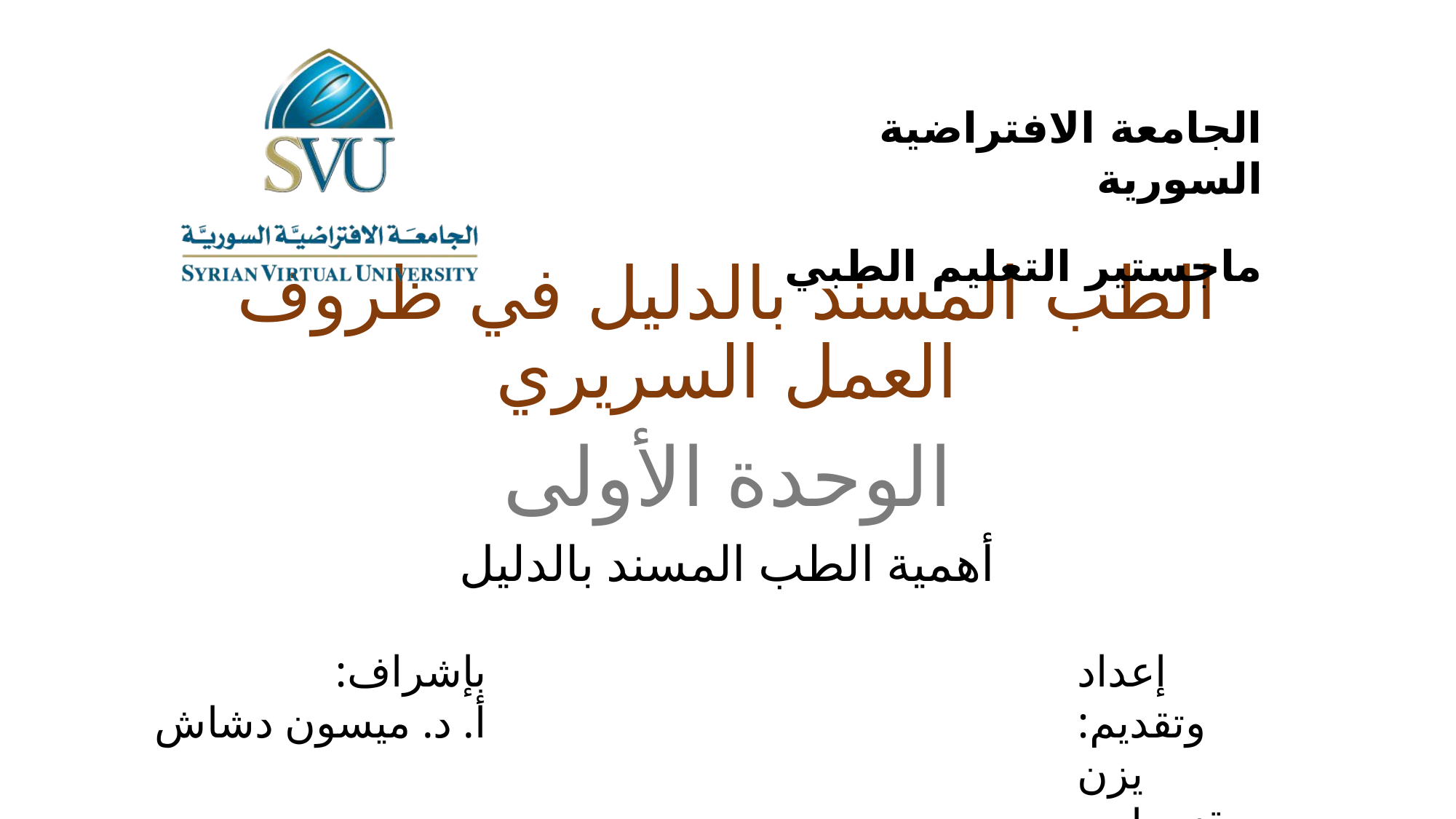

الجامعة الافتراضية السورية
ماجستير التعليم الطبي
# الطب المسند بالدليل في ظروف العمل السريري
الوحدة الأولى
أهمية الطب المسند بالدليل
إعداد وتقديم:
يزن قنجراوي
بإشراف:
أ. د. ميسون دشاش

## Slide 2
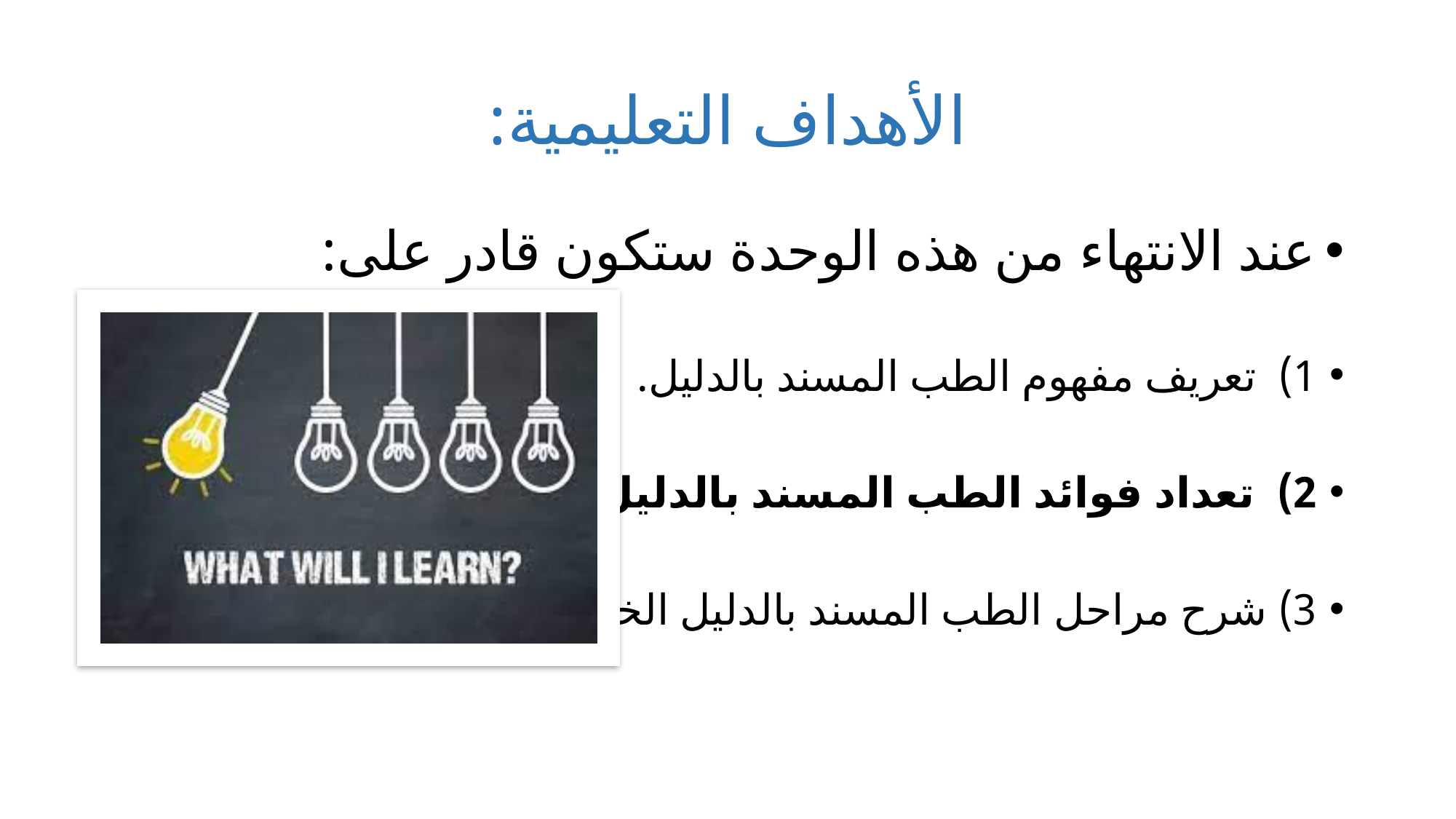

# الأهداف التعليمية:
عند الانتهاء من هذه الوحدة ستكون قادر على:
1) تعريف مفهوم الطب المسند بالدليل.
2) تعداد فوائد الطب المسند بالدليل.
3) شرح مراحل الطب المسند بالدليل الخمسة.

## Slide 3
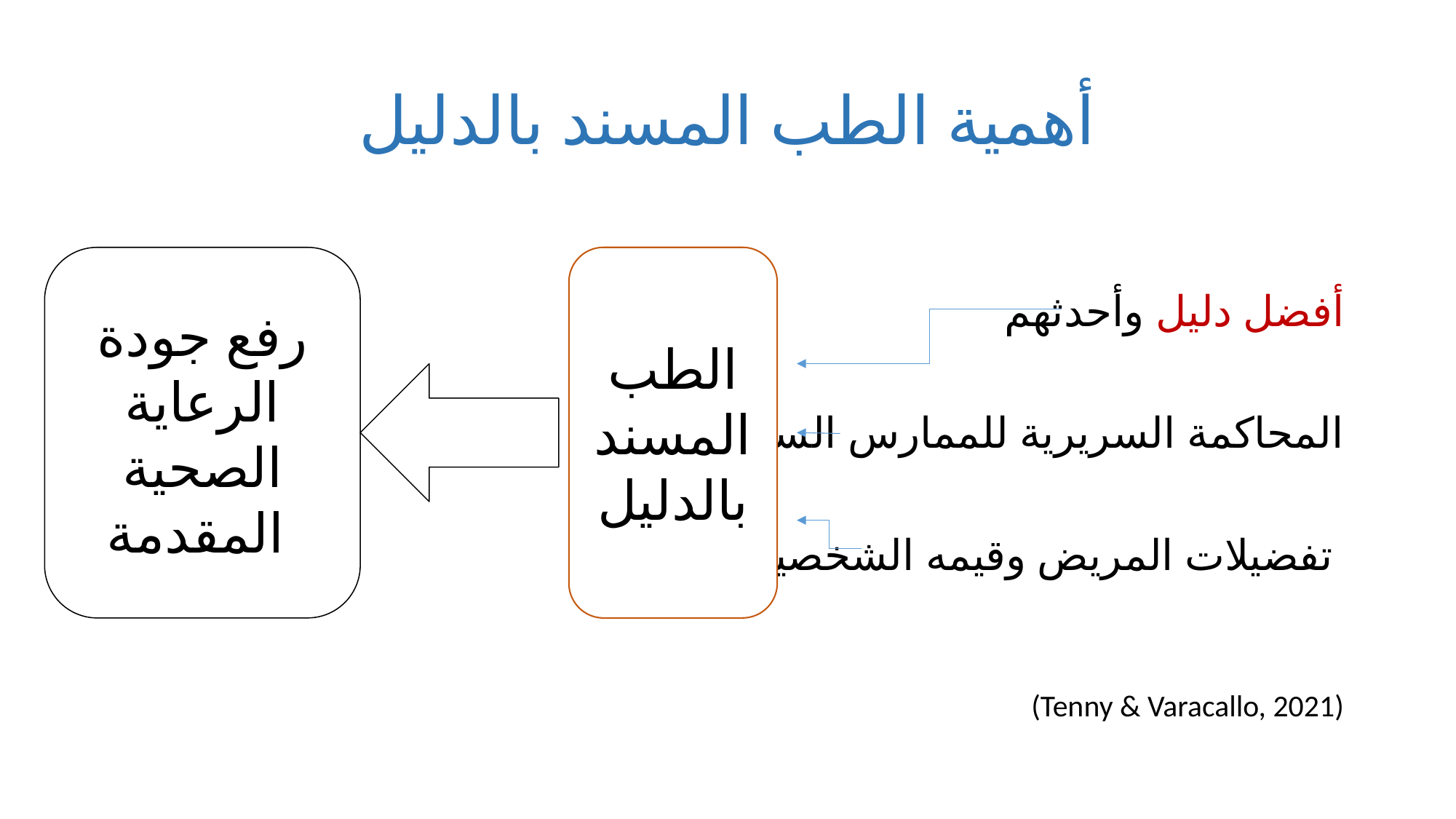

# أهمية الطب المسند بالدليل
أفضل دليل وأحدثهم
المحاكمة السريرية للممارس السريري
تفضيلات المريض وقيمه الشخصية
(Tenny & Varacallo, 2021)
رفع جودة الرعاية الصحية المقدمة
الطب المسند بالدليل

## Slide 4
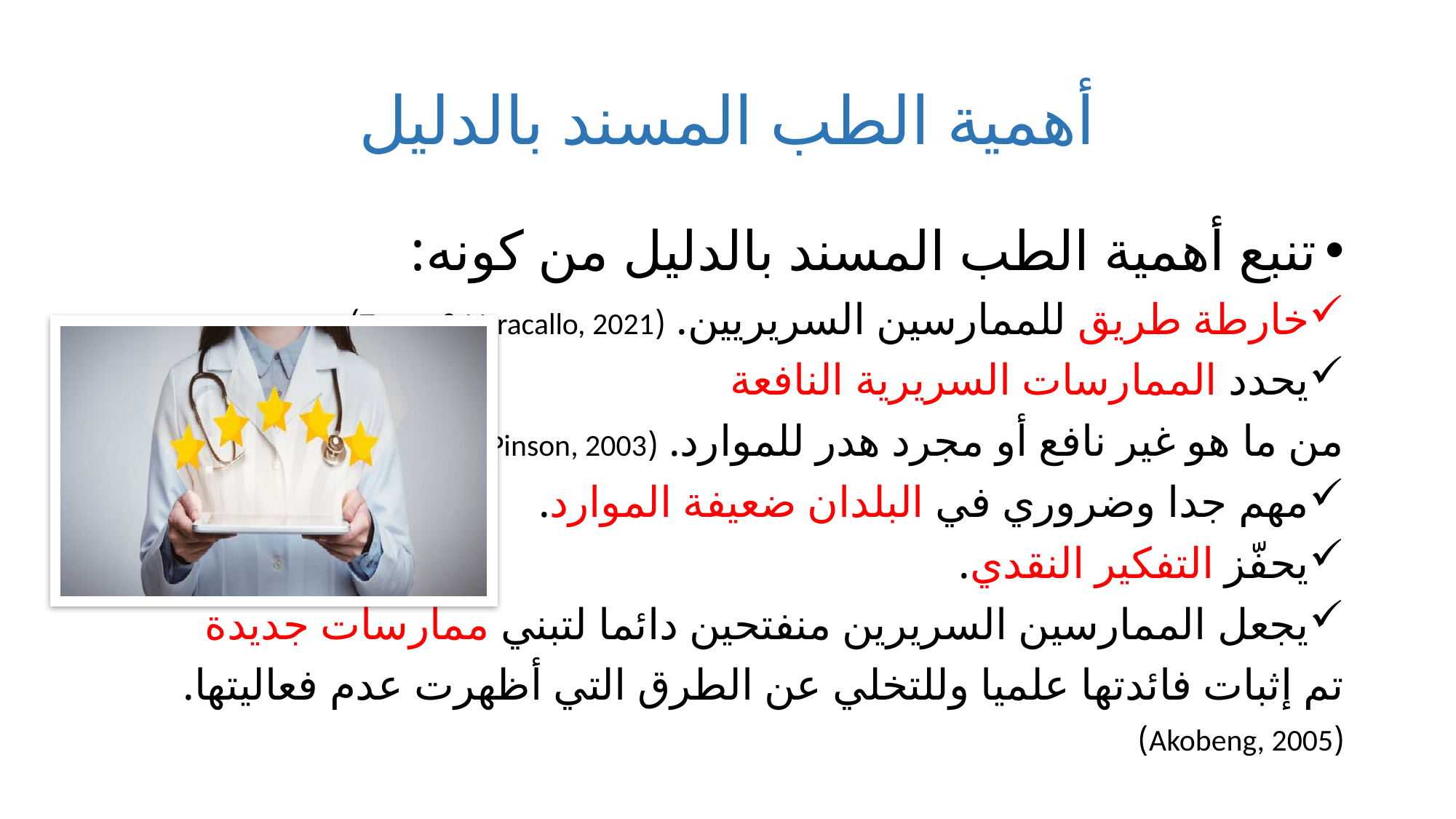

# أهمية الطب المسند بالدليل
تنبع أهمية الطب المسند بالدليل من كونه:
خارطة طريق للممارسين السريريين. (Tenny & Varacallo, 2021)
يحدد الممارسات السريرية النافعة
من ما هو غير نافع أو مجرد هدر للموارد. (Gray & Pinson, 2003)
مهم جدا وضروري في البلدان ضعيفة الموارد.
يحفّز التفكير النقدي.
يجعل الممارسين السريرين منفتحين دائما لتبني ممارسات جديدة
تم إثبات فائدتها علميا وللتخلي عن الطرق التي أظهرت عدم فعاليتها.
(Akobeng, 2005)

## Slide 5
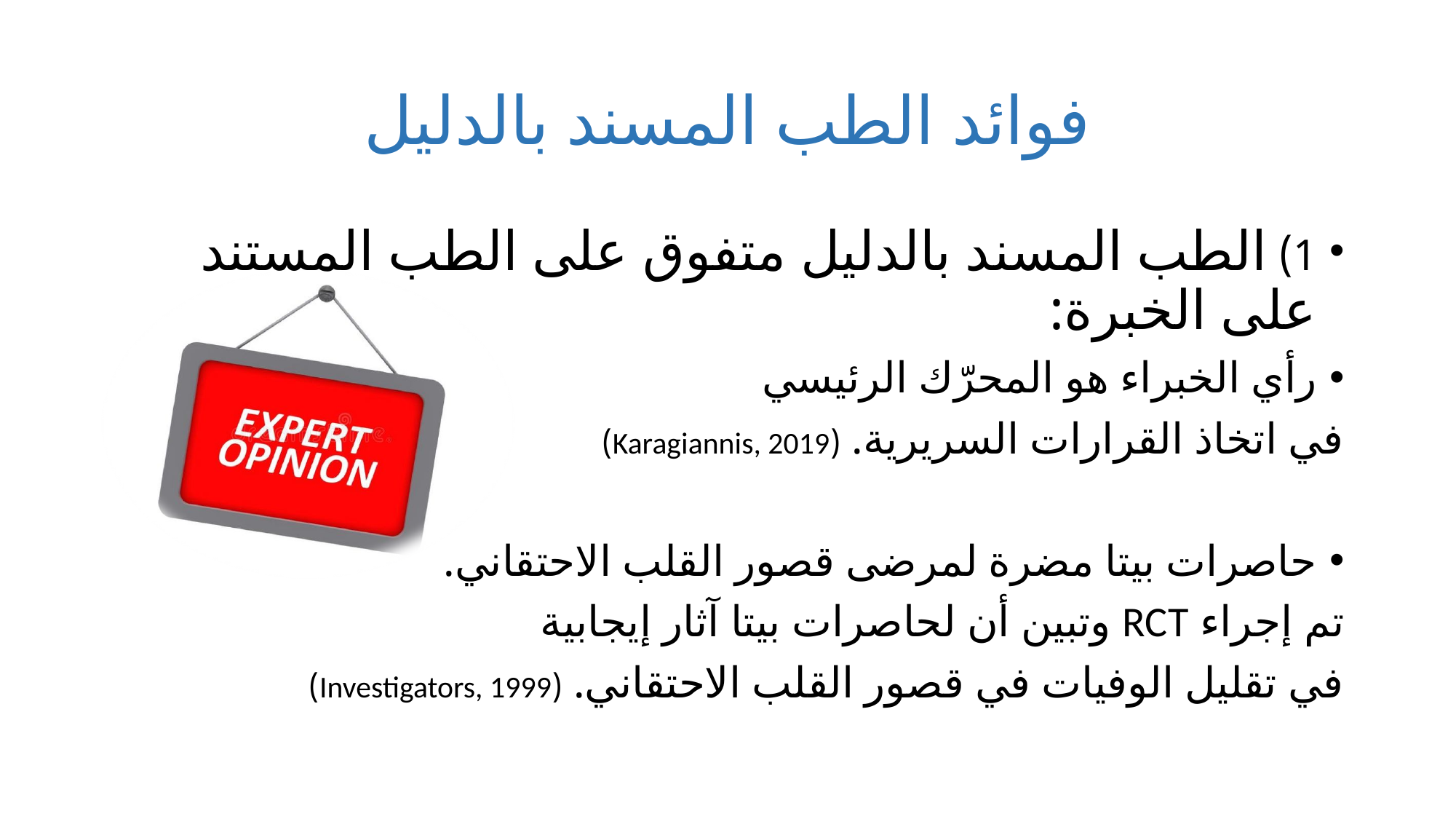

# فوائد الطب المسند بالدليل
1) الطب المسند بالدليل متفوق على الطب المستند على الخبرة:
رأي الخبراء هو المحرّك الرئيسي
في اتخاذ القرارات السريرية. (Karagiannis, 2019)
حاصرات بيتا مضرة لمرضى قصور القلب الاحتقاني.
تم إجراء RCT وتبين أن لحاصرات بيتا آثار إيجابية
في تقليل الوفيات في قصور القلب الاحتقاني. (Investigators, 1999)

## Slide 6
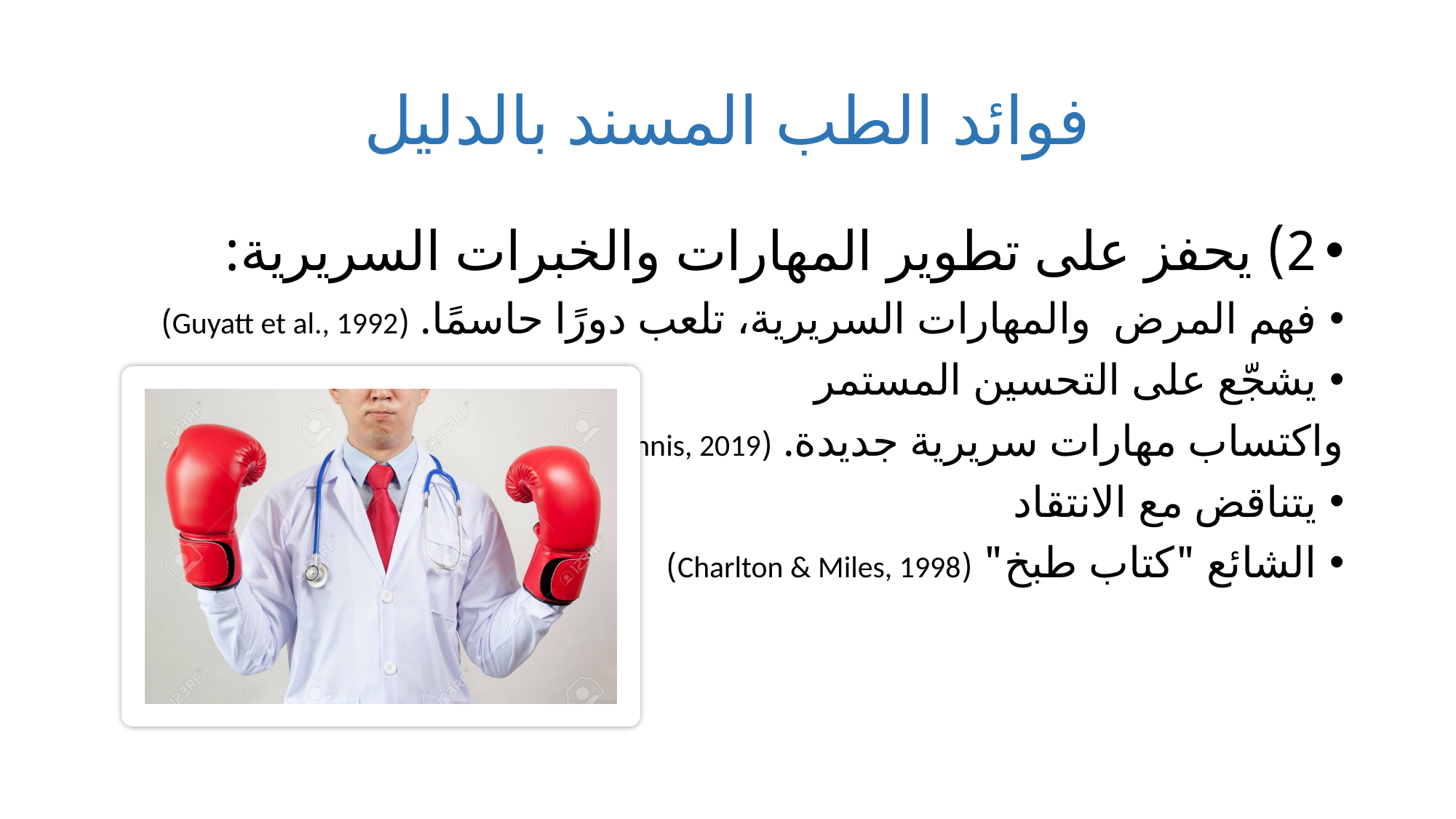

# فوائد الطب المسند بالدليل
2) يحفز على تطوير المهارات والخبرات السريرية:
فهم المرض والمهارات السريرية، تلعب دورًا حاسمًا. (Guyatt et al., 1992)
يشجّع على التحسين المستمر
واكتساب مهارات سريرية جديدة. (Karagiannis, 2019)
يتناقض مع الانتقاد
الشائع "كتاب طبخ" (Charlton & Miles, 1998)

## Slide 7
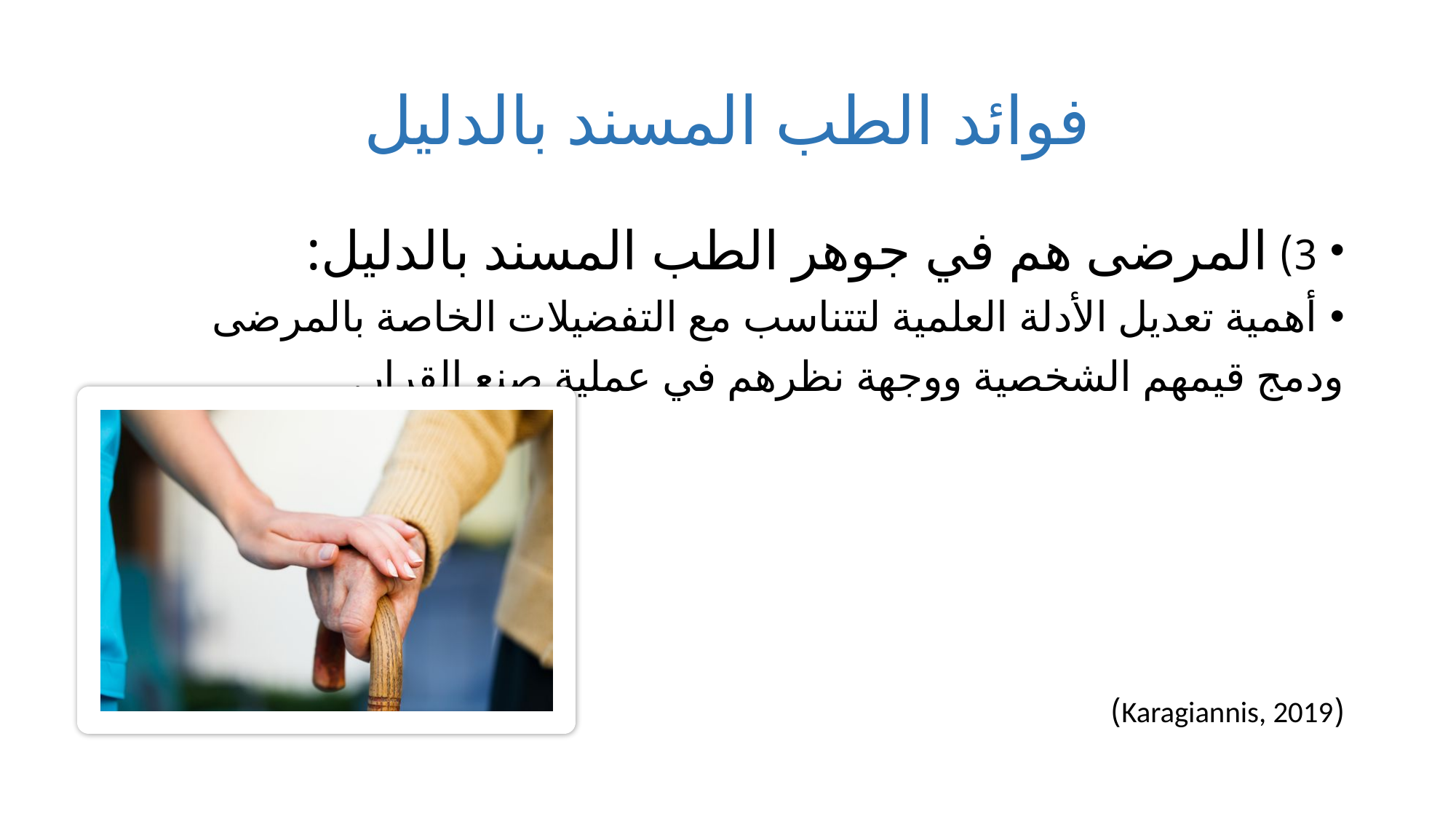

# فوائد الطب المسند بالدليل
3) المرضى هم في جوهر الطب المسند بالدليل:
أهمية تعديل الأدلة العلمية لتتناسب مع التفضيلات الخاصة بالمرضى
ودمج قيمهم الشخصية ووجهة نظرهم في عملية صنع القرار.
(Karagiannis, 2019)

## Slide 8
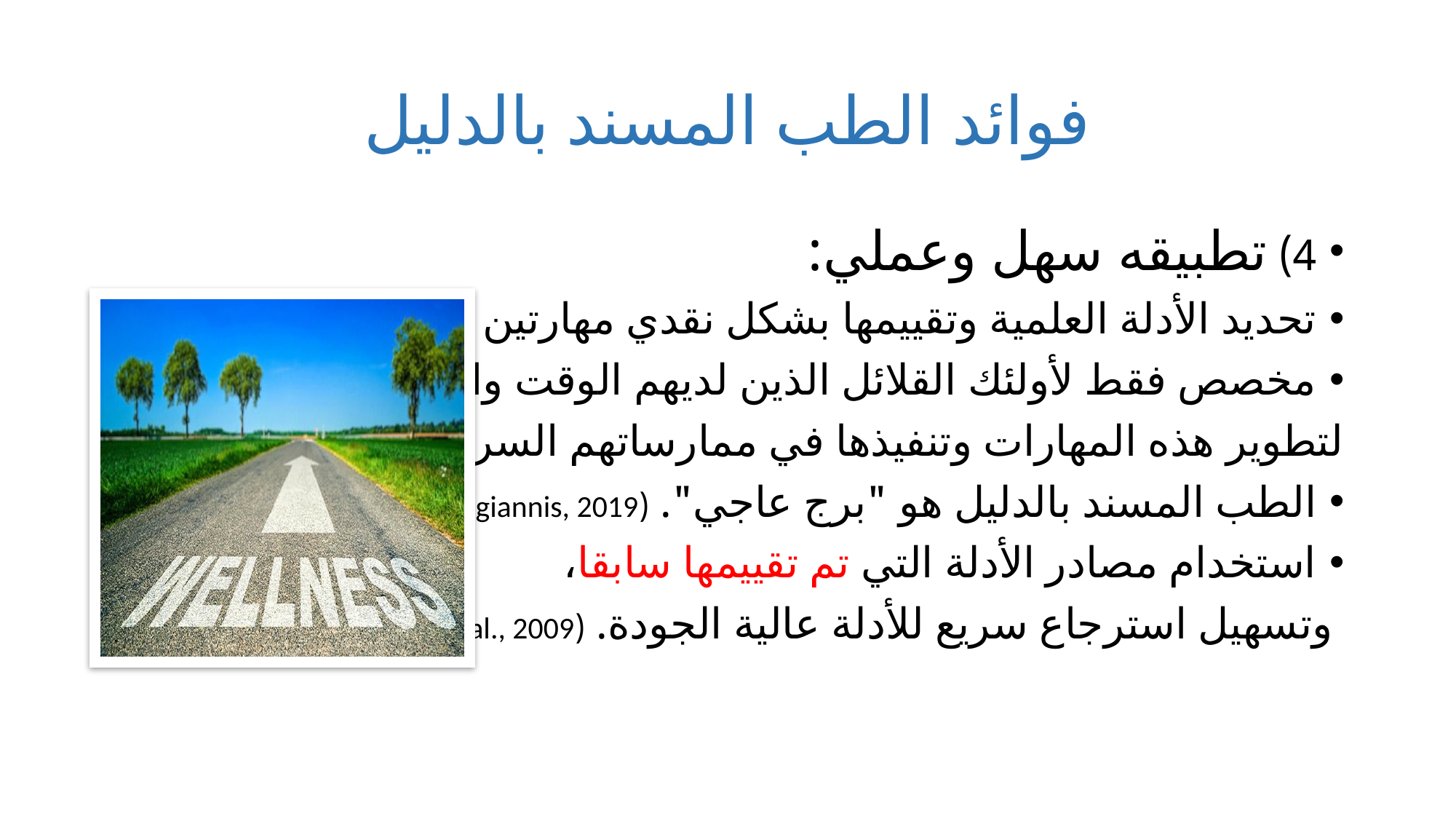

# فوائد الطب المسند بالدليل
4) تطبيقه سهل وعملي:
تحديد الأدلة العلمية وتقييمها بشكل نقدي مهارتين أساسيتين
مخصص فقط لأولئك القلائل الذين لديهم الوقت والموارد
لتطوير هذه المهارات وتنفيذها في ممارساتهم السريرية اليومية .
الطب المسند بالدليل هو "برج عاجي". (Karagiannis, 2019)
استخدام مصادر الأدلة التي تم تقييمها سابقا،
 وتسهيل استرجاع سريع للأدلة عالية الجودة. (DiCenso et al., 2009)

## Slide 9
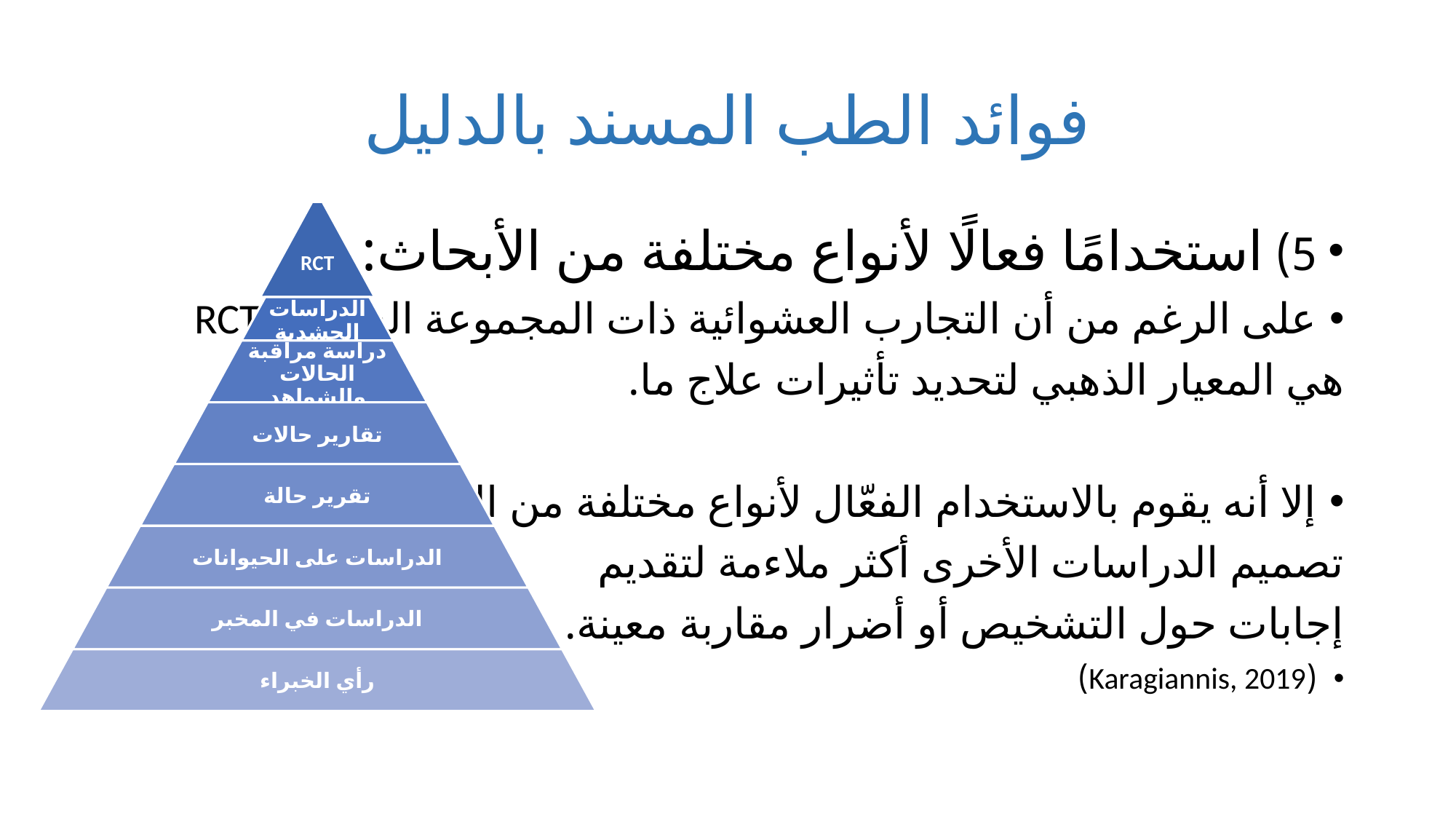

# فوائد الطب المسند بالدليل
5) استخدامًا فعالًا لأنواع مختلفة من الأبحاث:
على الرغم من أن التجارب العشوائية ذات المجموعة الشاهدة RCT
هي المعيار الذهبي لتحديد تأثيرات علاج ما.
إلا أنه يقوم بالاستخدام الفعّال لأنواع مختلفة من الأبحاث
تصميم الدراسات الأخرى أكثر ملاءمة لتقديم
إجابات حول التشخيص أو أضرار مقاربة معينة.
(Karagiannis, 2019)

## Slide 10
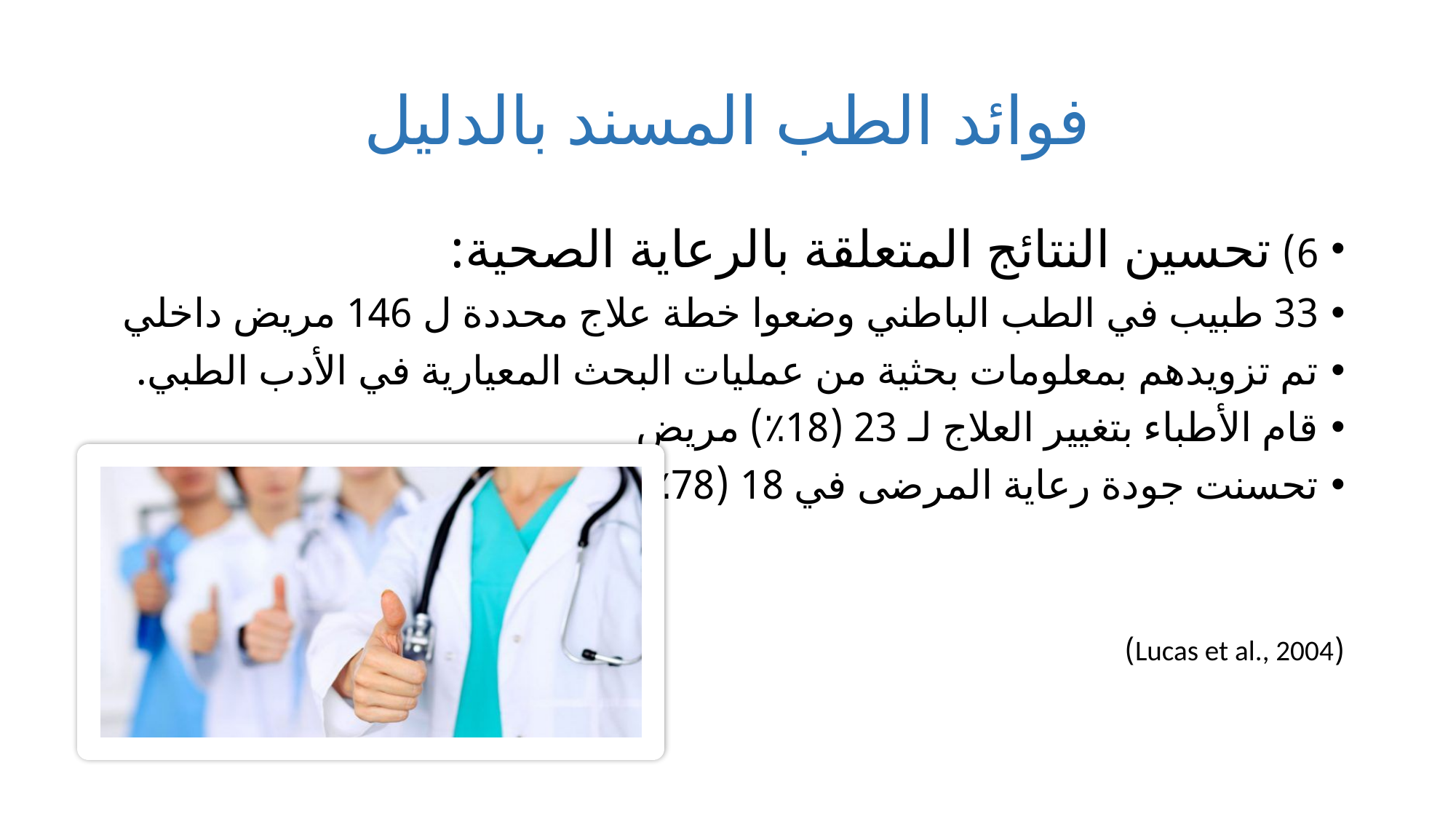

# فوائد الطب المسند بالدليل
6) تحسين النتائج المتعلقة بالرعاية الصحية:
33 طبيب في الطب الباطني وضعوا خطة علاج محددة ل 146 مريض داخلي
تم تزويدهم بمعلومات بحثية من عمليات البحث المعيارية في الأدب الطبي.
قام الأطباء بتغيير العلاج لـ 23 (18٪) مريض
تحسنت جودة رعاية المرضى في 18 (78٪).
(Lucas et al., 2004)

## Slide 11
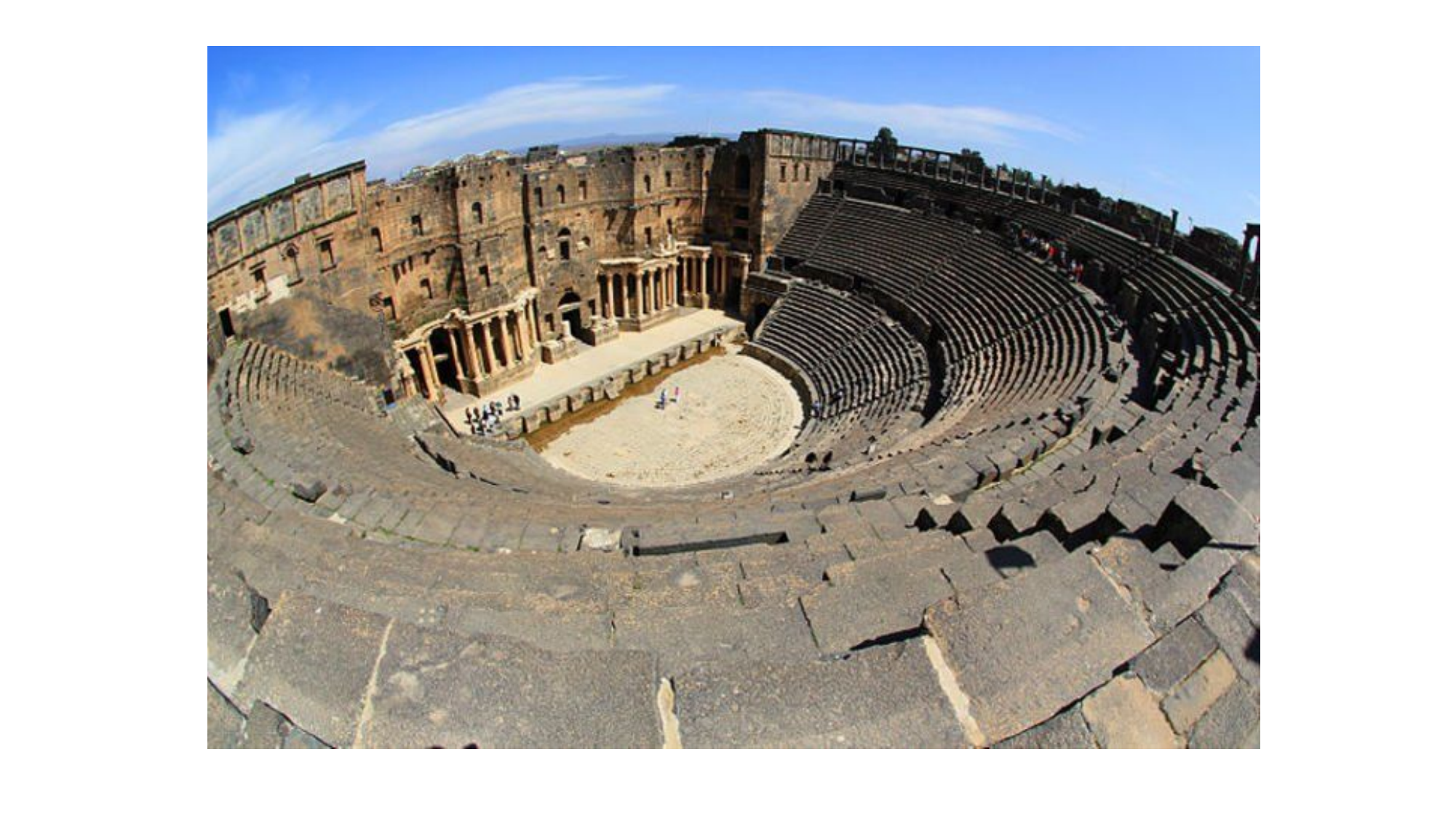

## Slide 12
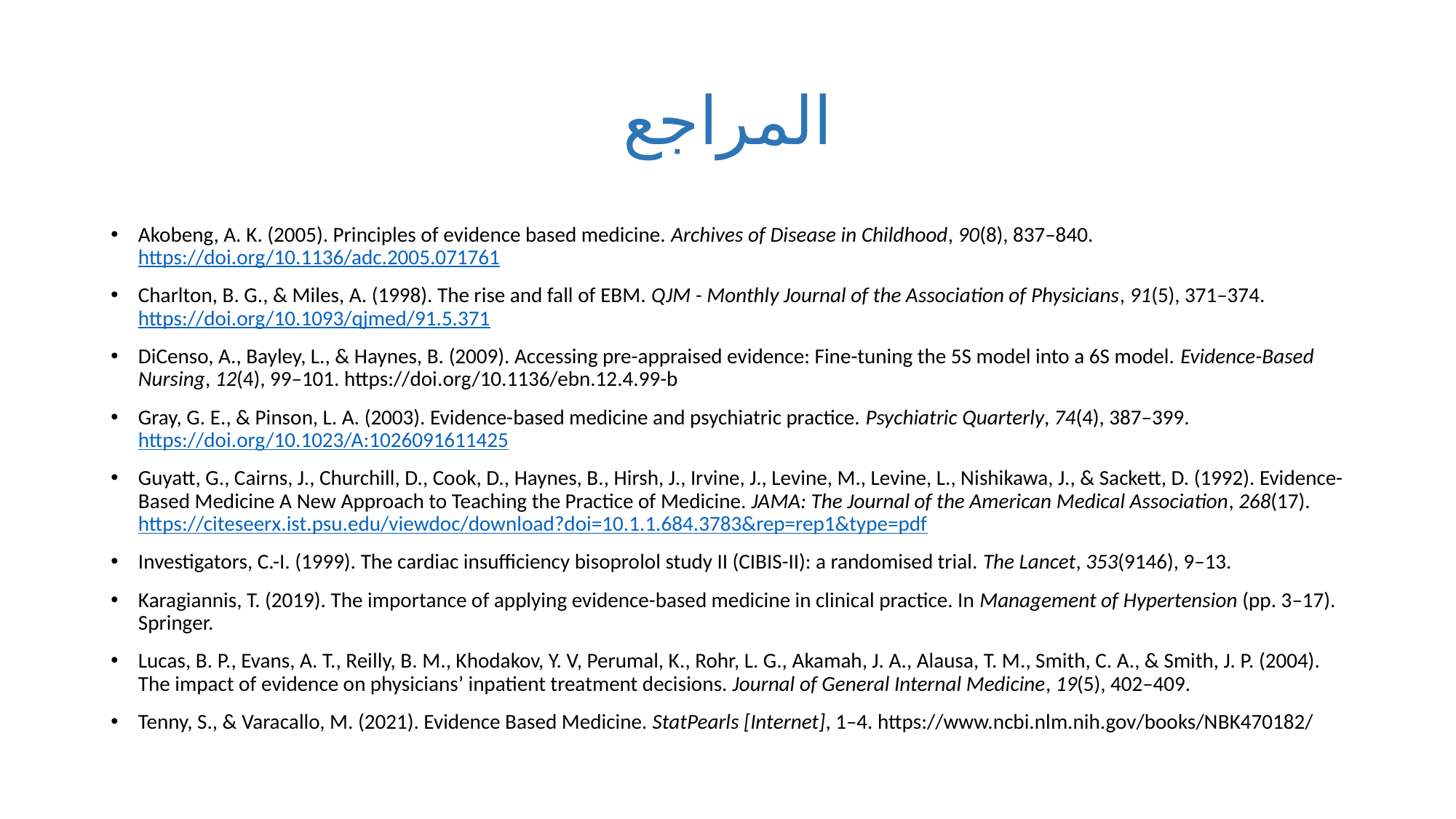

# المراجع
Akobeng, A. K. (2005). Principles of evidence based medicine. Archives of Disease in Childhood, 90(8), 837–840. https://doi.org/10.1136/adc.2005.071761
Charlton, B. G., & Miles, A. (1998). The rise and fall of EBM. QJM - Monthly Journal of the Association of Physicians, 91(5), 371–374. https://doi.org/10.1093/qjmed/91.5.371
DiCenso, A., Bayley, L., & Haynes, B. (2009). Accessing pre-appraised evidence: Fine-tuning the 5S model into a 6S model. Evidence-Based Nursing, 12(4), 99–101. https://doi.org/10.1136/ebn.12.4.99-b
Gray, G. E., & Pinson, L. A. (2003). Evidence-based medicine and psychiatric practice. Psychiatric Quarterly, 74(4), 387–399. https://doi.org/10.1023/A:1026091611425
Guyatt, G., Cairns, J., Churchill, D., Cook, D., Haynes, B., Hirsh, J., Irvine, J., Levine, M., Levine, L., Nishikawa, J., & Sackett, D. (1992). Evidence-Based Medicine A New Approach to Teaching the Practice of Medicine. JAMA: The Journal of the American Medical Association, 268(17). https://citeseerx.ist.psu.edu/viewdoc/download?doi=10.1.1.684.3783&rep=rep1&type=pdf
Investigators, C.-I. (1999). The cardiac insufficiency bisoprolol study II (CIBIS-II): a randomised trial. The Lancet, 353(9146), 9–13.
Karagiannis, T. (2019). The importance of applying evidence-based medicine in clinical practice. In Management of Hypertension (pp. 3–17). Springer.
Lucas, B. P., Evans, A. T., Reilly, B. M., Khodakov, Y. V, Perumal, K., Rohr, L. G., Akamah, J. A., Alausa, T. M., Smith, C. A., & Smith, J. P. (2004). The impact of evidence on physicians’ inpatient treatment decisions. Journal of General Internal Medicine, 19(5), 402–409.
Tenny, S., & Varacallo, M. (2021). Evidence Based Medicine. StatPearls [Internet], 1–4. https://www.ncbi.nlm.nih.gov/books/NBK470182/
