# Supplementary material for: The First Asynchronous Online Evidence-Based Medicine Course for Syrian Health Workforce: Effectiveness and Feasibility Pilot Study
Source: JMIR Form Res. 2022 Oct 25;6(10):e36782. doi: 10.2196/36782 (PMC9644249; doi:10.2196/36782)
Supplement: Multimedia Appendix 3 [file formative_v6i10e36782_app3.pptx]

## Slide 1
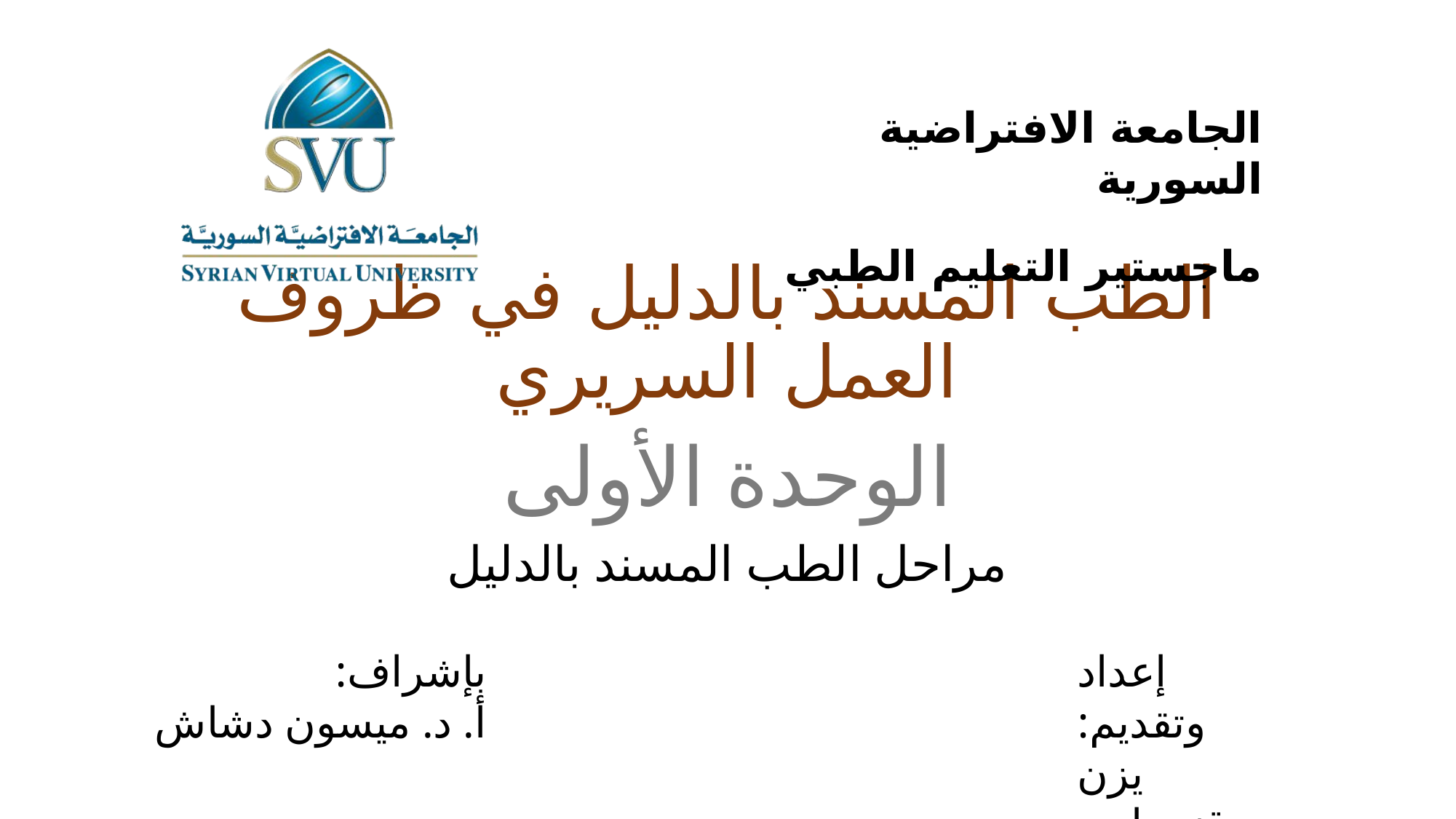

الجامعة الافتراضية السورية
ماجستير التعليم الطبي
# الطب المسند بالدليل في ظروف العمل السريري
الوحدة الأولى
مراحل الطب المسند بالدليل
إعداد وتقديم:
يزن قنجراوي
بإشراف:
أ. د. ميسون دشاش

## Slide 2
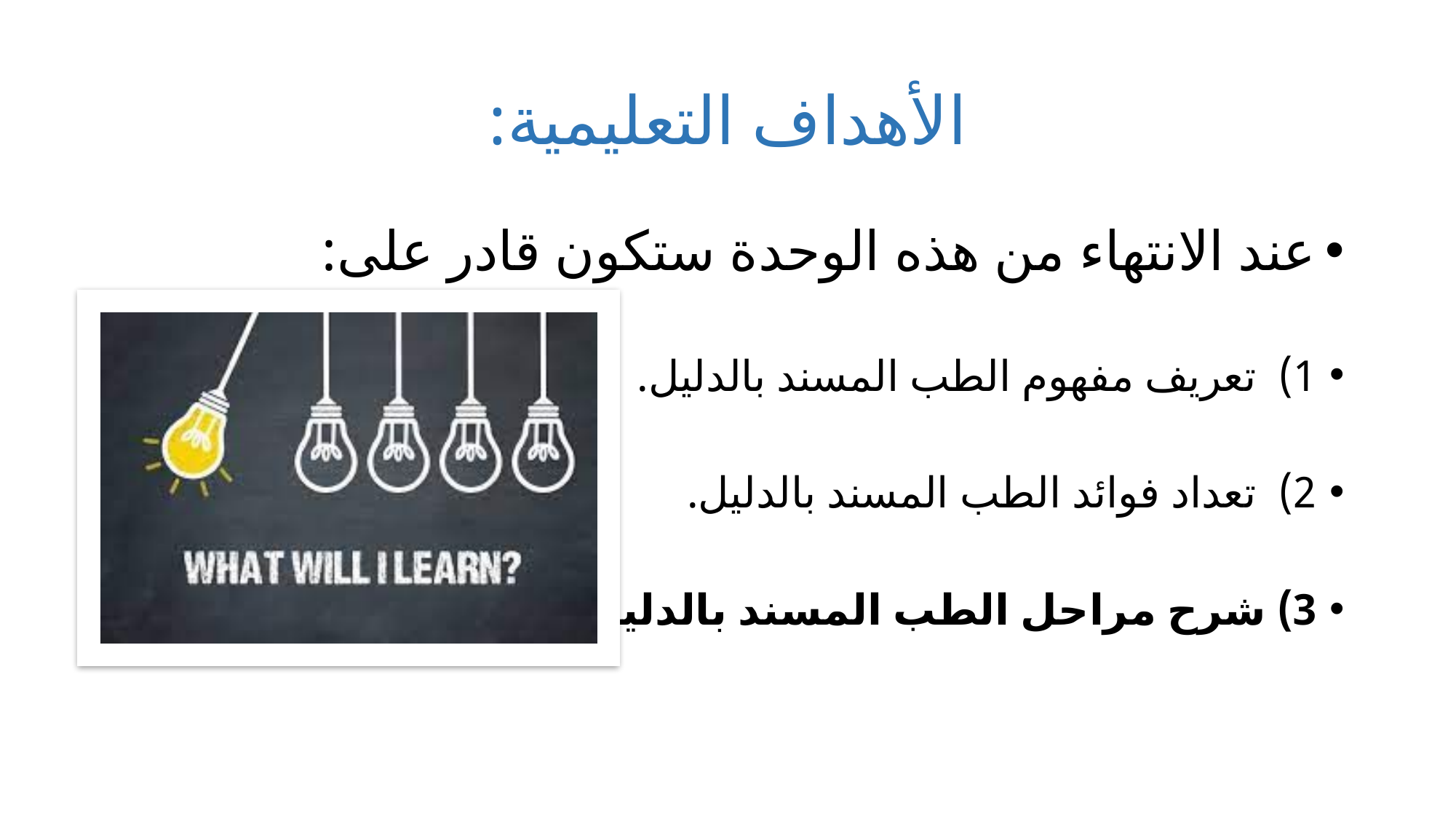

# الأهداف التعليمية:
عند الانتهاء من هذه الوحدة ستكون قادر على:
1) تعريف مفهوم الطب المسند بالدليل.
2) تعداد فوائد الطب المسند بالدليل.
3) شرح مراحل الطب المسند بالدليل الخمسة.

## Slide 3
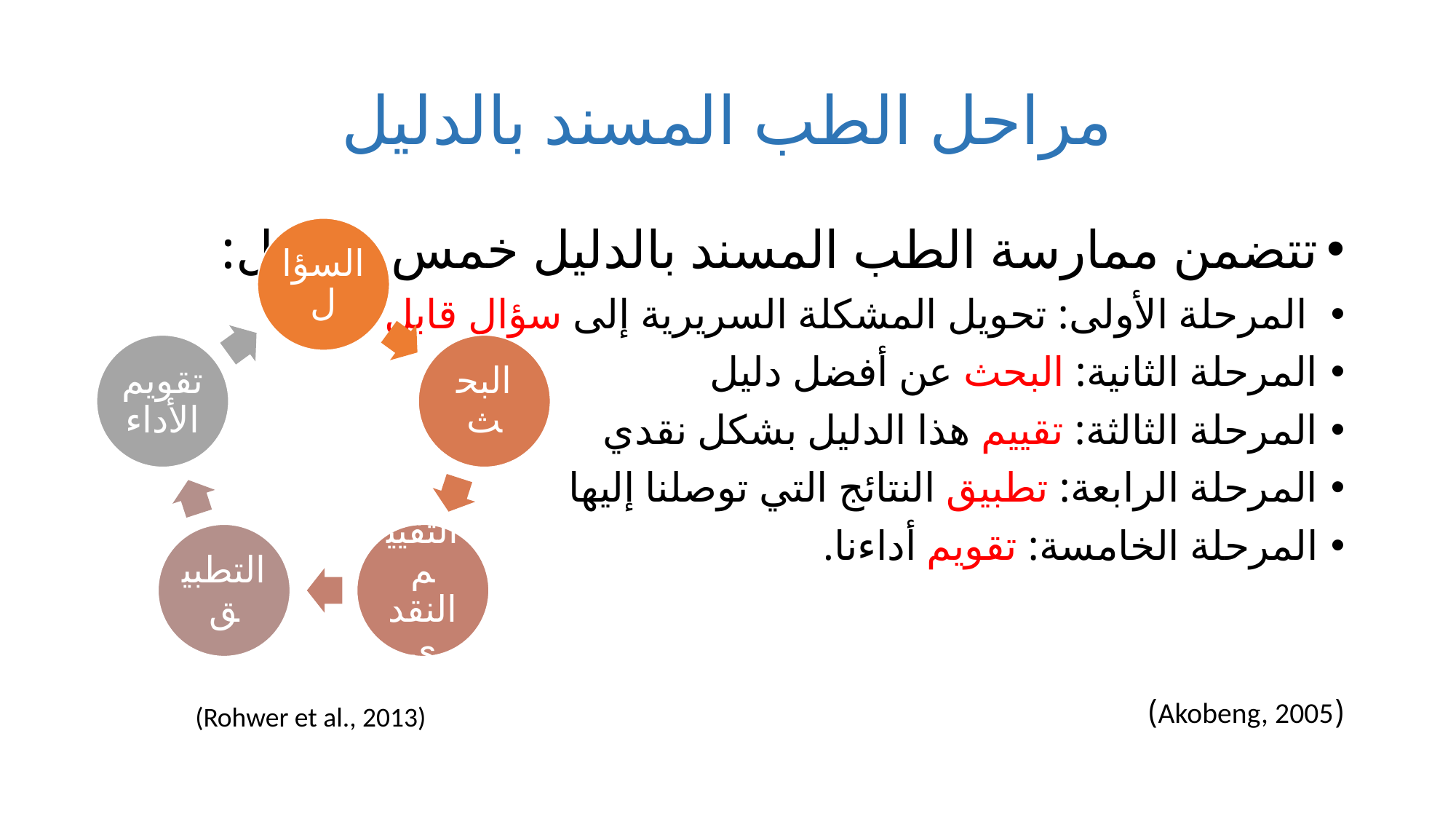

# مراحل الطب المسند بالدليل
تتضمن ممارسة الطب المسند بالدليل خمس مراحل:
 المرحلة الأولى: تحويل المشكلة السريرية إلى سؤال قابل للحل
المرحلة الثانية: البحث عن أفضل دليل
المرحلة الثالثة: تقييم هذا الدليل بشكل نقدي
المرحلة الرابعة: تطبيق النتائج التي توصلنا إليها
المرحلة الخامسة: تقويم أداءنا.
(Akobeng, 2005)
(Rohwer et al., 2013)

## Slide 4
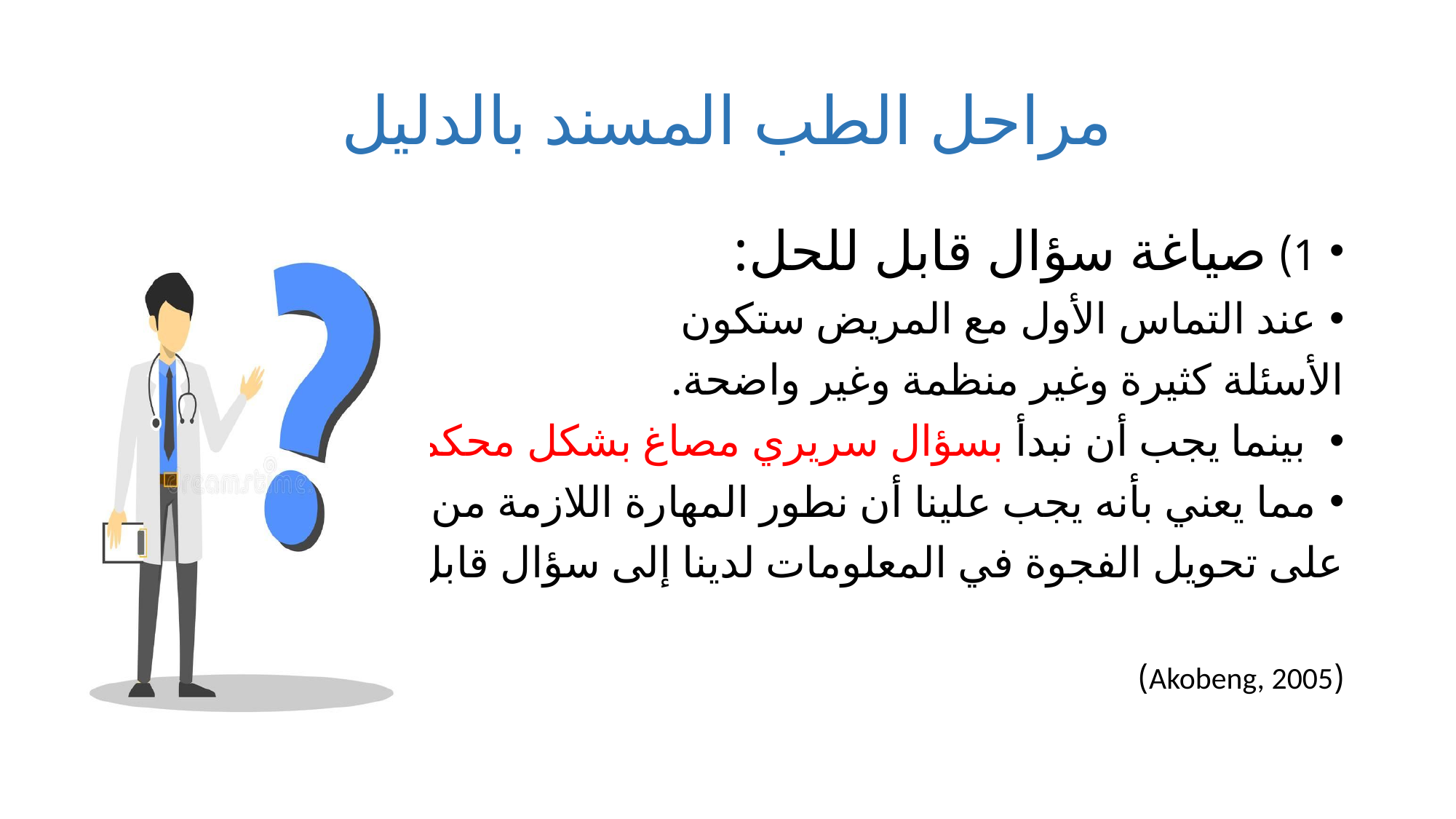

# مراحل الطب المسند بالدليل
1) صياغة سؤال قابل للحل:
عند التماس الأول مع المريض ستكون
الأسئلة كثيرة وغير منظمة وغير واضحة.
 بينما يجب أن نبدأ بسؤال سريري مصاغ بشكل محكم.
مما يعني بأنه يجب علينا أن نطور المهارة اللازمة من أجل القدرة
على تحويل الفجوة في المعلومات لدينا إلى سؤال قابل للحل.
(Akobeng, 2005)

## Slide 5
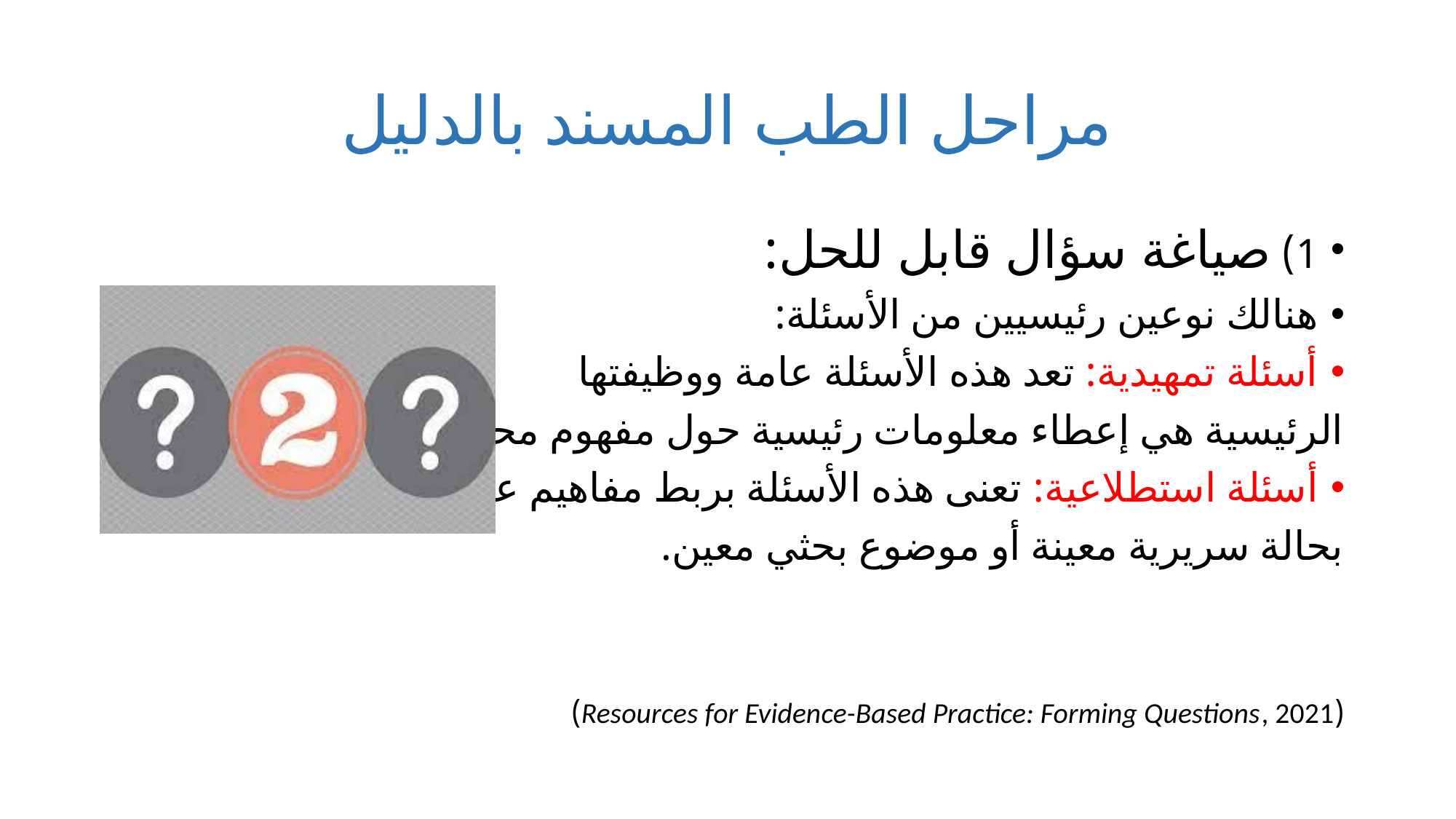

# مراحل الطب المسند بالدليل
1) صياغة سؤال قابل للحل:
هنالك نوعين رئيسيين من الأسئلة:
أسئلة تمهيدية: تعد هذه الأسئلة عامة ووظيفتها
الرئيسية هي إعطاء معلومات رئيسية حول مفهوم محدد.
أسئلة استطلاعية: تعنى هذه الأسئلة بربط مفاهيم عديدة
بحالة سريرية معينة أو موضوع بحثي معين.
(Resources for Evidence-Based Practice: Forming Questions, 2021)

## Slide 6
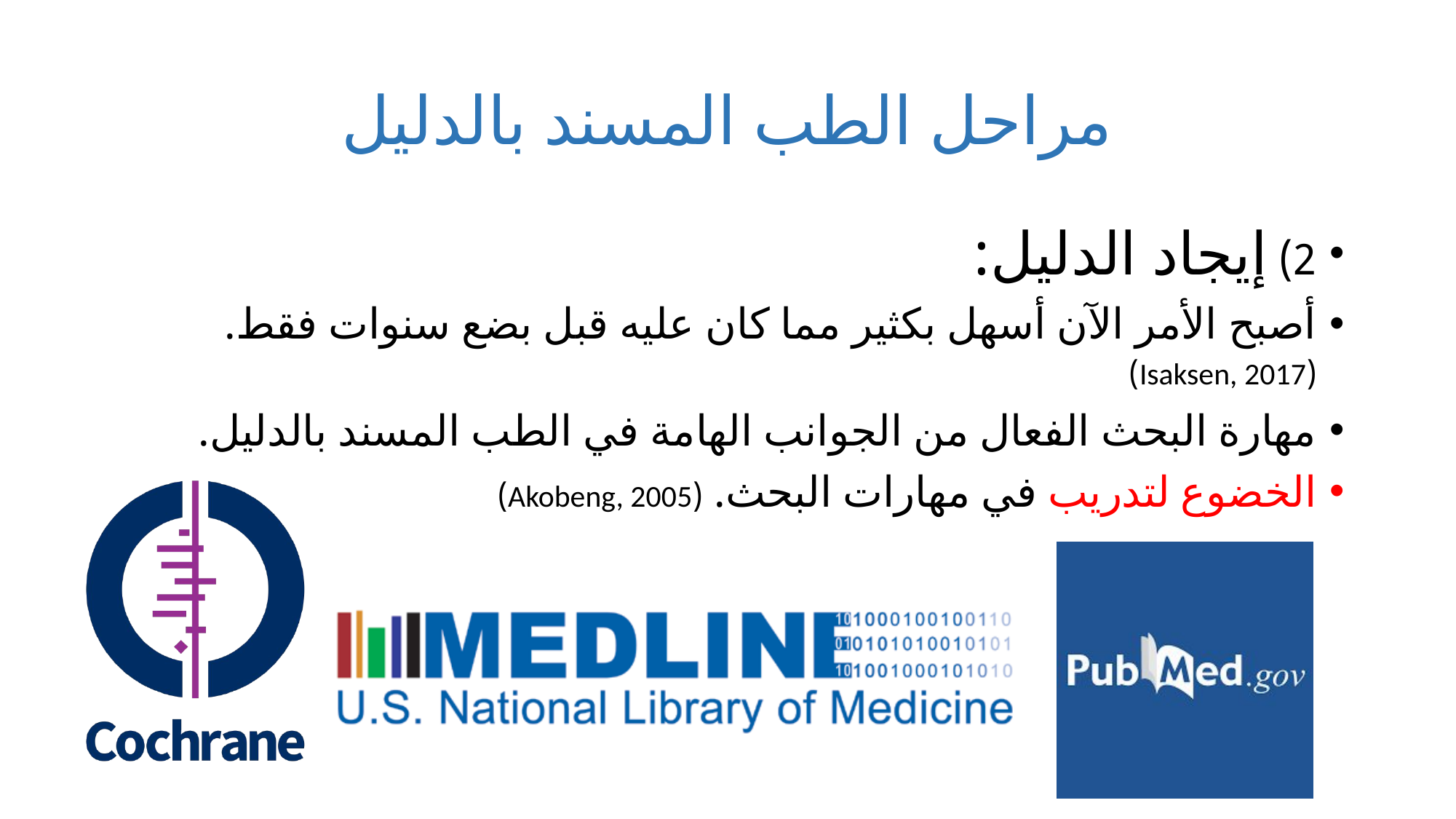

# مراحل الطب المسند بالدليل
2) إيجاد الدليل:
أصبح الأمر الآن أسهل بكثير مما كان عليه قبل بضع سنوات فقط. (Isaksen, 2017)
مهارة البحث الفعال من الجوانب الهامة في الطب المسند بالدليل.
الخضوع لتدريب في مهارات البحث. (Akobeng, 2005)

## Slide 7
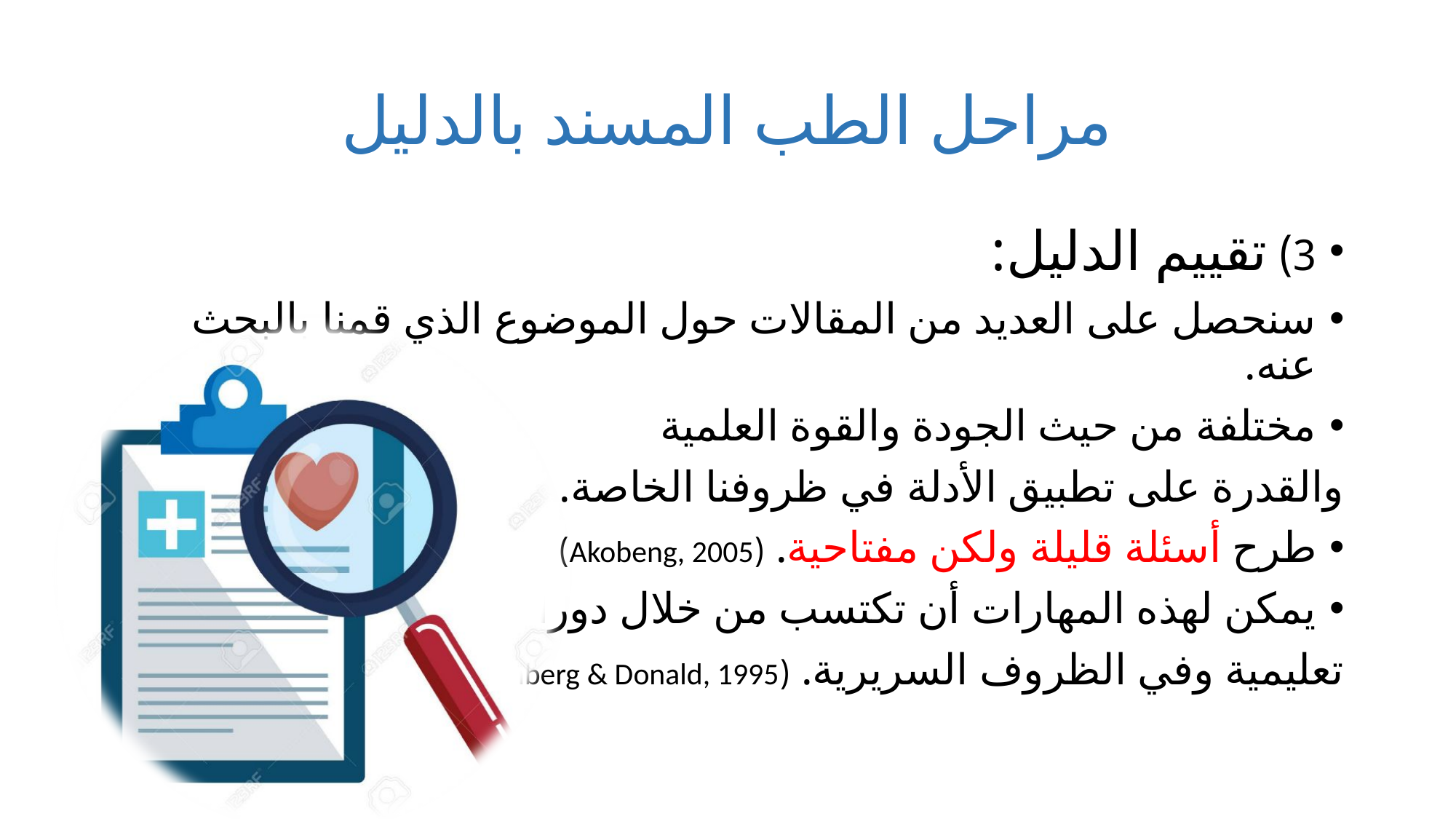

# مراحل الطب المسند بالدليل
3) تقييم الدليل:
سنحصل على العديد من المقالات حول الموضوع الذي قمنا بالبحث عنه.
مختلفة من حيث الجودة والقوة العلمية
والقدرة على تطبيق الأدلة في ظروفنا الخاصة.
طرح أسئلة قليلة ولكن مفتاحية. (Akobeng, 2005)
يمكن لهذه المهارات أن تكتسب من خلال دورات
تعليمية وفي الظروف السريرية. (Rosenberg & Donald, 1995)

## Slide 8
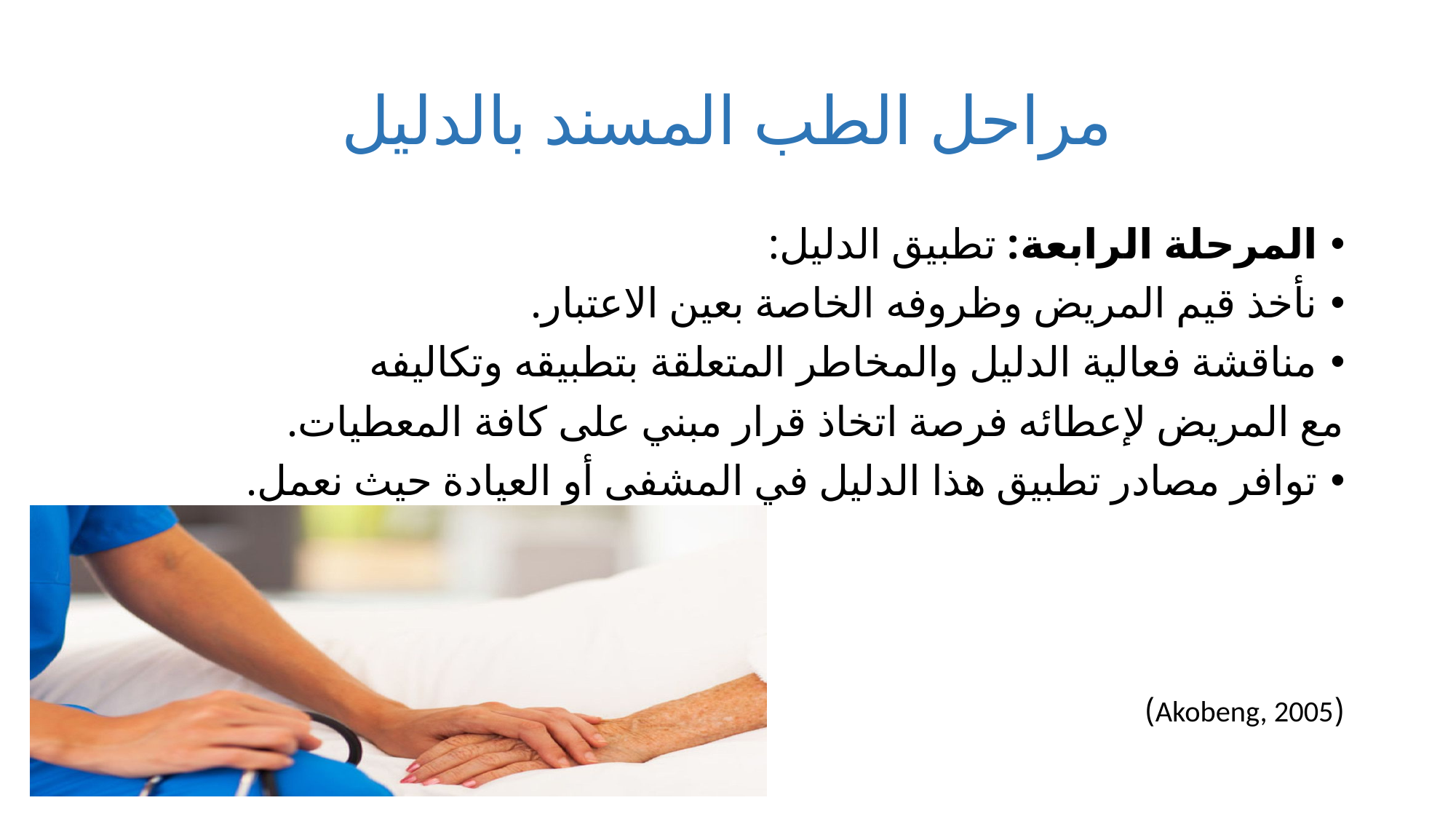

# مراحل الطب المسند بالدليل
المرحلة الرابعة: تطبيق الدليل:
نأخذ قيم المريض وظروفه الخاصة بعين الاعتبار.
مناقشة فعالية الدليل والمخاطر المتعلقة بتطبيقه وتكاليفه
مع المريض لإعطائه فرصة اتخاذ قرار مبني على كافة المعطيات.
توافر مصادر تطبيق هذا الدليل في المشفى أو العيادة حيث نعمل.
(Akobeng, 2005)

## Slide 9
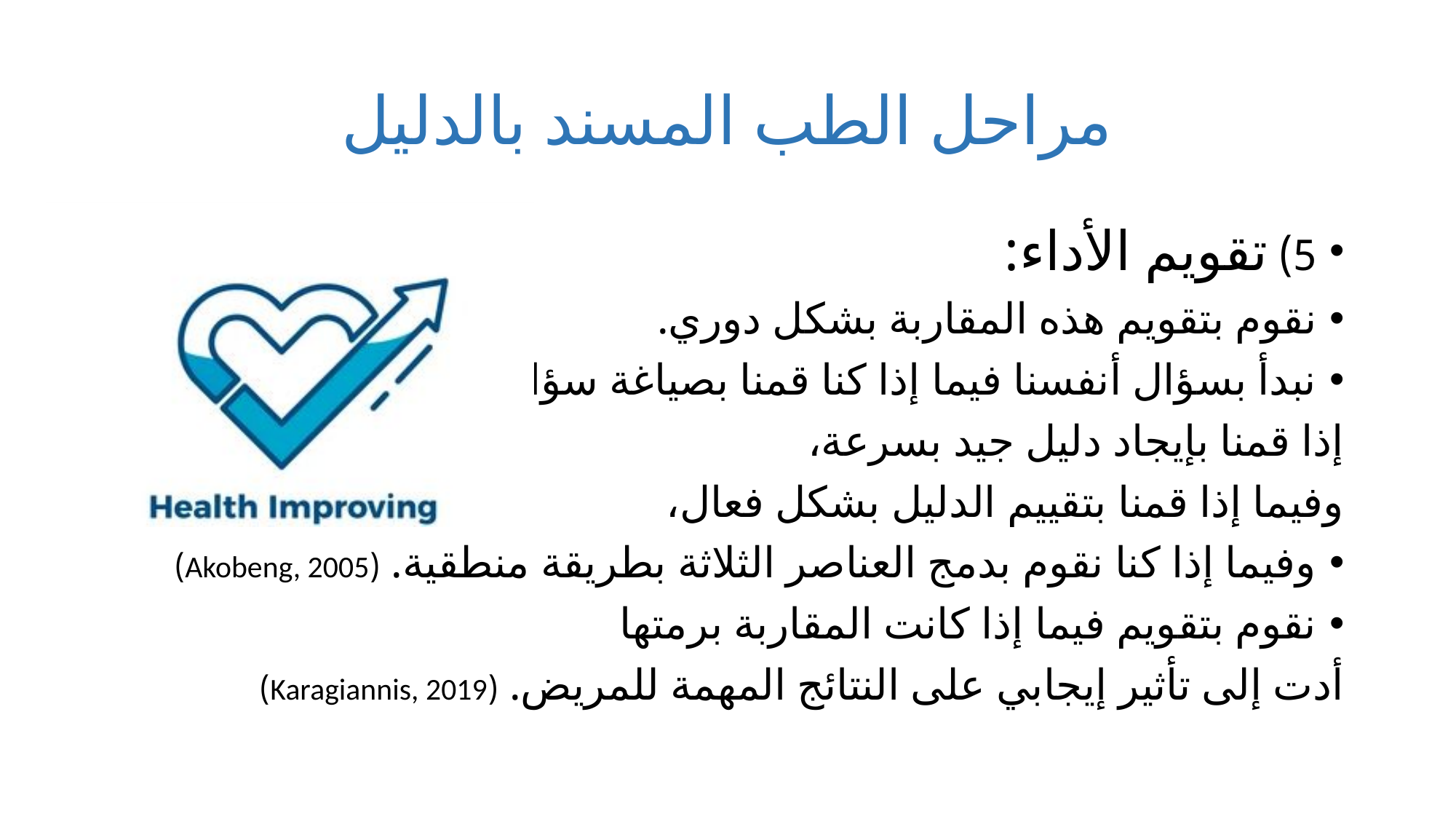

# مراحل الطب المسند بالدليل
5) تقويم الأداء:
نقوم بتقويم هذه المقاربة بشكل دوري.
نبدأ بسؤال أنفسنا فيما إذا كنا قمنا بصياغة سؤال قابل للحل،
إذا قمنا بإيجاد دليل جيد بسرعة،
وفيما إذا قمنا بتقييم الدليل بشكل فعال،
وفيما إذا كنا نقوم بدمج العناصر الثلاثة بطريقة منطقية. (Akobeng, 2005)
نقوم بتقويم فيما إذا كانت المقاربة برمتها
أدت إلى تأثير إيجابي على النتائج المهمة للمريض. (Karagiannis, 2019)

## Slide 10
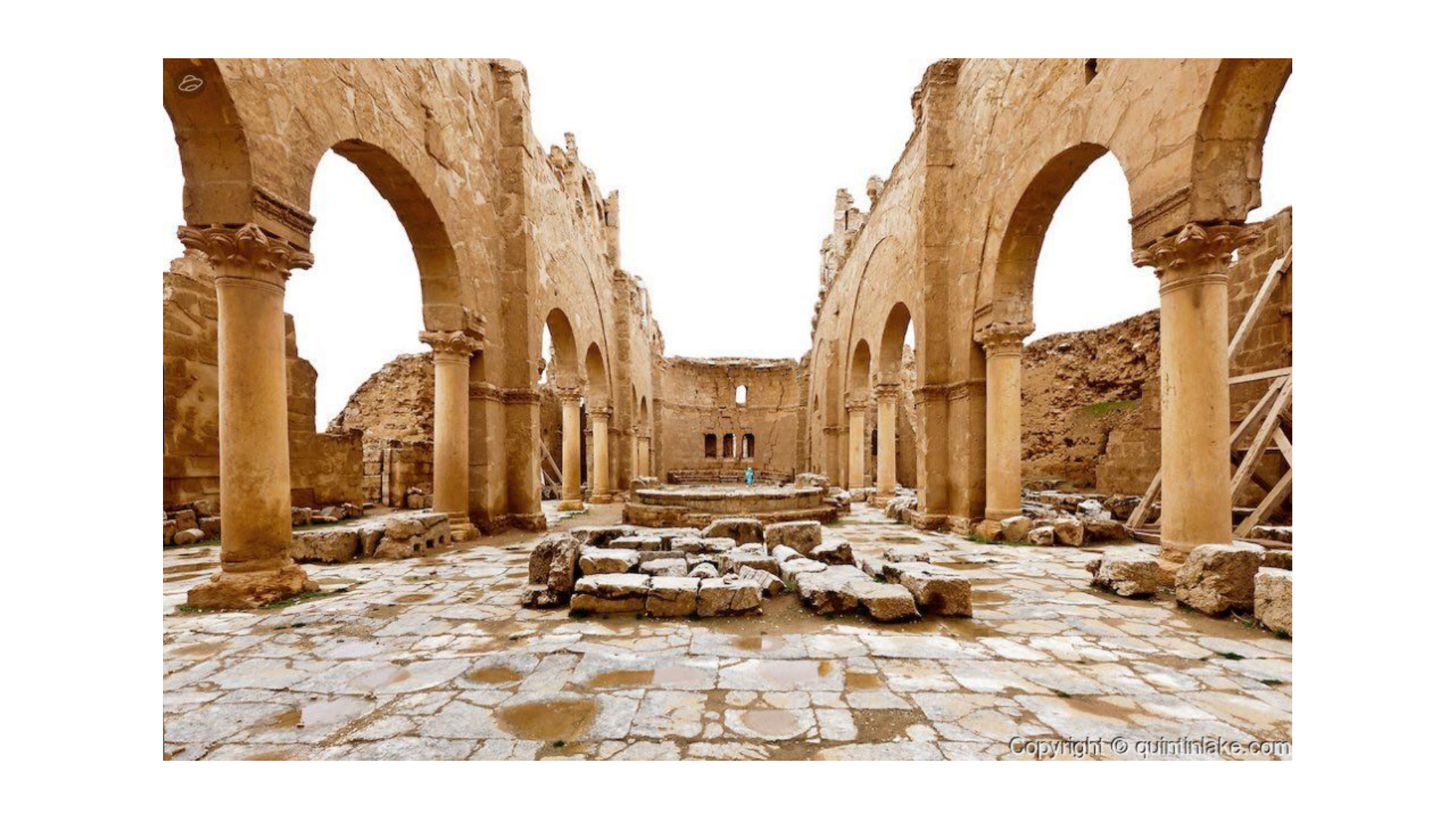

## Slide 11
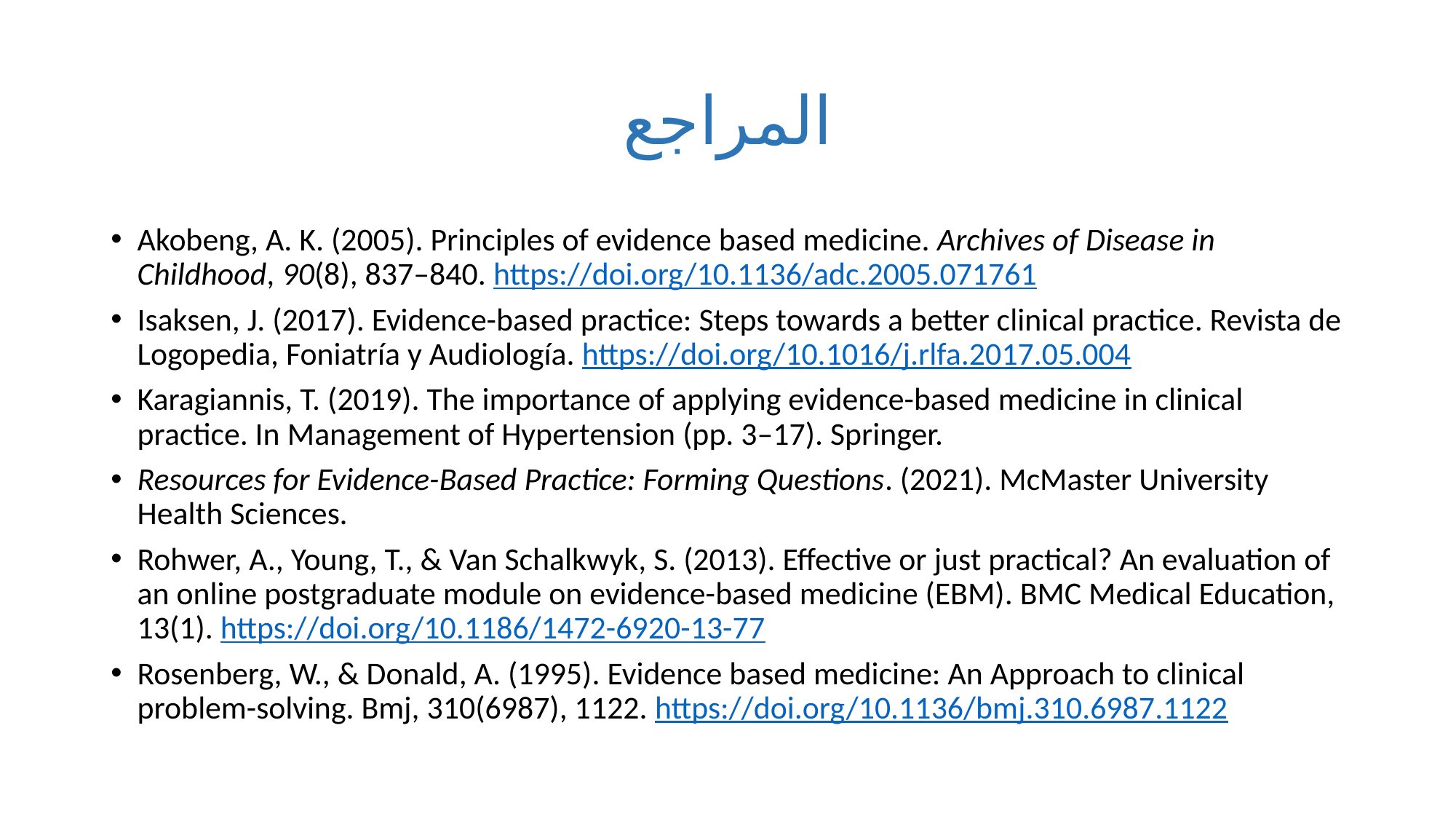

# المراجع
Akobeng, A. K. (2005). Principles of evidence based medicine. Archives of Disease in Childhood, 90(8), 837–840. https://doi.org/10.1136/adc.2005.071761
Isaksen, J. (2017). Evidence-based practice: Steps towards a better clinical practice. Revista de Logopedia, Foniatría y Audiología. https://doi.org/10.1016/j.rlfa.2017.05.004
Karagiannis, T. (2019). The importance of applying evidence-based medicine in clinical practice. In Management of Hypertension (pp. 3–17). Springer.
Resources for Evidence-Based Practice: Forming Questions. (2021). McMaster University Health Sciences.
Rohwer, A., Young, T., & Van Schalkwyk, S. (2013). Effective or just practical? An evaluation of an online postgraduate module on evidence-based medicine (EBM). BMC Medical Education, 13(1). https://doi.org/10.1186/1472-6920-13-77
Rosenberg, W., & Donald, A. (1995). Evidence based medicine: An Approach to clinical problem-solving. Bmj, 310(6987), 1122. https://doi.org/10.1136/bmj.310.6987.1122
