# Supplementary material for: The First Asynchronous Online Evidence-Based Medicine Course for Syrian Health Workforce: Effectiveness and Feasibility Pilot Study
Source: JMIR Form Res. 2022 Oct 25;6(10):e36782. doi: 10.2196/36782 (PMC9644249; doi:10.2196/36782)
Supplement: Multimedia Appendix 4 [file formative_v6i10e36782_app4.pptx]

## Slide 1
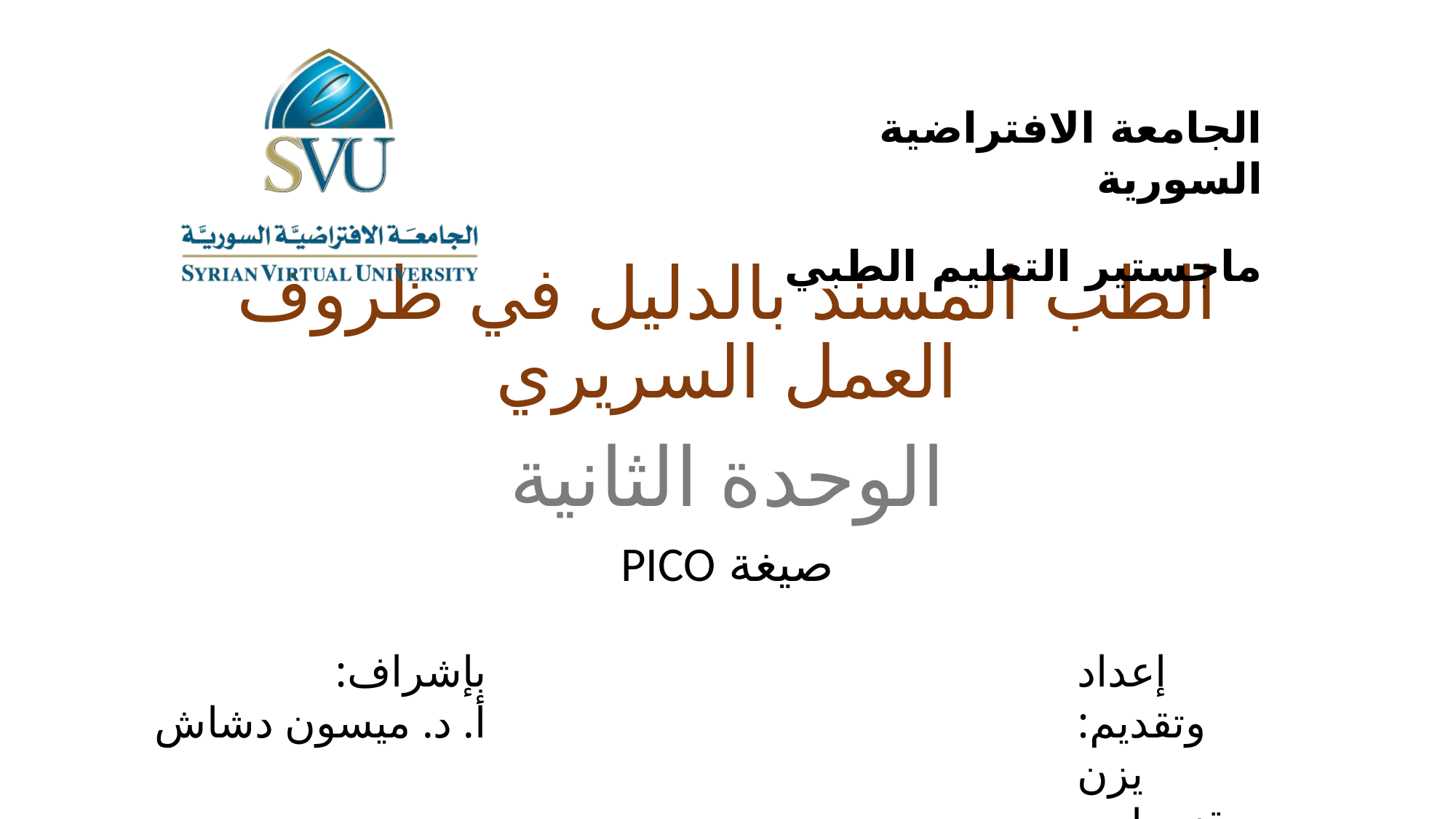

الجامعة الافتراضية السورية
ماجستير التعليم الطبي
# الطب المسند بالدليل في ظروف العمل السريري
الوحدة الثانية
صيغة PICO
إعداد وتقديم:
يزن قنجراوي
بإشراف:
أ. د. ميسون دشاش

## Slide 2
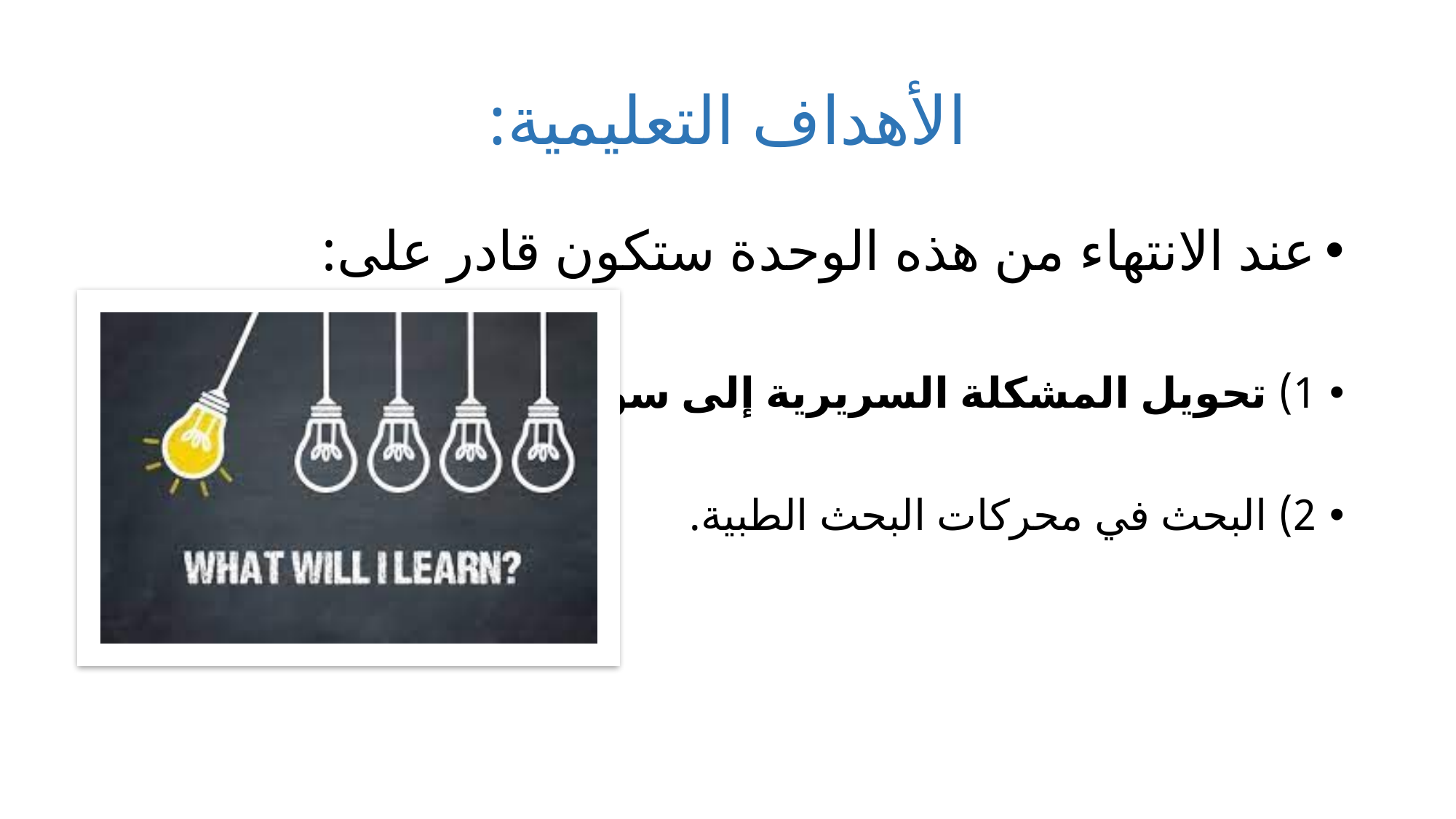

# الأهداف التعليمية:
عند الانتهاء من هذه الوحدة ستكون قادر على:
1) تحويل المشكلة السريرية إلى سؤال.
2) البحث في محركات البحث الطبية.

## Slide 3
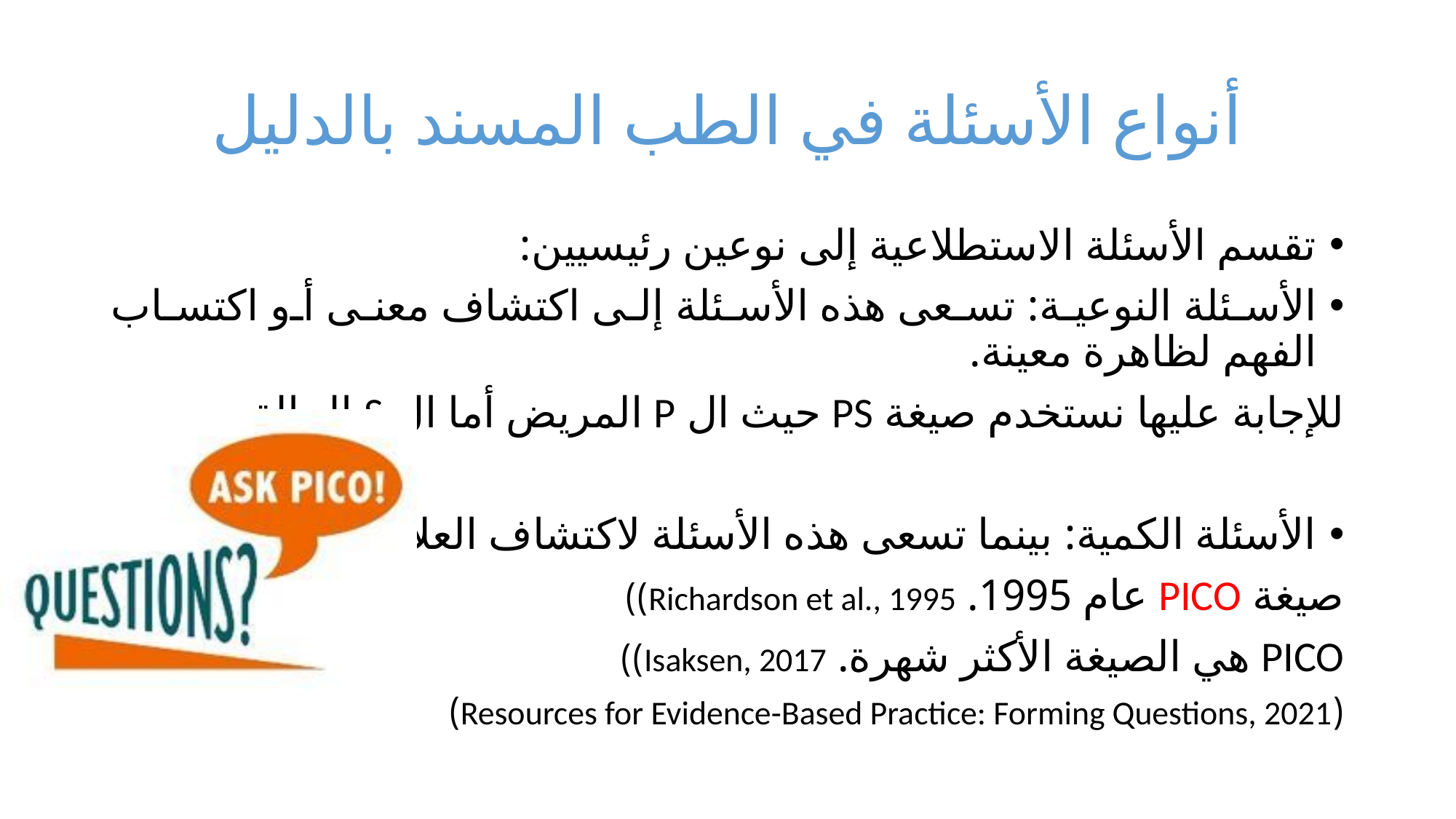

# أنواع الأسئلة في الطب المسند بالدليل
تقسم الأسئلة الاستطلاعية إلى نوعين رئيسيين:
الأسئلة النوعية: تسعى هذه الأسئلة إلى اكتشاف معنى أو اكتساب الفهم لظاهرة معينة.
للإجابة عليها نستخدم صيغة PS حيث ال P المريض أما ال S الحالة.
الأسئلة الكمية: بينما تسعى هذه الأسئلة لاكتشاف العلاقات السببية.
صيغة PICO عام 1995. Richardson et al., 1995))
PICO هي الصيغة الأكثر شهرة. Isaksen, 2017))
(Resources for Evidence-Based Practice: Forming Questions, 2021)

## Slide 4
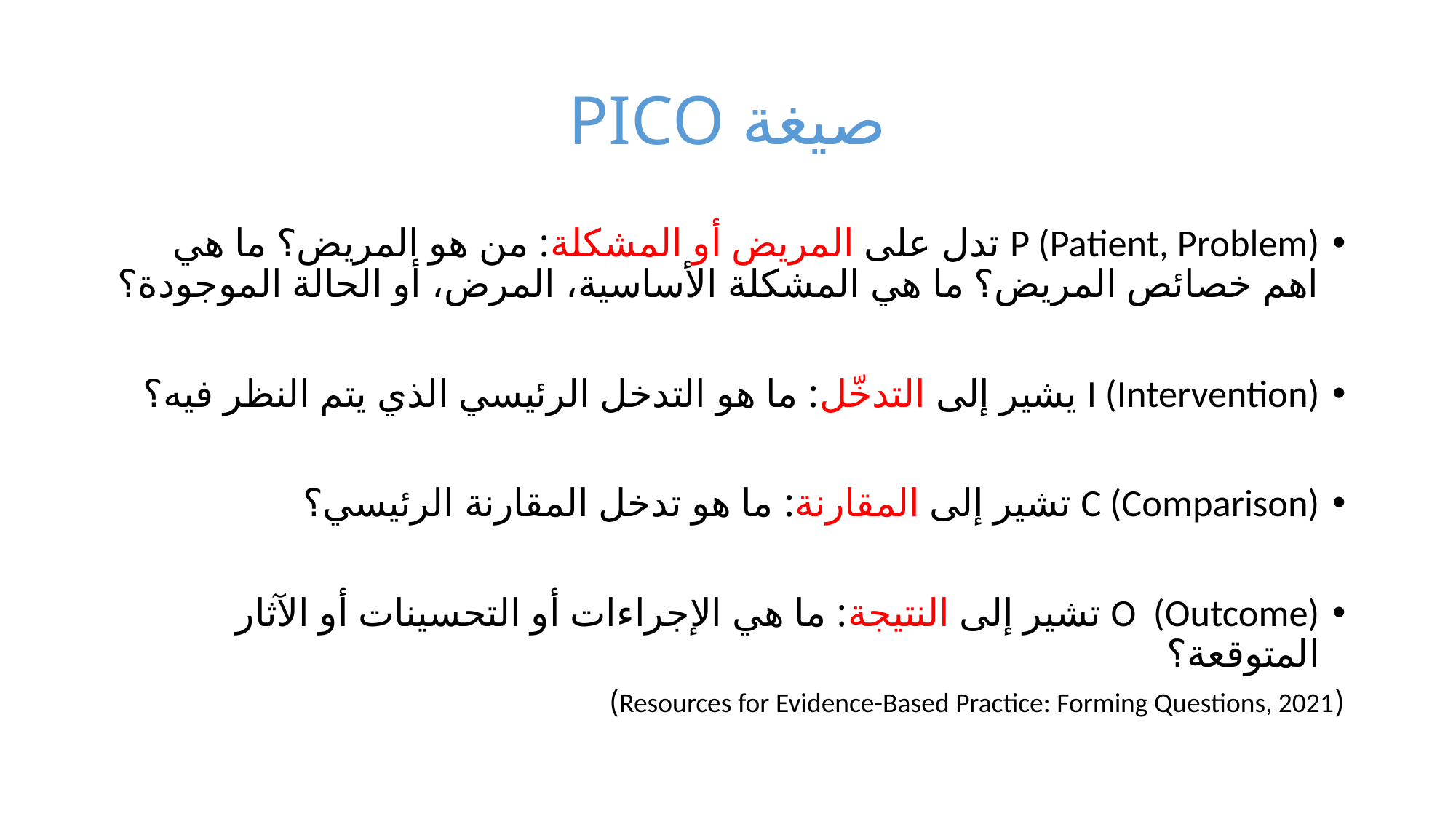

# صيغة PICO
P (Patient, Problem) تدل على المريض أو المشكلة: من هو المريض؟ ما هي اهم خصائص المريض؟ ما هي المشكلة الأساسية، المرض، أو الحالة الموجودة؟
I (Intervention) يشير إلى التدخّل: ما هو التدخل الرئيسي الذي يتم النظر فيه؟
C (Comparison) تشير إلى المقارنة: ما هو تدخل المقارنة الرئيسي؟
O (Outcome) تشير إلى النتيجة: ما هي الإجراءات أو التحسينات أو الآثار المتوقعة؟
(Resources for Evidence-Based Practice: Forming Questions, 2021)

## Slide 5
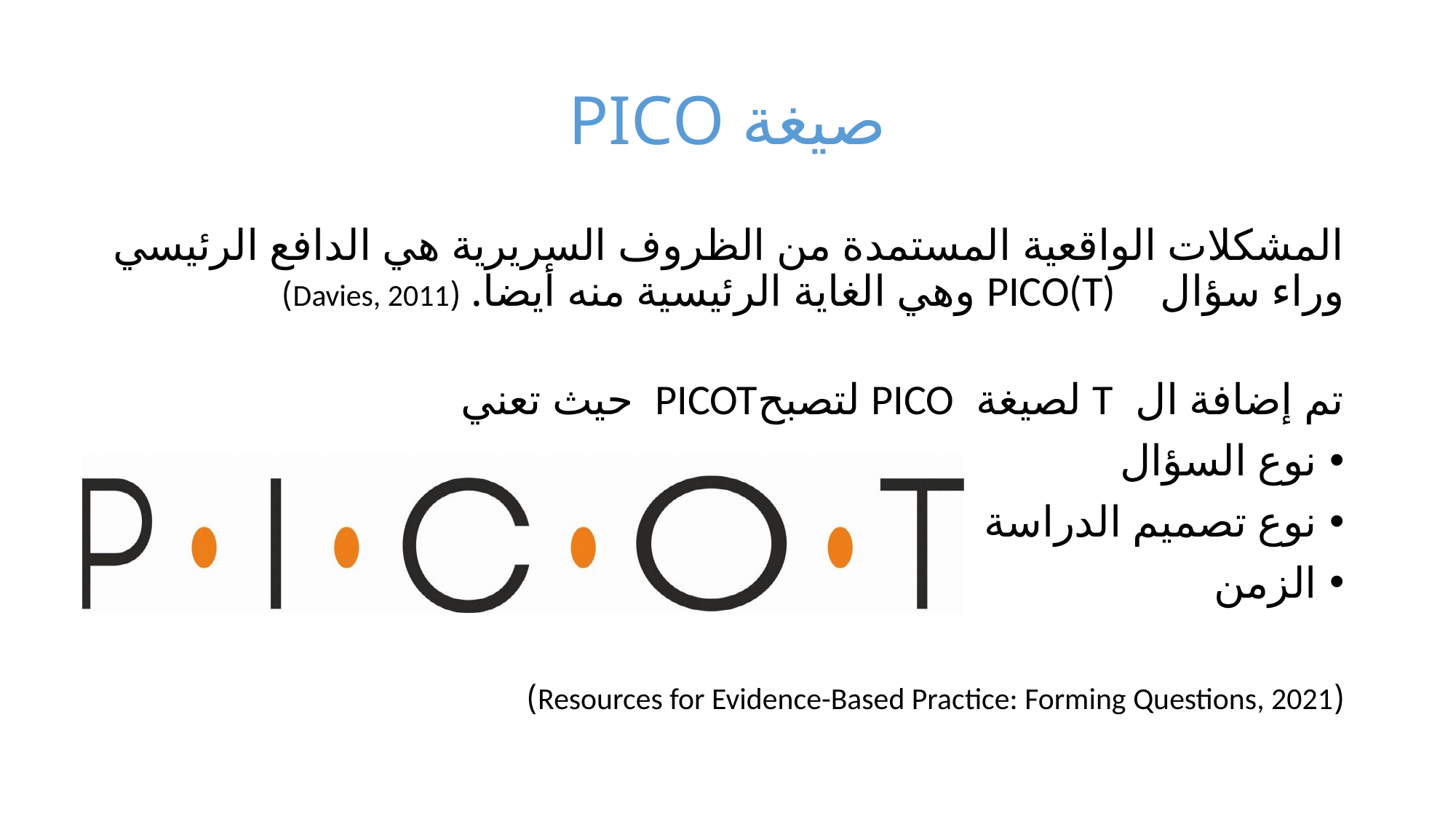

# صيغة PICO
المشكلات الواقعية المستمدة من الظروف السريرية هي الدافع الرئيسي وراء سؤال PICO(T) وهي الغاية الرئيسية منه أيضا. (Davies, 2011)
تم إضافة ال T لصيغة PICO لتصبحPICOT حيث تعني
نوع السؤال
نوع تصميم الدراسة
الزمن
(Resources for Evidence-Based Practice: Forming Questions, 2021)

## Slide 6
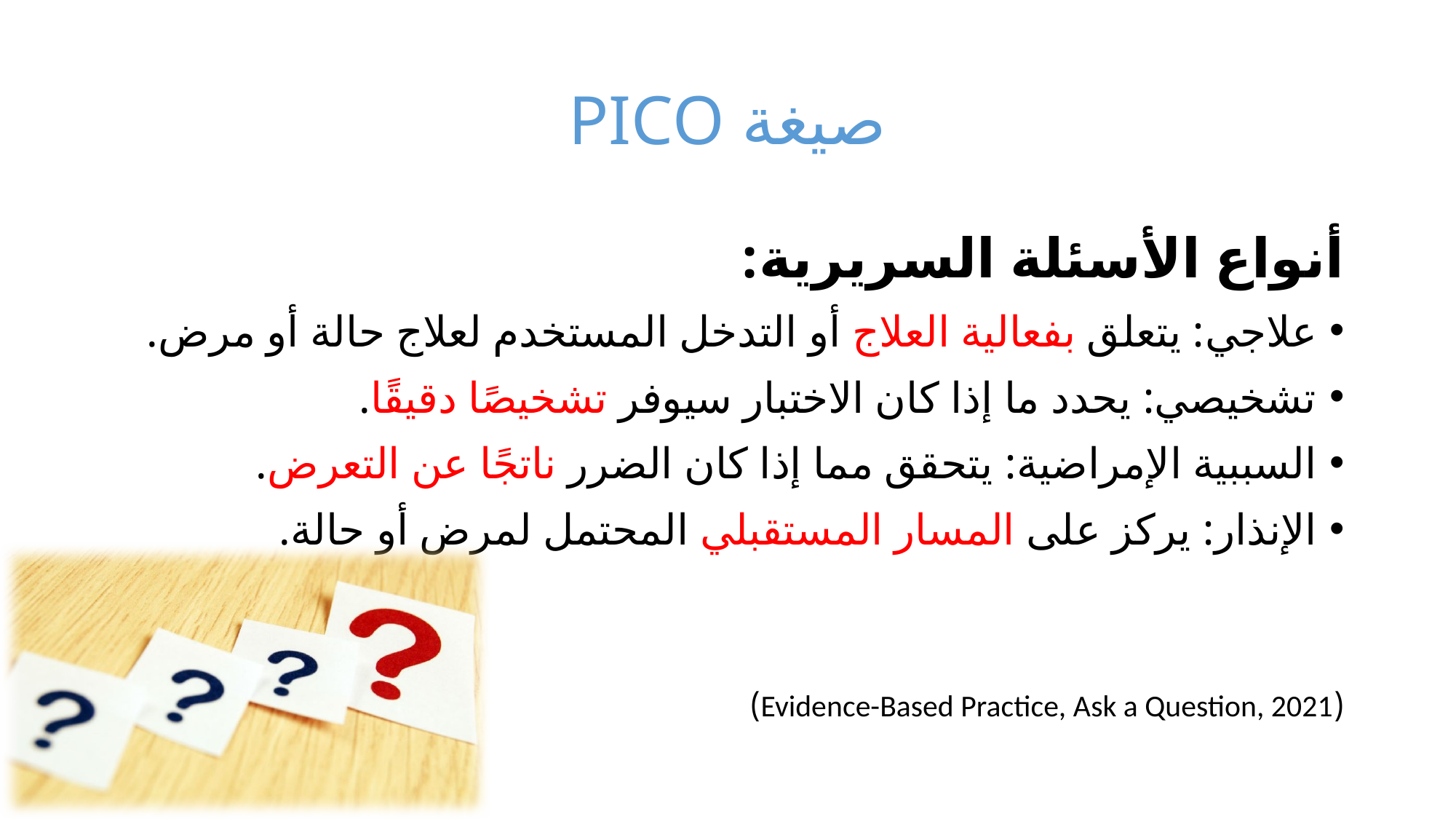

# صيغة PICO
أنواع الأسئلة السريرية:
علاجي: يتعلق بفعالية العلاج أو التدخل المستخدم لعلاج حالة أو مرض.
تشخيصي: يحدد ما إذا كان الاختبار سيوفر تشخيصًا دقيقًا.
السببية الإمراضية: يتحقق مما إذا كان الضرر ناتجًا عن التعرض.
الإنذار: يركز على المسار المستقبلي المحتمل لمرض أو حالة.
(Evidence-Based Practice, Ask a Question, 2021)

## Slide 7
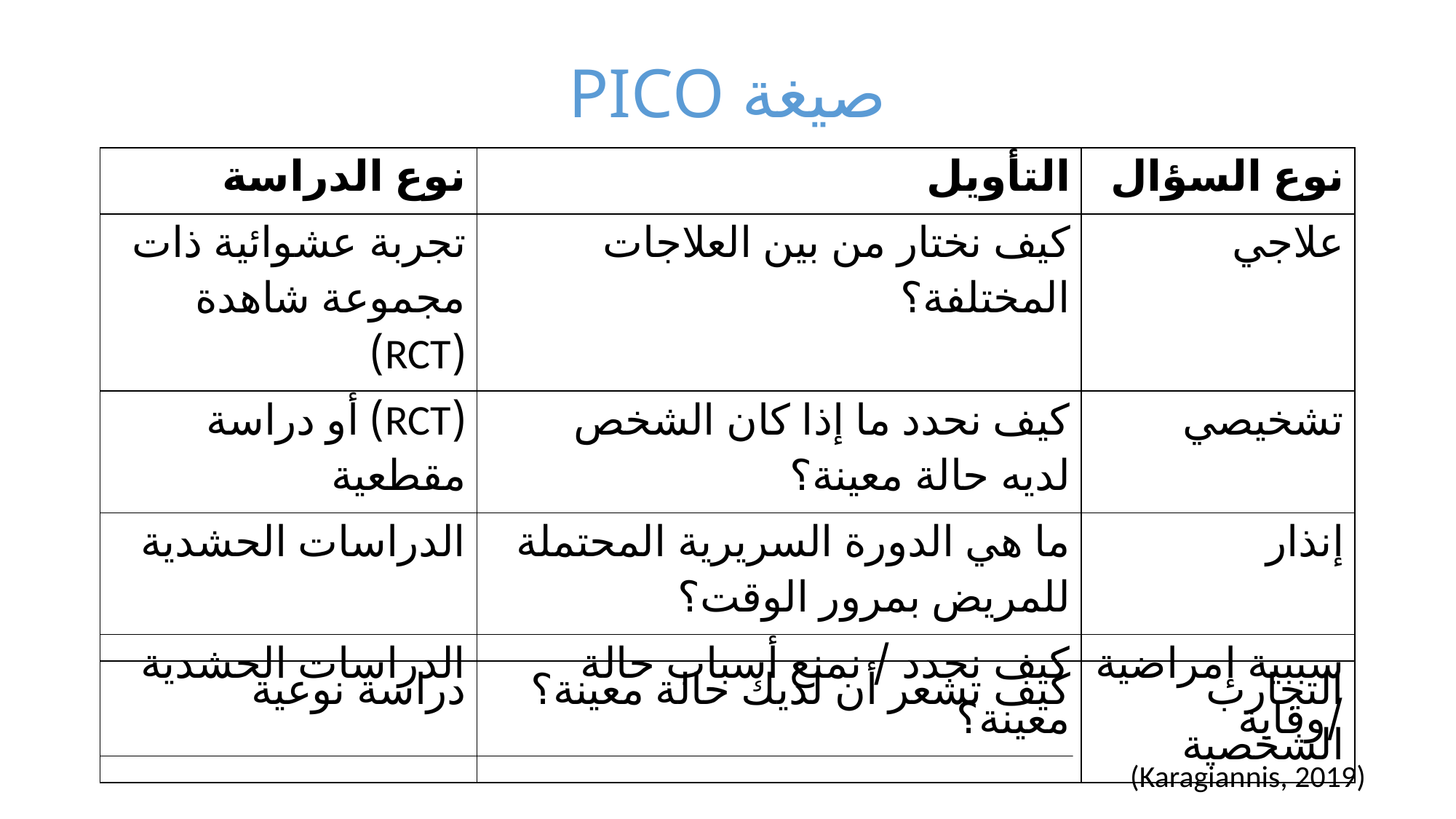

# صيغة PICO
| نوع الدراسة | التأويل | نوع السؤال |
| --- | --- | --- |
| تجربة عشوائية ذات مجموعة شاهدة (RCT) | كيف نختار من بين العلاجات المختلفة؟ | علاجي |
| (RCT) أو دراسة مقطعية | كيف نحدد ما إذا كان الشخص لديه حالة معينة؟ | تشخيصي |
| الدراسات الحشدية | ما هي الدورة السريرية المحتملة للمريض بمرور الوقت؟ | إنذار |
| الدراسات الحشدية | كيف نحدد / نمنع أسباب حالة معينة؟ | سببية إمراضية /وقاية |
| دراسة نوعية | كيف تشعر أن لديك حالة معينة؟ | التجارب الشخصية |
| --- | --- | --- |
(Karagiannis, 2019)

## Slide 8
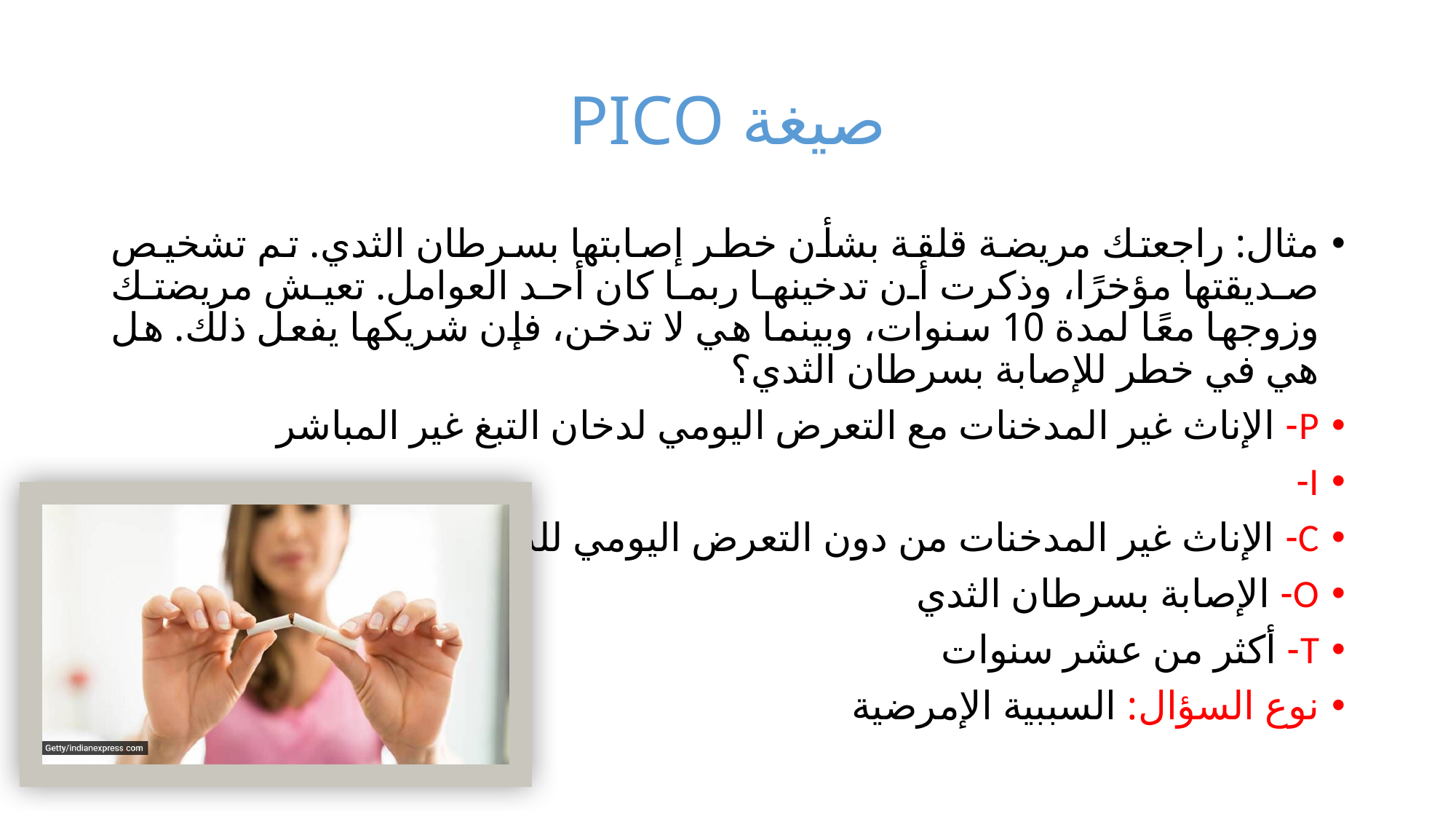

# صيغة PICO
مثال: راجعتك مريضة قلقة بشأن خطر إصابتها بسرطان الثدي. تم تشخيص صديقتها مؤخرًا، وذكرت أن تدخينها ربما كان أحد العوامل. تعيش مريضتك وزوجها معًا لمدة 10 سنوات، وبينما هي لا تدخن، فإن شريكها يفعل ذلك. هل هي في خطر للإصابة بسرطان الثدي؟
P- الإناث غير المدخنات مع التعرض اليومي لدخان التبغ غير المباشر
I-
C- الإناث غير المدخنات من دون التعرض اليومي للدخان
O- الإصابة بسرطان الثدي
T- أكثر من عشر سنوات
نوع السؤال: السببية الإمرضية

## Slide 9
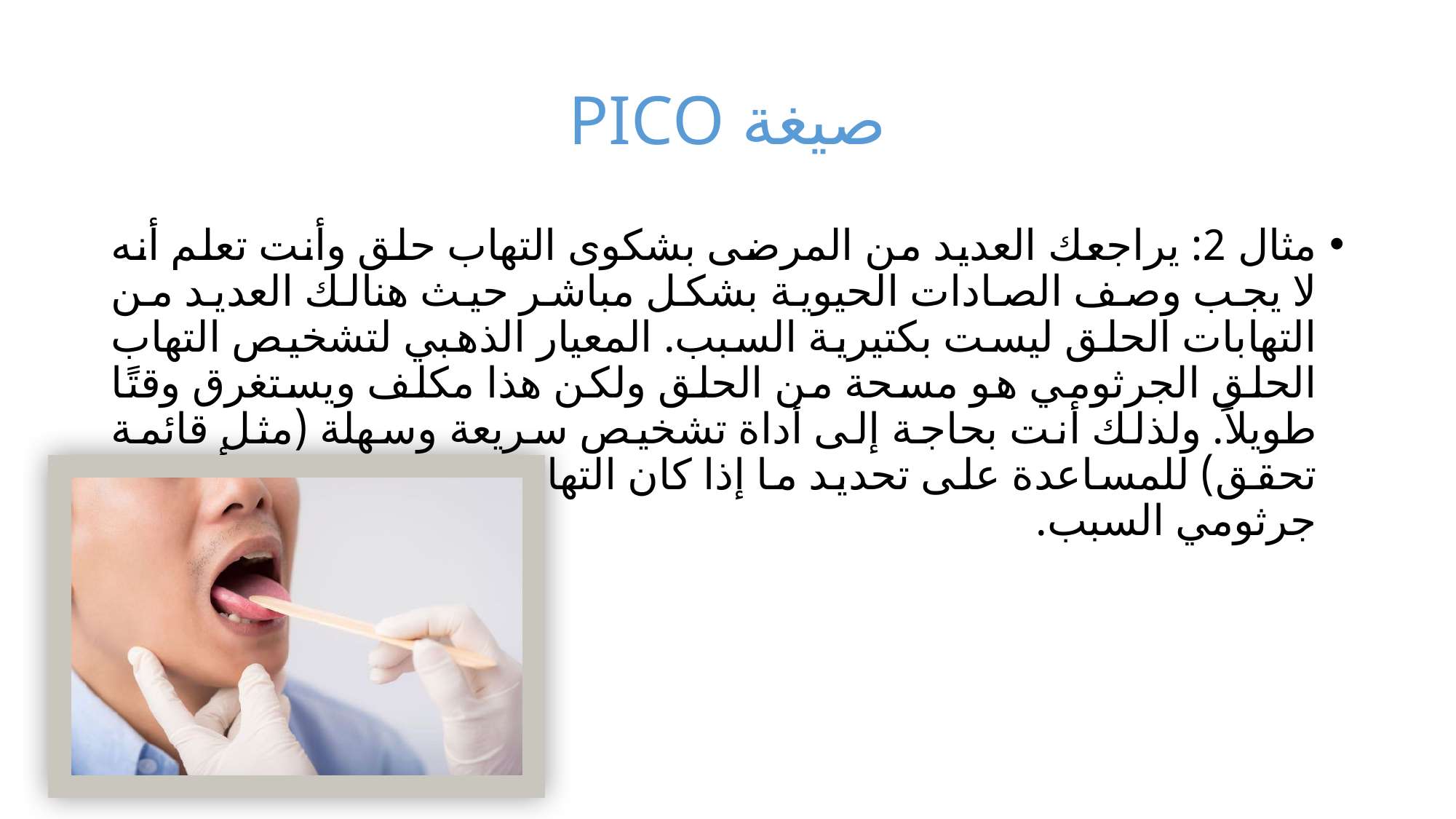

# صيغة PICO
مثال 2: يراجعك العديد من المرضى بشكوى التهاب حلق وأنت تعلم أنه لا يجب وصف الصادات الحيوية بشكل مباشر حيث هنالك العديد من التهابات الحلق ليست بكتيرية السبب. المعيار الذهبي لتشخيص التهاب الحلق الجرثومي هو مسحة من الحلق ولكن هذا مكلف ويستغرق وقتًا طويلاً. ولذلك أنت بحاجة إلى أداة تشخيص سريعة وسهلة (مثل قائمة تحقق) للمساعدة على تحديد ما إذا كان التهاب الحلق جرثوميًا أم غير جرثومي السبب.

## Slide 10
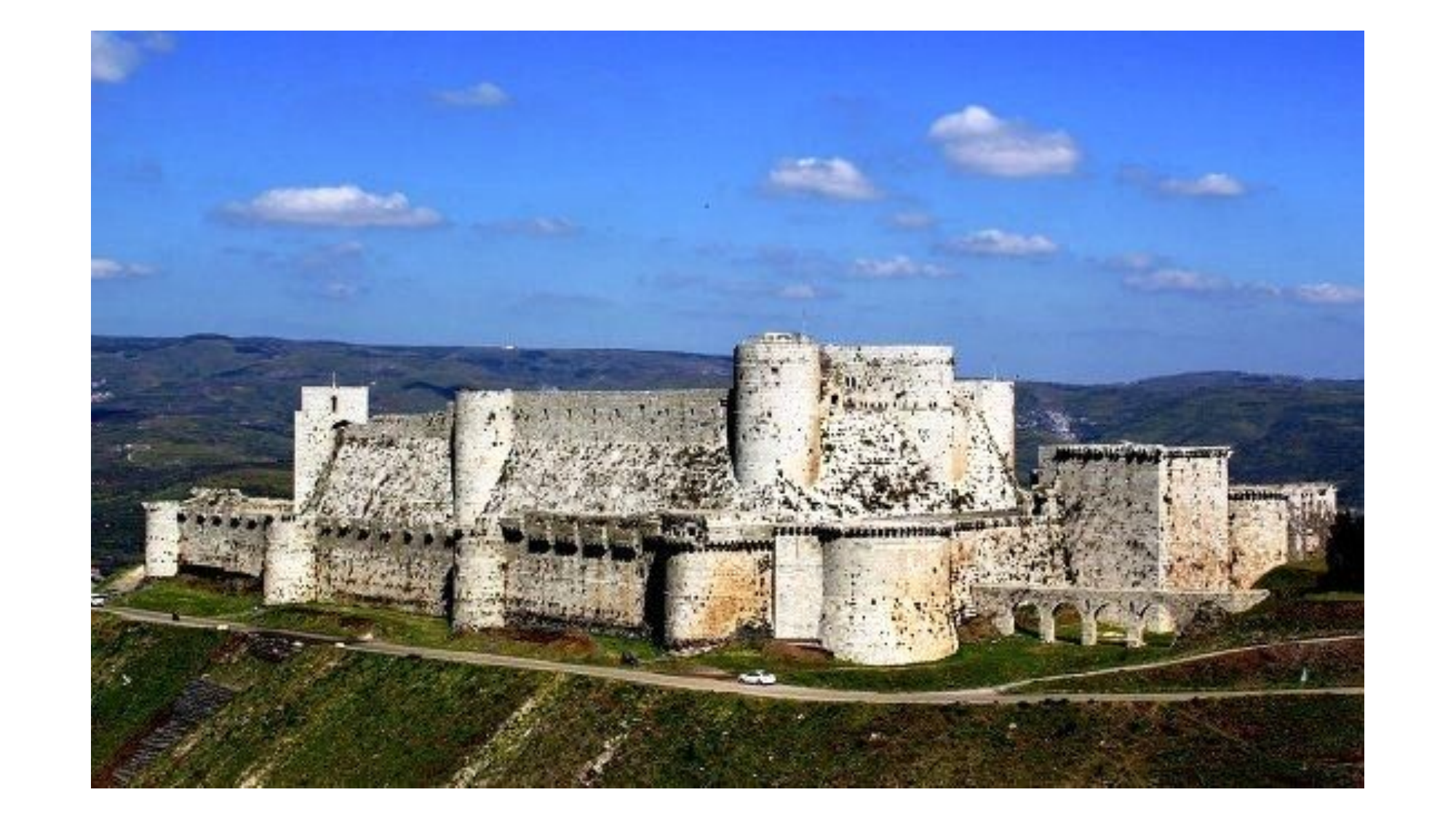

## Slide 11
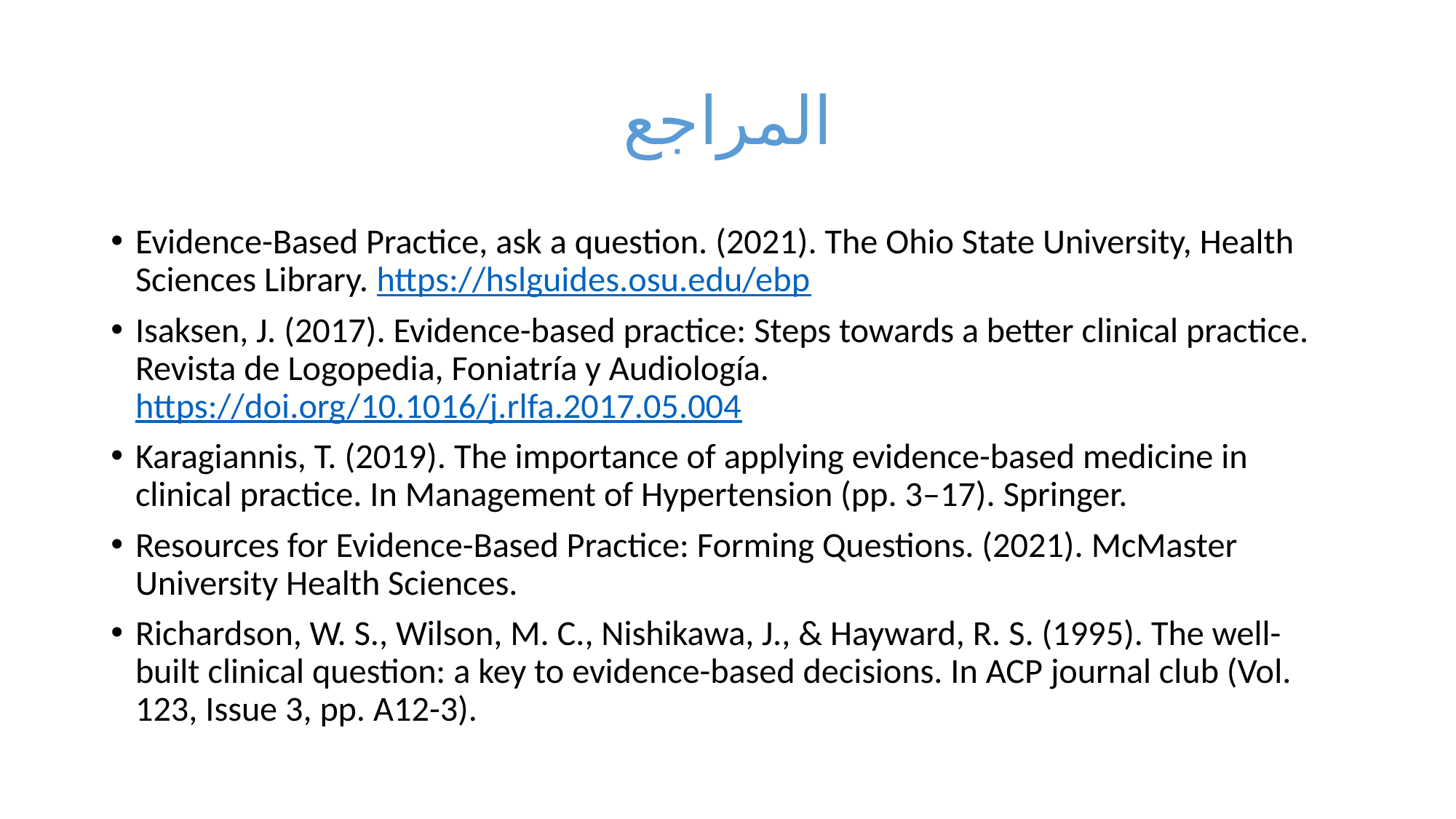

# المراجع
Evidence-Based Practice, ask a question. (2021). The Ohio State University, Health Sciences Library. https://hslguides.osu.edu/ebp
Isaksen, J. (2017). Evidence-based practice: Steps towards a better clinical practice. Revista de Logopedia, Foniatría y Audiología. https://doi.org/10.1016/j.rlfa.2017.05.004
Karagiannis, T. (2019). The importance of applying evidence-based medicine in clinical practice. In Management of Hypertension (pp. 3–17). Springer.
Resources for Evidence-Based Practice: Forming Questions. (2021). McMaster University Health Sciences.
Richardson, W. S., Wilson, M. C., Nishikawa, J., & Hayward, R. S. (1995). The well-built clinical question: a key to evidence-based decisions. In ACP journal club (Vol. 123, Issue 3, pp. A12-3).
