# Supplementary material for: The First Asynchronous Online Evidence-Based Medicine Course for Syrian Health Workforce: Effectiveness and Feasibility Pilot Study
Source: JMIR Form Res. 2022 Oct 25;6(10):e36782. doi: 10.2196/36782 (PMC9644249; doi:10.2196/36782)
Supplement: Multimedia Appendix 5 [file formative_v6i10e36782_app5.pptx]

## Slide 1
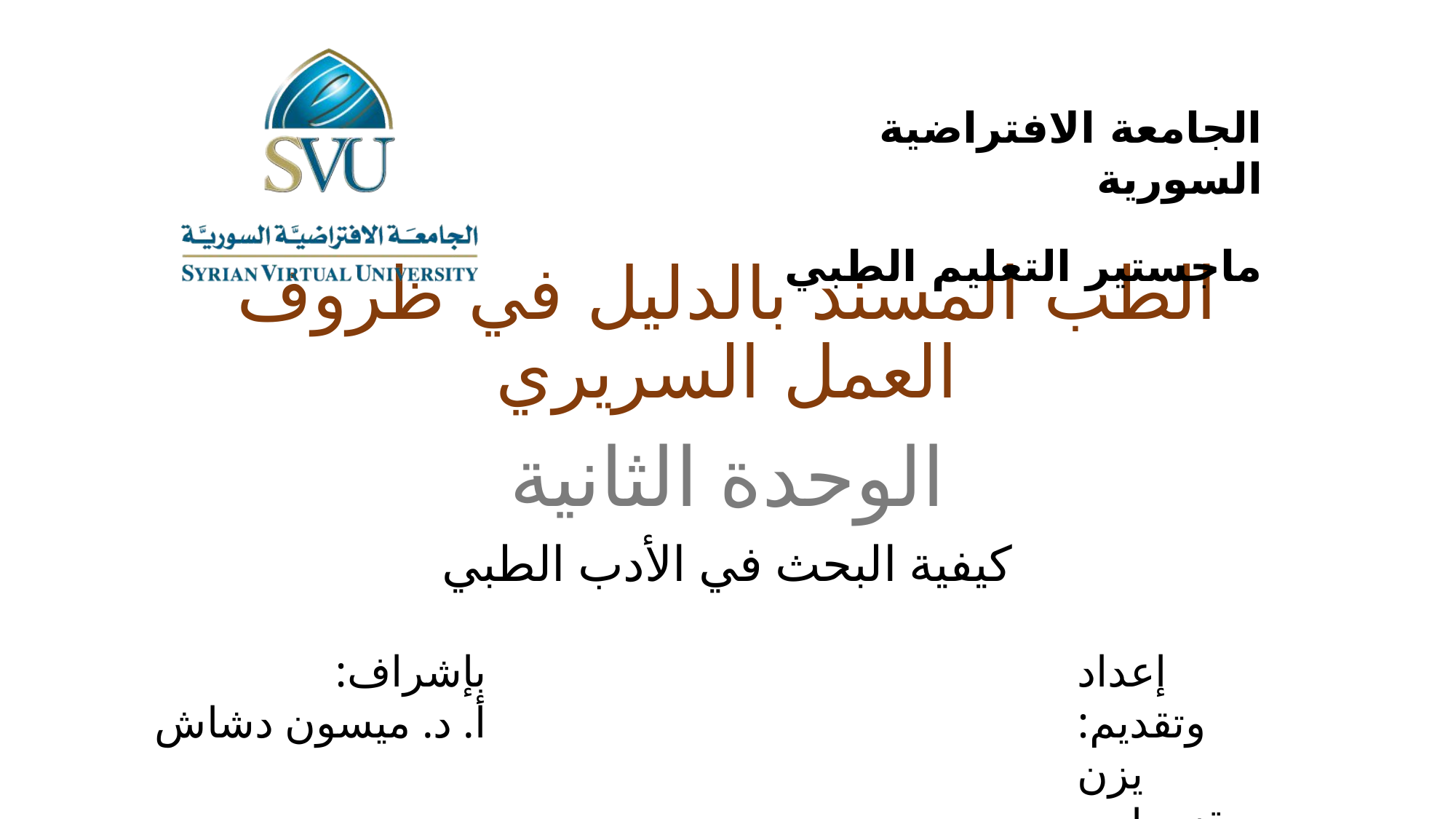

الجامعة الافتراضية السورية
ماجستير التعليم الطبي
# الطب المسند بالدليل في ظروف العمل السريري
الوحدة الثانية
كيفية البحث في الأدب الطبي
إعداد وتقديم:
يزن قنجراوي
بإشراف:
أ. د. ميسون دشاش

## Slide 2
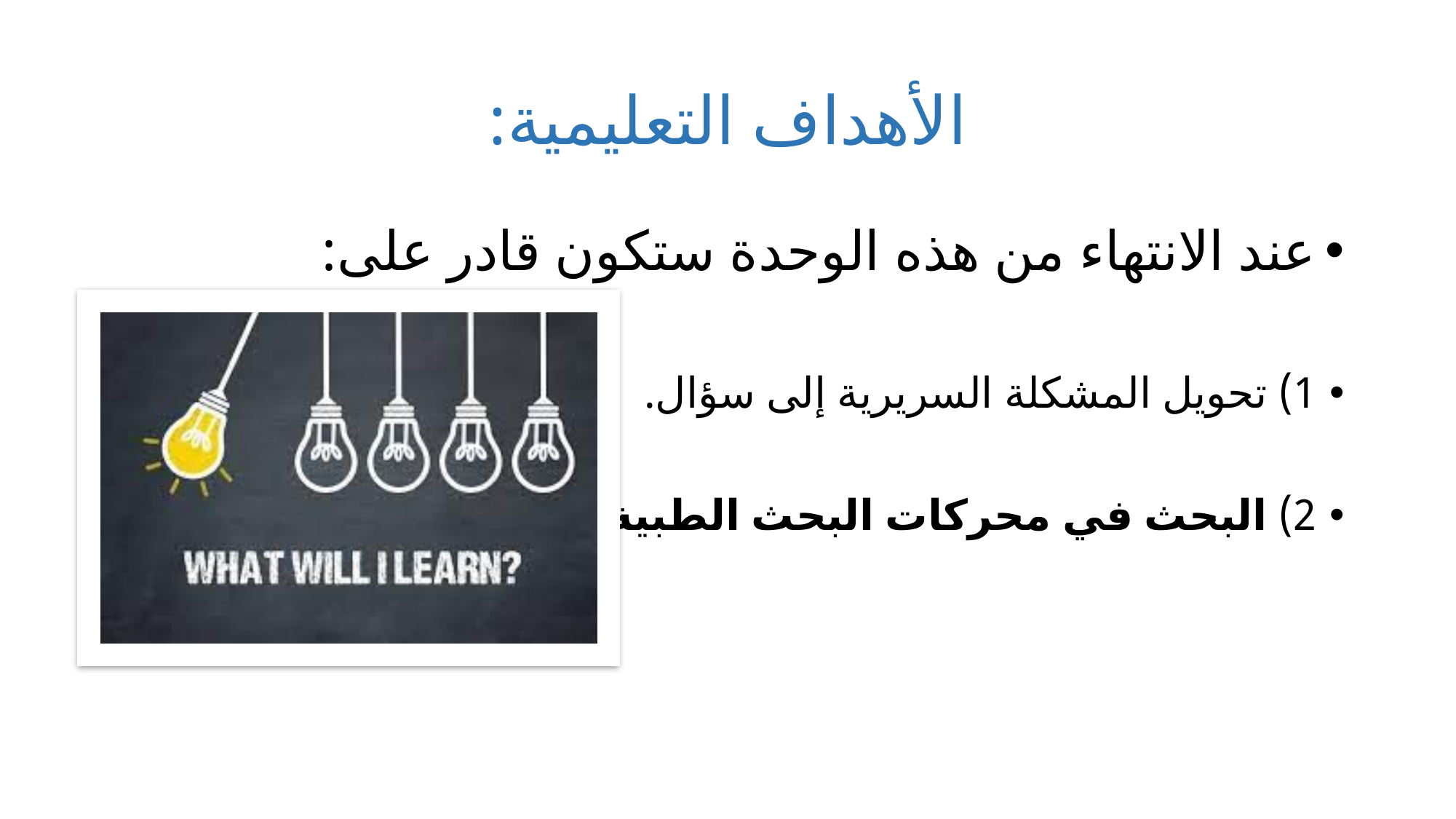

# الأهداف التعليمية:
عند الانتهاء من هذه الوحدة ستكون قادر على:
1) تحويل المشكلة السريرية إلى سؤال.
2) البحث في محركات البحث الطبية.

## Slide 3
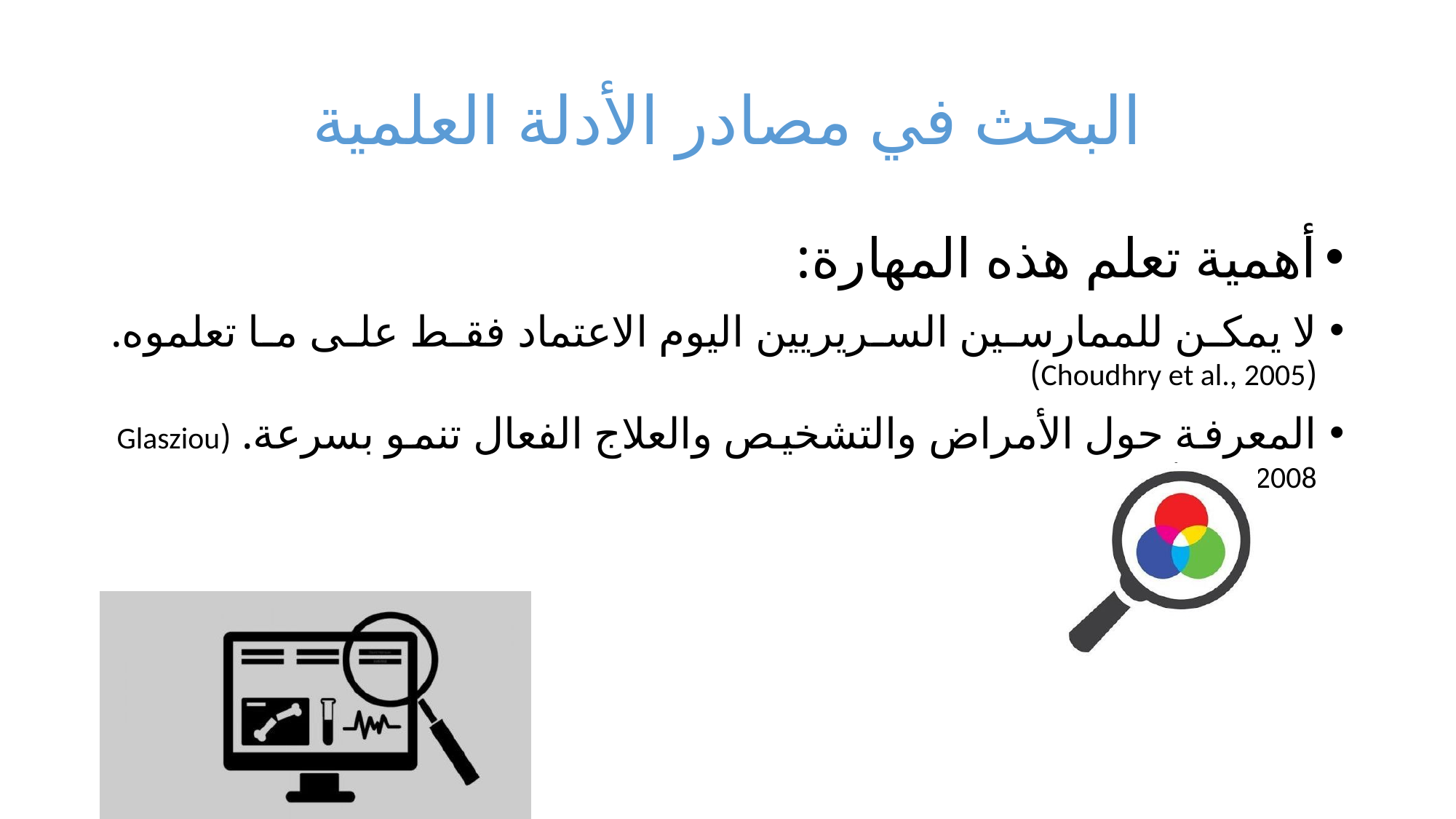

# البحث في مصادر الأدلة العلمية
أهمية تعلم هذه المهارة:
لا يمكن للممارسين السريريين اليوم الاعتماد فقط على ما تعلموه. (Choudhry et al., 2005)
المعرفة حول الأمراض والتشخيص والعلاج الفعال تنمو بسرعة. (Glasziou et al., 2008)

## Slide 4
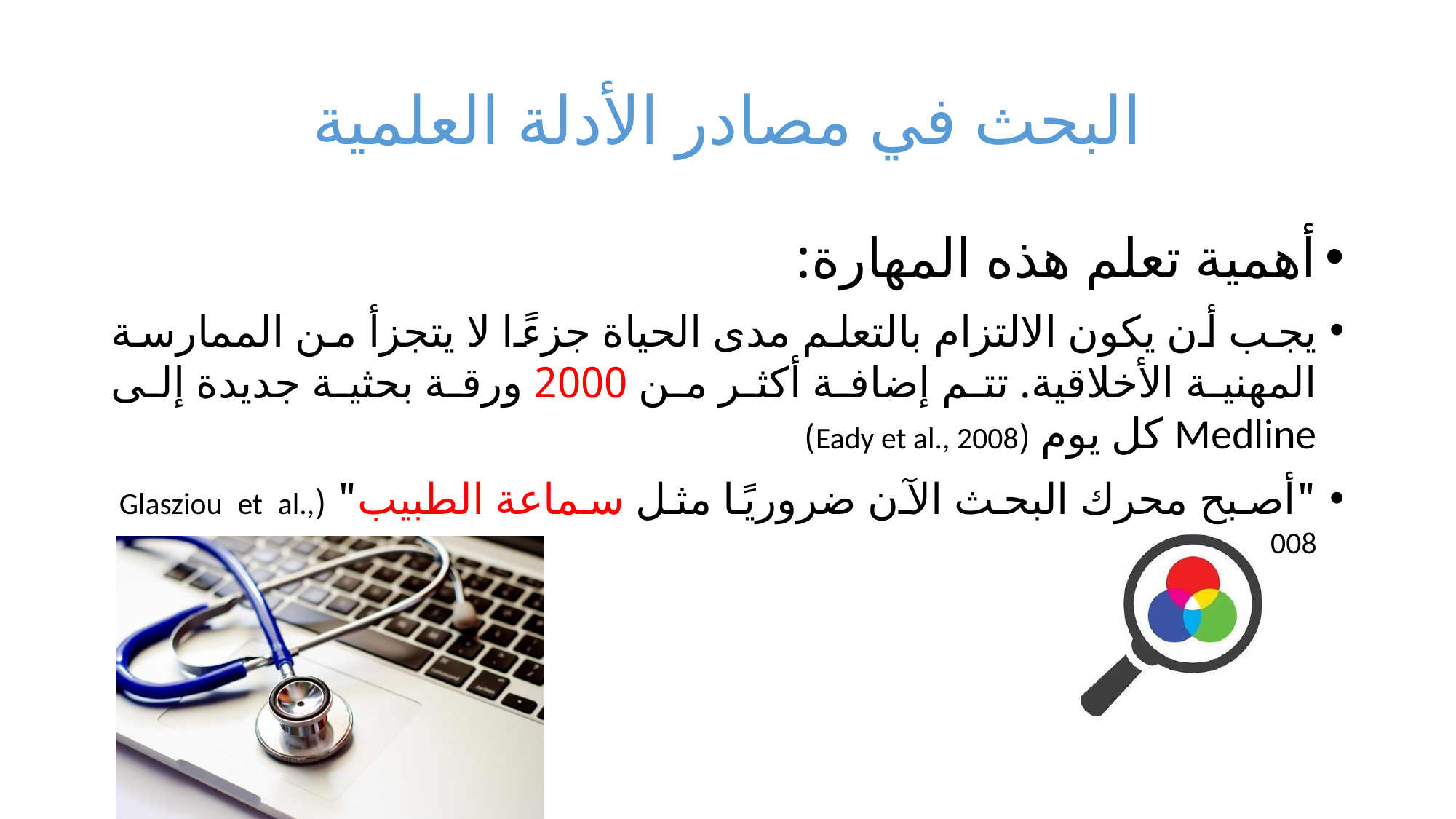

# البحث في مصادر الأدلة العلمية
أهمية تعلم هذه المهارة:
يجب أن يكون الالتزام بالتعلم مدى الحياة جزءًا لا يتجزأ من الممارسة المهنية الأخلاقية. تتم إضافة أكثر من 2000 ورقة بحثية جديدة إلى Medline كل يوم (Eady et al., 2008)
"أصبح محرك البحث الآن ضروريًا مثل سماعة الطبيب" (Glasziou et al., 2008)

## Slide 5
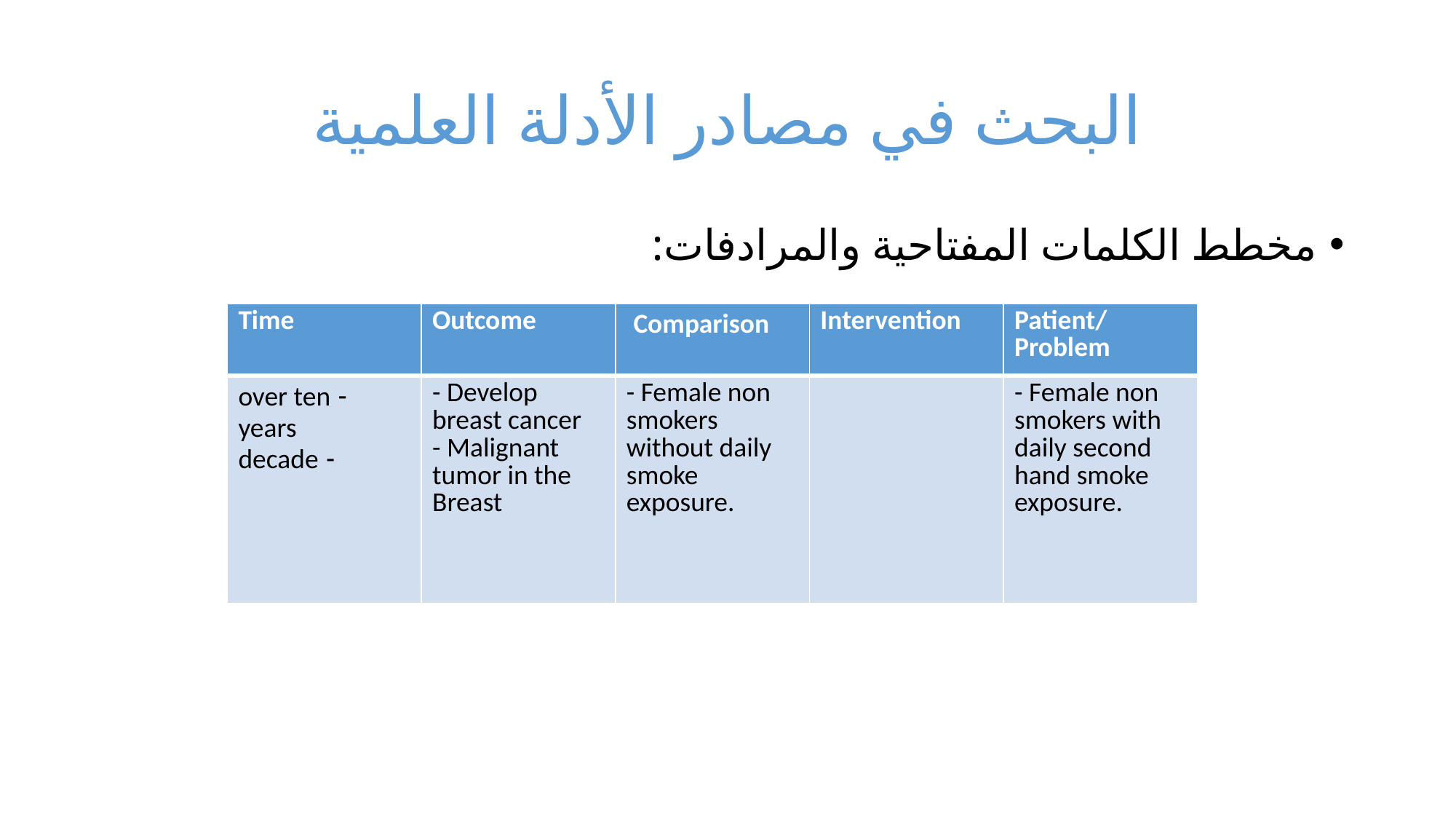

# البحث في مصادر الأدلة العلمية
مخطط الكلمات المفتاحية والمرادفات:
| Time | Outcome | Comparison | Intervention | Patient/ Problem |
| --- | --- | --- | --- | --- |
| - over ten years - decade | - Develop breast cancer - Malignant tumor in the Breast | - Female non smokers without daily smoke exposure. | | - Female non smokers with daily second hand smoke exposure. |

## Slide 6
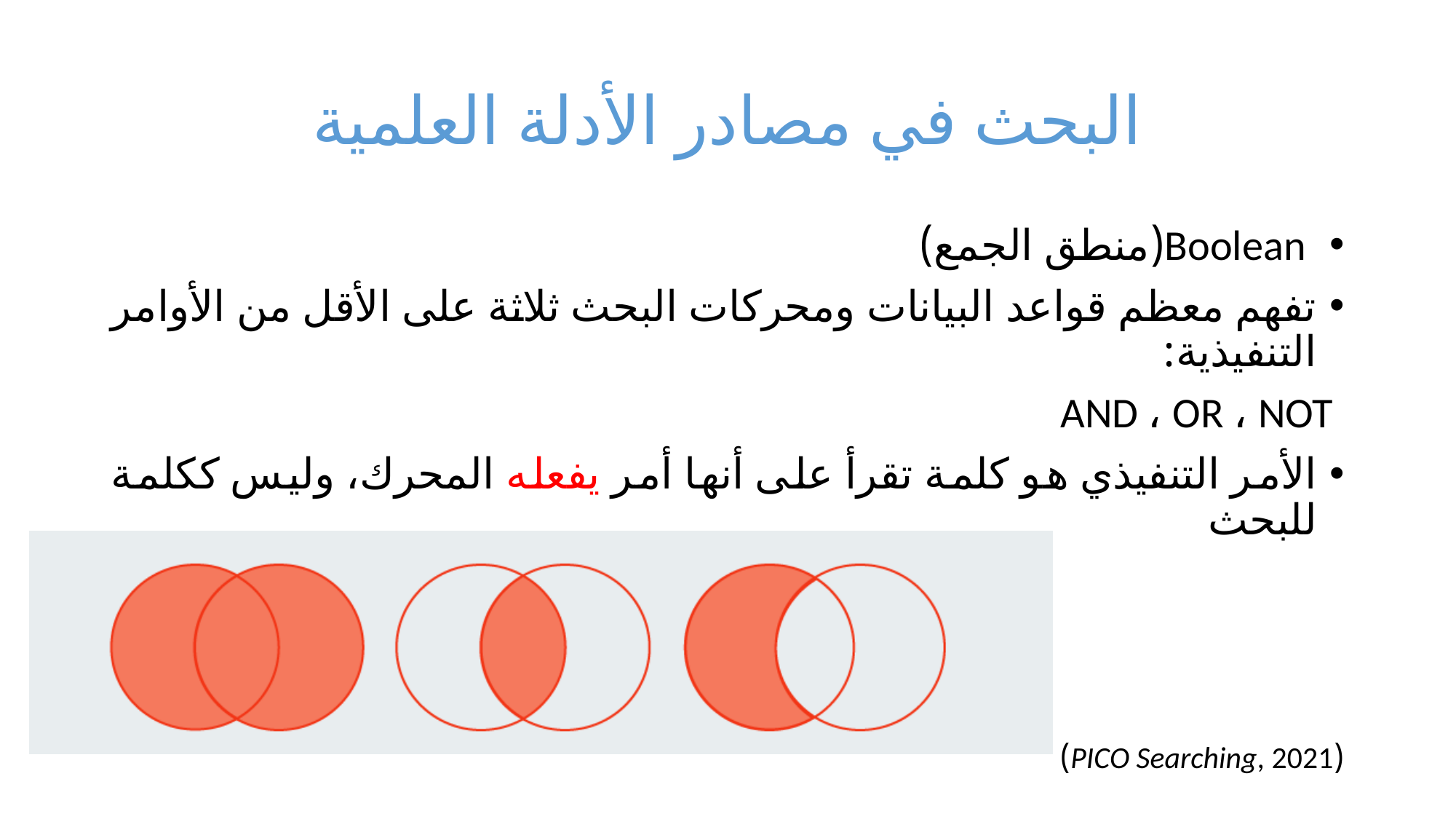

# البحث في مصادر الأدلة العلمية
 Boolean(منطق الجمع)
تفهم معظم قواعد البيانات ومحركات البحث ثلاثة على الأقل من الأوامر التنفيذية:
 AND ، OR ، NOT
الأمر التنفيذي هو كلمة تقرأ على أنها أمر يفعله المحرك، وليس ككلمة للبحث
(PICO Searching, 2021)

## Slide 7
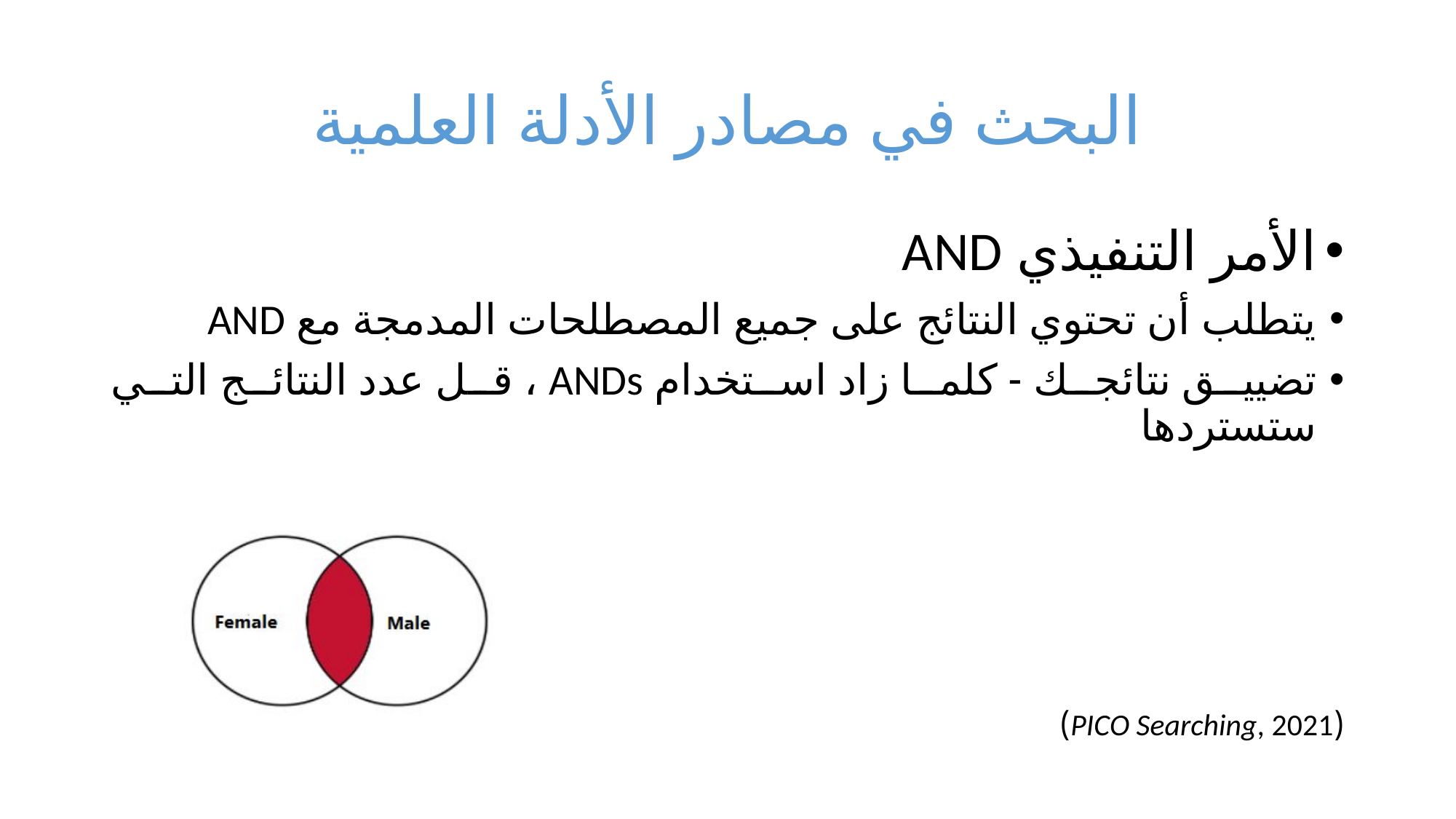

# البحث في مصادر الأدلة العلمية
الأمر التنفيذي AND
يتطلب أن تحتوي النتائج على جميع المصطلحات المدمجة مع AND
تضييق نتائجك - كلما زاد استخدام ANDs ، قل عدد النتائج التي ستستردها
(PICO Searching, 2021)

## Slide 8
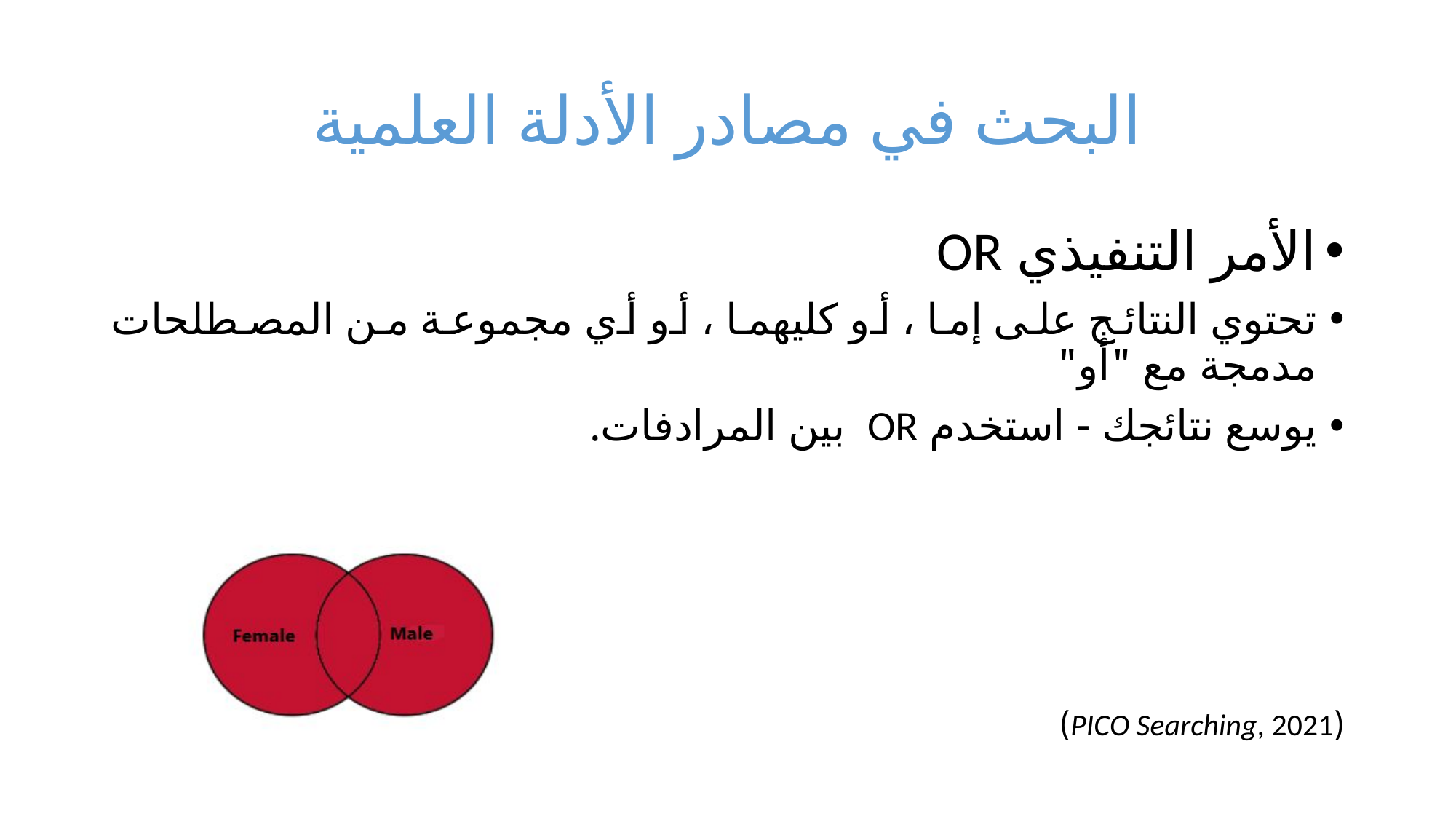

# البحث في مصادر الأدلة العلمية
الأمر التنفيذي OR
تحتوي النتائج على إما ، أو كليهما ، أو أي مجموعة من المصطلحات مدمجة مع "أو"
يوسع نتائجك - استخدم OR بين المرادفات.
(PICO Searching, 2021)

## Slide 9
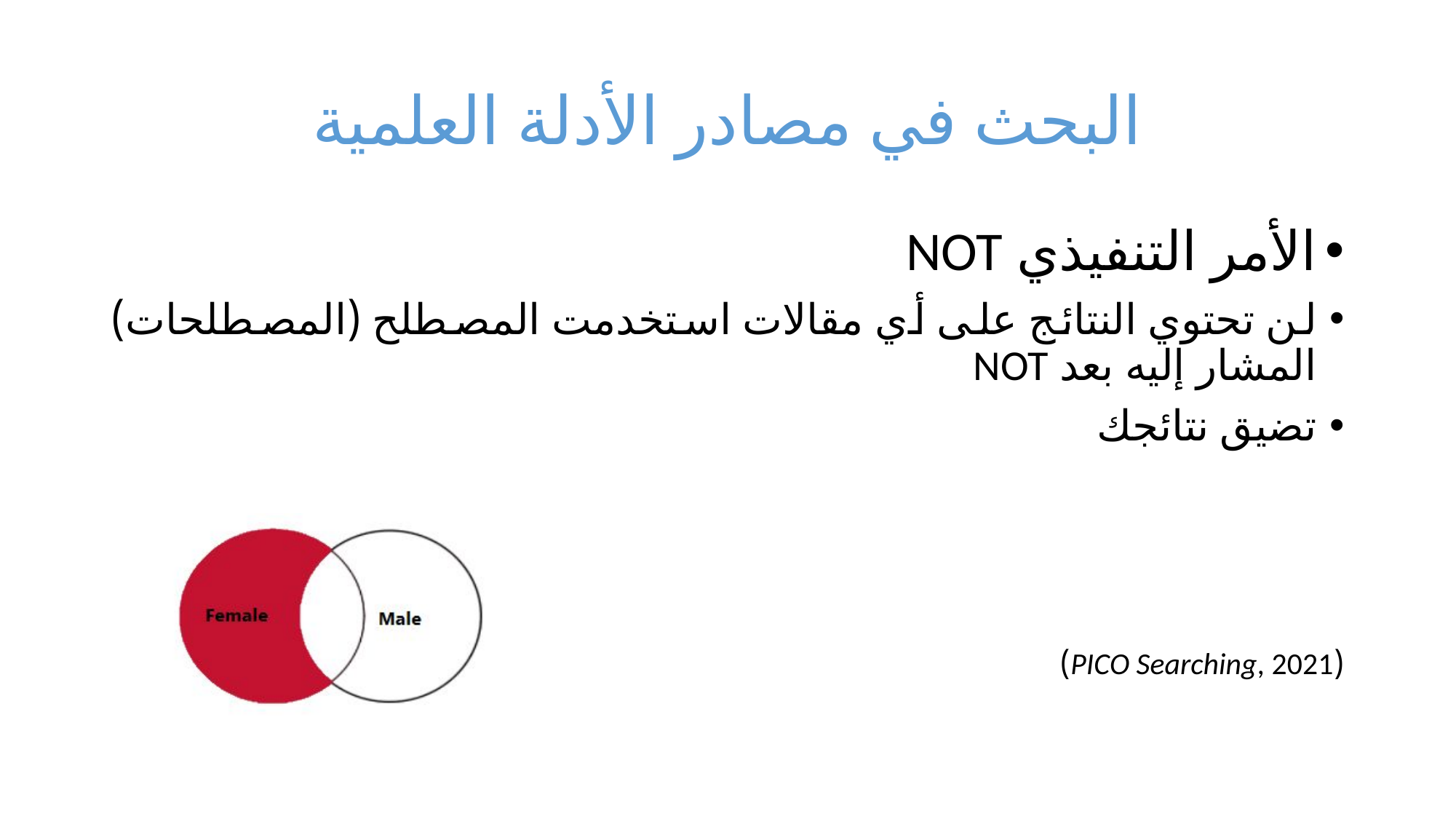

# البحث في مصادر الأدلة العلمية
الأمر التنفيذي NOT
لن تحتوي النتائج على أي مقالات استخدمت المصطلح (المصطلحات) المشار إليه بعد NOT
تضيق نتائجك
(PICO Searching, 2021)

## Slide 10
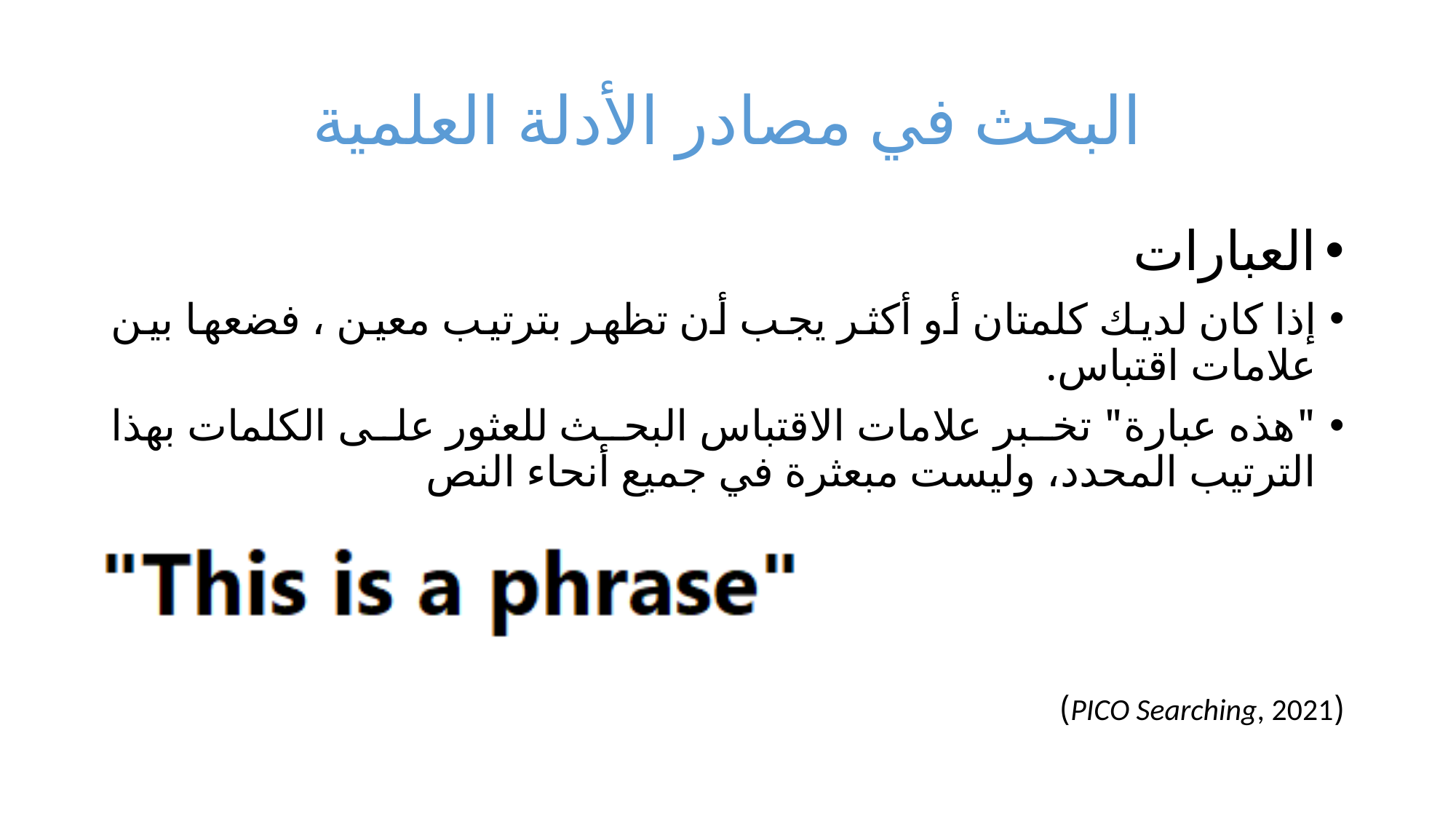

# البحث في مصادر الأدلة العلمية
العبارات
إذا كان لديك كلمتان أو أكثر يجب أن تظهر بترتيب معين ، فضعها بين علامات اقتباس.
"هذه عبارة" تخبر علامات الاقتباس البحث للعثور على الكلمات بهذا الترتيب المحدد، وليست مبعثرة في جميع أنحاء النص
(PICO Searching, 2021)

## Slide 11
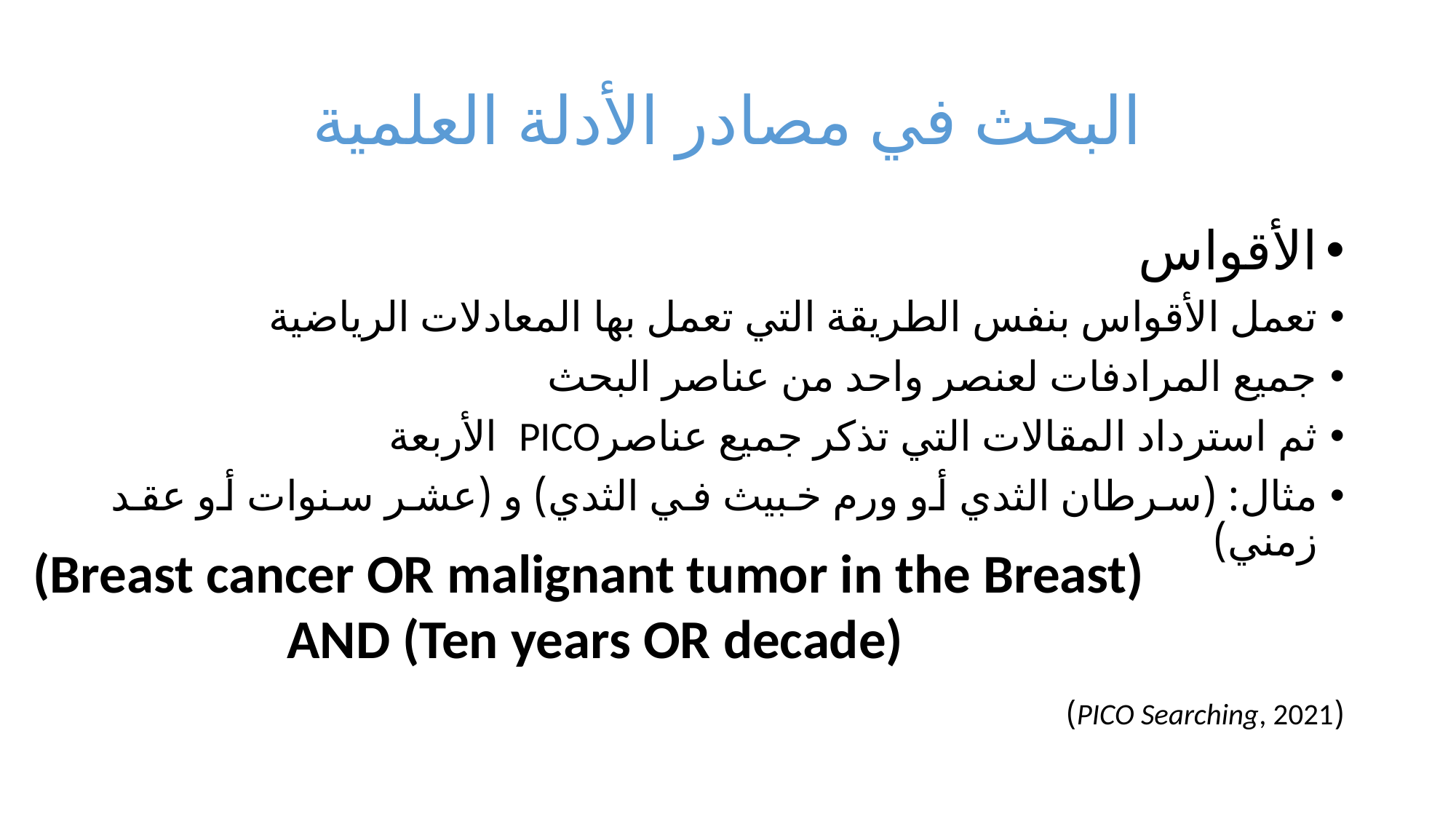

# البحث في مصادر الأدلة العلمية
الأقواس
تعمل الأقواس بنفس الطريقة التي تعمل بها المعادلات الرياضية
جميع المرادفات لعنصر واحد من عناصر البحث
ثم استرداد المقالات التي تذكر جميع عناصرPICO الأربعة
مثال: (سرطان الثدي أو ورم خبيث في الثدي) و (عشر سنوات أو عقد زمني)
(PICO Searching, 2021)
(Breast cancer OR malignant tumor in the Breast)
AND (Ten years OR decade)

## Slide 12
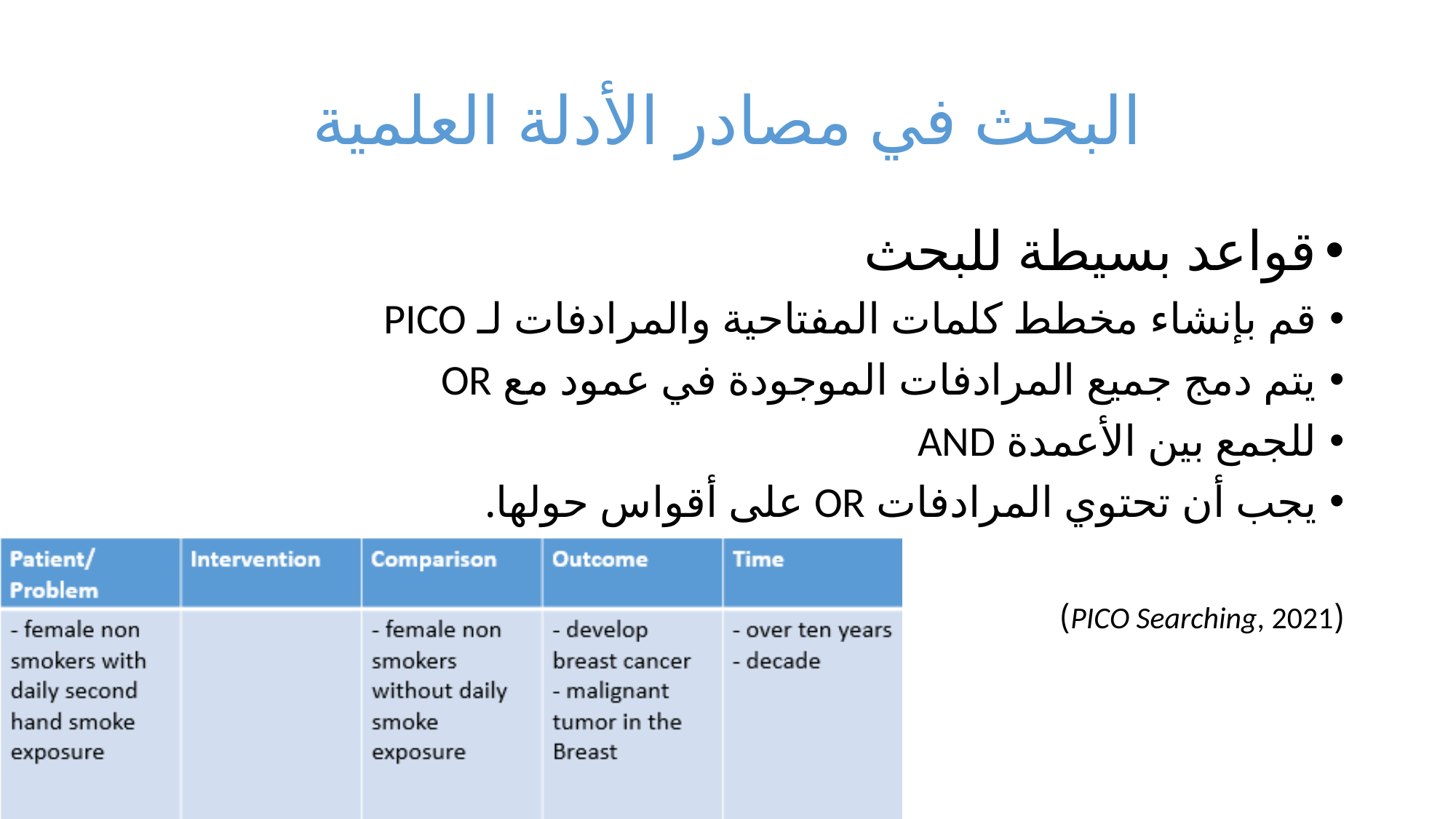

# البحث في مصادر الأدلة العلمية
قواعد بسيطة للبحث
قم بإنشاء مخطط كلمات المفتاحية والمرادفات لـ PICO
يتم دمج جميع المرادفات الموجودة في عمود مع OR
للجمع بين الأعمدة AND
يجب أن تحتوي المرادفات OR على أقواس حولها.
(PICO Searching, 2021)

## Slide 13
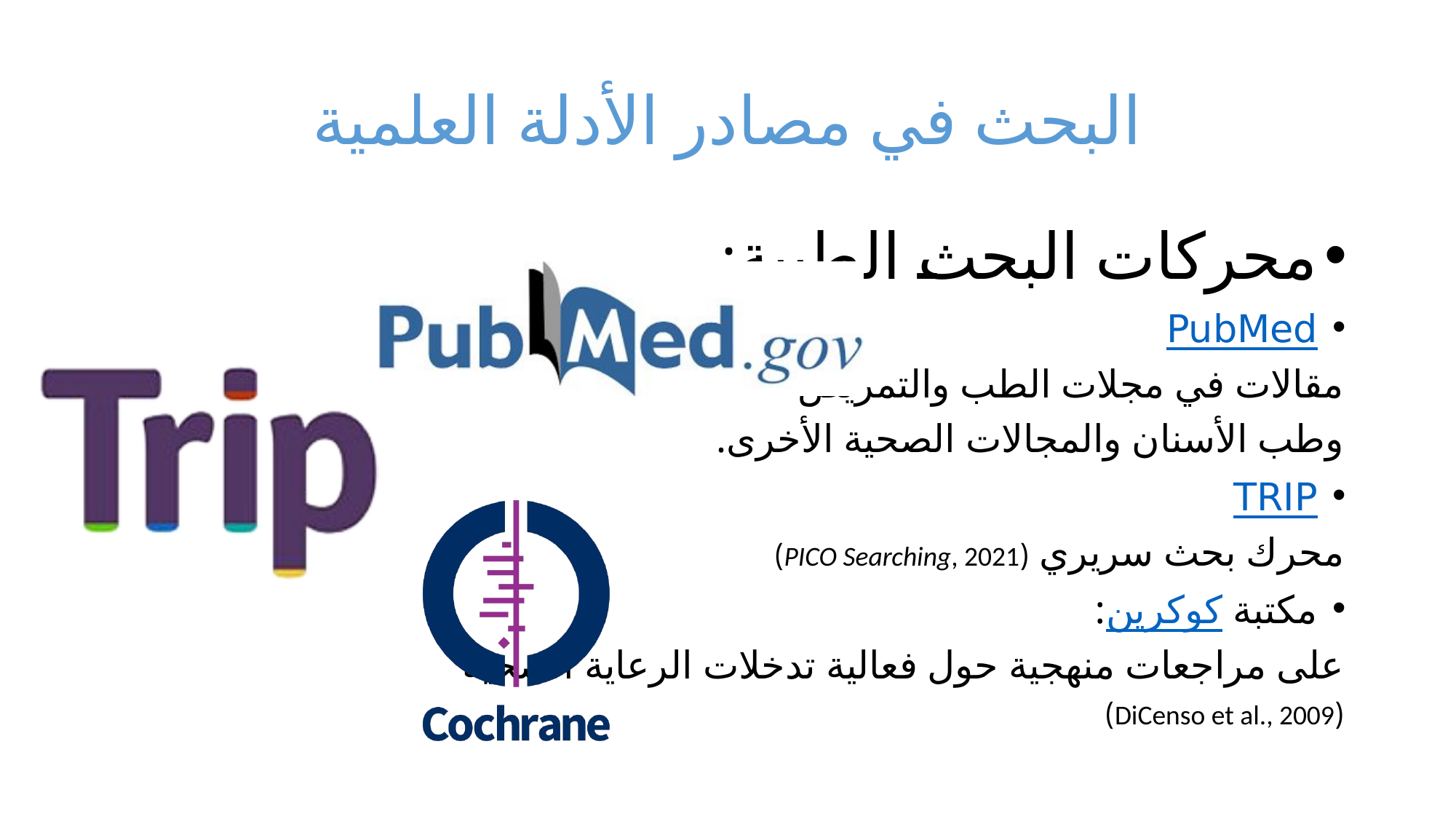

# البحث في مصادر الأدلة العلمية
محركات البحث الطبية:
PubMed
مقالات في مجلات الطب والتمريض
وطب الأسنان والمجالات الصحية الأخرى.
TRIP
محرك بحث سريري (PICO Searching, 2021)
مكتبة كوكرين:
على مراجعات منهجية حول فعالية تدخلات الرعاية الصحية
(DiCenso et al., 2009)

## Slide 14
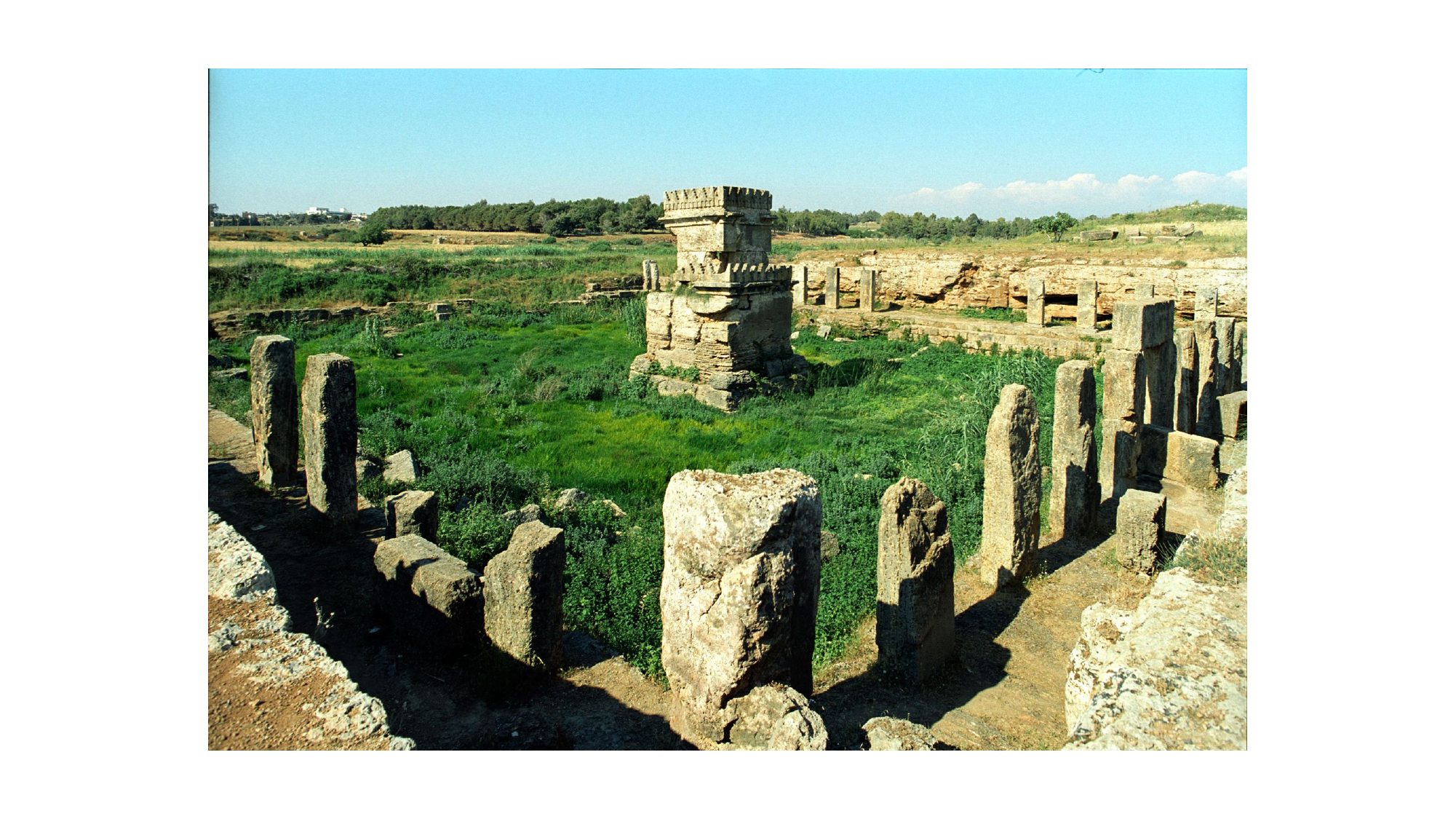

## Slide 15
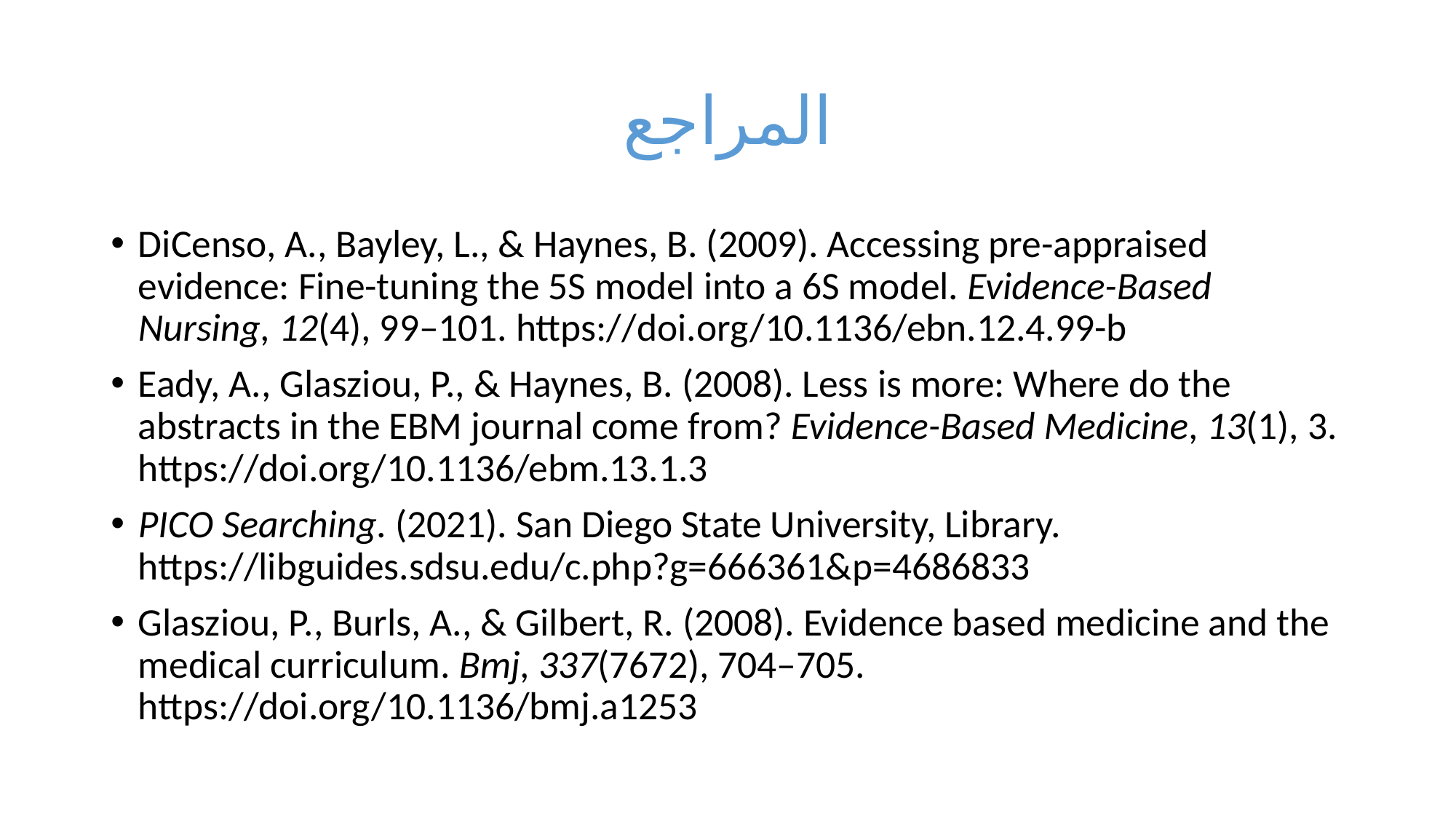

# المراجع
DiCenso, A., Bayley, L., & Haynes, B. (2009). Accessing pre-appraised evidence: Fine-tuning the 5S model into a 6S model. Evidence-Based Nursing, 12(4), 99–101. https://doi.org/10.1136/ebn.12.4.99-b
Eady, A., Glasziou, P., & Haynes, B. (2008). Less is more: Where do the abstracts in the EBM journal come from? Evidence-Based Medicine, 13(1), 3. https://doi.org/10.1136/ebm.13.1.3
PICO Searching. (2021). San Diego State University, Library. https://libguides.sdsu.edu/c.php?g=666361&p=4686833
Glasziou, P., Burls, A., & Gilbert, R. (2008). Evidence based medicine and the medical curriculum. Bmj, 337(7672), 704–705. https://doi.org/10.1136/bmj.a1253
