# Supplementary material for: The First Asynchronous Online Evidence-Based Medicine Course for Syrian Health Workforce: Effectiveness and Feasibility Pilot Study
Source: JMIR Form Res. 2022 Oct 25;6(10):e36782. doi: 10.2196/36782 (PMC9644249; doi:10.2196/36782)
Supplement: Multimedia Appendix 6 [file formative_v6i10e36782_app6.pptx]

## Slide 1
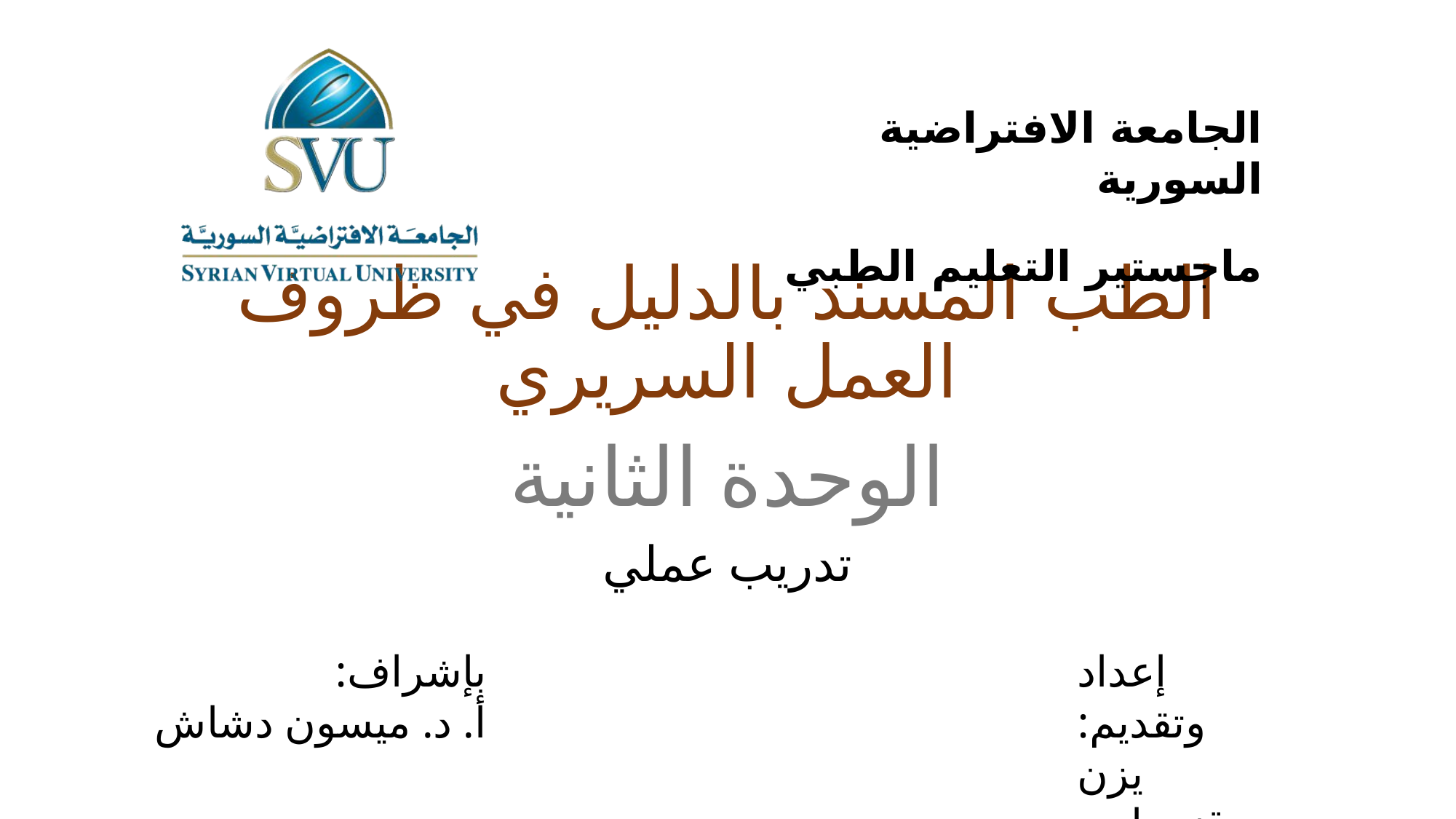

الجامعة الافتراضية السورية
ماجستير التعليم الطبي
# الطب المسند بالدليل في ظروف العمل السريري
الوحدة الثانية
تدريب عملي
إعداد وتقديم:
يزن قنجراوي
بإشراف:
أ. د. ميسون دشاش

## Slide 2
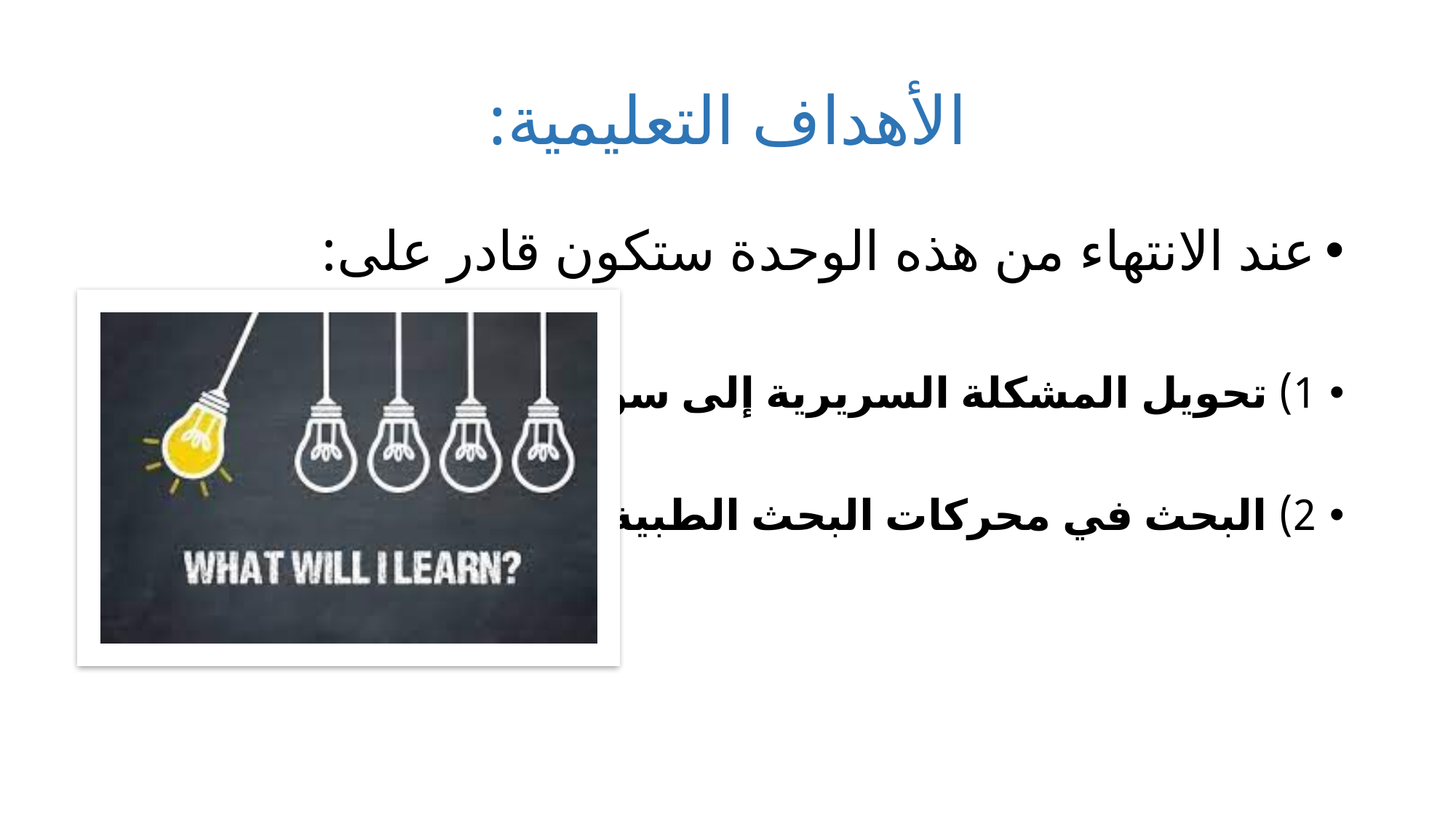

# الأهداف التعليمية:
عند الانتهاء من هذه الوحدة ستكون قادر على:
1) تحويل المشكلة السريرية إلى سؤال.
2) البحث في محركات البحث الطبية.

## Slide 3
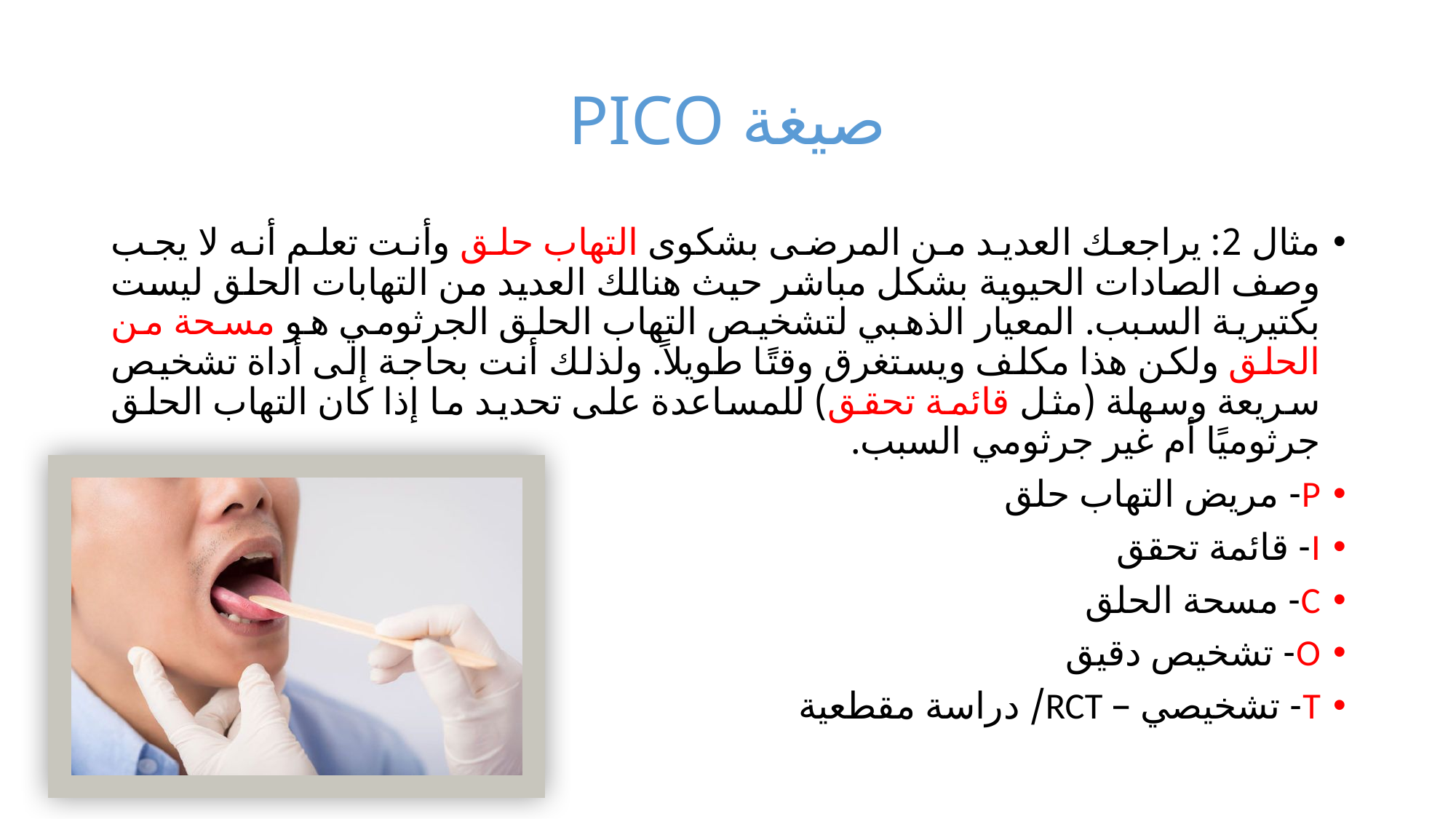

# صيغة PICO
مثال 2: يراجعك العديد من المرضى بشكوى التهاب حلق وأنت تعلم أنه لا يجب وصف الصادات الحيوية بشكل مباشر حيث هنالك العديد من التهابات الحلق ليست بكتيرية السبب. المعيار الذهبي لتشخيص التهاب الحلق الجرثومي هو مسحة من الحلق ولكن هذا مكلف ويستغرق وقتًا طويلاً. ولذلك أنت بحاجة إلى أداة تشخيص سريعة وسهلة (مثل قائمة تحقق) للمساعدة على تحديد ما إذا كان التهاب الحلق جرثوميًا أم غير جرثومي السبب.
P- مريض التهاب حلق
I- قائمة تحقق
C- مسحة الحلق
O- تشخيص دقيق
T- تشخيصي – RCT/ دراسة مقطعية

## Slide 4
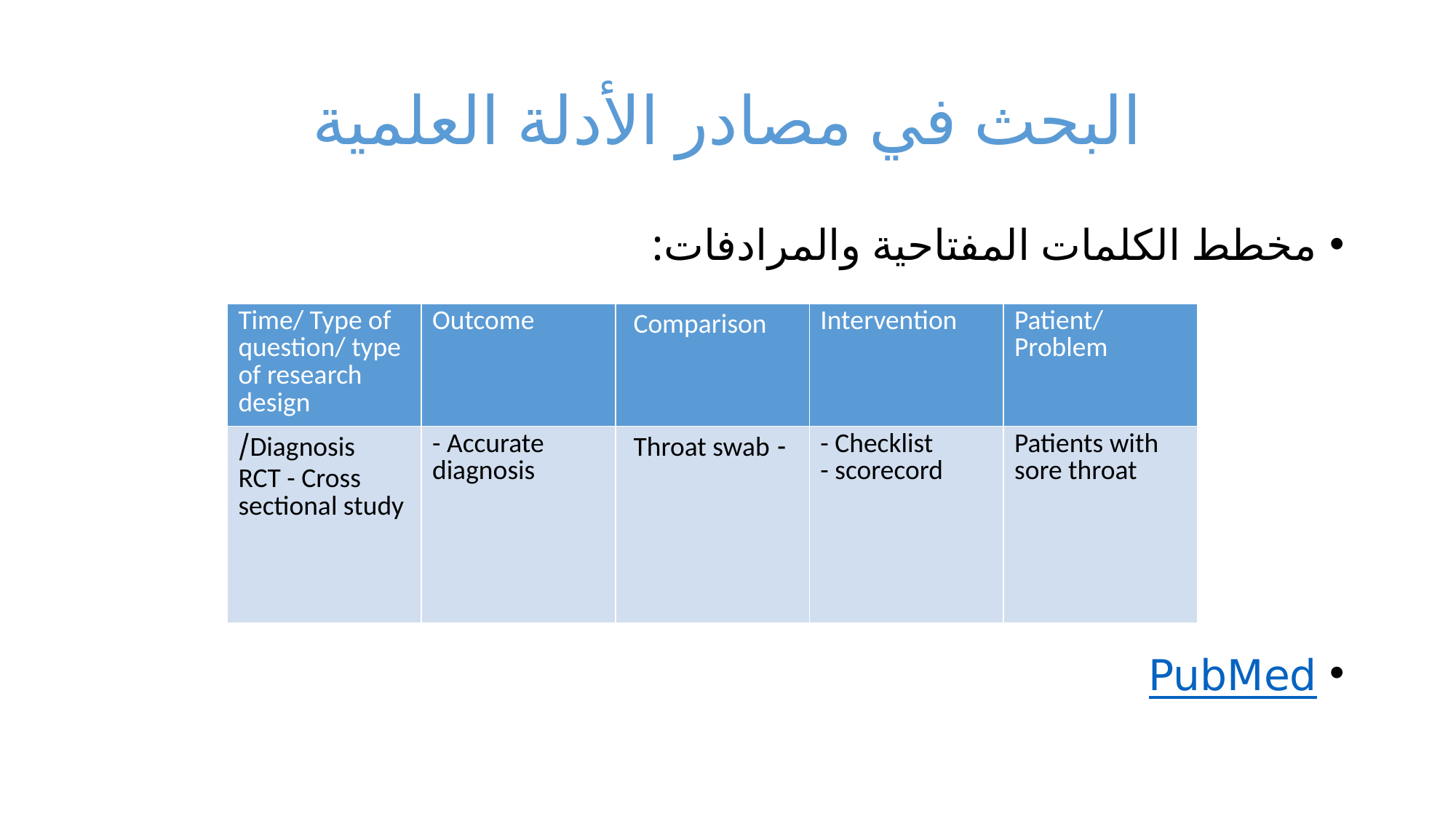

# البحث في مصادر الأدلة العلمية
مخطط الكلمات المفتاحية والمرادفات:
PubMed
| Time/ Type of question/ type of research design | Outcome | Comparison | Intervention | Patient/ Problem |
| --- | --- | --- | --- | --- |
| Diagnosis/ RCT - Cross sectional study | - Accurate diagnosis | - Throat swab | - Checklist - scorecord | Patients with sore throat |
