# Supplementary material for: The First Asynchronous Online Evidence-Based Medicine Course for Syrian Health Workforce: Effectiveness and Feasibility Pilot Study
Source: JMIR Form Res. 2022 Oct 25;6(10):e36782. doi: 10.2196/36782 (PMC9644249; doi:10.2196/36782)
Supplement: Multimedia Appendix 7 [file formative_v6i10e36782_app7.pptx]

## Slide 1
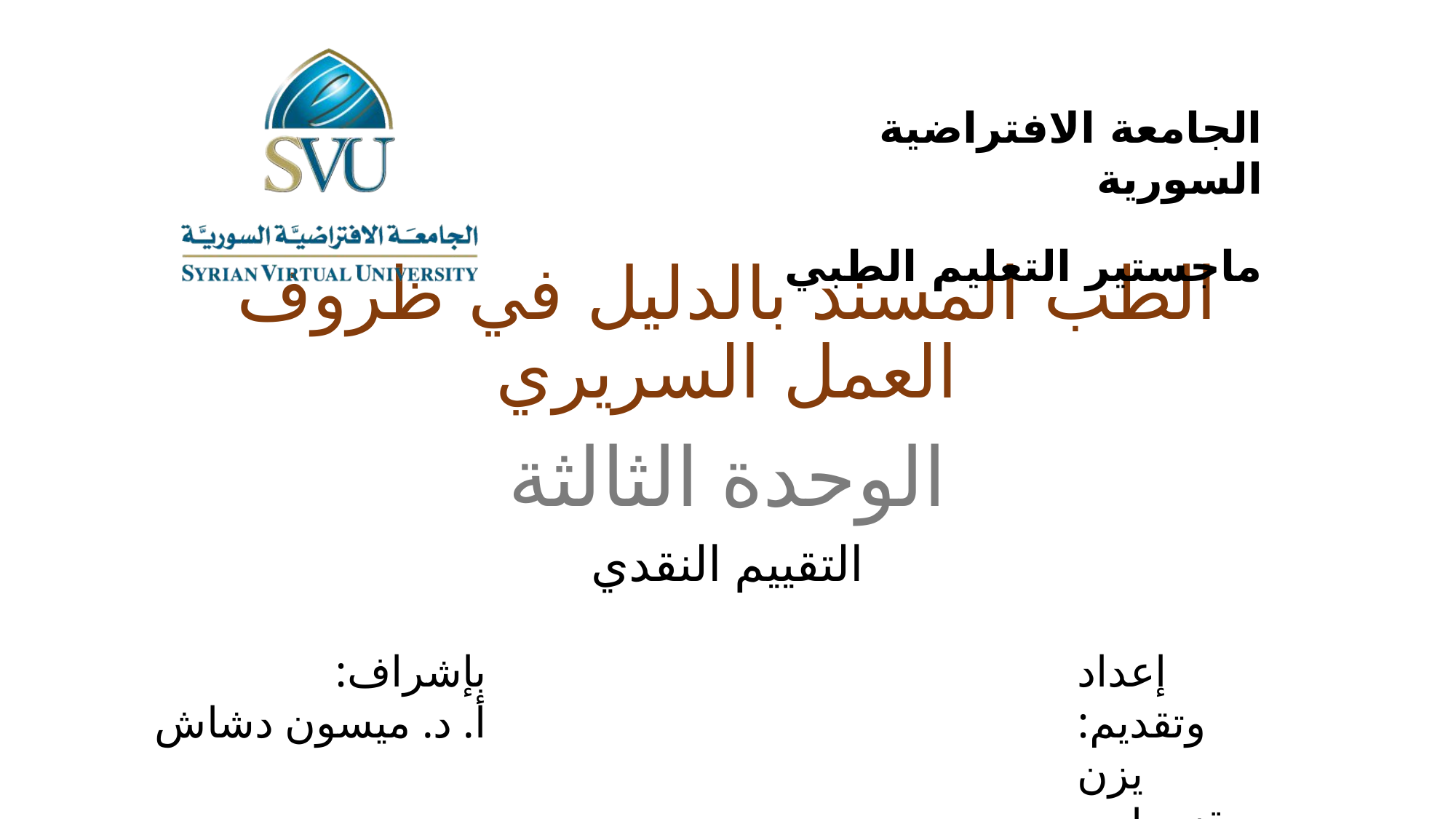

الجامعة الافتراضية السورية
ماجستير التعليم الطبي
# الطب المسند بالدليل في ظروف العمل السريري
الوحدة الثالثة
التقييم النقدي
إعداد وتقديم:
يزن قنجراوي
بإشراف:
أ. د. ميسون دشاش

## Slide 2
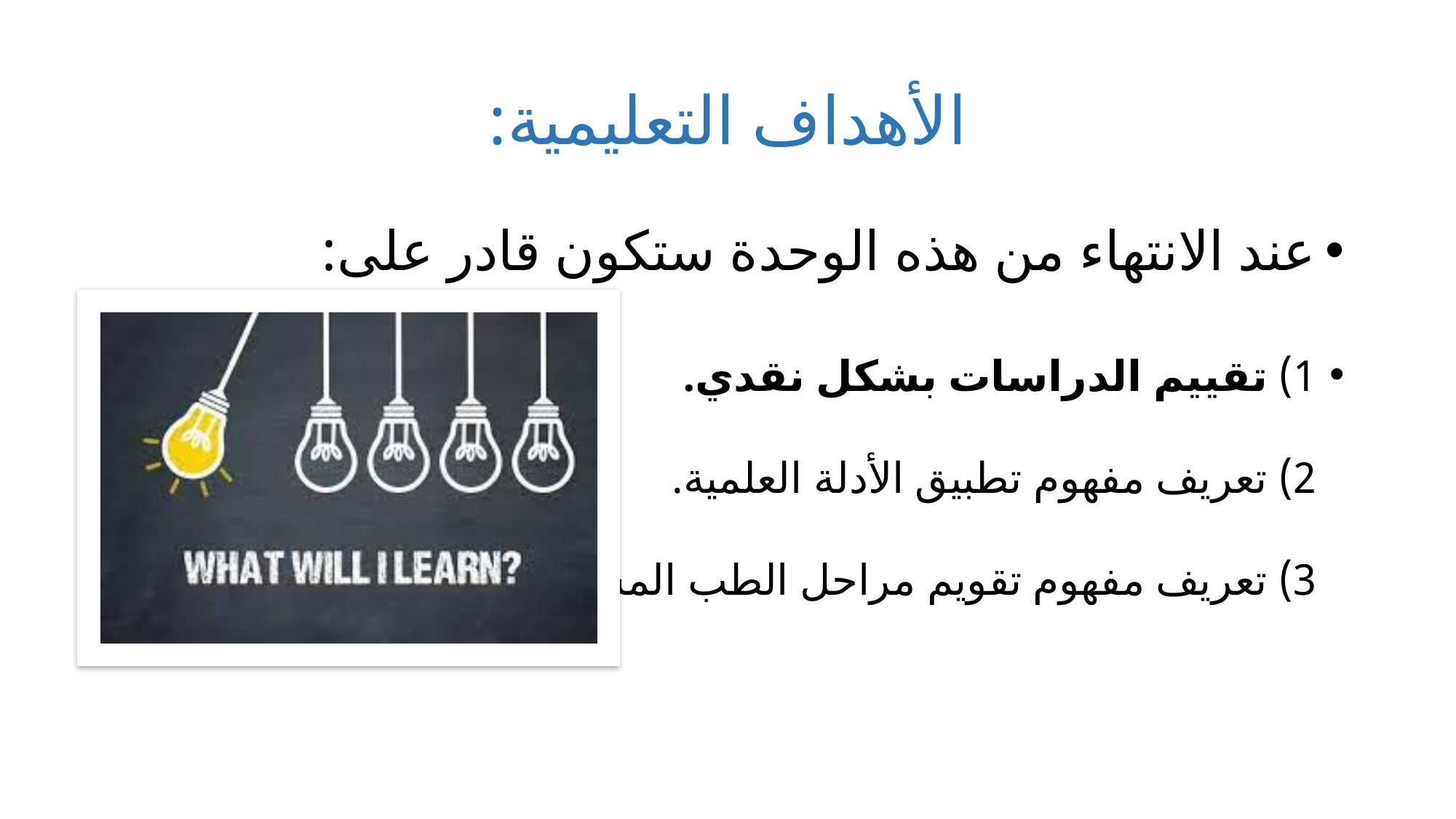

# الأهداف التعليمية:
عند الانتهاء من هذه الوحدة ستكون قادر على:
1) تقييم الدراسات بشكل نقدي.
2) تعريف مفهوم تطبيق الأدلة العلمية.
3) تعريف مفهوم تقويم مراحل الطب المسند بالدليل.

## Slide 3
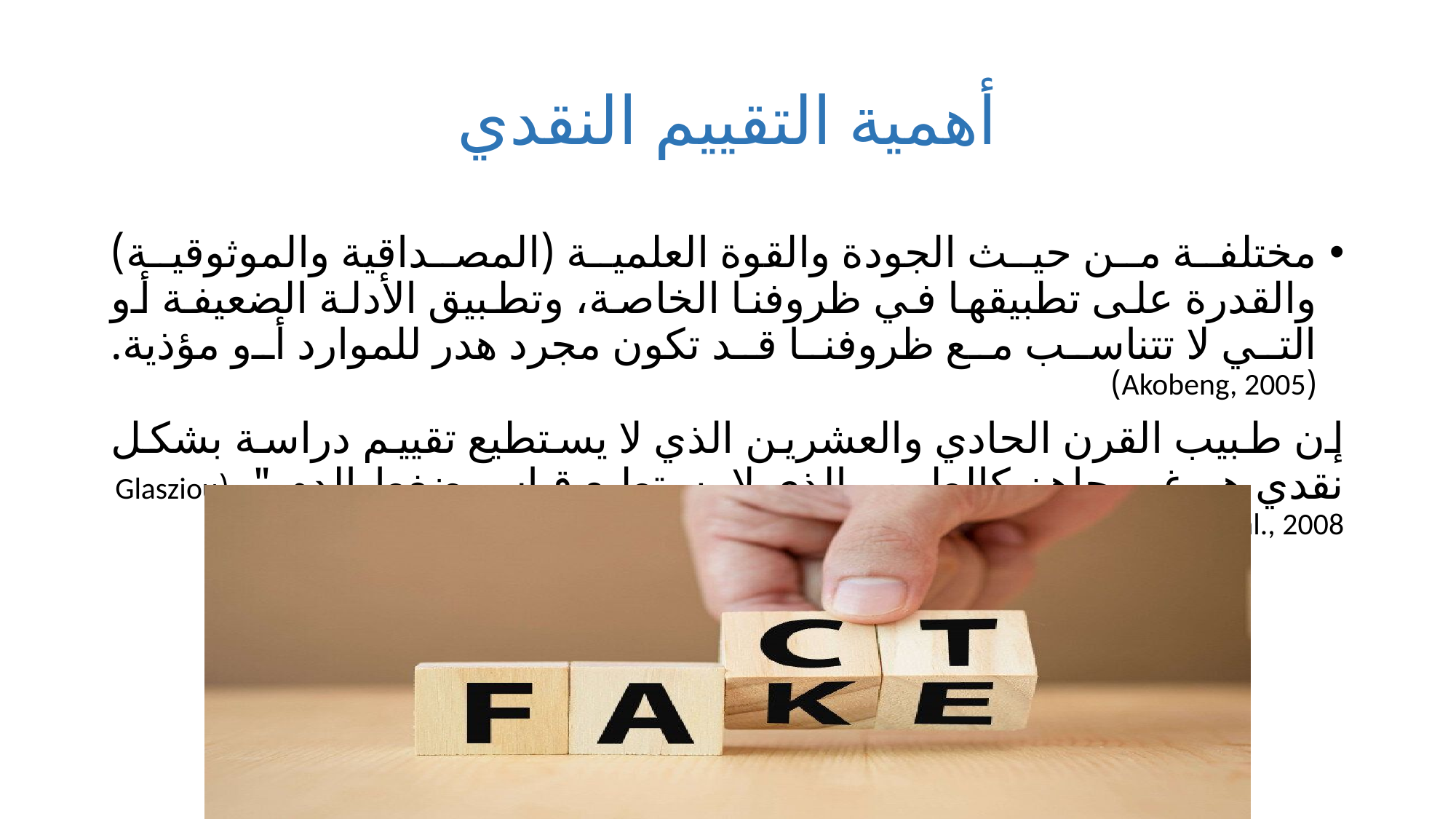

# أهمية التقييم النقدي
مختلفة من حيث الجودة والقوة العلمية (المصداقية والموثوقية) والقدرة على تطبيقها في ظروفنا الخاصة، وتطبيق الأدلة الضعيفة أو التي لا تتناسب مع ظروفنا قد تكون مجرد هدر للموارد أو مؤذية. (Akobeng, 2005)
إن طبيب القرن الحادي والعشرين الذي لا يستطيع تقييم دراسة بشكل نقدي هو غير جاهز كالطبيب الذي لا يستطيع قياس ضغط الدم ". (Glasziou et al., 2008)

## Slide 4
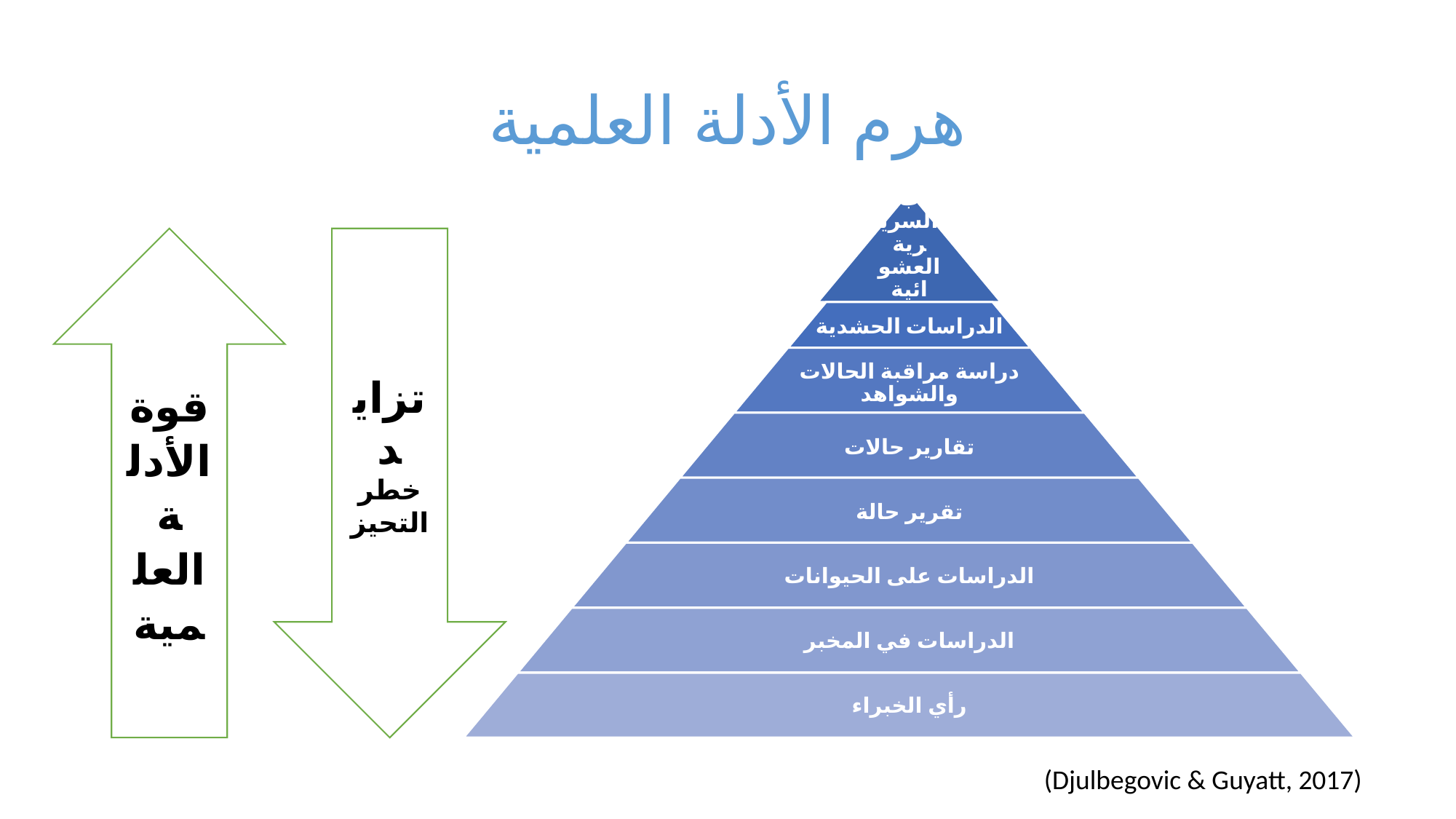

# هرم الأدلة العلمية
قوة الأدلة العلمية
تزايد خطر التحيز
(Djulbegovic & Guyatt, 2017)

## Slide 5
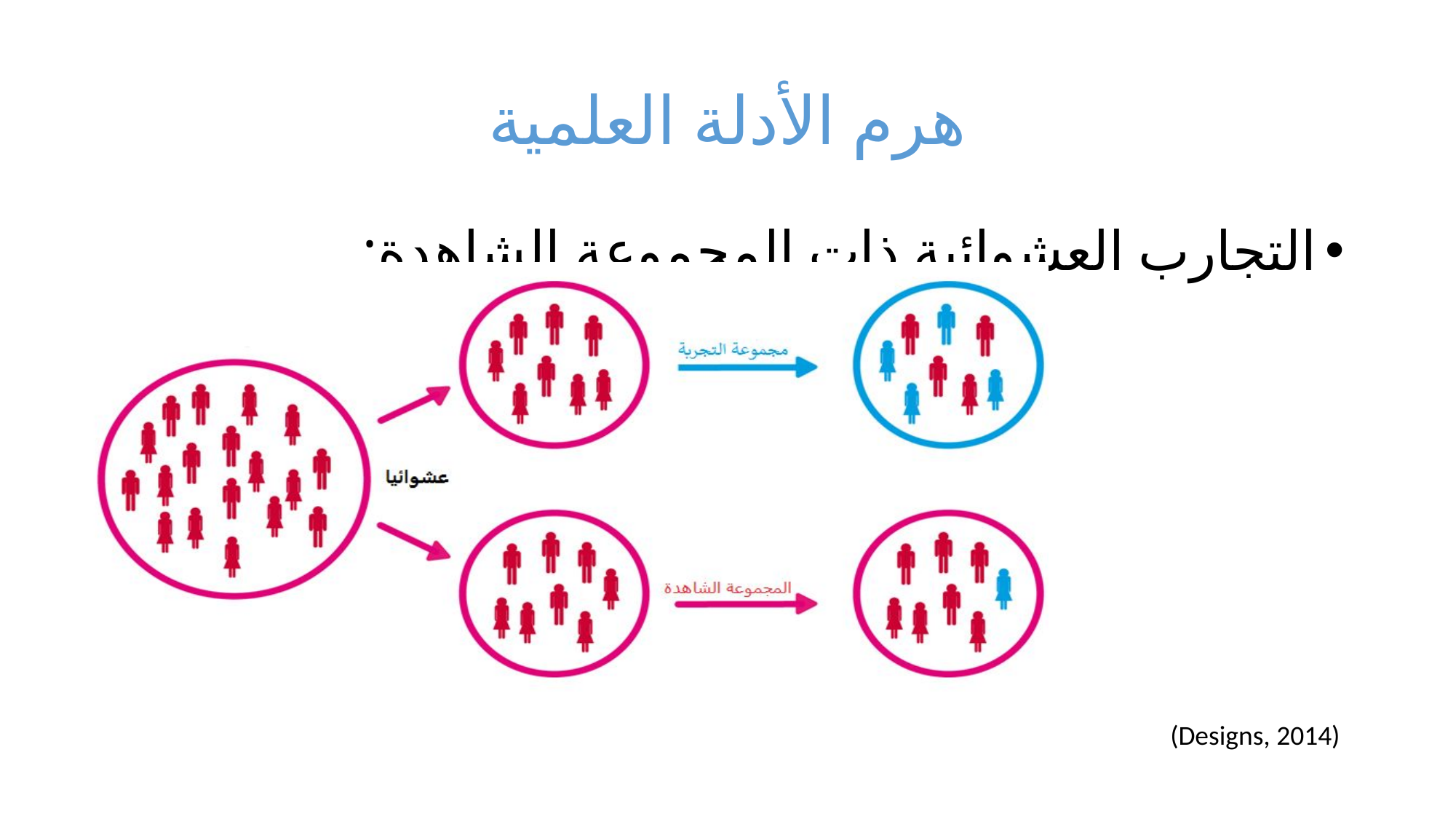

# هرم الأدلة العلمية
التجارب العشوائية ذات المجموعة الشاهدة:
(Designs, 2014)

## Slide 6
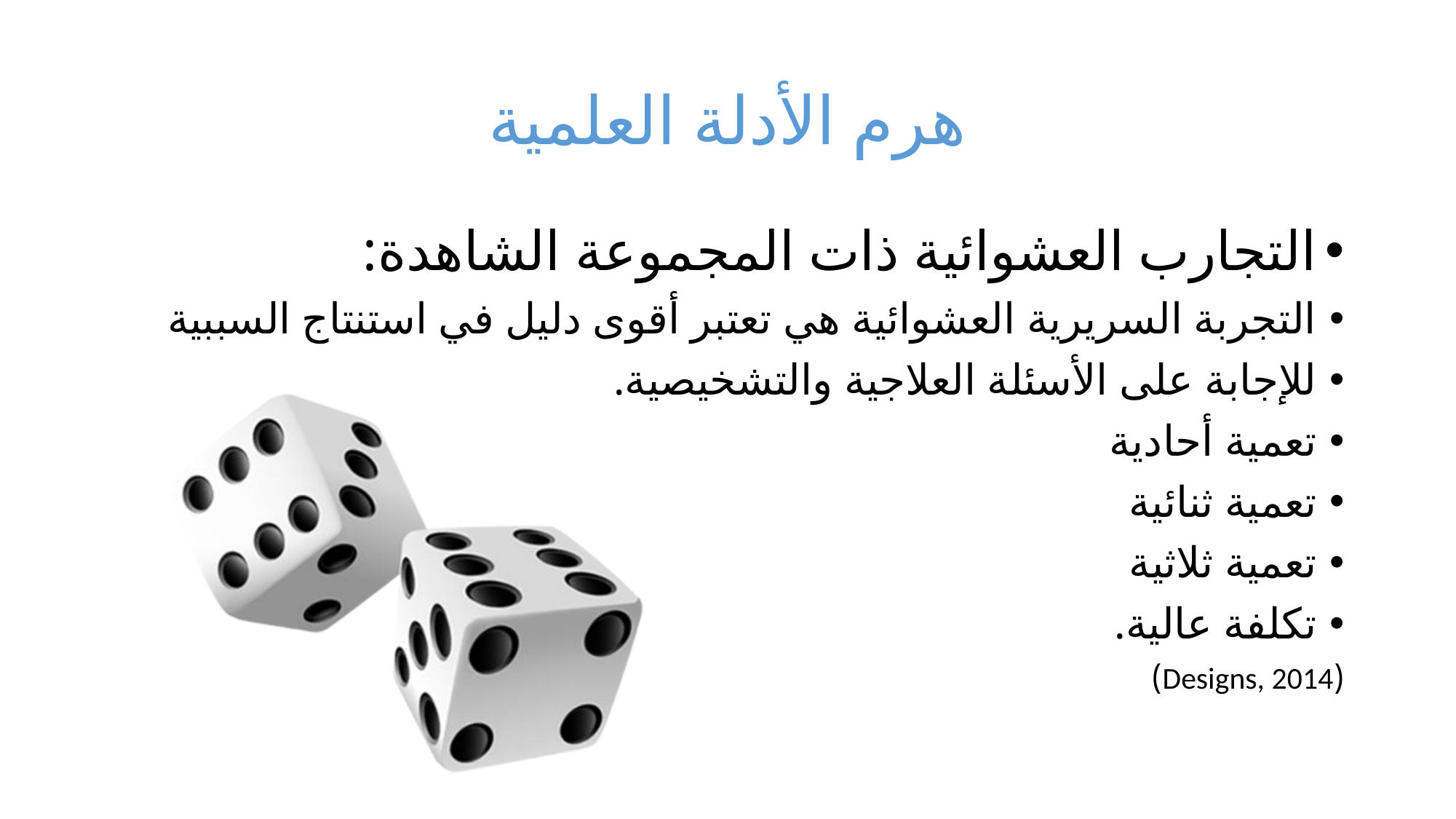

# هرم الأدلة العلمية
التجارب العشوائية ذات المجموعة الشاهدة:
التجربة السريرية العشوائية هي تعتبر أقوى دليل في استنتاج السببية
للإجابة على الأسئلة العلاجية والتشخيصية.
تعمية أحادية
تعمية ثنائية
تعمية ثلاثية
تكلفة عالية.
(Designs, 2014)

## Slide 7
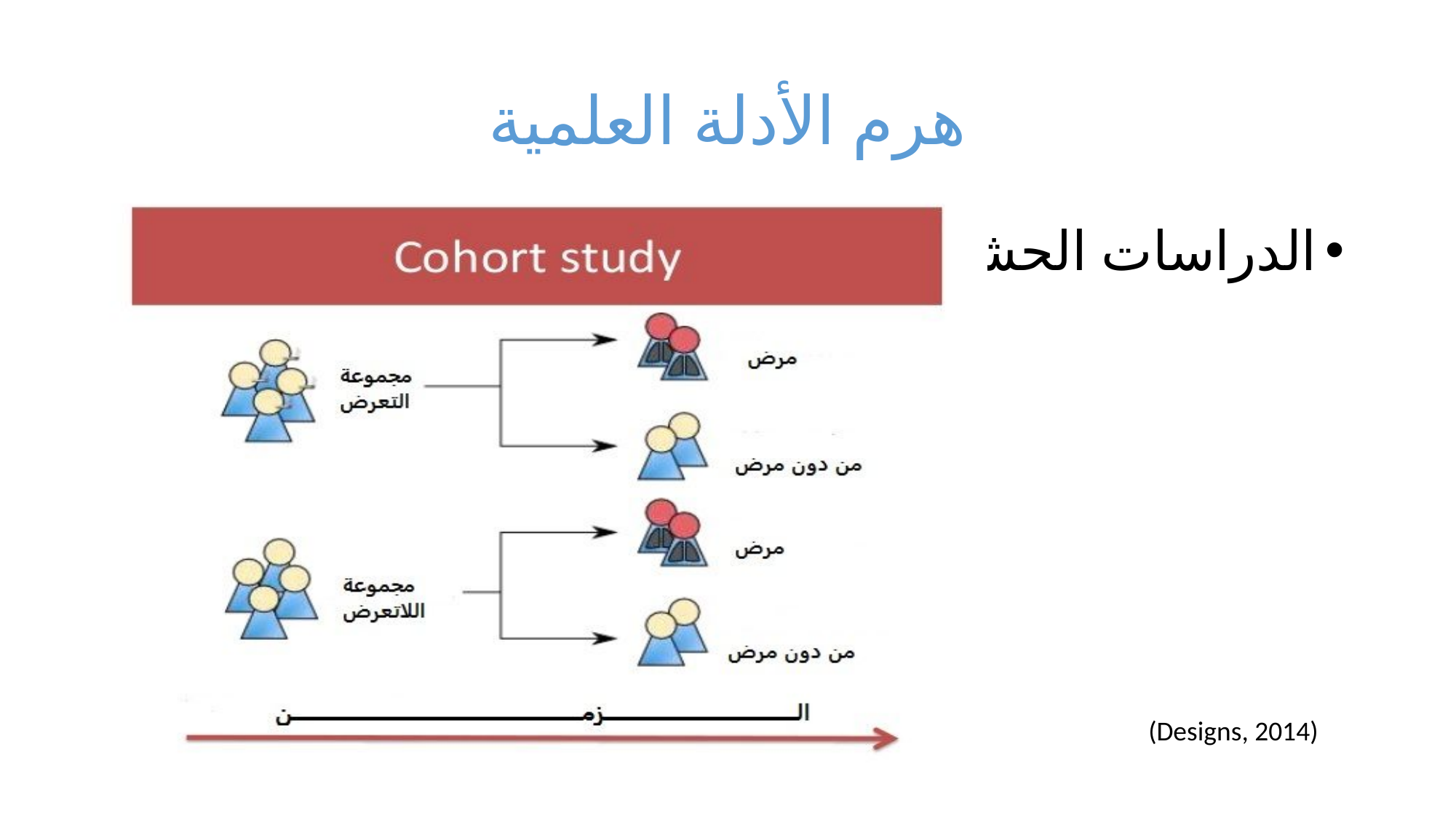

# هرم الأدلة العلمية
الدراسات الحشدية:
(Designs, 2014)

## Slide 8
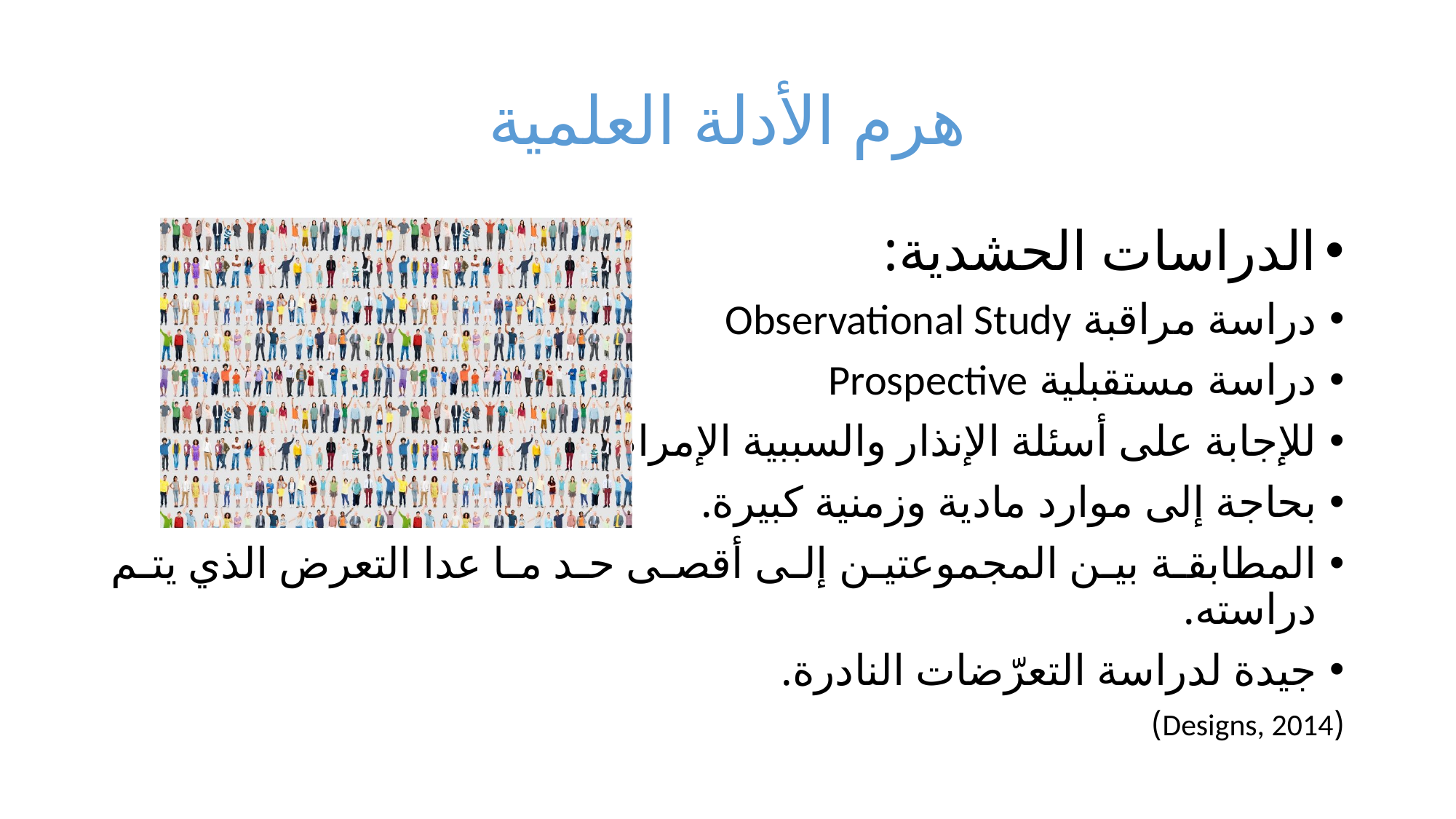

# هرم الأدلة العلمية
الدراسات الحشدية:
دراسة مراقبة Observational Study
دراسة مستقبلية Prospective
للإجابة على أسئلة الإنذار والسببية الإمراضية.
بحاجة إلى موارد مادية وزمنية كبيرة.
المطابقة بين المجموعتين إلى أقصى حد ما عدا التعرض الذي يتم دراسته.
جيدة لدراسة التعرّضات النادرة.
(Designs, 2014)

## Slide 9
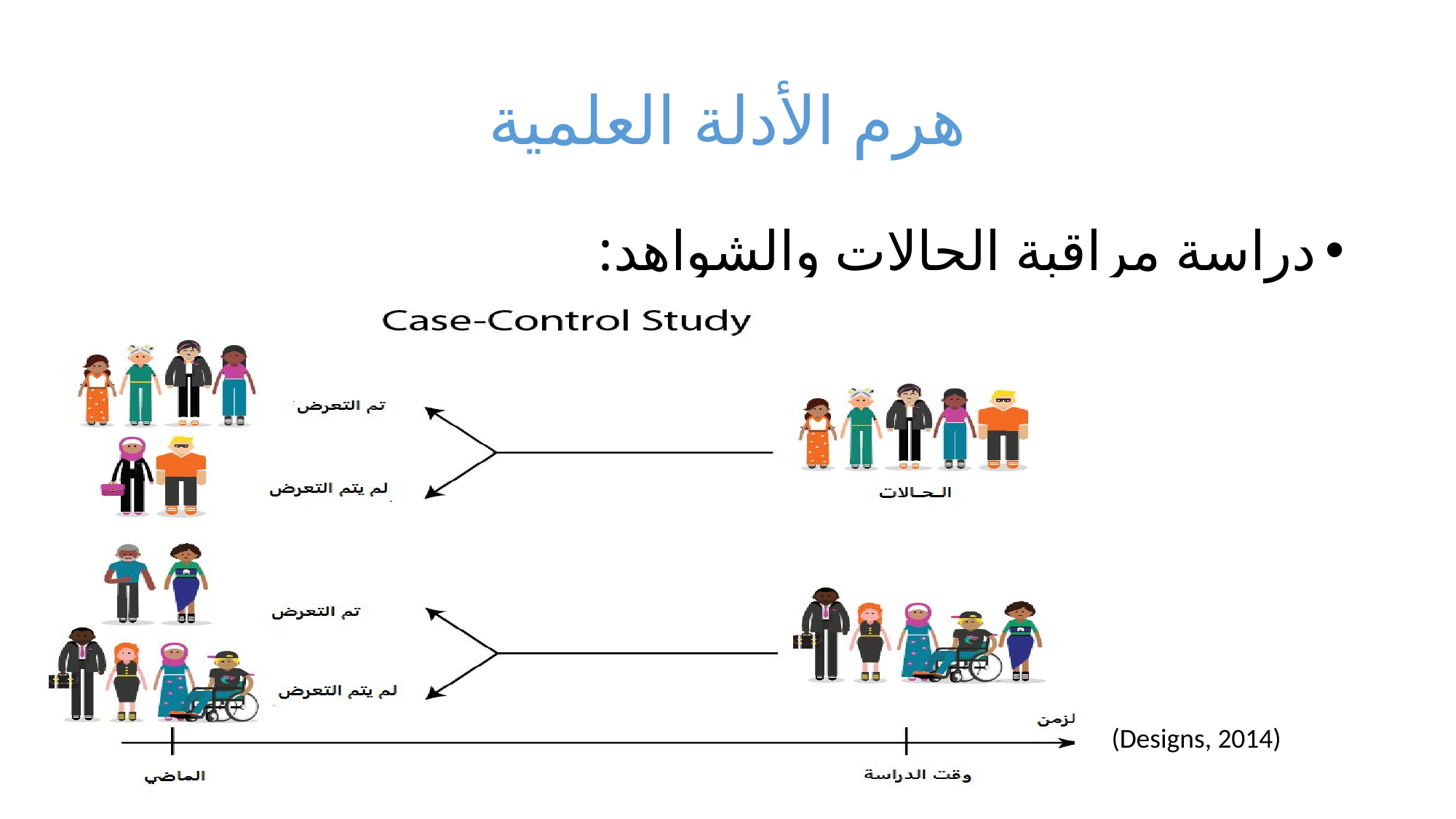

# هرم الأدلة العلمية
دراسة مراقبة الحالات والشواهد:
(Designs, 2014)

## Slide 10
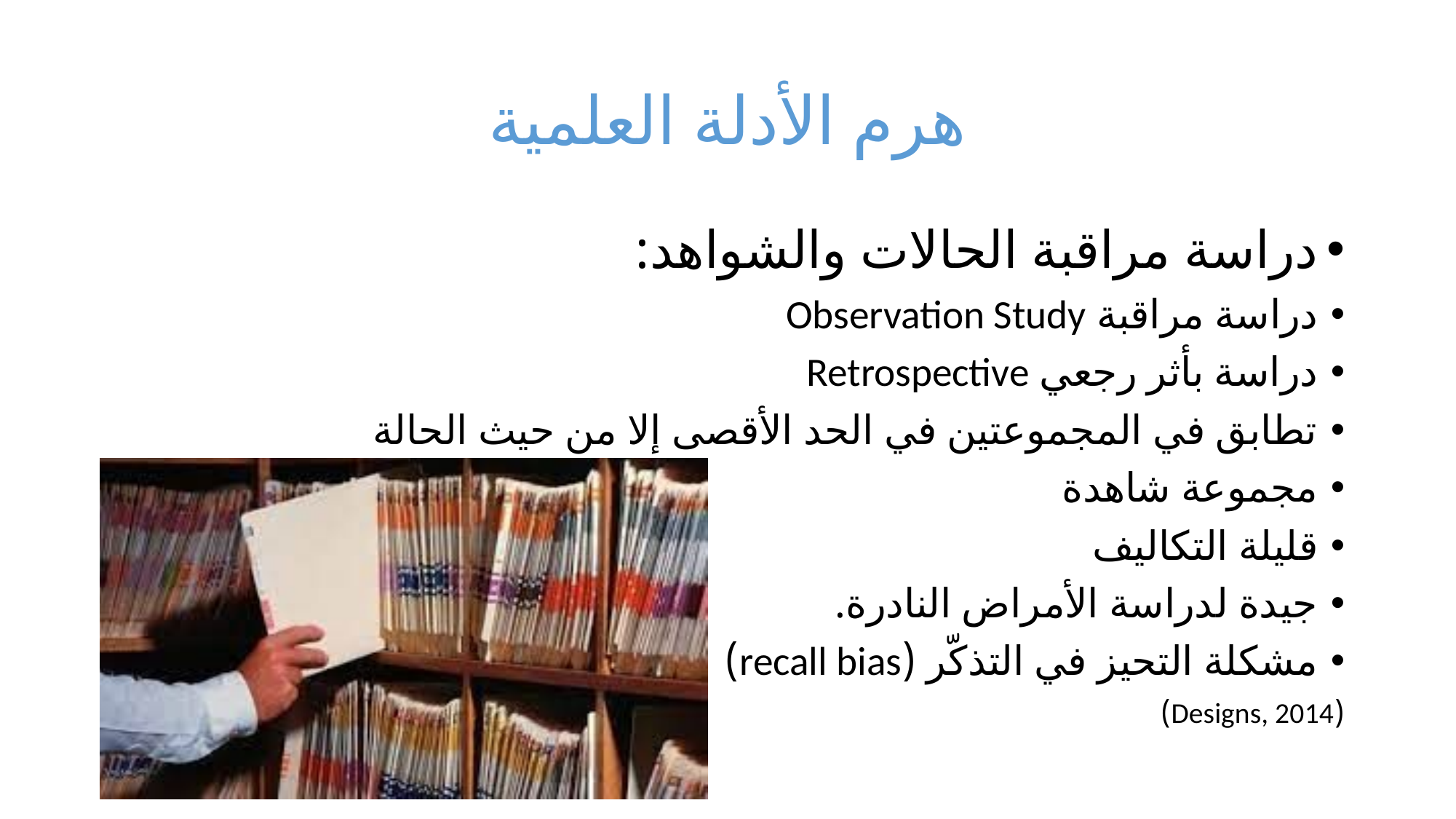

# هرم الأدلة العلمية
دراسة مراقبة الحالات والشواهد:
دراسة مراقبة Observation Study
دراسة بأثر رجعي Retrospective
تطابق في المجموعتين في الحد الأقصى إلا من حيث الحالة
مجموعة شاهدة
قليلة التكاليف
جيدة لدراسة الأمراض النادرة.
مشكلة التحيز في التذكّر (recall bias)
(Designs, 2014)

## Slide 11
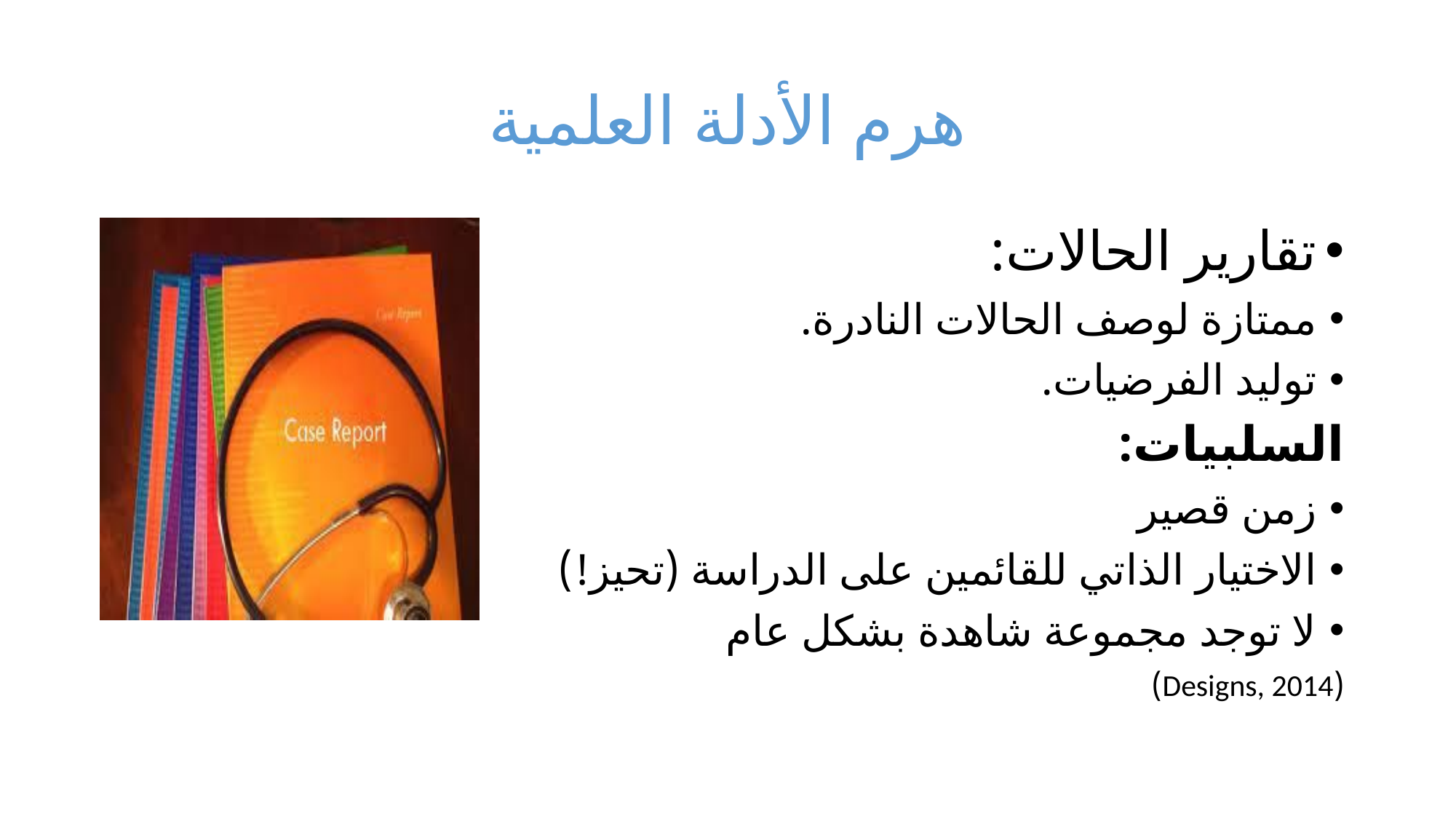

# هرم الأدلة العلمية
تقارير الحالات:
ممتازة لوصف الحالات النادرة.
توليد الفرضيات.
السلبيات:
زمن قصير
الاختيار الذاتي للقائمين على الدراسة (تحيز!)
لا توجد مجموعة شاهدة بشكل عام
(Designs, 2014)

## Slide 12
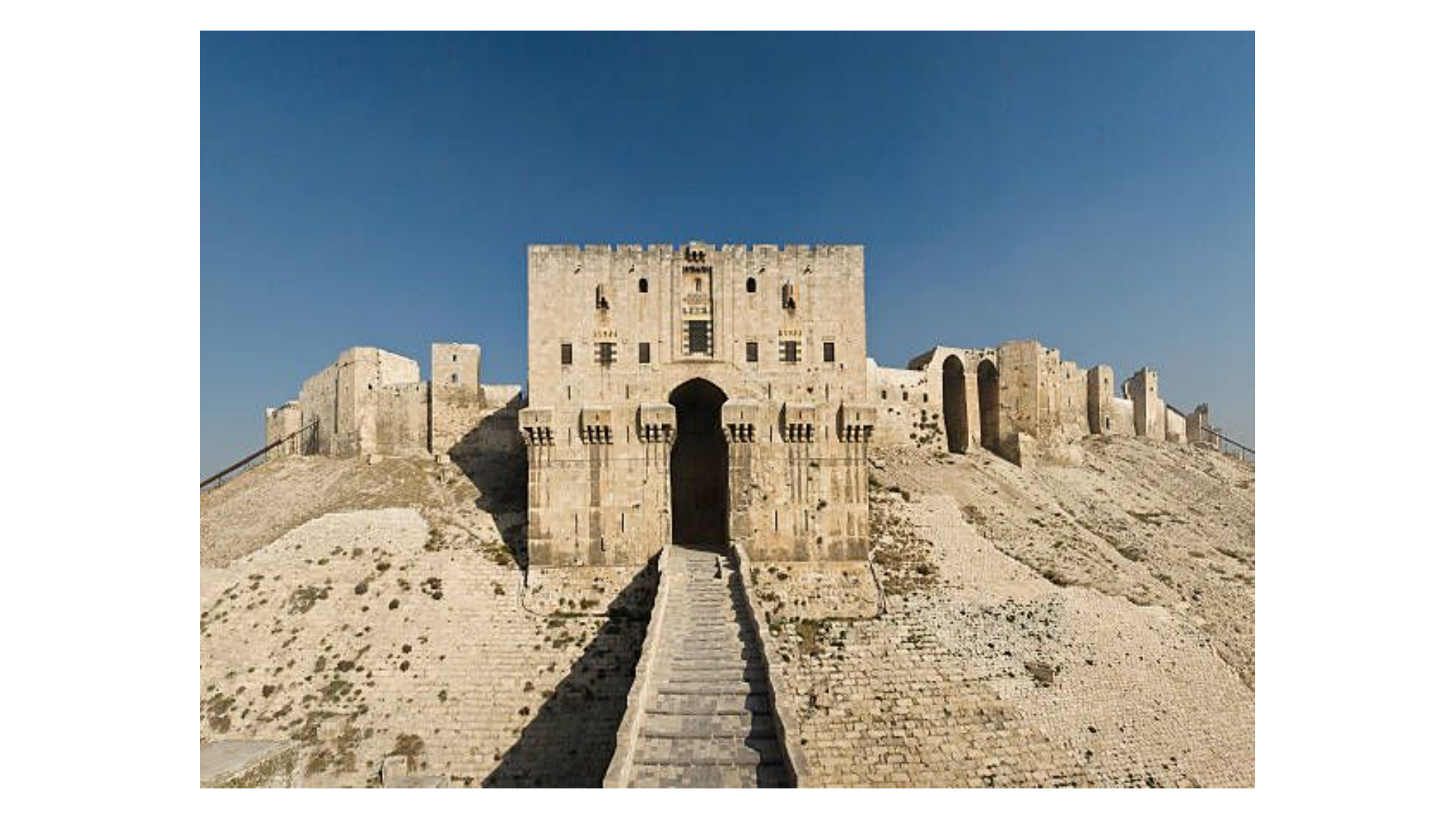

## Slide 13
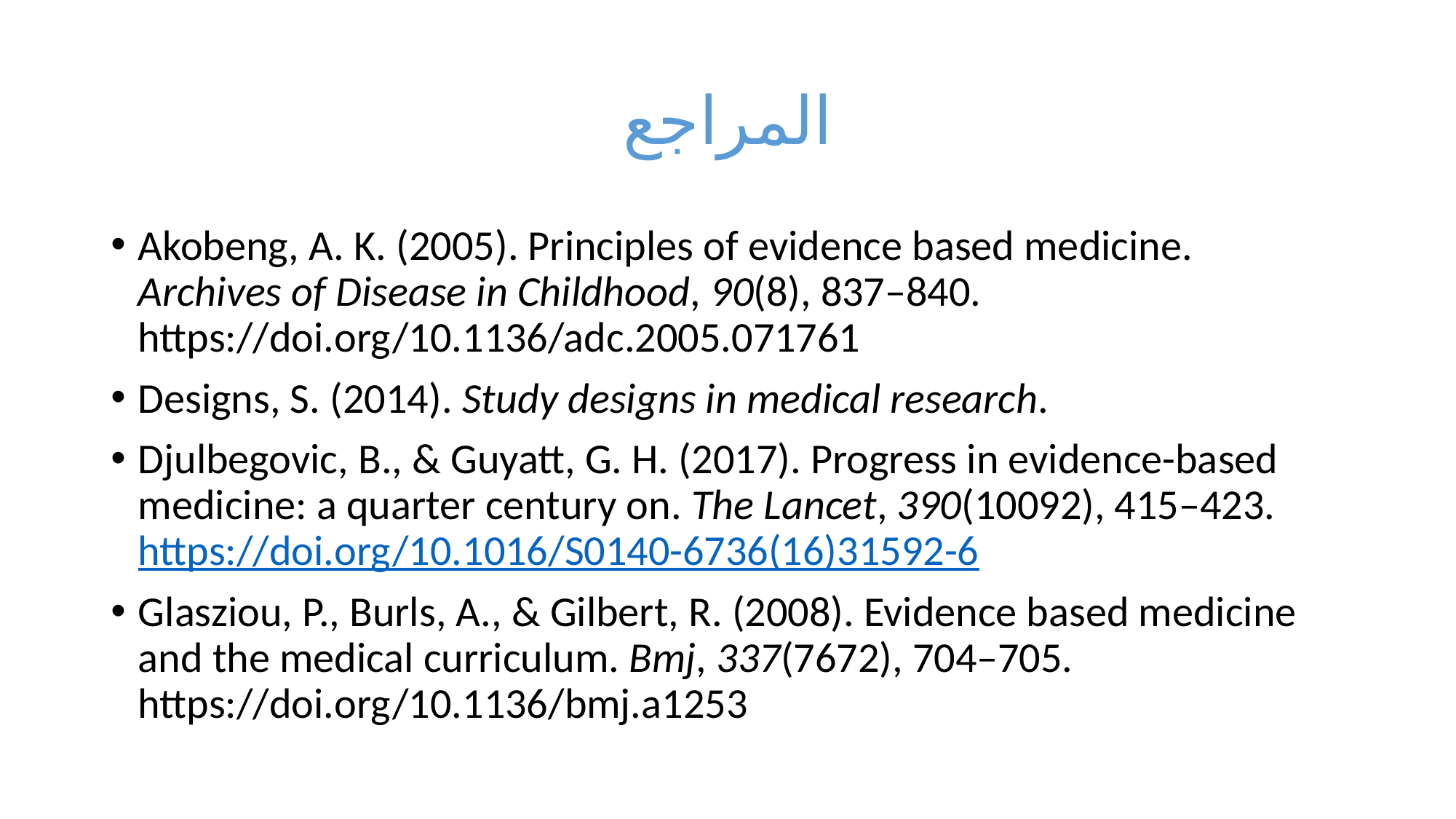

# المراجع
Akobeng, A. K. (2005). Principles of evidence based medicine. Archives of Disease in Childhood, 90(8), 837–840. https://doi.org/10.1136/adc.2005.071761
Designs, S. (2014). Study designs in medical research.
Djulbegovic, B., & Guyatt, G. H. (2017). Progress in evidence-based medicine: a quarter century on. The Lancet, 390(10092), 415–423. https://doi.org/10.1016/S0140-6736(16)31592-6
Glasziou, P., Burls, A., & Gilbert, R. (2008). Evidence based medicine and the medical curriculum. Bmj, 337(7672), 704–705. https://doi.org/10.1136/bmj.a1253
