# Supplementary material for: The First Asynchronous Online Evidence-Based Medicine Course for Syrian Health Workforce: Effectiveness and Feasibility Pilot Study
Source: JMIR Form Res. 2022 Oct 25;6(10):e36782. doi: 10.2196/36782 (PMC9644249; doi:10.2196/36782)
Supplement: Multimedia Appendix 8 [file formative_v6i10e36782_app8.pptx]

## Slide 1
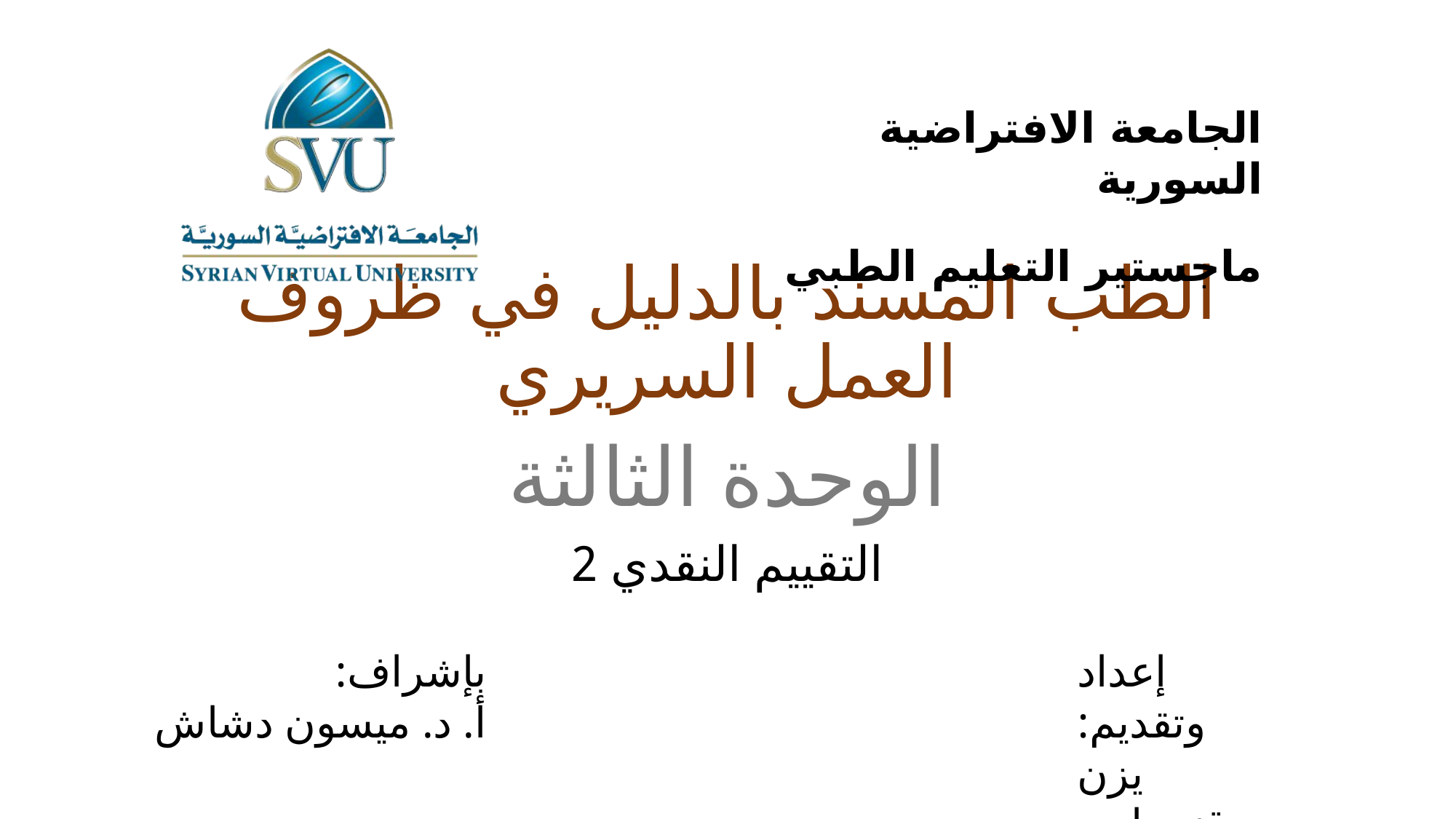

الجامعة الافتراضية السورية
ماجستير التعليم الطبي
# الطب المسند بالدليل في ظروف العمل السريري
الوحدة الثالثة
التقييم النقدي 2
إعداد وتقديم:
يزن قنجراوي
بإشراف:
أ. د. ميسون دشاش

## Slide 2
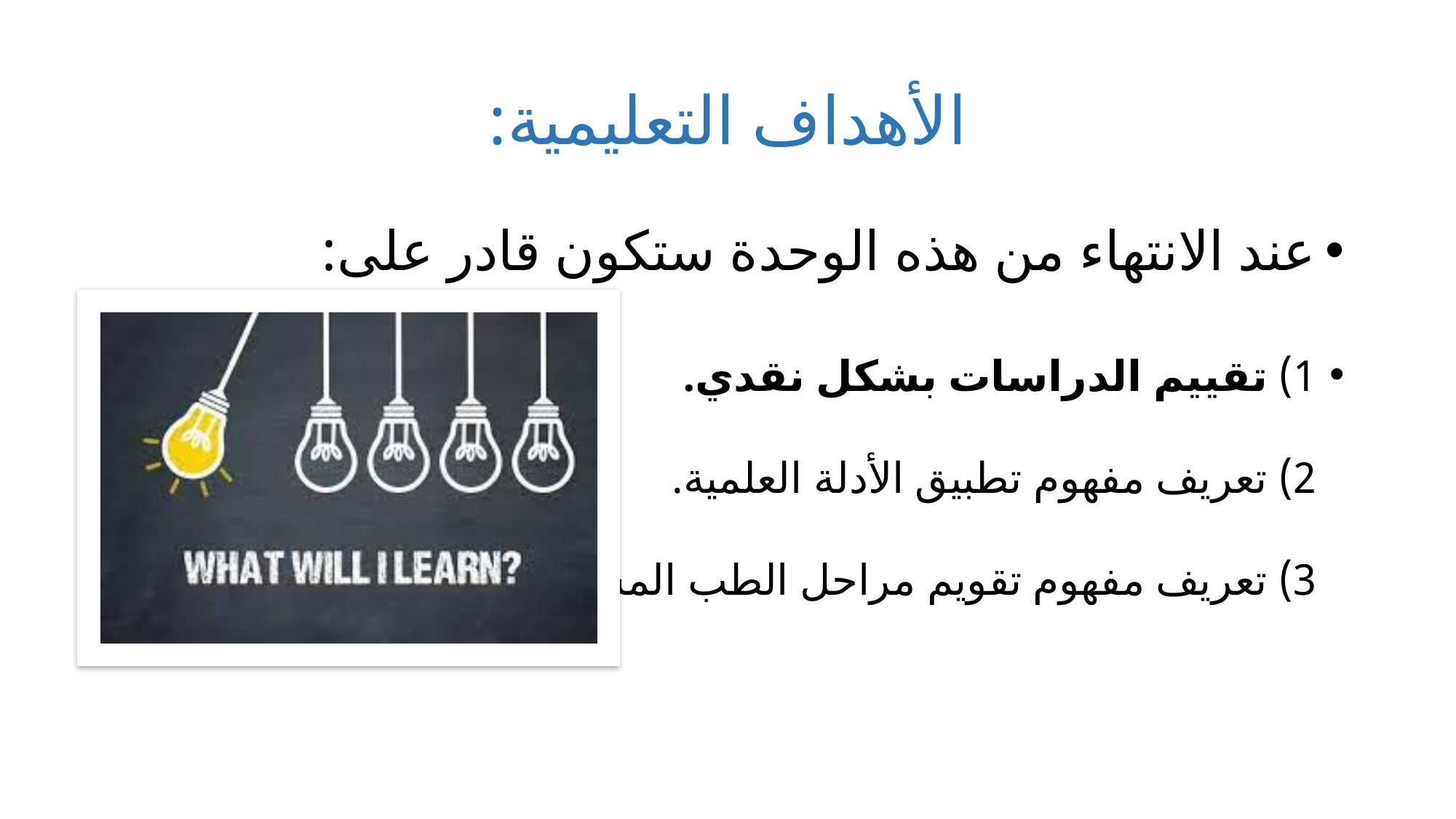

# الأهداف التعليمية:
عند الانتهاء من هذه الوحدة ستكون قادر على:
1) تقييم الدراسات بشكل نقدي.
2) تعريف مفهوم تطبيق الأدلة العلمية.
3) تعريف مفهوم تقويم مراحل الطب المسند بالدليل.

## Slide 3
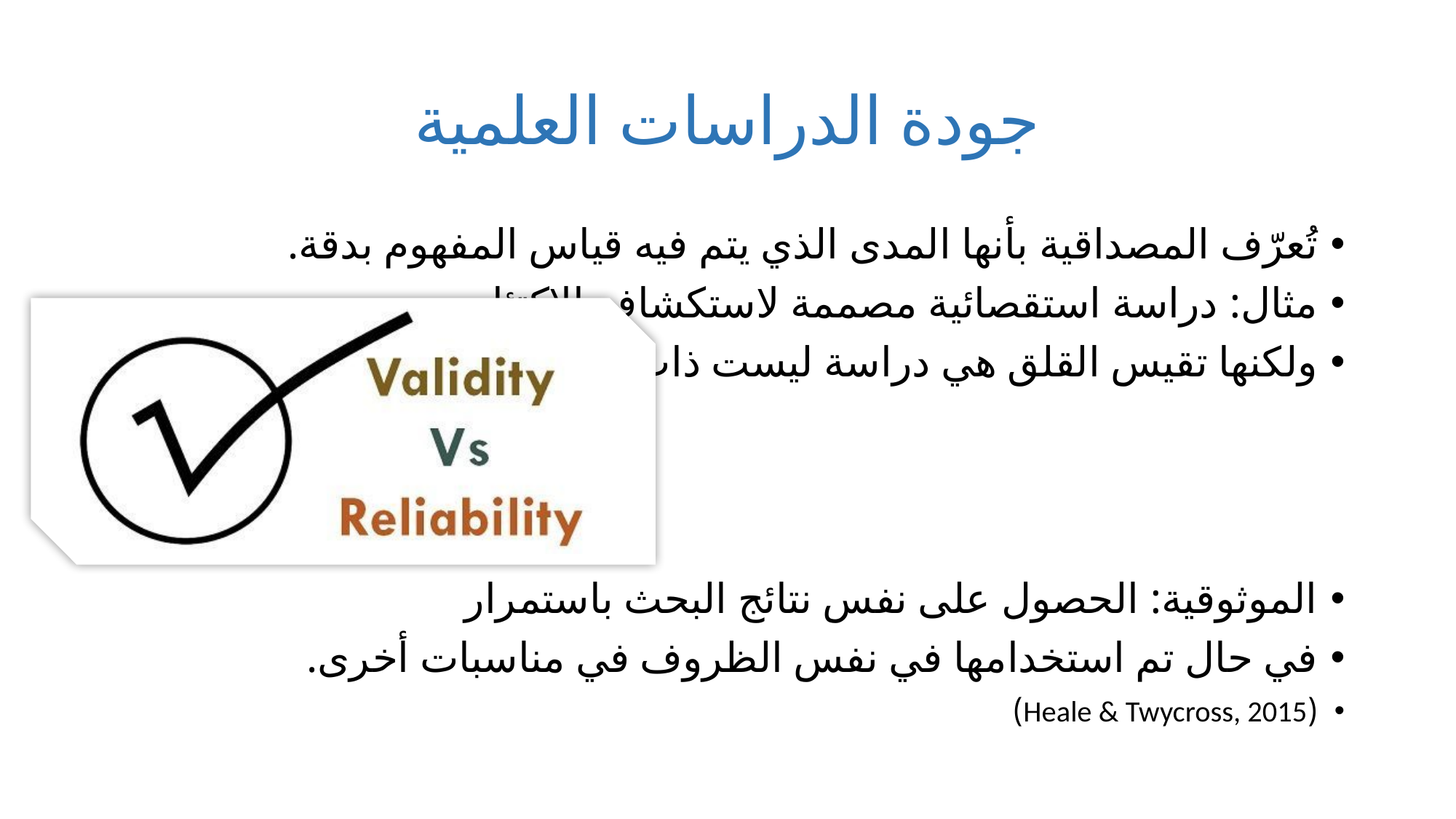

# جودة الدراسات العلمية
تُعرّف المصداقية بأنها المدى الذي يتم فيه قياس المفهوم بدقة.
مثال: دراسة استقصائية مصممة لاستكشاف الاكتئاب
ولكنها تقيس القلق هي دراسة ليست ذات مصداقية.
الموثوقية: الحصول على نفس نتائج البحث باستمرار
في حال تم استخدامها في نفس الظروف في مناسبات أخرى.
(Heale & Twycross, 2015)

## Slide 4
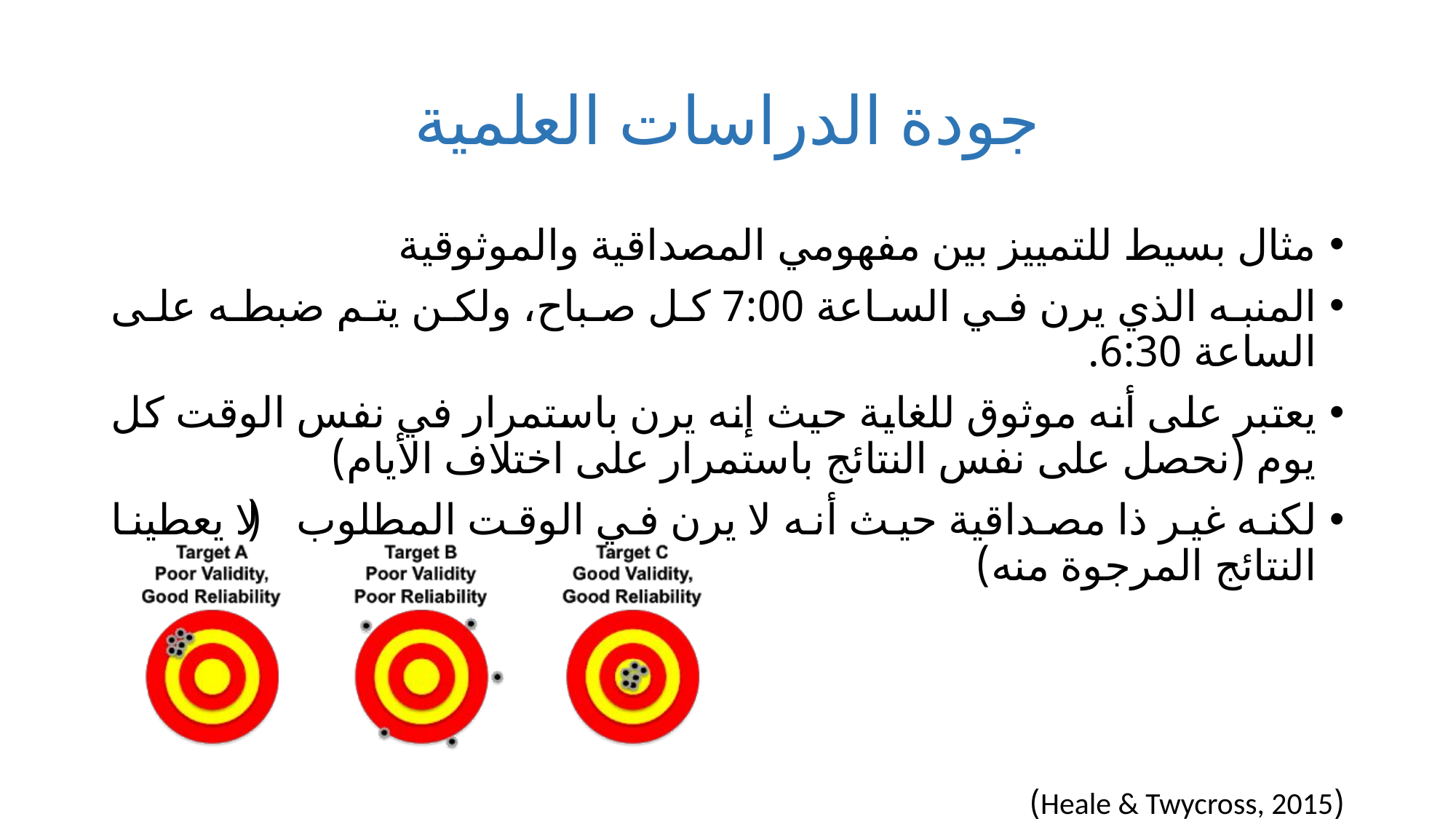

# جودة الدراسات العلمية
مثال بسيط للتمييز بين مفهومي المصداقية والموثوقية
المنبه الذي يرن في الساعة 7:00 كل صباح، ولكن يتم ضبطه على الساعة 6:30.
يعتبر على أنه موثوق للغاية حيث إنه يرن باستمرار في نفس الوقت كل يوم (نحصل على نفس النتائج باستمرار على اختلاف الأيام)
لكنه غير ذا مصداقية حيث أنه لا يرن في الوقت المطلوب ( لا يعطينا النتائج المرجوة منه)
(Heale & Twycross, 2015)

## Slide 5
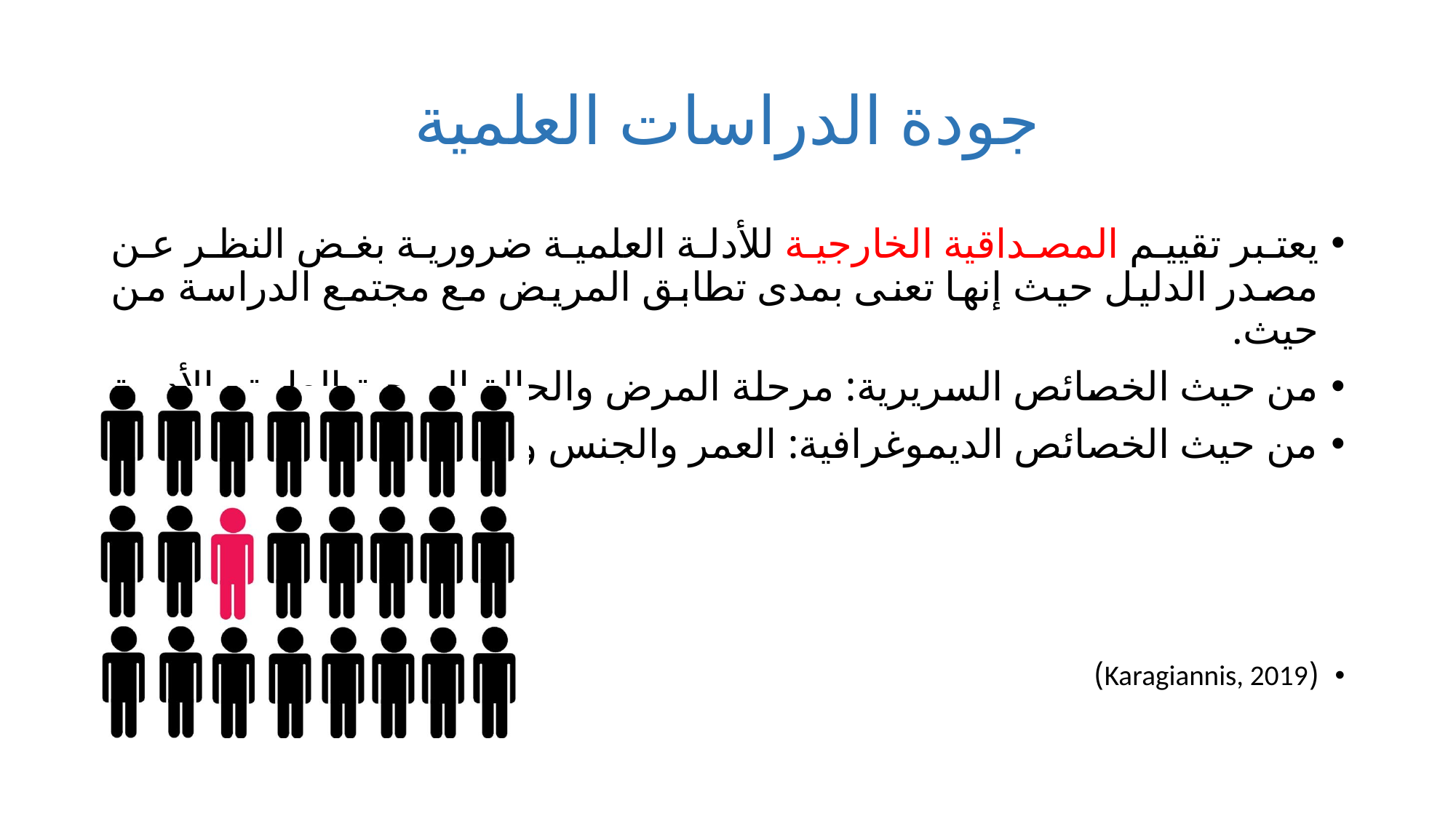

# جودة الدراسات العلمية
يعتبر تقييم المصداقية الخارجية للأدلة العلمية ضرورية بغض النظر عن مصدر الدليل حيث إنها تعنى بمدى تطابق المريض مع مجتمع الدراسة من حيث.
من حيث الخصائص السريرية: مرحلة المرض والحالة الصحية العامة والأدوية
من حيث الخصائص الديموغرافية: العمر والجنس والعرق
(Karagiannis, 2019)

## Slide 6
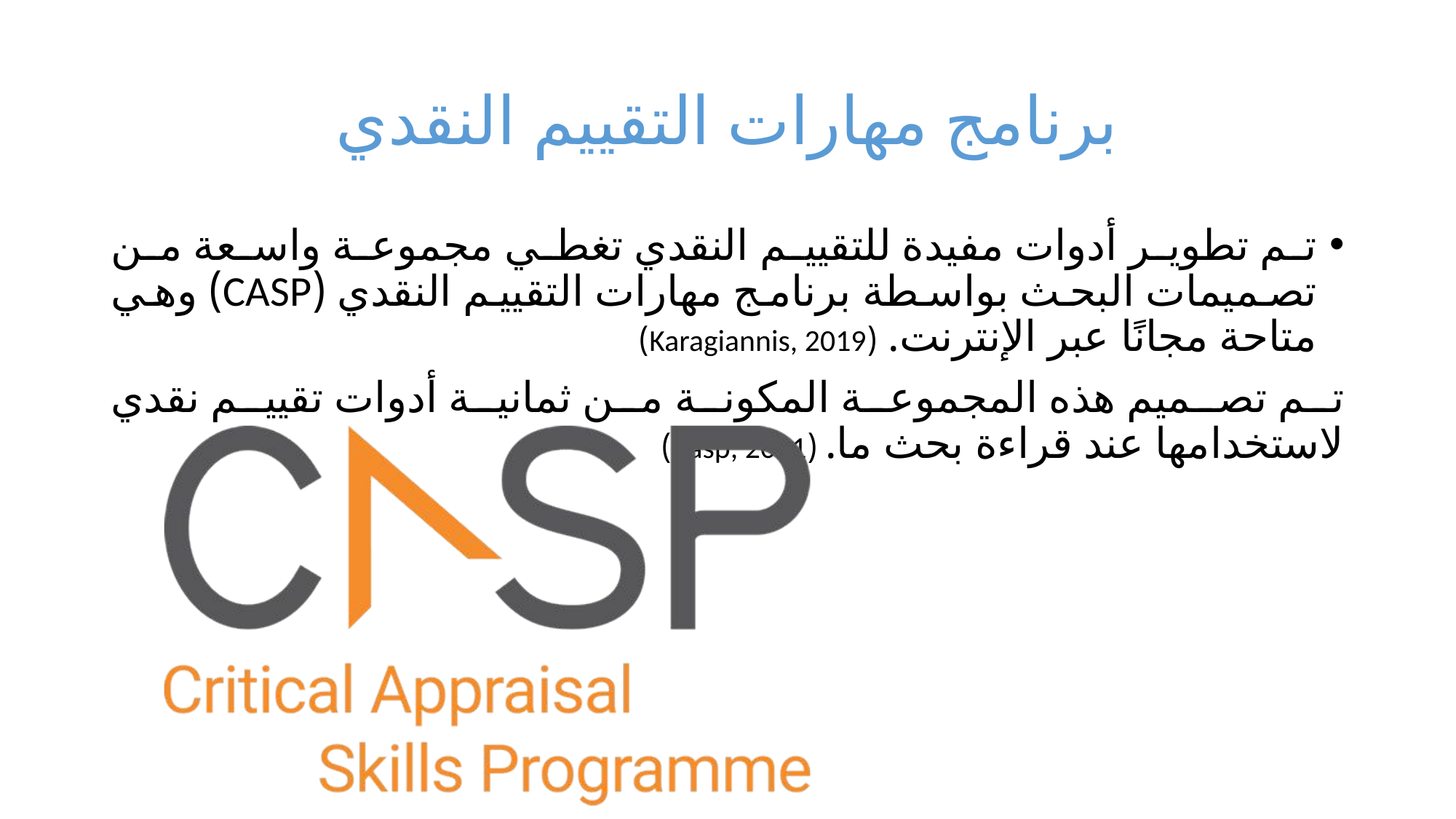

# برنامج مهارات التقييم النقدي
تم تطوير أدوات مفيدة للتقييم النقدي تغطي مجموعة واسعة من تصميمات البحث بواسطة برنامج مهارات التقييم النقدي (CASP) وهي متاحة مجانًا عبر الإنترنت. (Karagiannis, 2019)
تم تصميم هذه المجموعة المكونة من ثمانية أدوات تقييم نقدي لاستخدامها عند قراءة بحث ما. (Casp, 2021)

## Slide 7
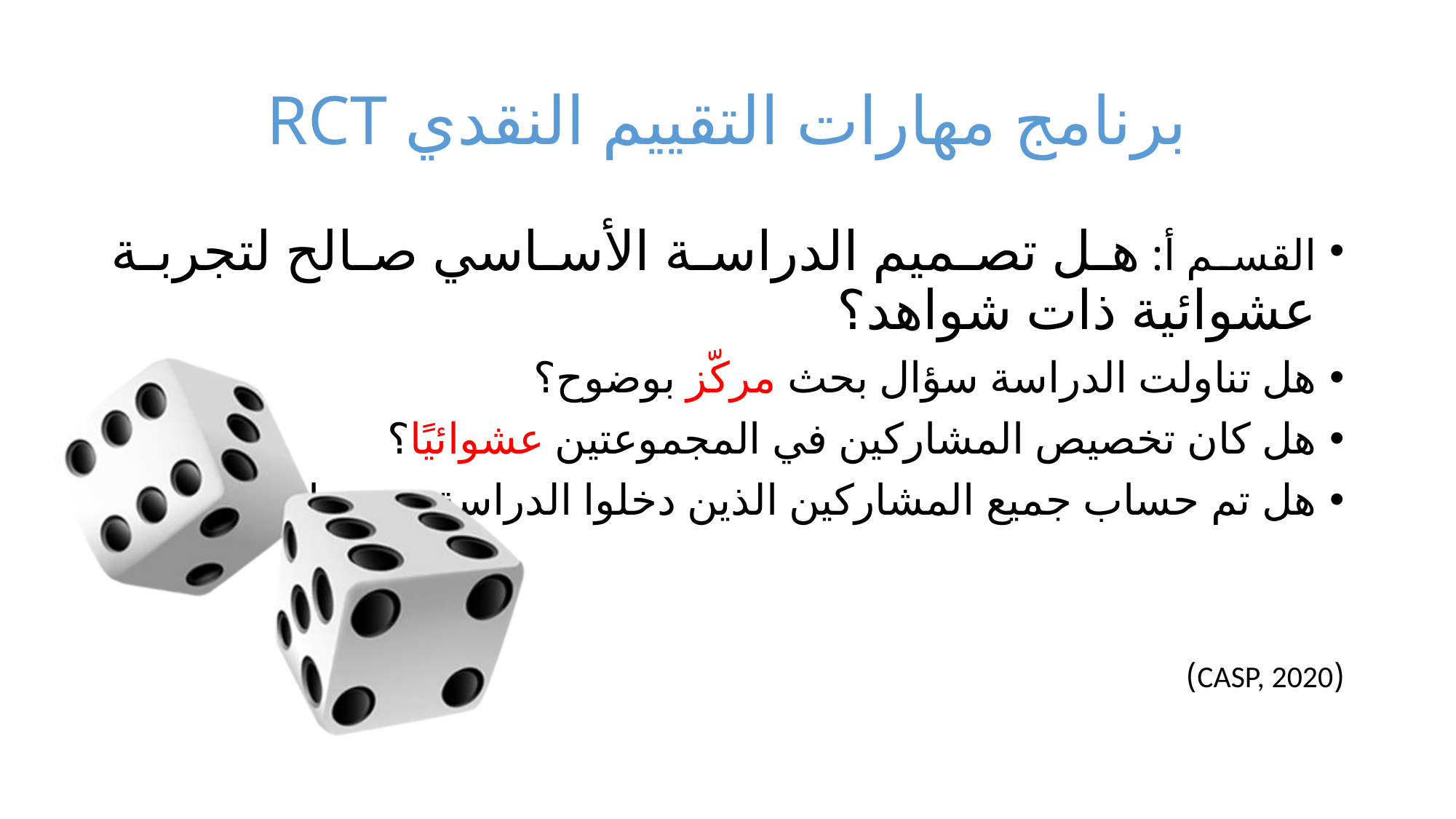

# برنامج مهارات التقييم النقدي RCT
القسم أ: هل تصميم الدراسة الأساسي صالح لتجربة عشوائية ذات شواهد؟
هل تناولت الدراسة سؤال بحث مركّز بوضوح؟
هل كان تخصيص المشاركين في المجموعتين عشوائيًا؟
هل تم حساب جميع المشاركين الذين دخلوا الدراسة عند نهايتها؟
(CASP, 2020)

## Slide 8
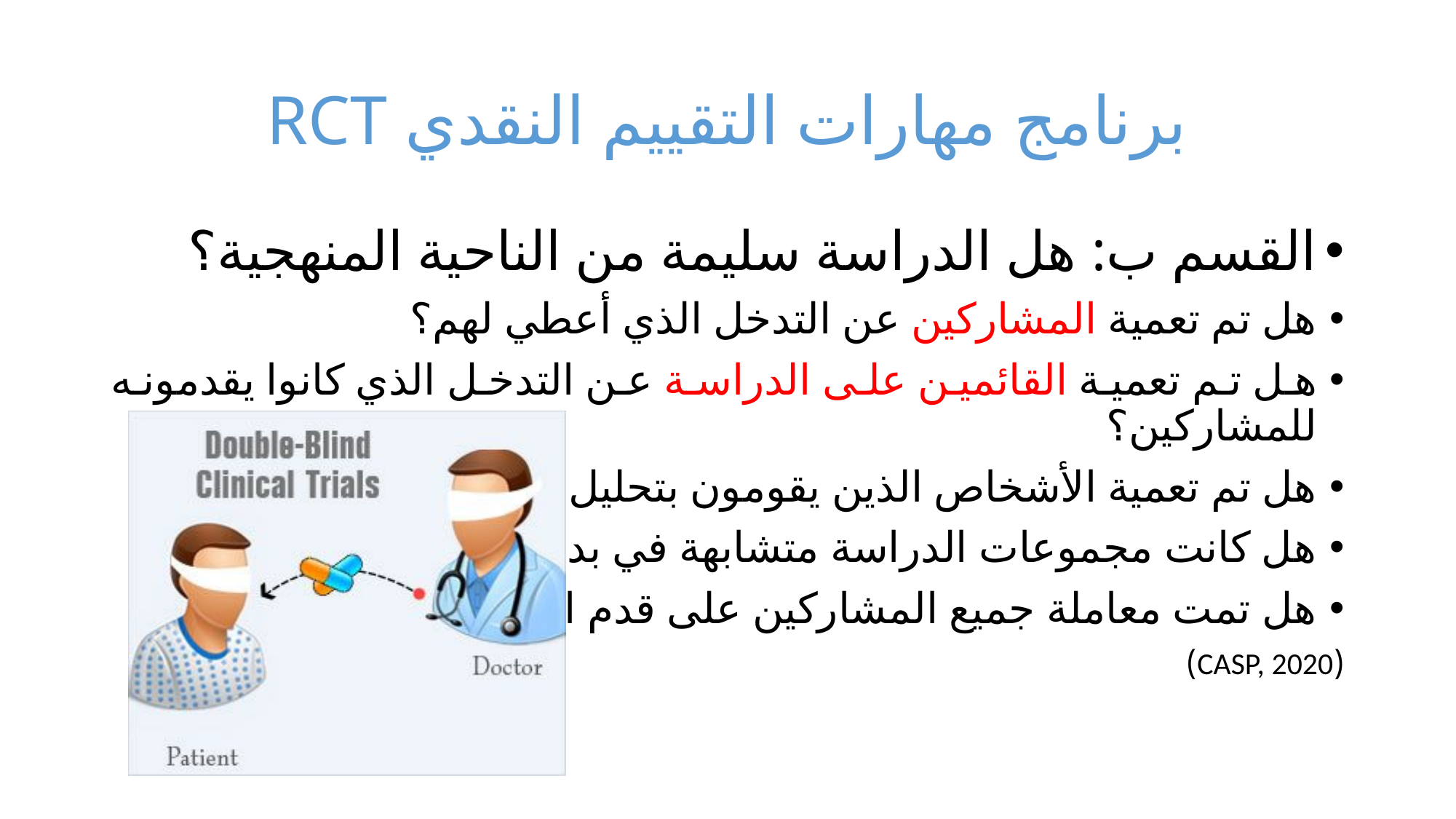

# برنامج مهارات التقييم النقدي RCT
القسم ب: هل الدراسة سليمة من الناحية المنهجية؟
هل تم تعمية المشاركين عن التدخل الذي أعطي لهم؟
هل تم تعمية القائمين على الدراسة عن التدخل الذي كانوا يقدمونه للمشاركين؟
هل تم تعمية الأشخاص الذين يقومون بتحليل النتائج؟
هل كانت مجموعات الدراسة متشابهة في بداية التجربة؟
هل تمت معاملة جميع المشاركين على قدم المساواة؟
(CASP, 2020)

## Slide 9
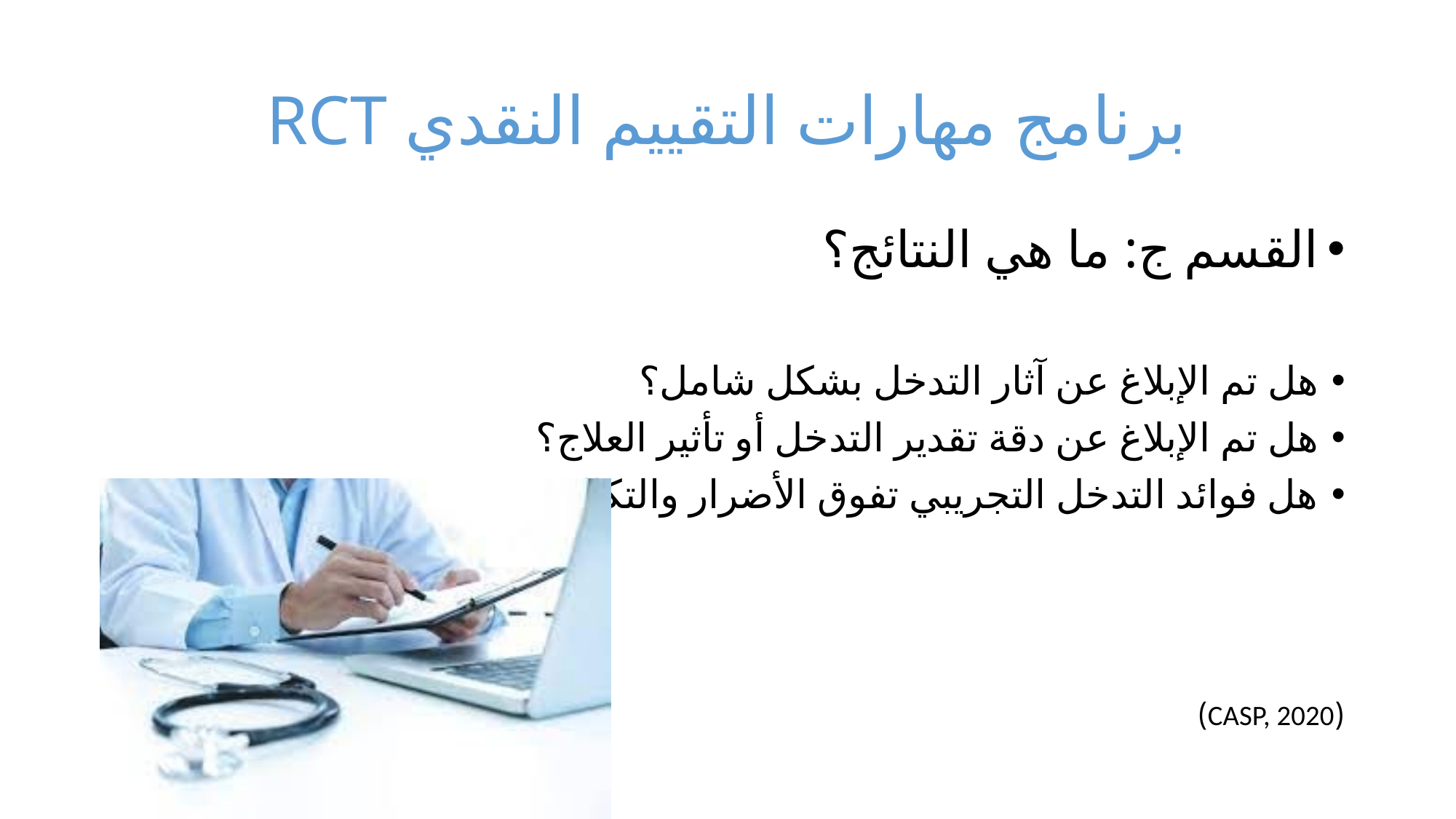

# برنامج مهارات التقييم النقدي RCT
القسم ج: ما هي النتائج؟
هل تم الإبلاغ عن آثار التدخل بشكل شامل؟
هل تم الإبلاغ عن دقة تقدير التدخل أو تأثير العلاج؟
هل فوائد التدخل التجريبي تفوق الأضرار والتكاليف؟
(CASP, 2020)

## Slide 10
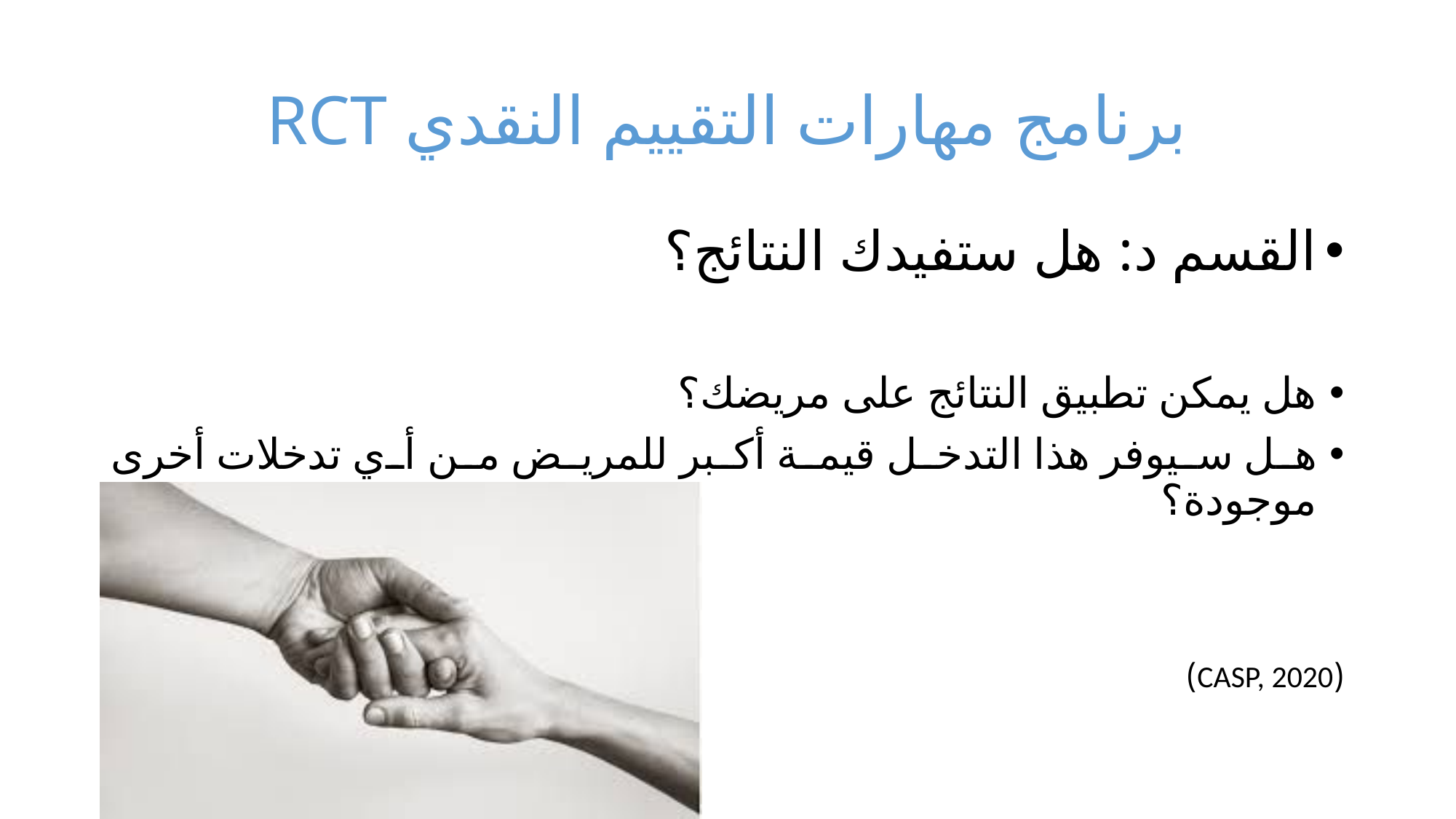

# برنامج مهارات التقييم النقدي RCT
القسم د: هل ستفيدك النتائج؟
هل يمكن تطبيق النتائج على مريضك؟
هل سيوفر هذا التدخل قيمة أكبر للمريض من أي تدخلات أخرى موجودة؟
(CASP, 2020)

## Slide 11
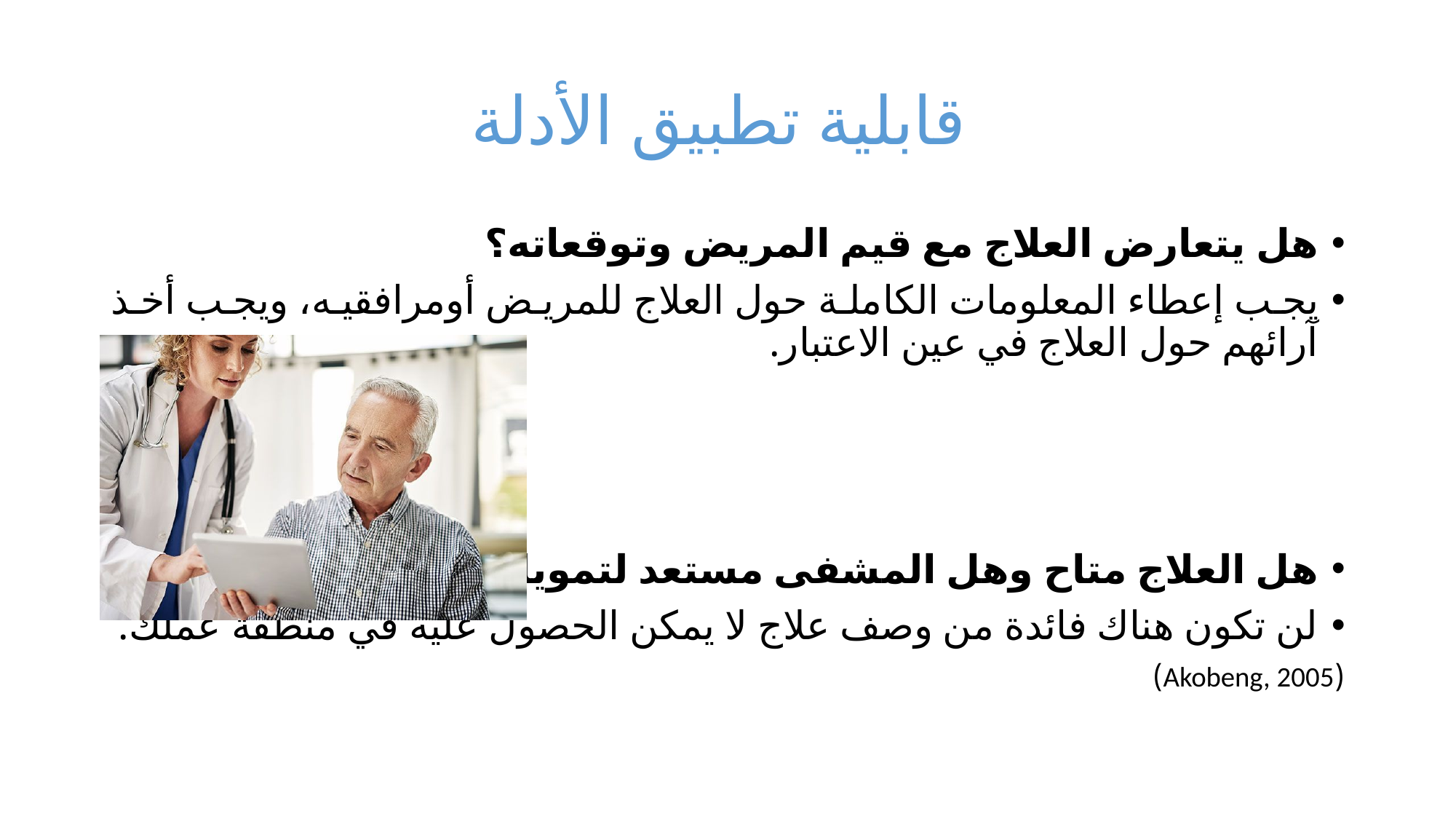

# قابلية تطبيق الأدلة
هل يتعارض العلاج مع قيم المريض وتوقعاته؟
يجب إعطاء المعلومات الكاملة حول العلاج للمريض أومرافقيه، ويجب أخذ آرائهم حول العلاج في عين الاعتبار.
هل العلاج متاح وهل المشفى مستعد لتمويله؟
لن تكون هناك فائدة من وصف علاج لا يمكن الحصول عليه في منطقة عملك.
(Akobeng, 2005)

## Slide 12
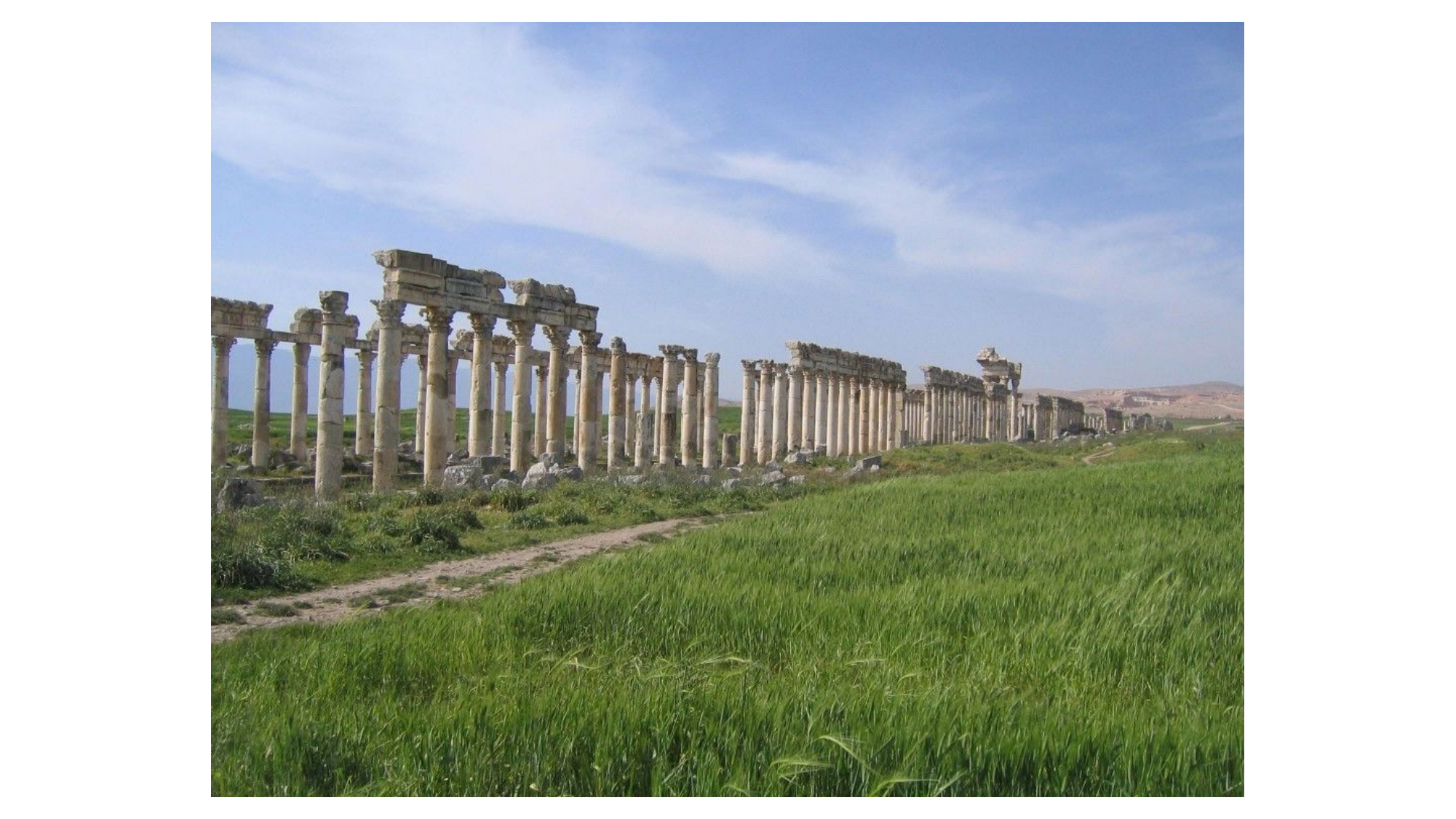

## Slide 13
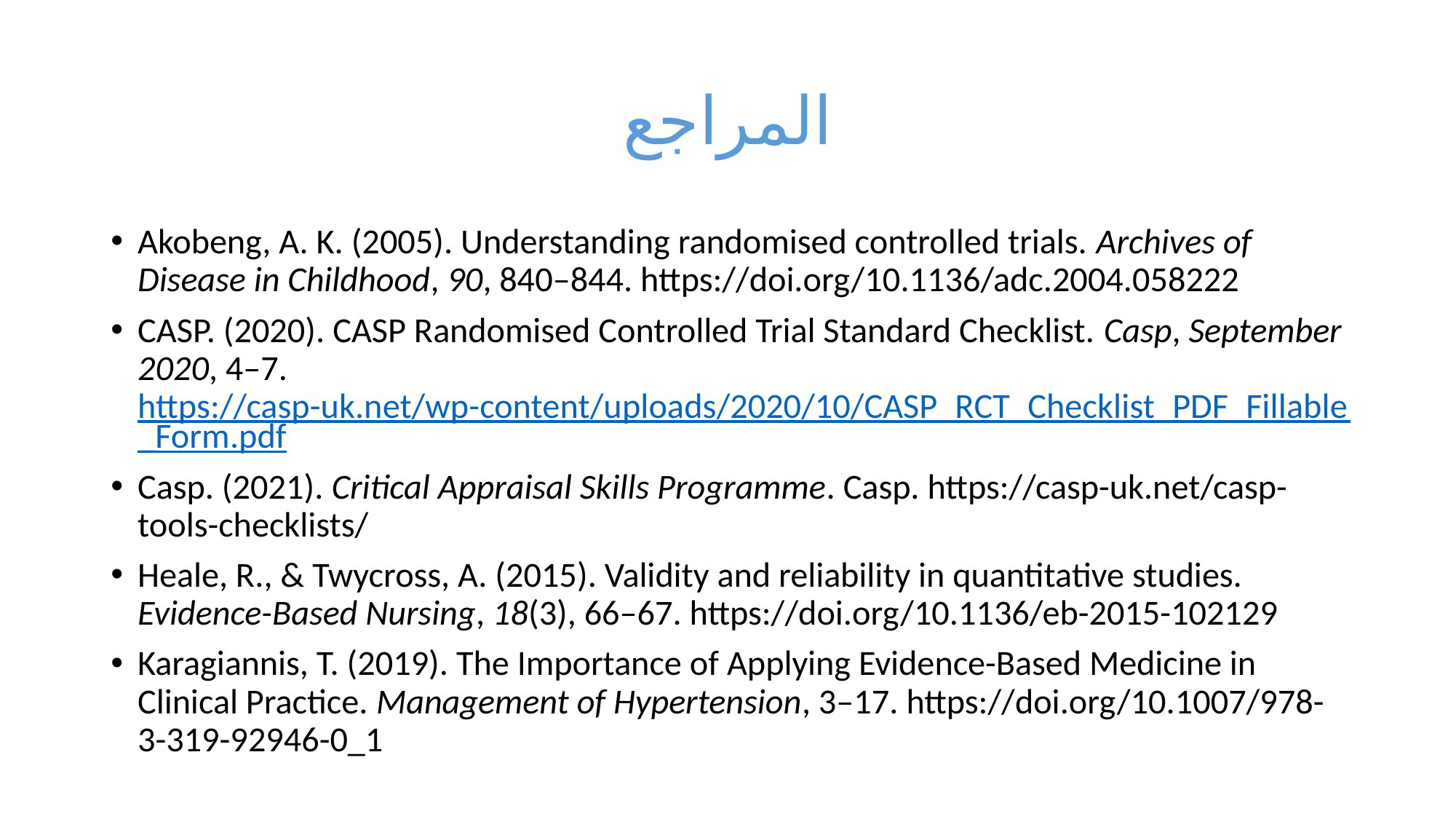

# المراجع
Akobeng, A. K. (2005). Understanding randomised controlled trials. Archives of Disease in Childhood, 90, 840–844. https://doi.org/10.1136/adc.2004.058222
CASP. (2020). CASP Randomised Controlled Trial Standard Checklist. Casp, September 2020, 4–7. https://casp-uk.net/wp-content/uploads/2020/10/CASP_RCT_Checklist_PDF_Fillable_Form.pdf
Casp. (2021). Critical Appraisal Skills Programme. Casp. https://casp-uk.net/casp-tools-checklists/
Heale, R., & Twycross, A. (2015). Validity and reliability in quantitative studies. Evidence-Based Nursing, 18(3), 66–67. https://doi.org/10.1136/eb-2015-102129
Karagiannis, T. (2019). The Importance of Applying Evidence-Based Medicine in Clinical Practice. Management of Hypertension, 3–17. https://doi.org/10.1007/978-3-319-92946-0_1
