# Supplementary material for: The First Asynchronous Online Evidence-Based Medicine Course for Syrian Health Workforce: Effectiveness and Feasibility Pilot Study
Source: JMIR Form Res. 2022 Oct 25;6(10):e36782. doi: 10.2196/36782 (PMC9644249; doi:10.2196/36782)
Supplement: Multimedia Appendix 9 [file formative_v6i10e36782_app9.pptx]

## Slide 1
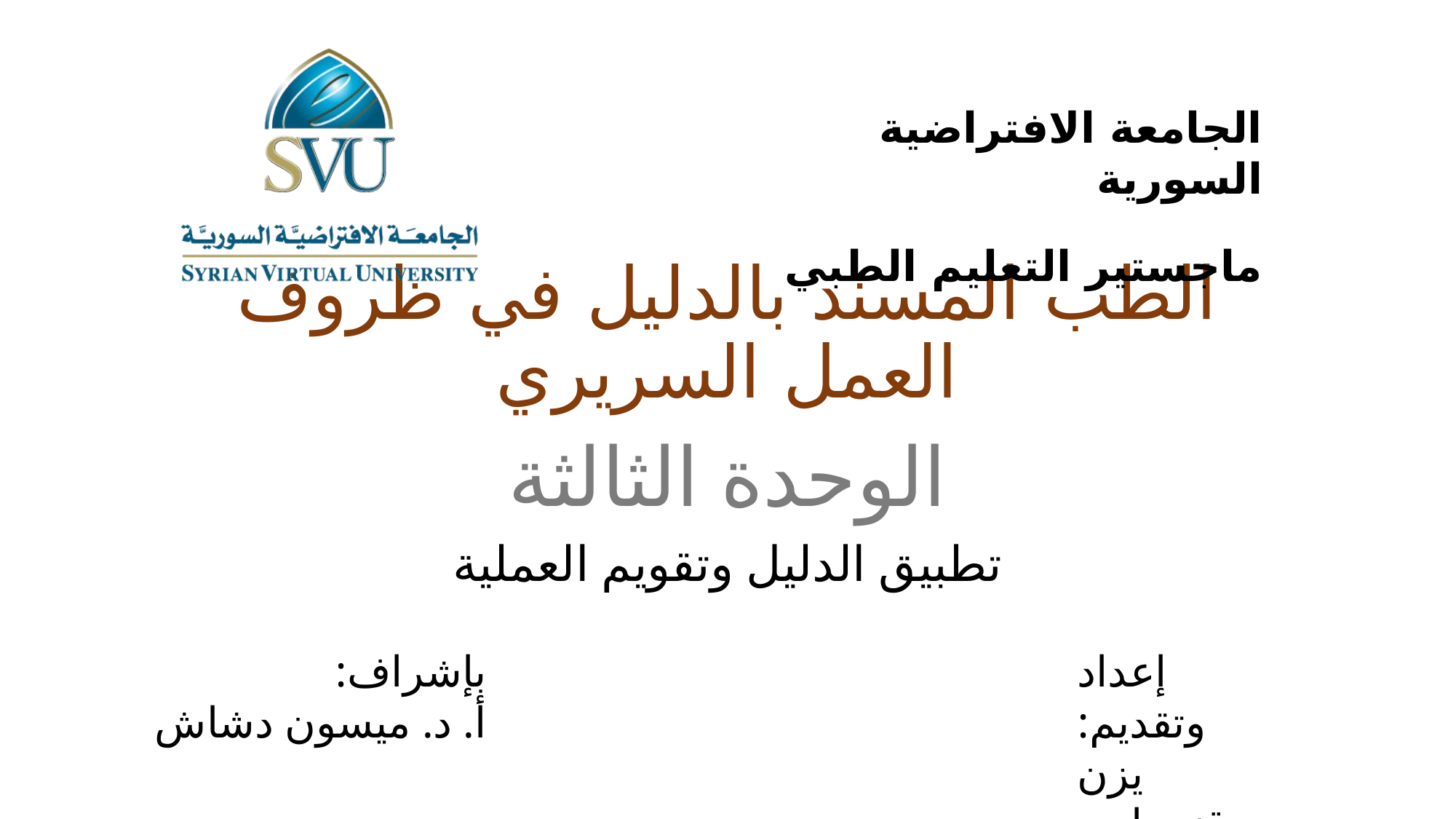

الجامعة الافتراضية السورية
ماجستير التعليم الطبي
# الطب المسند بالدليل في ظروف العمل السريري
الوحدة الثالثة
تطبيق الدليل وتقويم العملية
إعداد وتقديم:
يزن قنجراوي
بإشراف:
أ. د. ميسون دشاش

## Slide 2
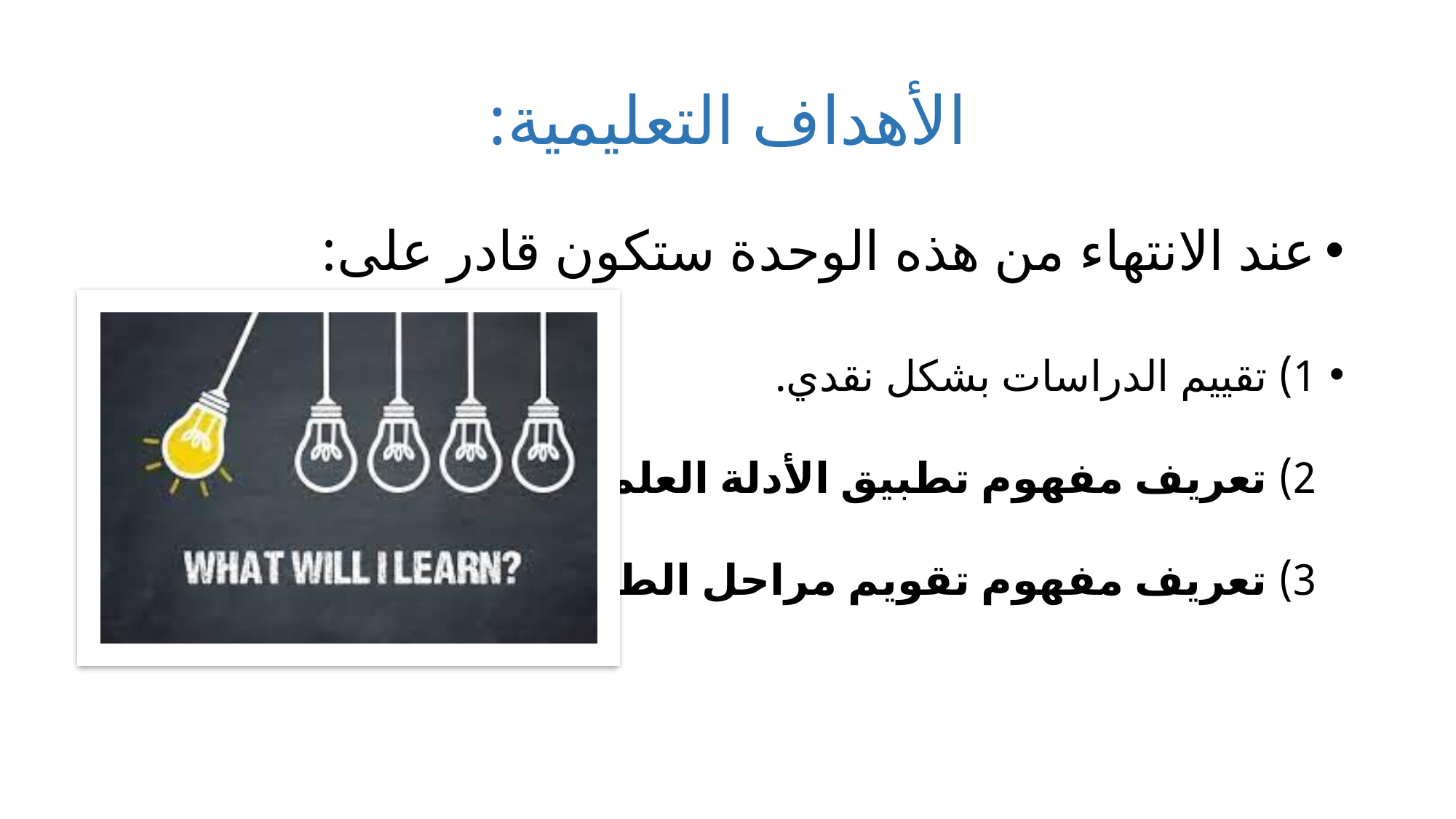

# الأهداف التعليمية:
عند الانتهاء من هذه الوحدة ستكون قادر على:
1) تقييم الدراسات بشكل نقدي.
2) تعريف مفهوم تطبيق الأدلة العلمية.
3) تعريف مفهوم تقويم مراحل الطب المسند بالدليل.

## Slide 3
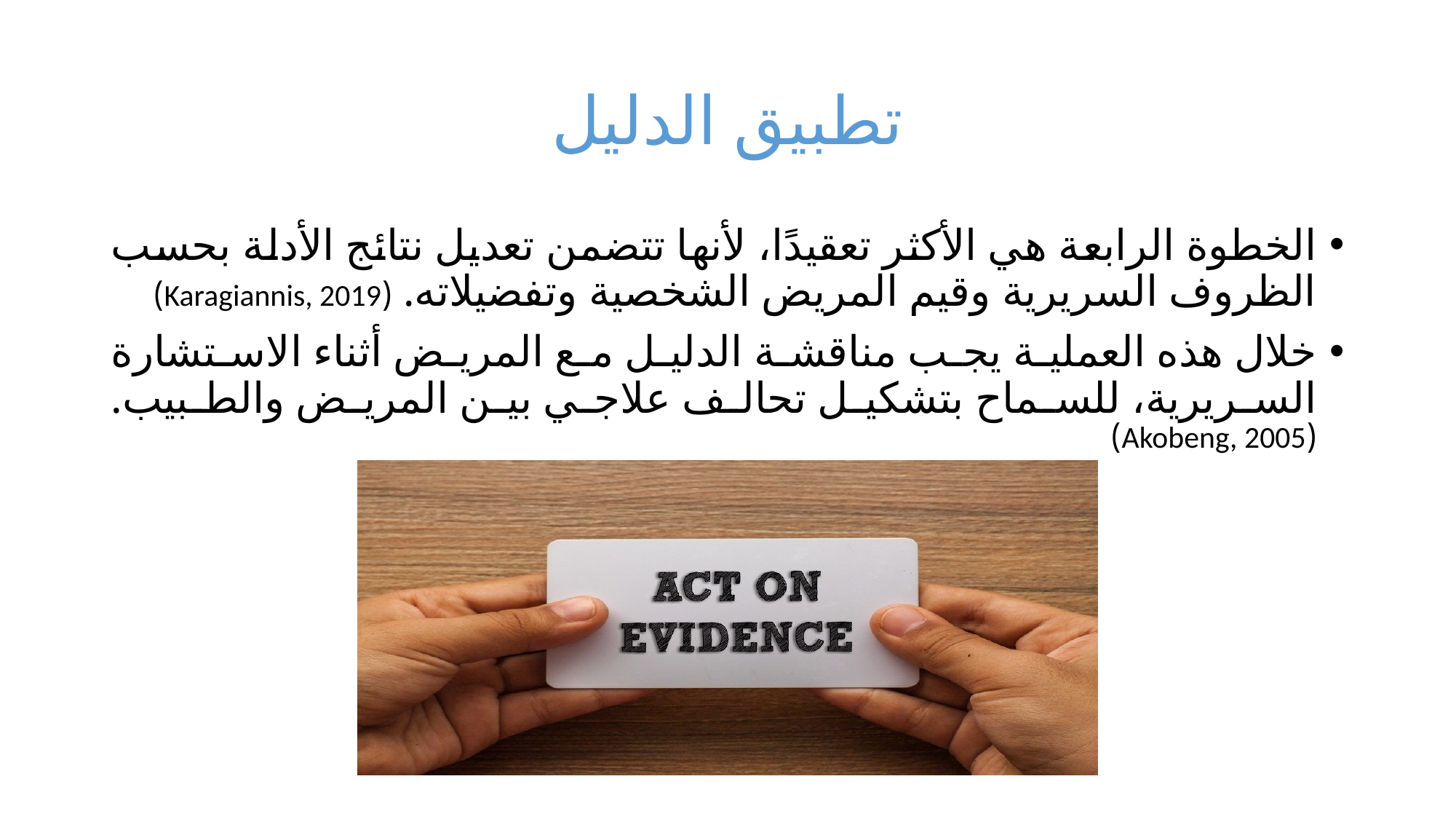

# تطبيق الدليل
الخطوة الرابعة هي الأكثر تعقيدًا، لأنها تتضمن تعديل نتائج الأدلة بحسب الظروف السريرية وقيم المريض الشخصية وتفضيلاته. (Karagiannis, 2019)
خلال هذه العملية يجب مناقشة الدليل مع المريض أثناء الاستشارة السريرية، للسماح بتشكيل تحالف علاجي بين المريض والطبيب. (Akobeng, 2005)

## Slide 4
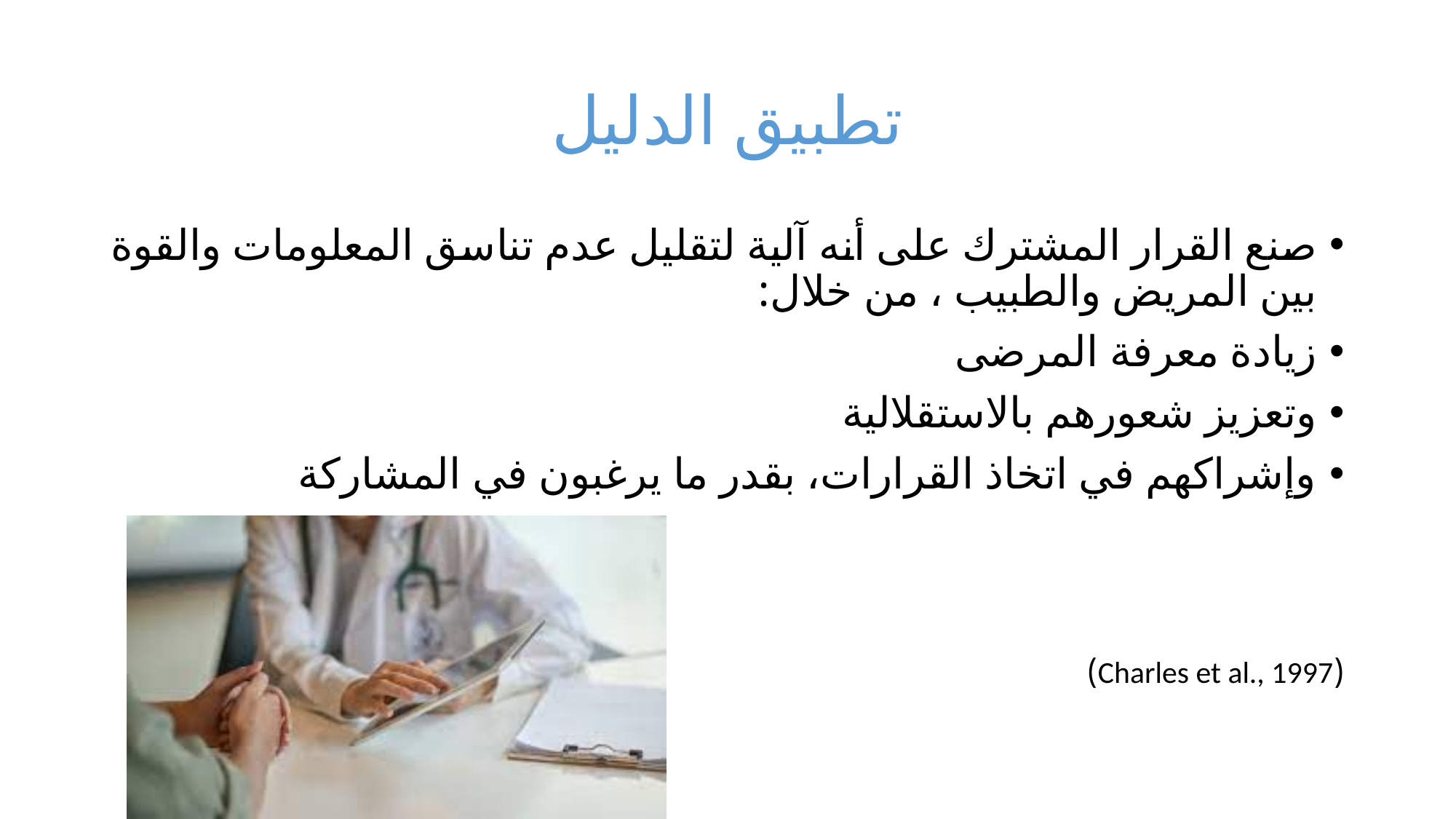

# تطبيق الدليل
صنع القرار المشترك على أنه آلية لتقليل عدم تناسق المعلومات والقوة بين المريض والطبيب ، من خلال:
زيادة معرفة المرضى
وتعزيز شعورهم بالاستقلالية
وإشراكهم في اتخاذ القرارات، بقدر ما يرغبون في المشاركة
(Charles et al., 1997)

## Slide 5
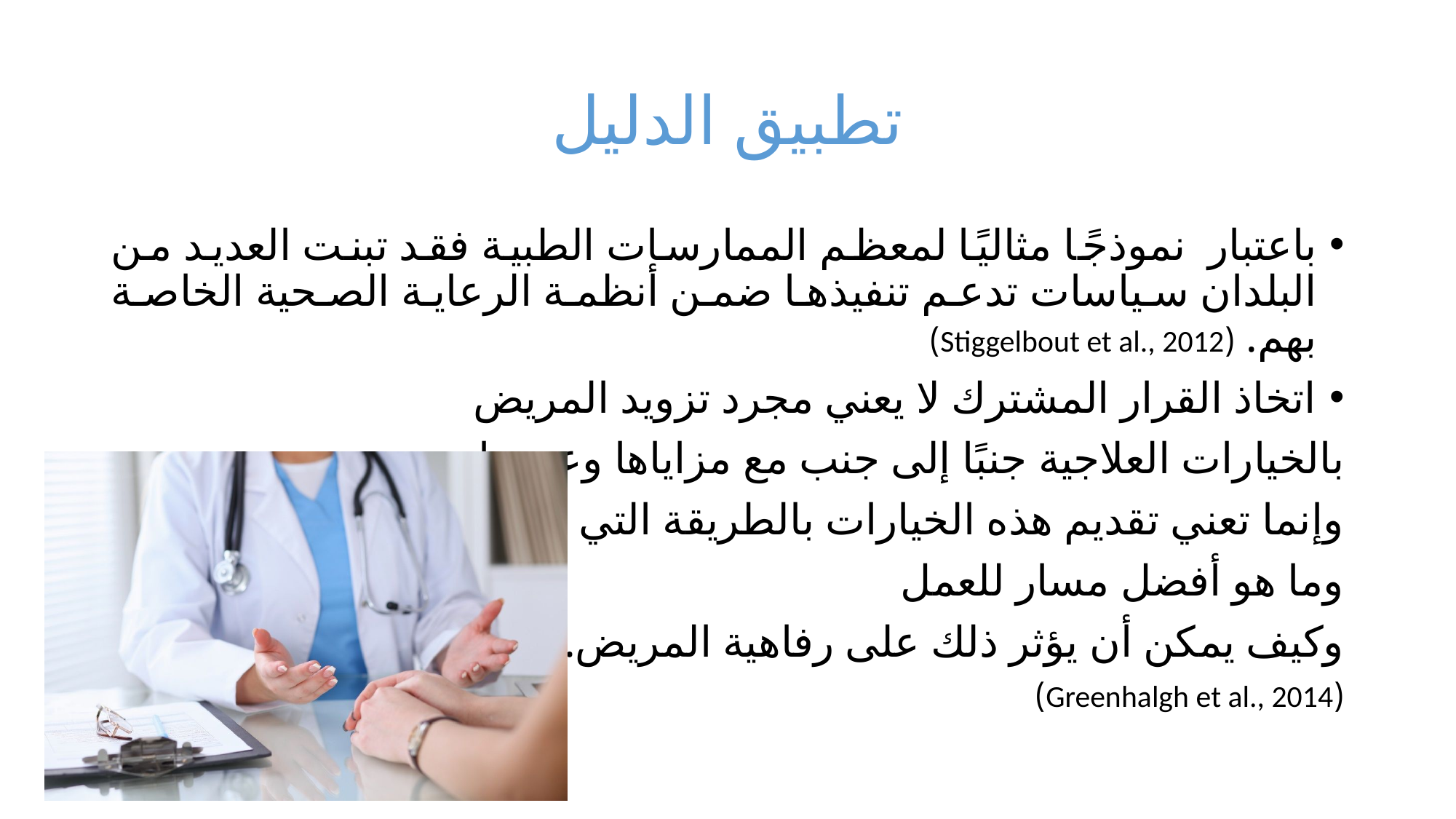

# تطبيق الدليل
باعتبار نموذجًا مثاليًا لمعظم الممارسات الطبية فقد تبنت العديد من البلدان سياسات تدعم تنفيذها ضمن أنظمة الرعاية الصحية الخاصة بهم. (Stiggelbout et al., 2012)
اتخاذ القرار المشترك لا يعني مجرد تزويد المريض
بالخيارات العلاجية جنبًا إلى جنب مع مزاياها وعيوبها.
وإنما تعني تقديم هذه الخيارات بالطريقة التي تهم المريض
وما هو أفضل مسار للعمل
وكيف يمكن أن يؤثر ذلك على رفاهية المريض.
(Greenhalgh et al., 2014)

## Slide 6
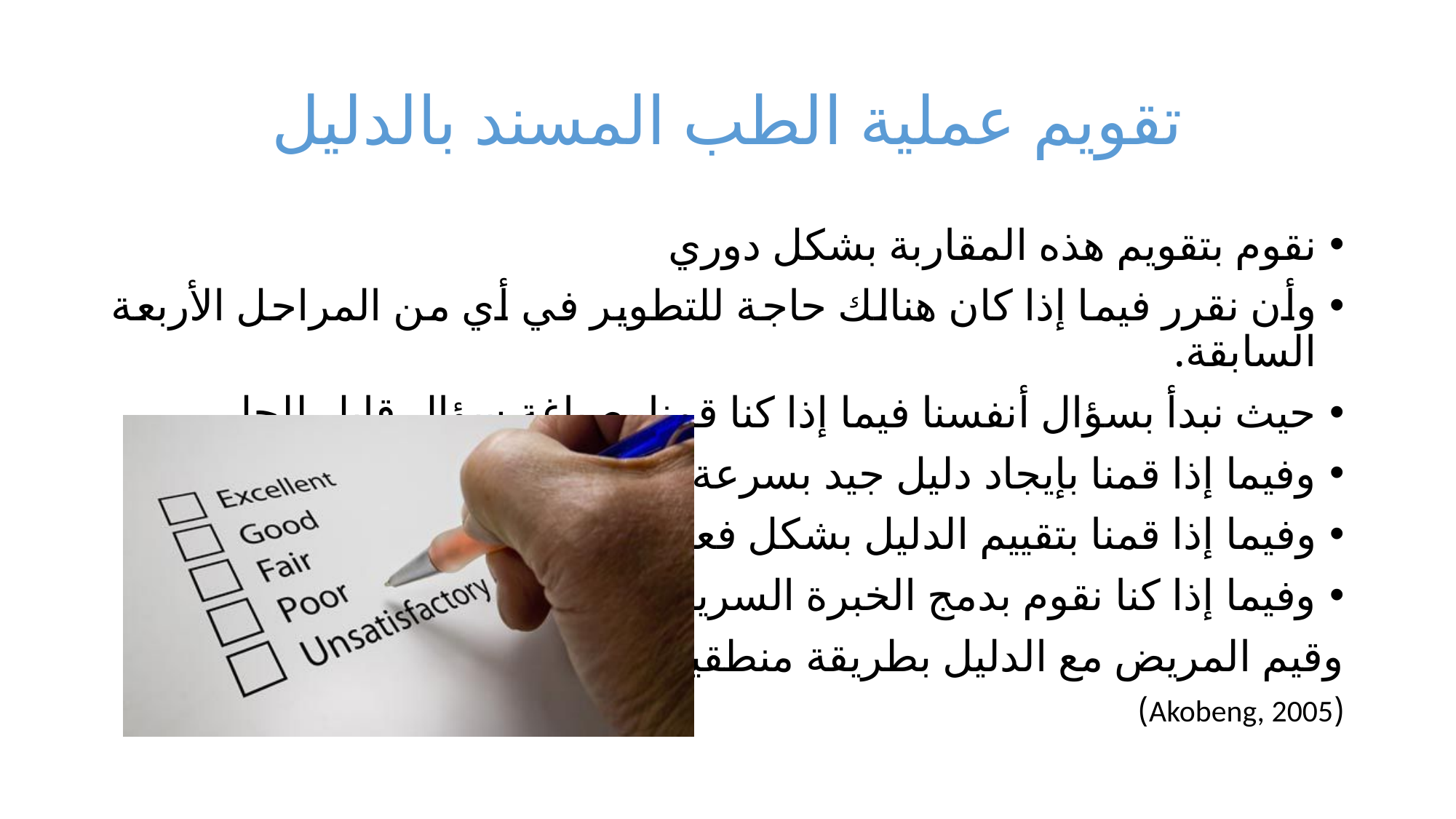

# تقويم عملية الطب المسند بالدليل
نقوم بتقويم هذه المقاربة بشكل دوري
وأن نقرر فيما إذا كان هنالك حاجة للتطوير في أي من المراحل الأربعة السابقة.
حيث نبدأ بسؤال أنفسنا فيما إذا كنا قمنا بصياغة سؤال قابل للحل.
وفيما إذا قمنا بإيجاد دليل جيد بسرعة.
وفيما إذا قمنا بتقييم الدليل بشكل فعال.
وفيما إذا كنا نقوم بدمج الخبرة السريرية
وقيم المريض مع الدليل بطريقة منطقية مقبولة.
(Akobeng, 2005)

## Slide 7
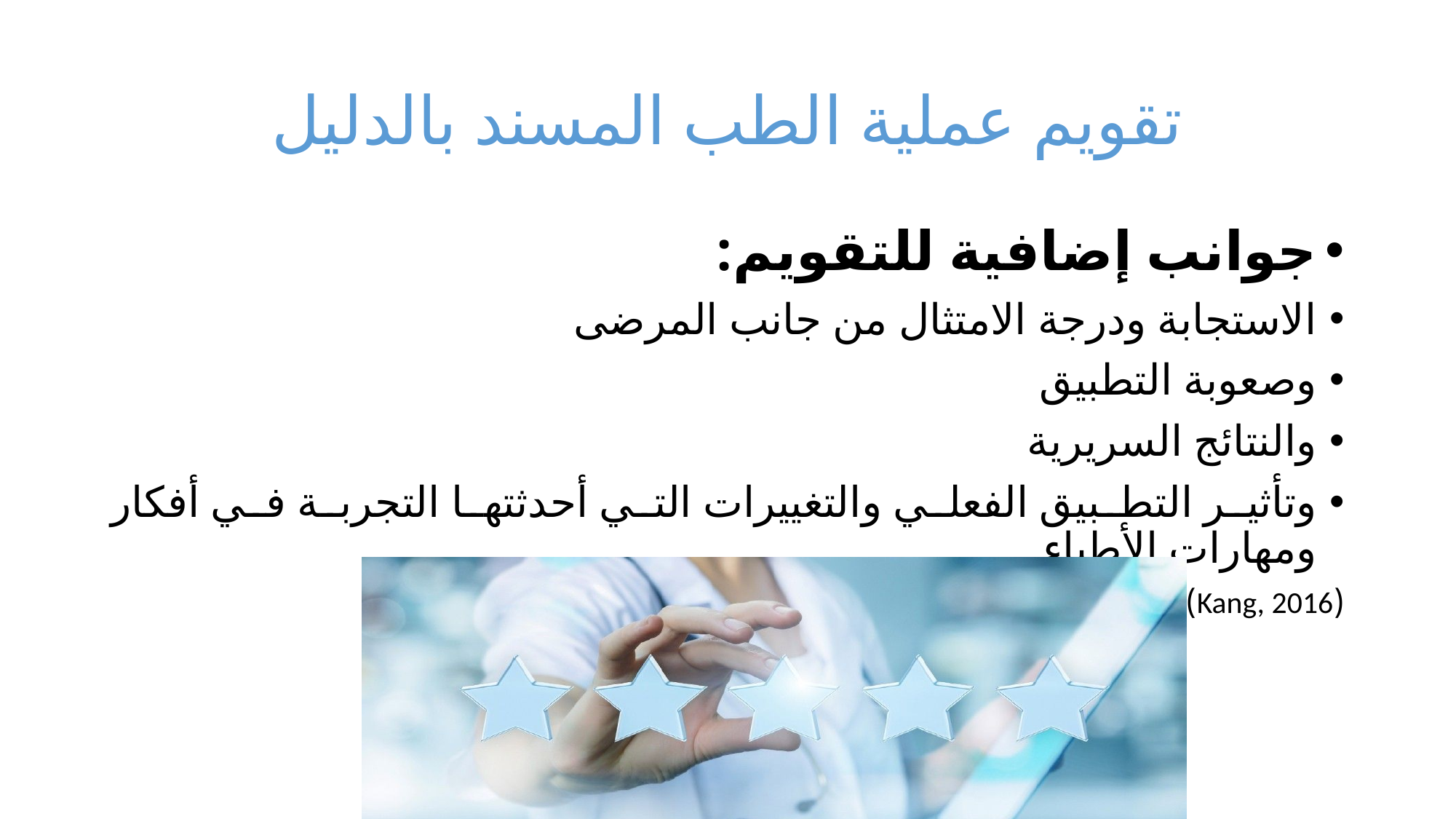

# تقويم عملية الطب المسند بالدليل
جوانب إضافية للتقويم:
الاستجابة ودرجة الامتثال من جانب المرضى
وصعوبة التطبيق
والنتائج السريرية
وتأثير التطبيق الفعلي والتغييرات التي أحدثتها التجربة في أفكار ومهارات الأطباء.
(Kang, 2016)

## Slide 8
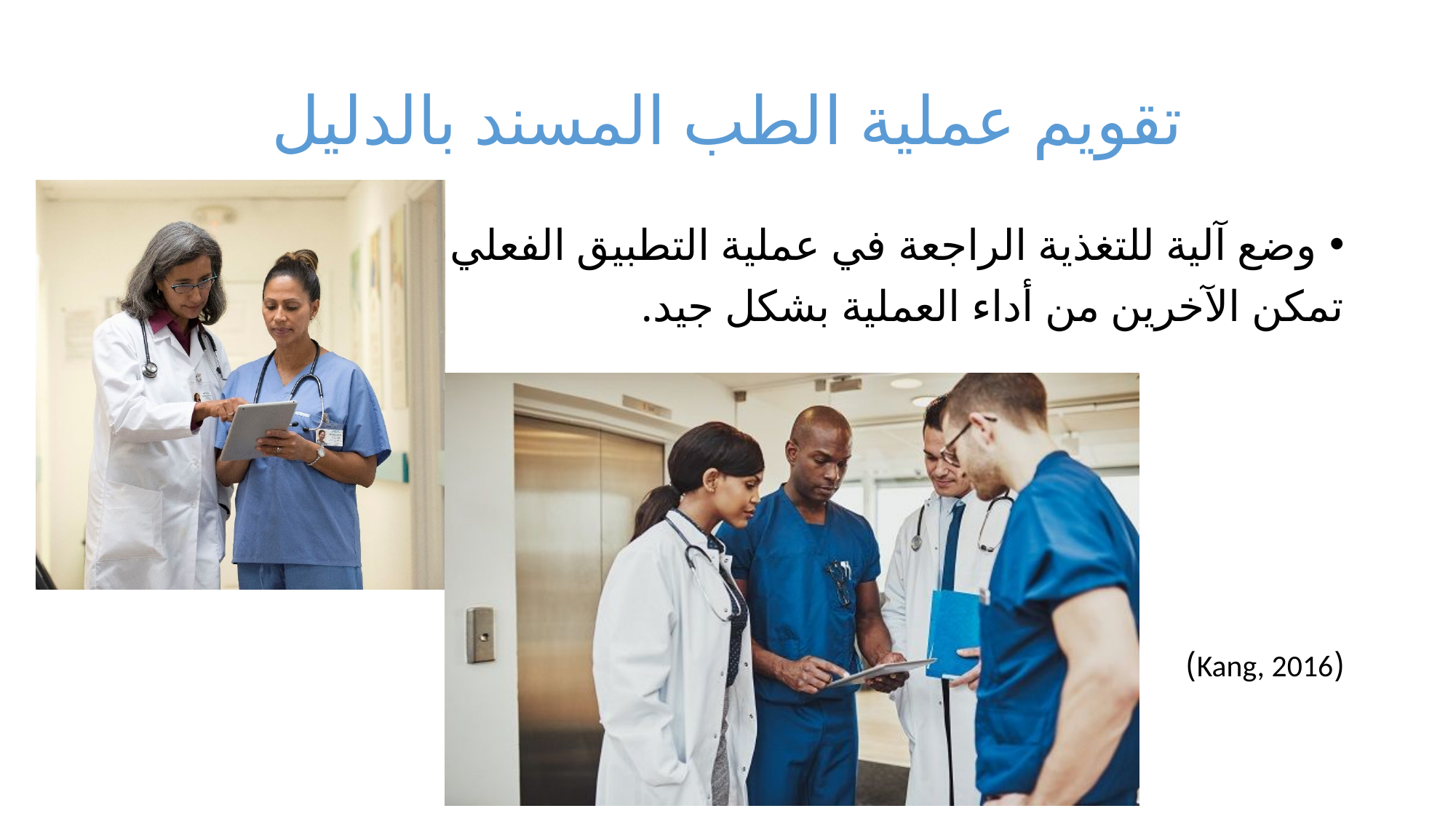

# تقويم عملية الطب المسند بالدليل
وضع آلية للتغذية الراجعة في عملية التطبيق الفعلي للأدلة،
تمكن الآخرين من أداء العملية بشكل جيد.
(Kang, 2016)

## Slide 9
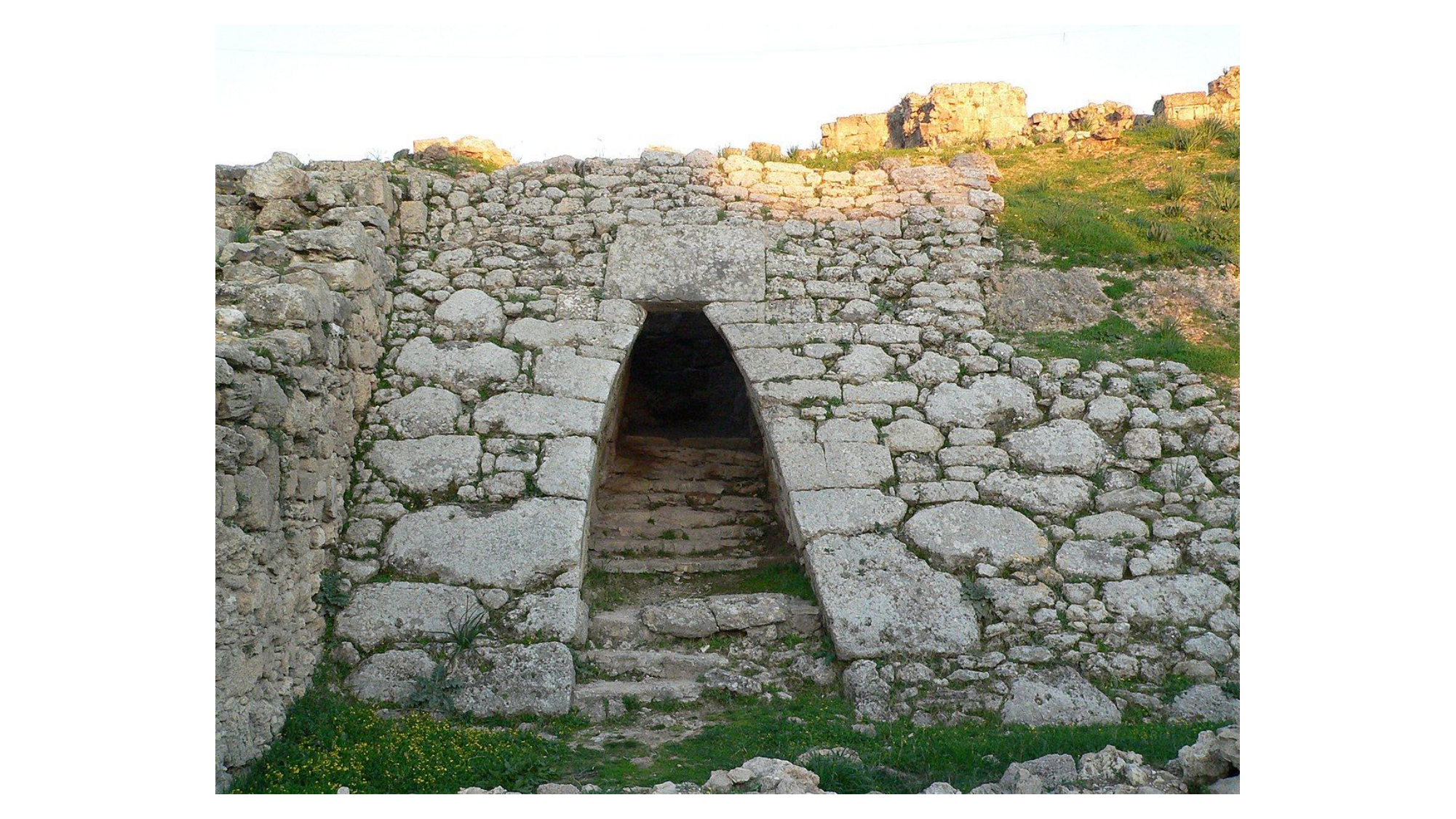

## Slide 10
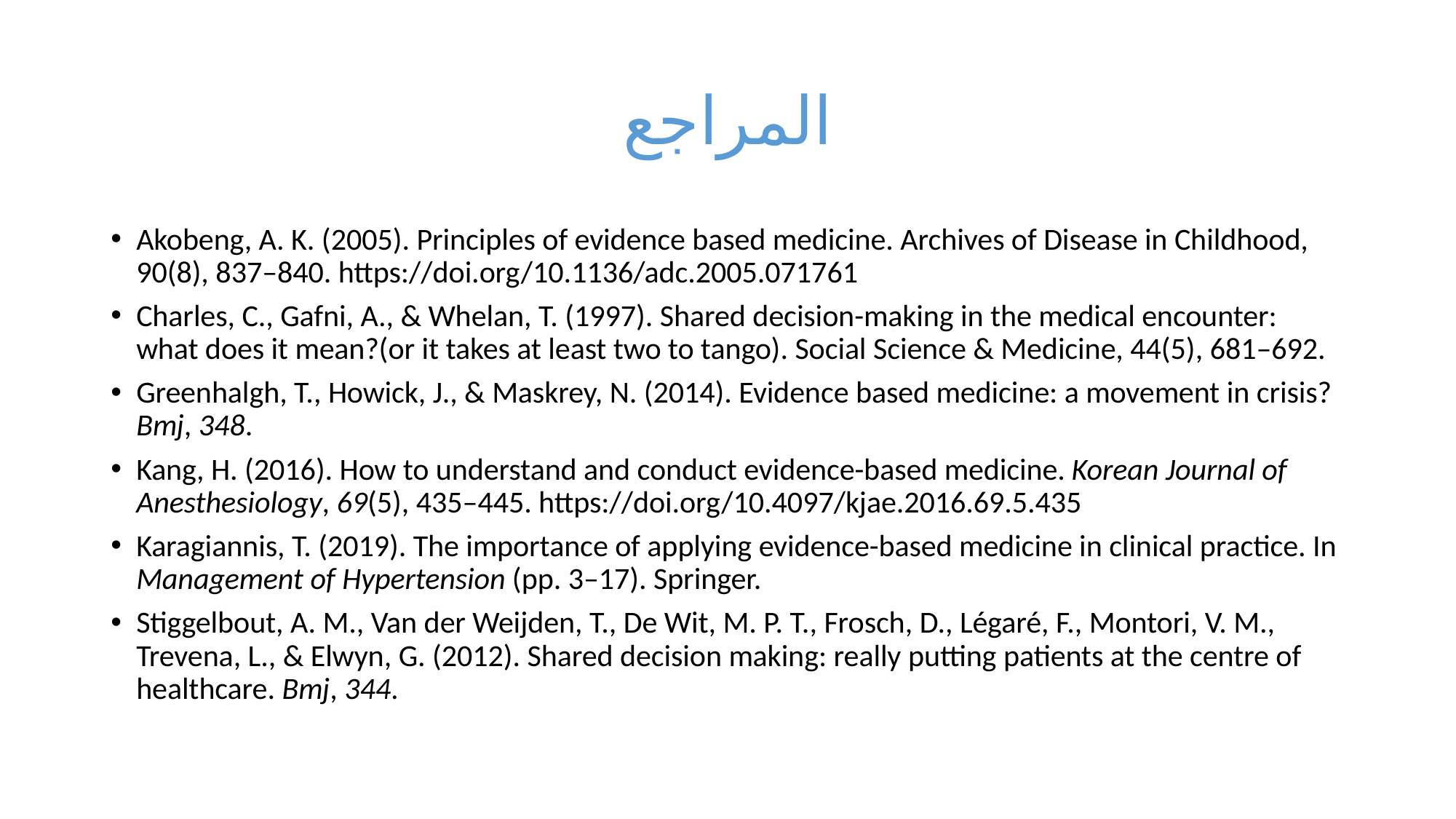

# المراجع
Akobeng, A. K. (2005). Principles of evidence based medicine. Archives of Disease in Childhood, 90(8), 837–840. https://doi.org/10.1136/adc.2005.071761
Charles, C., Gafni, A., & Whelan, T. (1997). Shared decision-making in the medical encounter: what does it mean?(or it takes at least two to tango). Social Science & Medicine, 44(5), 681–692.
Greenhalgh, T., Howick, J., & Maskrey, N. (2014). Evidence based medicine: a movement in crisis? Bmj, 348.
Kang, H. (2016). How to understand and conduct evidence-based medicine. Korean Journal of Anesthesiology, 69(5), 435–445. https://doi.org/10.4097/kjae.2016.69.5.435
Karagiannis, T. (2019). The importance of applying evidence-based medicine in clinical practice. In Management of Hypertension (pp. 3–17). Springer.
Stiggelbout, A. M., Van der Weijden, T., De Wit, M. P. T., Frosch, D., Légaré, F., Montori, V. M., Trevena, L., & Elwyn, G. (2012). Shared decision making: really putting patients at the centre of healthcare. Bmj, 344.
