# Supplementary material for: The First Asynchronous Online Evidence-Based Medicine Course for Syrian Health Workforce: Effectiveness and Feasibility Pilot Study
Source: JMIR Form Res. 2022 Oct 25;6(10):e36782. doi: 10.2196/36782 (PMC9644249; doi:10.2196/36782)
Supplement: Multimedia Appendix 10 [file formative_v6i10e36782_app10.pptx]

## Slide 1
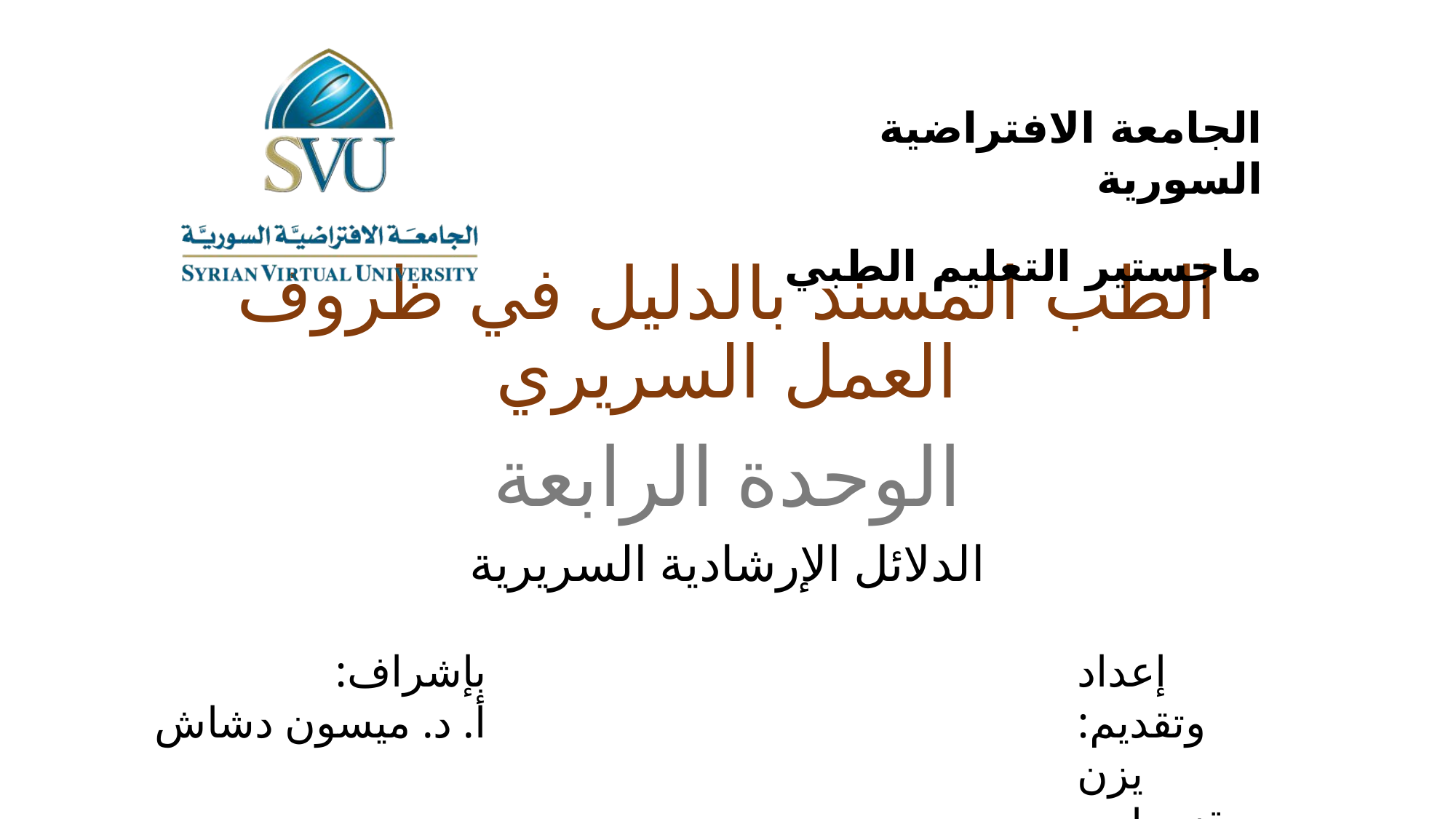

الجامعة الافتراضية السورية
ماجستير التعليم الطبي
# الطب المسند بالدليل في ظروف العمل السريري
الوحدة الرابعة
الدلائل الإرشادية السريرية
إعداد وتقديم:
يزن قنجراوي
بإشراف:
أ. د. ميسون دشاش

## Slide 2
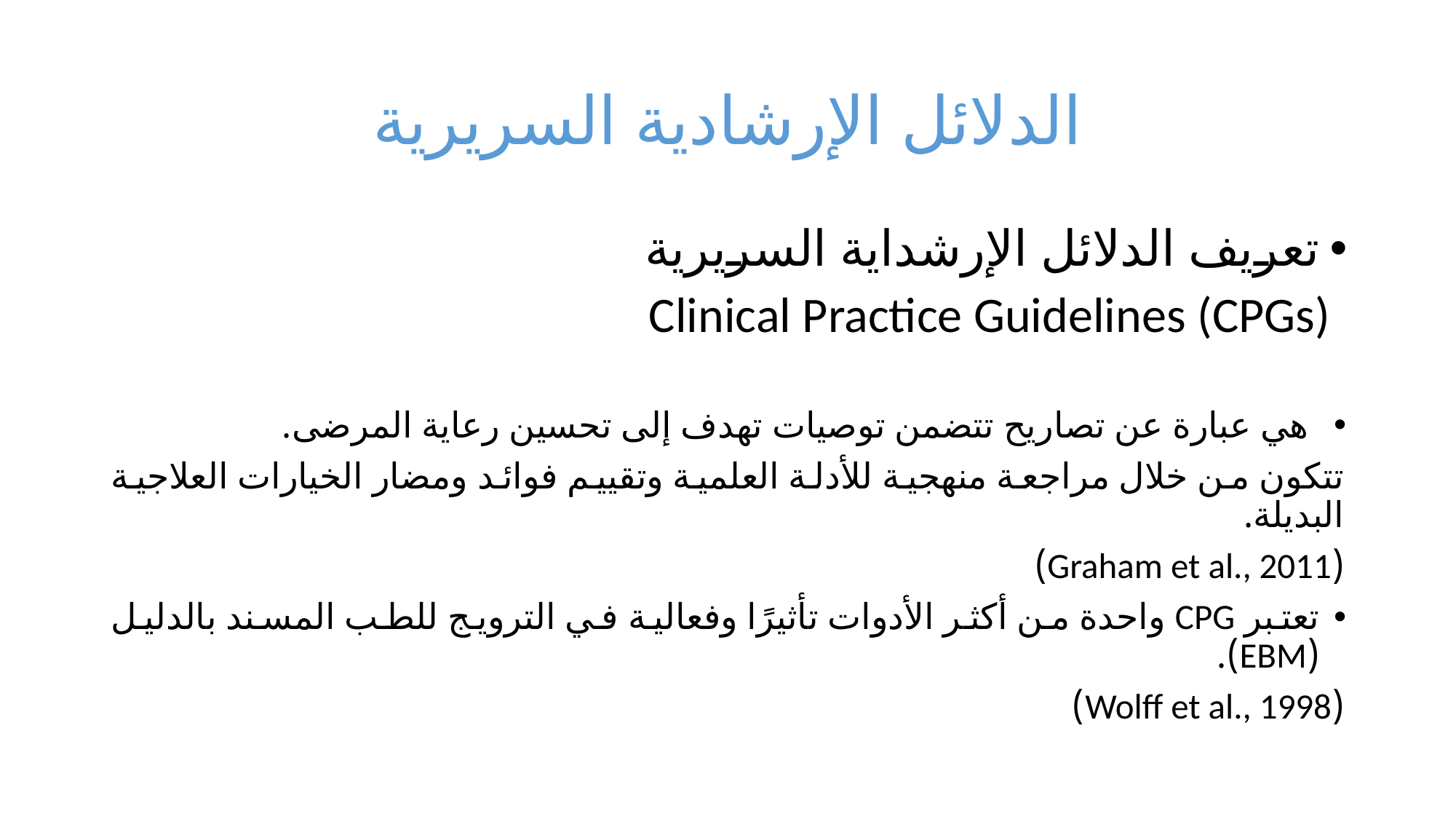

# الدلائل الإرشادية السريرية
تعريف الدلائل الإرشداية السريرية
 Clinical Practice Guidelines (CPGs)
 هي عبارة عن تصاريح تتضمن توصيات تهدف إلى تحسين رعاية المرضى.
تتكون من خلال مراجعة منهجية للأدلة العلمية وتقييم فوائد ومضار الخيارات العلاجية البديلة.
(Graham et al., 2011)
تعتبر CPG واحدة من أكثر الأدوات تأثيرًا وفعالية في الترويج للطب المسند بالدليل (EBM).
(Wolff et al., 1998)

## Slide 3
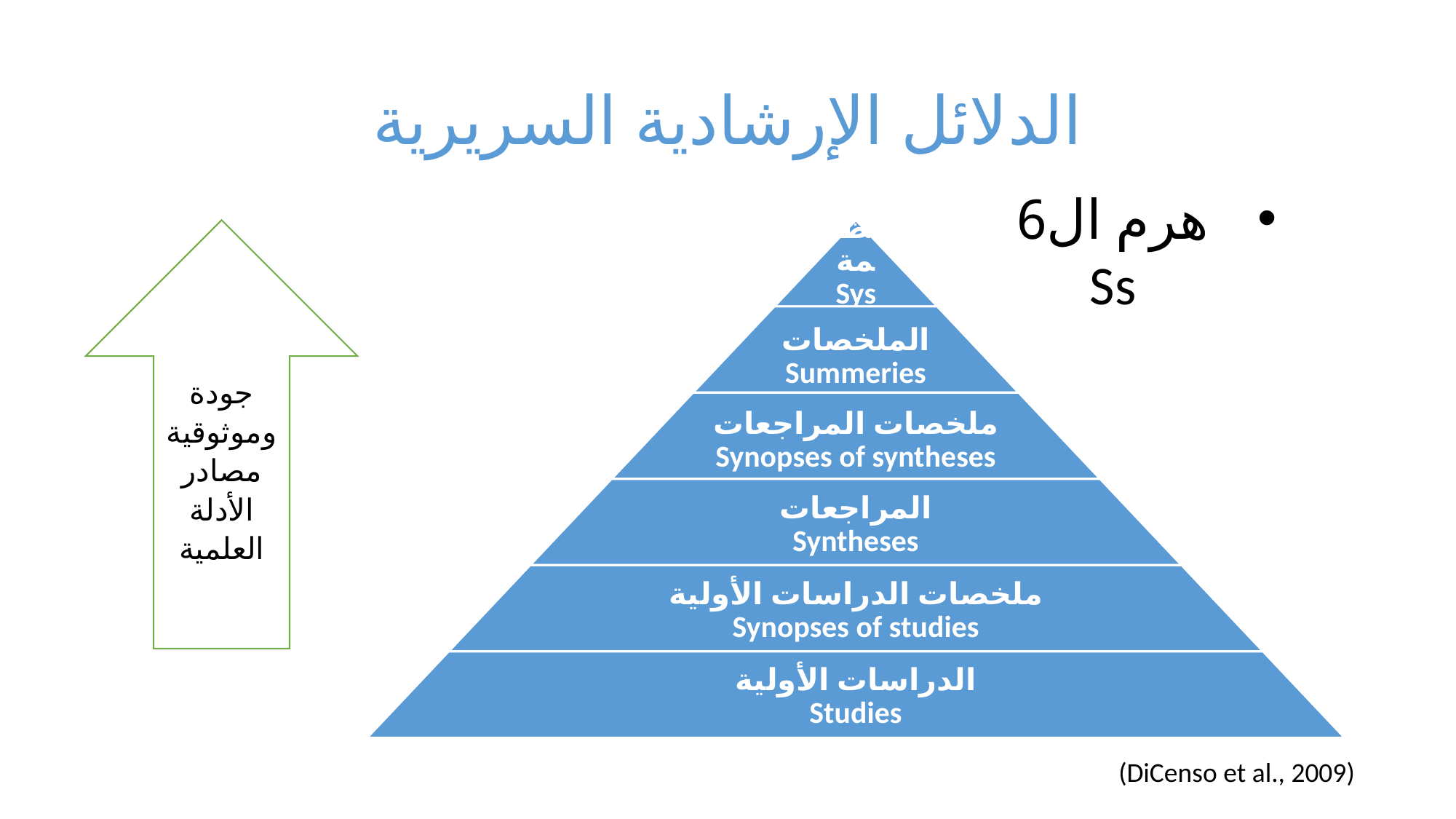

# الدلائل الإرشادية السريرية
هرم ال6 Ss
جودة وموثوقية مصادر الأدلة العلمية
(DiCenso et al., 2009)

## Slide 4
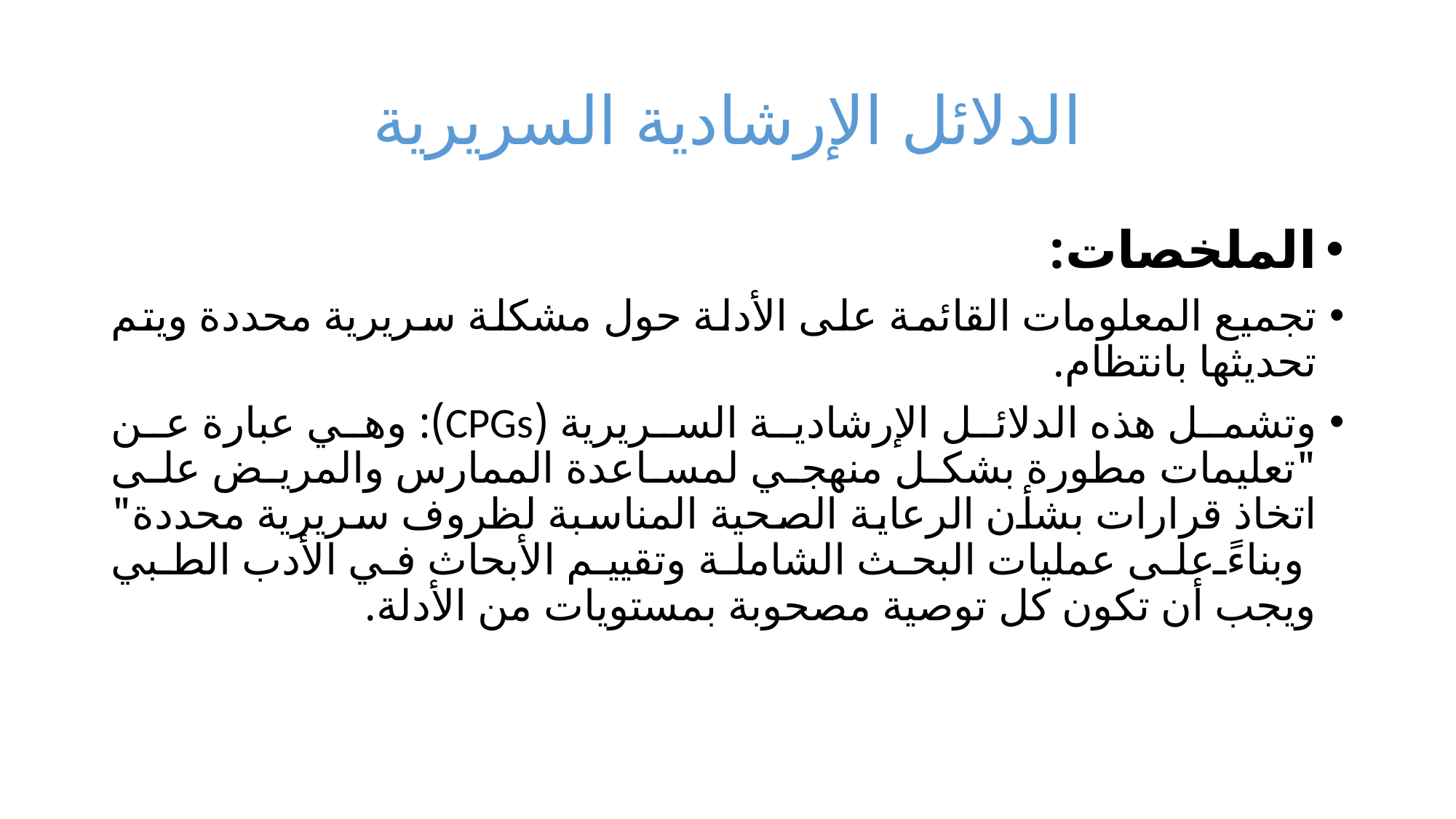

# الدلائل الإرشادية السريرية
الملخصات:
تجميع المعلومات القائمة على الأدلة حول مشكلة سريرية محددة ويتم تحديثها بانتظام.
وتشمل هذه الدلائل الإرشادية السريرية (CPGs): وهي عبارة عن "تعليمات مطورة بشكل منهجي لمساعدة الممارس والمريض على اتخاذ قرارات بشأن الرعاية الصحية المناسبة لظروف سريرية محددة" وبناءً على عمليات البحث الشاملة وتقييم الأبحاث في الأدب الطبي ويجب أن تكون كل توصية مصحوبة بمستويات من الأدلة.

## Slide 5
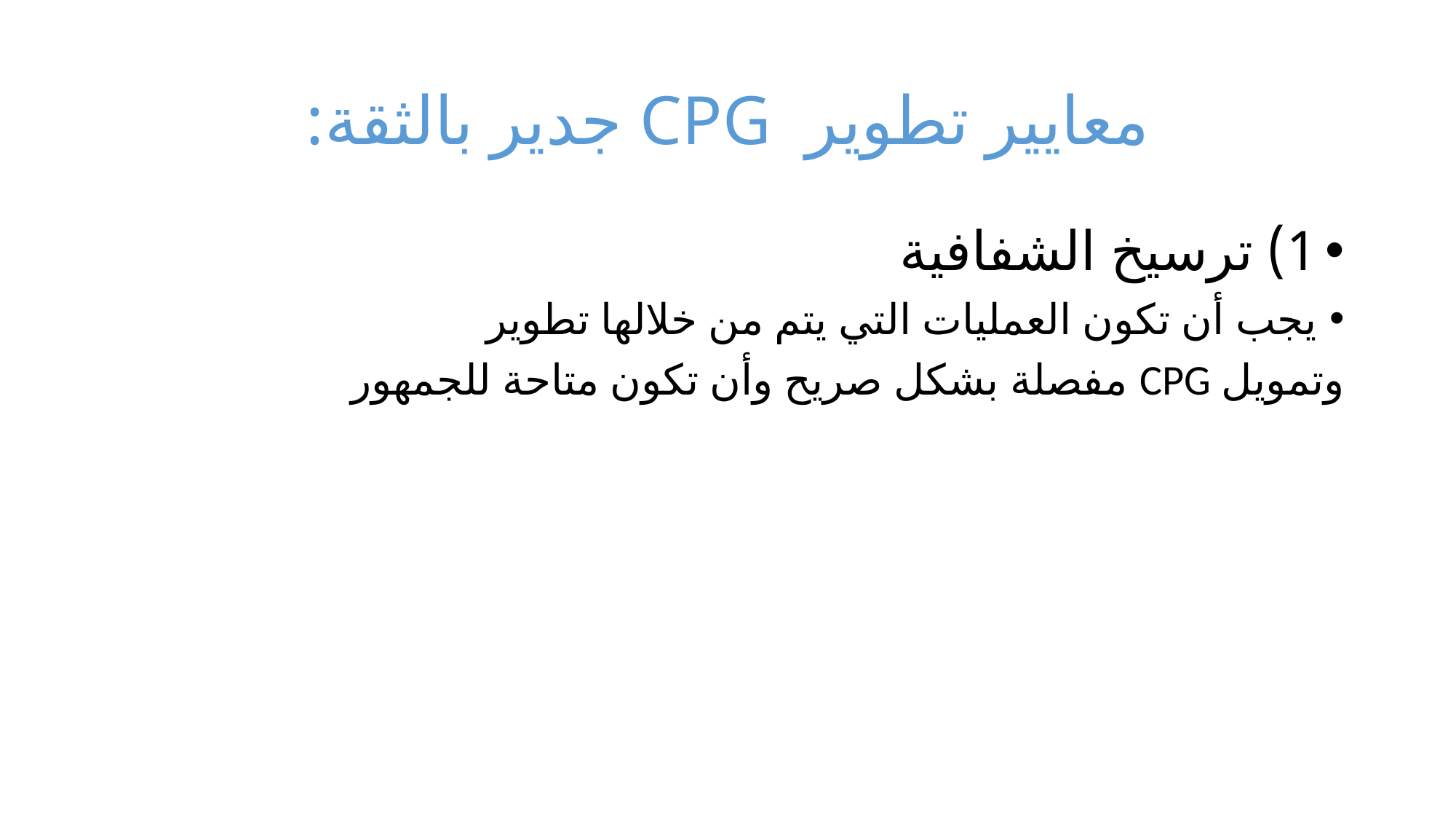

# معايير تطوير CPG جدير بالثقة:
1) ترسيخ الشفافية
يجب أن تكون العمليات التي يتم من خلالها تطوير
وتمويل CPG مفصلة بشكل صريح وأن تكون متاحة للجمهور

## Slide 6
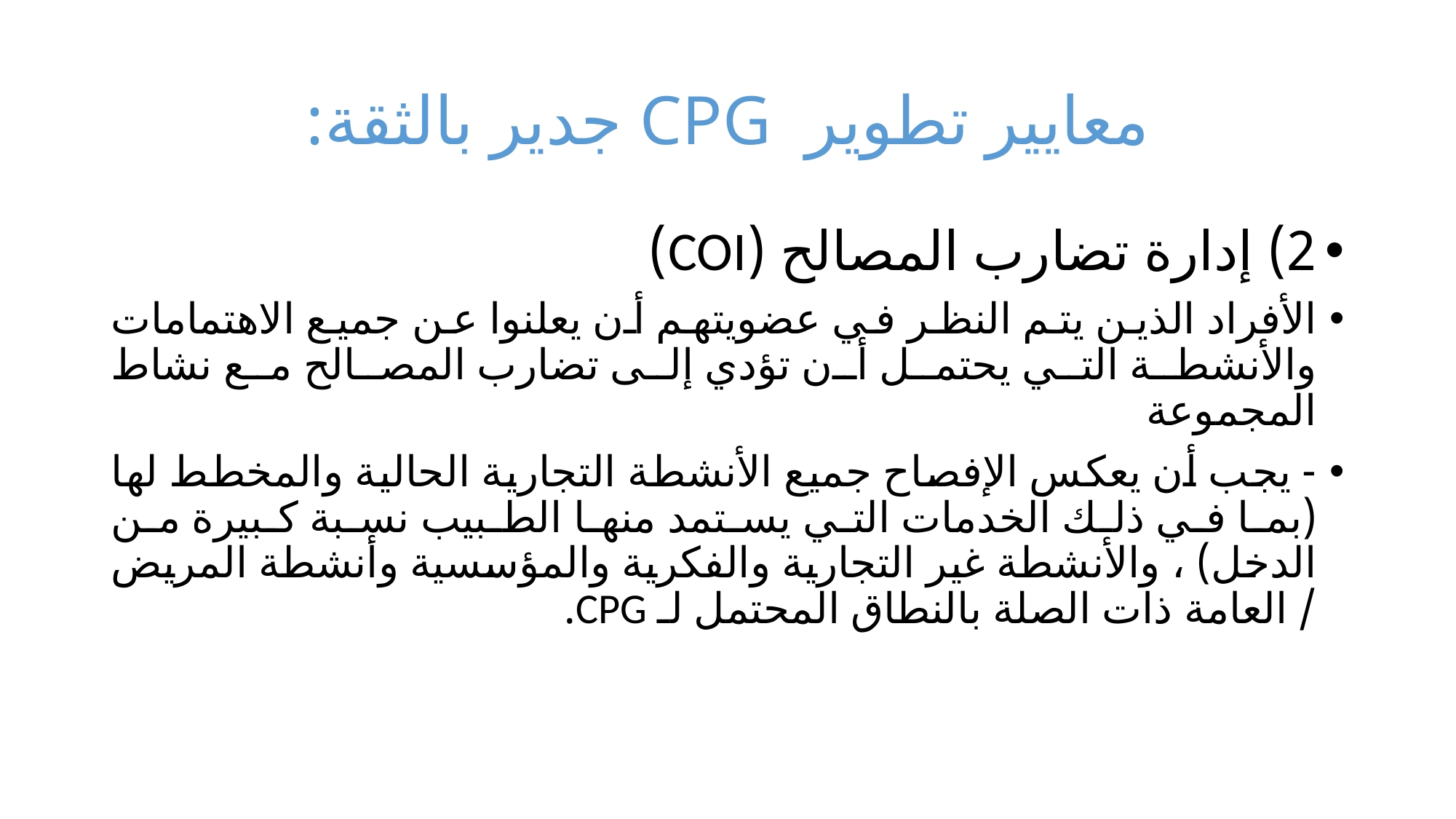

# معايير تطوير CPG جدير بالثقة:
2) إدارة تضارب المصالح (COI)
الأفراد الذين يتم النظر في عضويتهم أن يعلنوا عن جميع الاهتمامات والأنشطة التي يحتمل أن تؤدي إلى تضارب المصالح مع نشاط المجموعة
- يجب أن يعكس الإفصاح جميع الأنشطة التجارية الحالية والمخطط لها (بما في ذلك الخدمات التي يستمد منها الطبيب نسبة كبيرة من الدخل) ، والأنشطة غير التجارية والفكرية والمؤسسية وأنشطة المريض / العامة ذات الصلة بالنطاق المحتمل لـ CPG.

## Slide 7
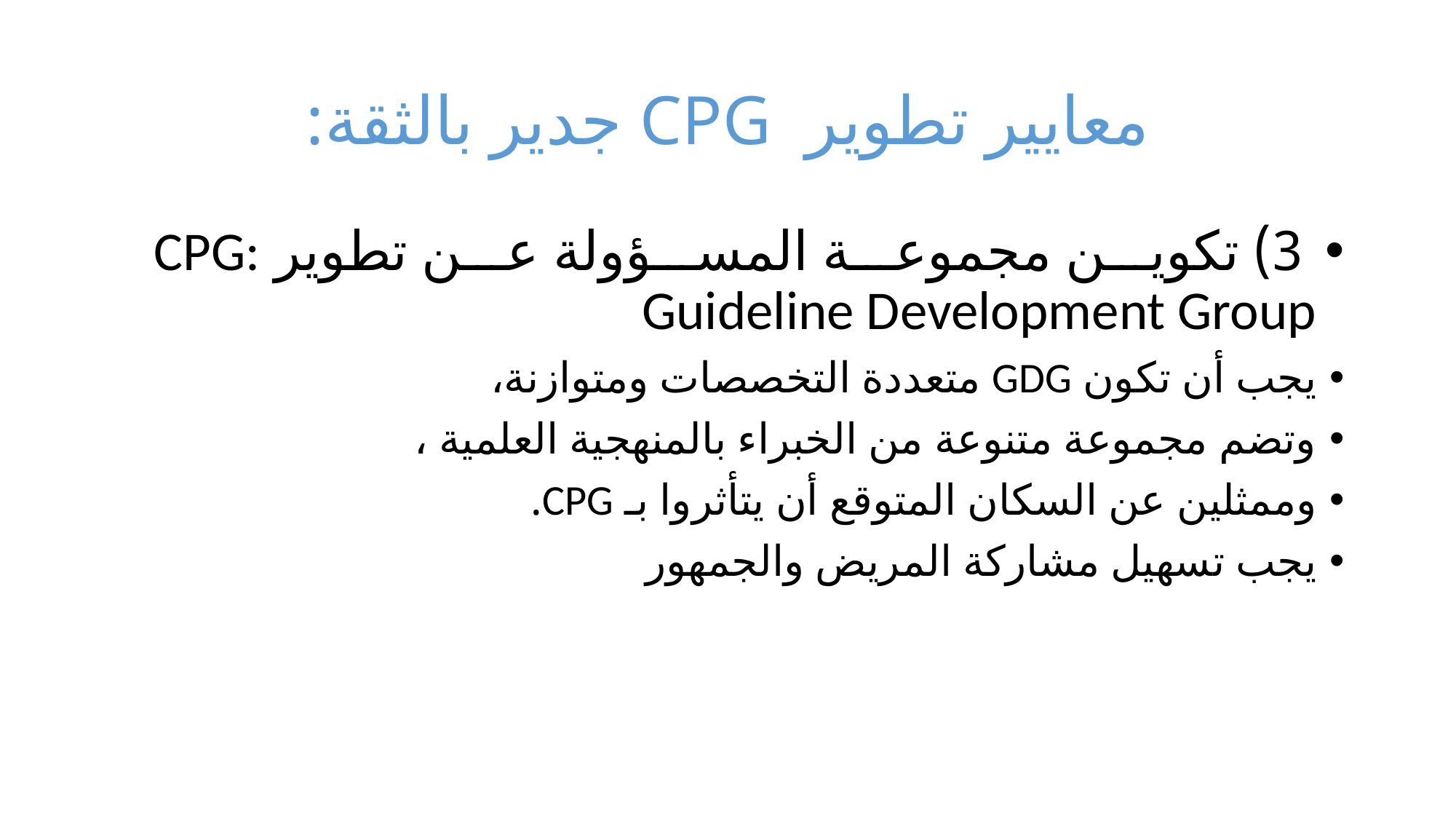

# معايير تطوير CPG جدير بالثقة:
 3) تكوين مجموعة المسؤولة عن تطوير CPG: Guideline Development Group
يجب أن تكون GDG متعددة التخصصات ومتوازنة،
وتضم مجموعة متنوعة من الخبراء بالمنهجية العلمية ،
وممثلين عن السكان المتوقع أن يتأثروا بـ CPG.
يجب تسهيل مشاركة المريض والجمهور

## Slide 8
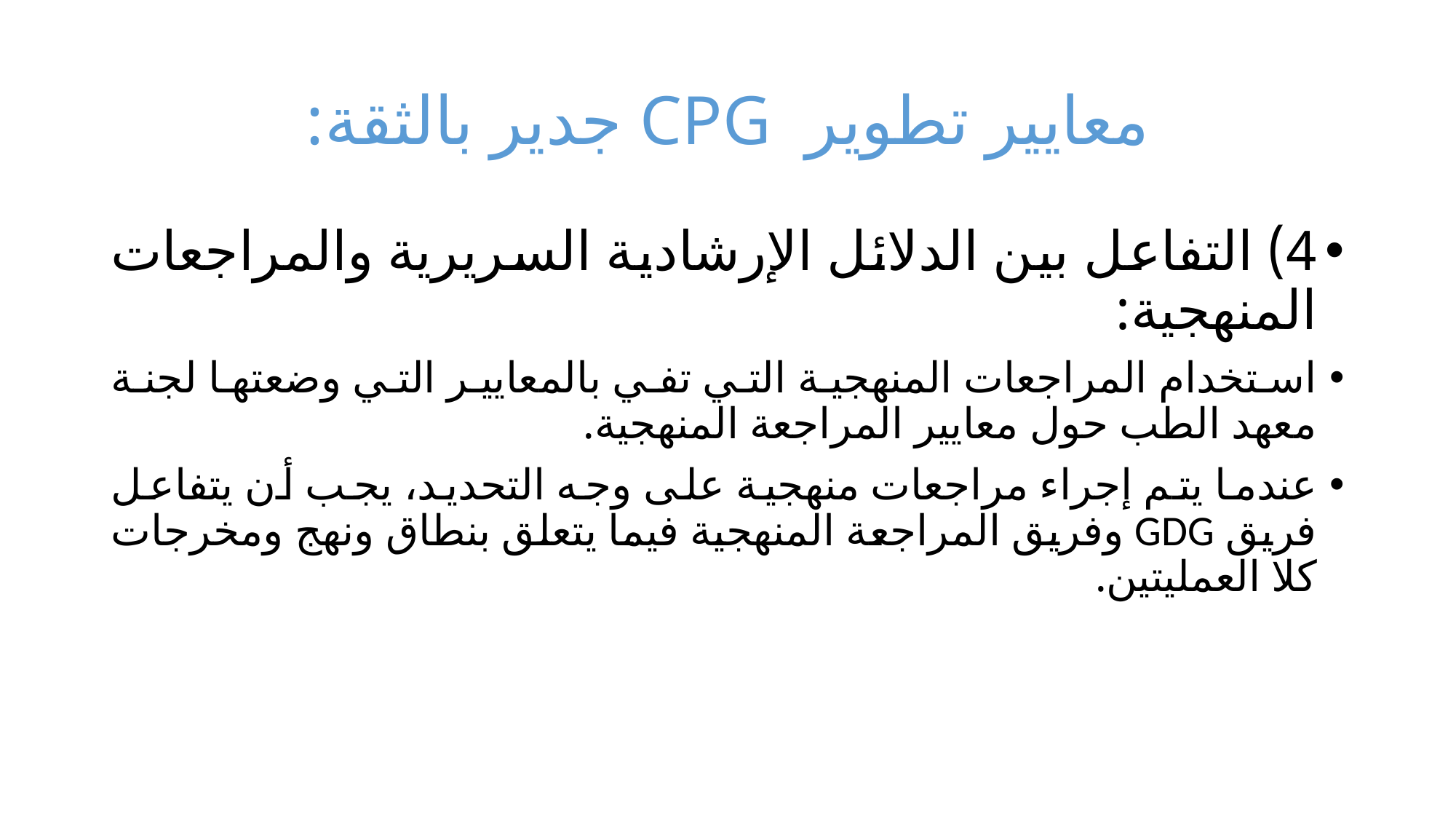

# معايير تطوير CPG جدير بالثقة:
4) التفاعل بين الدلائل الإرشادية السريرية والمراجعات المنهجية:
استخدام المراجعات المنهجية التي تفي بالمعايير التي وضعتها لجنة معهد الطب حول معايير المراجعة المنهجية.
عندما يتم إجراء مراجعات منهجية على وجه التحديد، يجب أن يتفاعل فريق GDG وفريق المراجعة المنهجية فيما يتعلق بنطاق ونهج ومخرجات كلا العمليتين.

## Slide 9
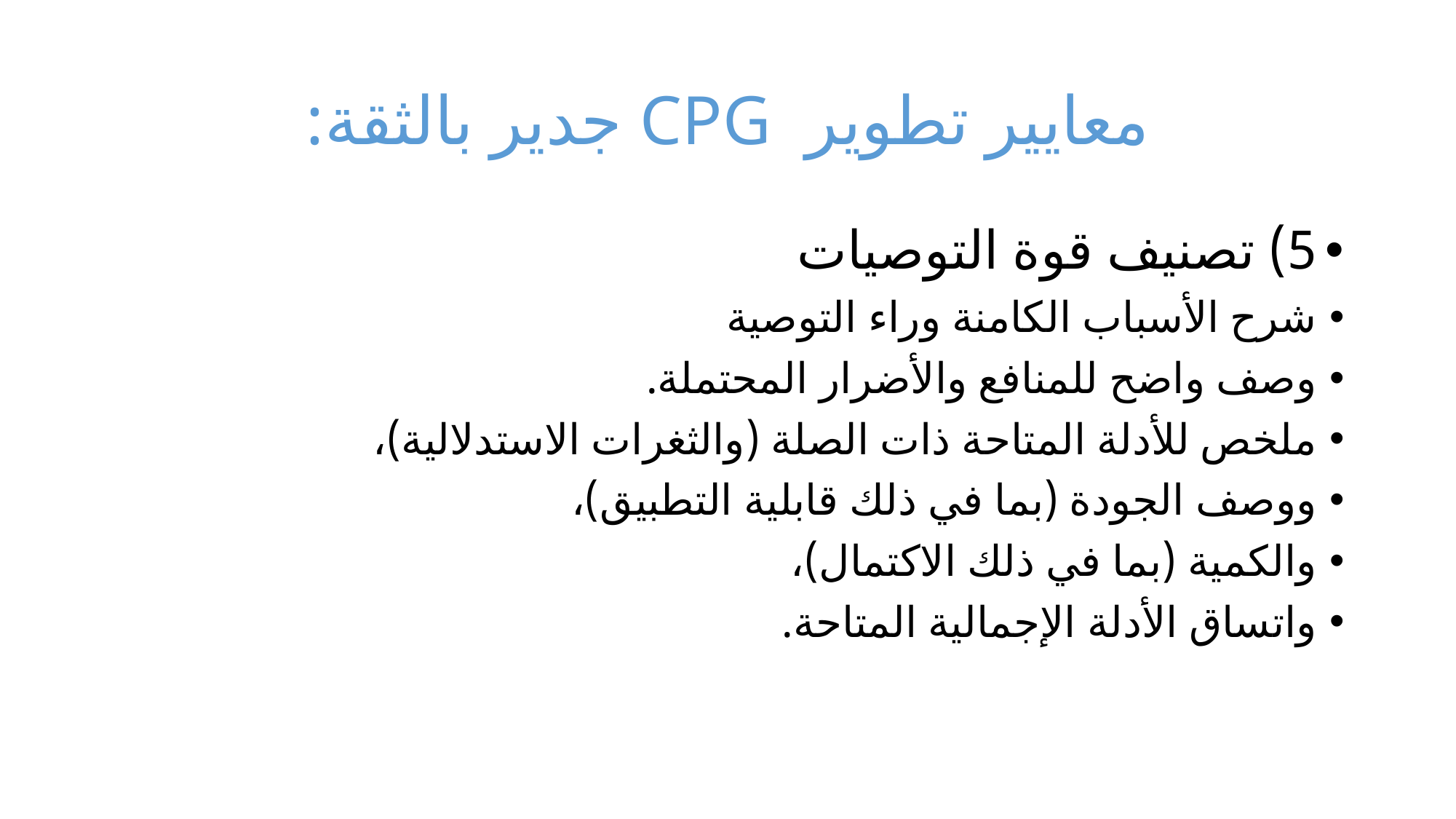

# معايير تطوير CPG جدير بالثقة:
5) تصنيف قوة التوصيات
شرح الأسباب الكامنة وراء التوصية
وصف واضح للمنافع والأضرار المحتملة.
ملخص للأدلة المتاحة ذات الصلة (والثغرات الاستدلالية)،
ووصف الجودة (بما في ذلك قابلية التطبيق)،
والكمية (بما في ذلك الاكتمال)،
واتساق الأدلة الإجمالية المتاحة.

## Slide 10
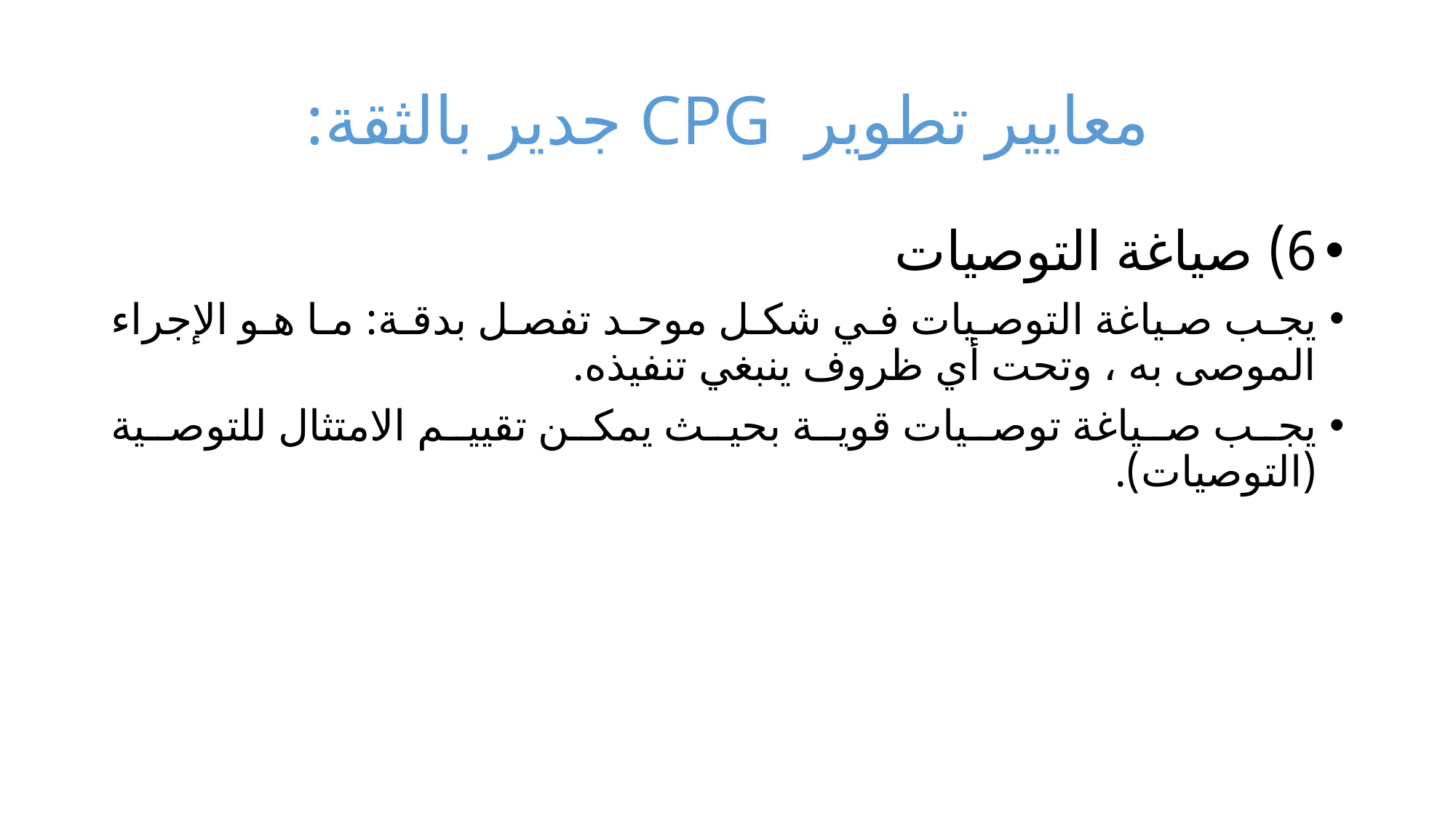

# معايير تطوير CPG جدير بالثقة:
6) صياغة التوصيات
يجب صياغة التوصيات في شكل موحد تفصل بدقة: ما هو الإجراء الموصى به ، وتحت أي ظروف ينبغي تنفيذه.
يجب صياغة توصيات قوية بحيث يمكن تقييم الامتثال للتوصية (التوصيات).

## Slide 11
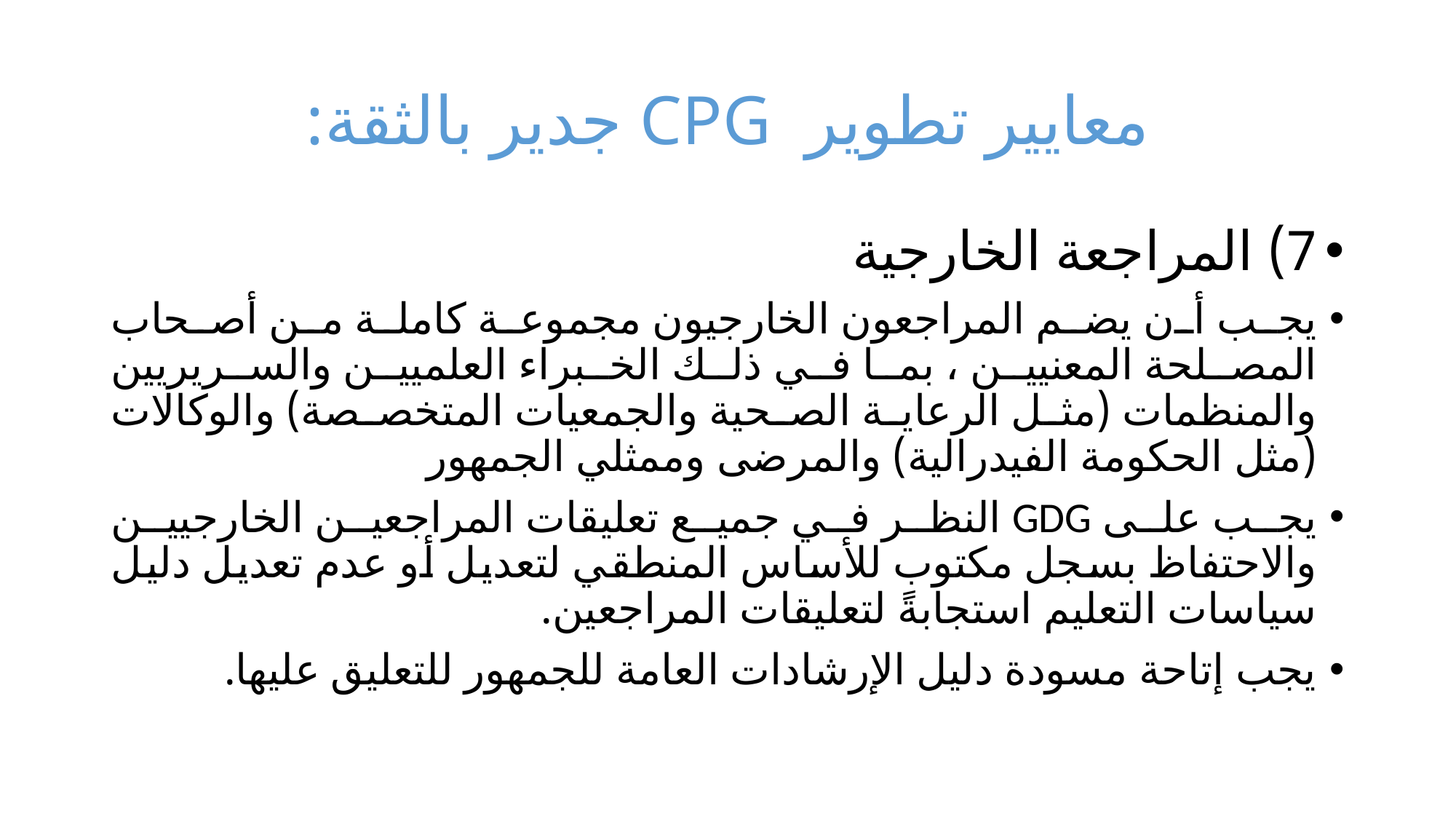

# معايير تطوير CPG جدير بالثقة:
7) المراجعة الخارجية
يجب أن يضم المراجعون الخارجيون مجموعة كاملة من أصحاب المصلحة المعنيين ، بما في ذلك الخبراء العلميين والسريريين والمنظمات (مثل الرعاية الصحية والجمعيات المتخصصة) والوكالات (مثل الحكومة الفيدرالية) والمرضى وممثلي الجمهور
يجب على GDG النظر في جميع تعليقات المراجعين الخارجيين والاحتفاظ بسجل مكتوب للأساس المنطقي لتعديل أو عدم تعديل دليل سياسات التعليم استجابةً لتعليقات المراجعين.
يجب إتاحة مسودة دليل الإرشادات العامة للجمهور للتعليق عليها.

## Slide 12
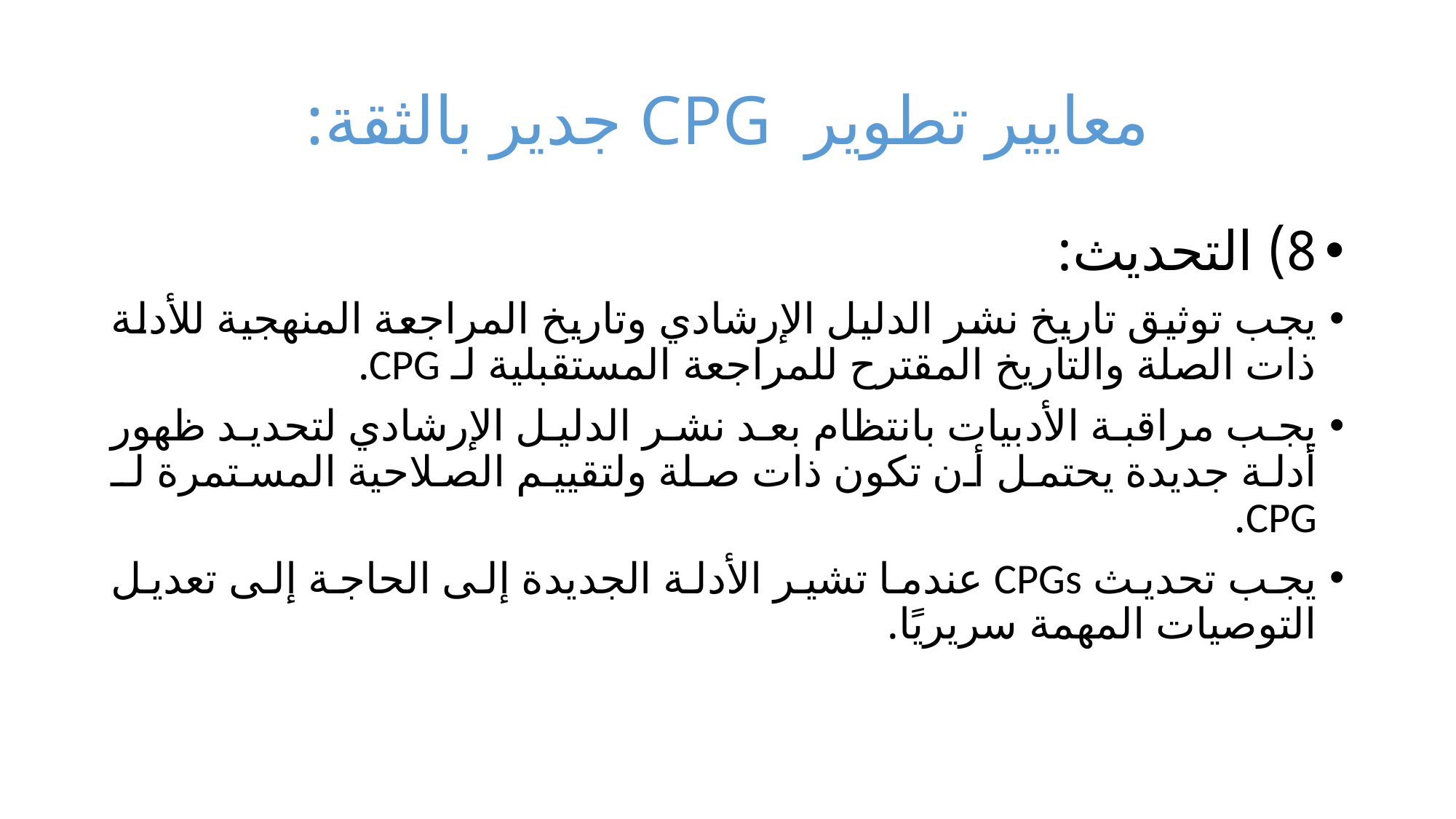

# معايير تطوير CPG جدير بالثقة:
8) التحديث:
يجب توثيق تاريخ نشر الدليل الإرشادي وتاريخ المراجعة المنهجية للأدلة ذات الصلة والتاريخ المقترح للمراجعة المستقبلية لـ CPG.
يجب مراقبة الأدبيات بانتظام بعد نشر الدليل الإرشادي لتحديد ظهور أدلة جديدة يحتمل أن تكون ذات صلة ولتقييم الصلاحية المستمرة لـ CPG.
يجب تحديث CPGs عندما تشير الأدلة الجديدة إلى الحاجة إلى تعديل التوصيات المهمة سريريًا.

## Slide 13
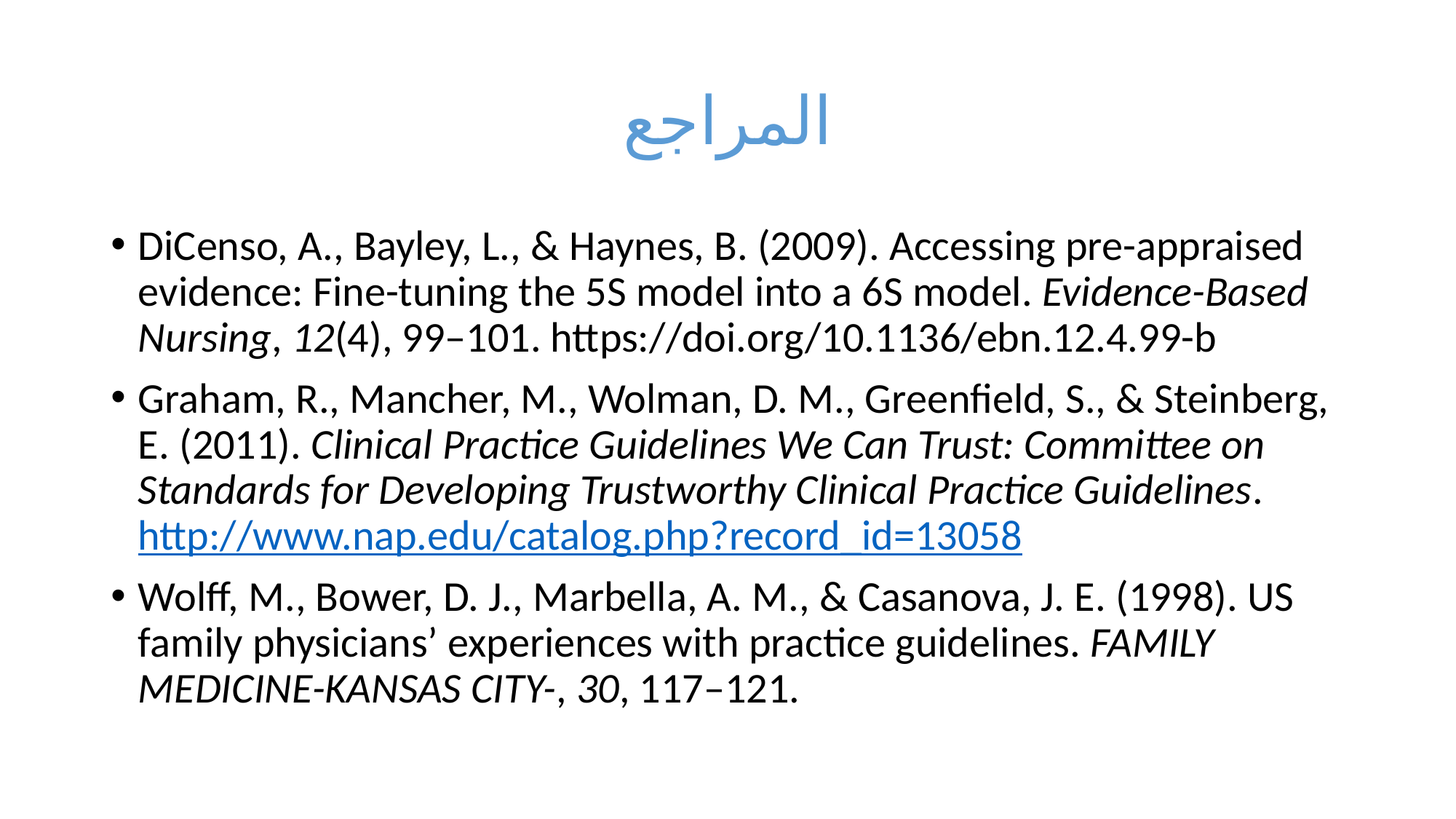

# المراجع
DiCenso, A., Bayley, L., & Haynes, B. (2009). Accessing pre-appraised evidence: Fine-tuning the 5S model into a 6S model. Evidence-Based Nursing, 12(4), 99–101. https://doi.org/10.1136/ebn.12.4.99-b
Graham, R., Mancher, M., Wolman, D. M., Greenfield, S., & Steinberg, E. (2011). Clinical Practice Guidelines We Can Trust: Committee on Standards for Developing Trustworthy Clinical Practice Guidelines. http://www.nap.edu/catalog.php?record_id=13058
Wolff, M., Bower, D. J., Marbella, A. M., & Casanova, J. E. (1998). US family physicians’ experiences with practice guidelines. FAMILY MEDICINE-KANSAS CITY-, 30, 117–121.
