# Supplementary material for: The First Asynchronous Online Evidence-Based Medicine Course for Syrian Health Workforce: Effectiveness and Feasibility Pilot Study
Source: JMIR Form Res. 2022 Oct 25;6(10):e36782. doi: 10.2196/36782 (PMC9644249; doi:10.2196/36782)
Supplement: Multimedia Appendix 11 [file formative_v6i10e36782_app11.pptx]

## Slide 1
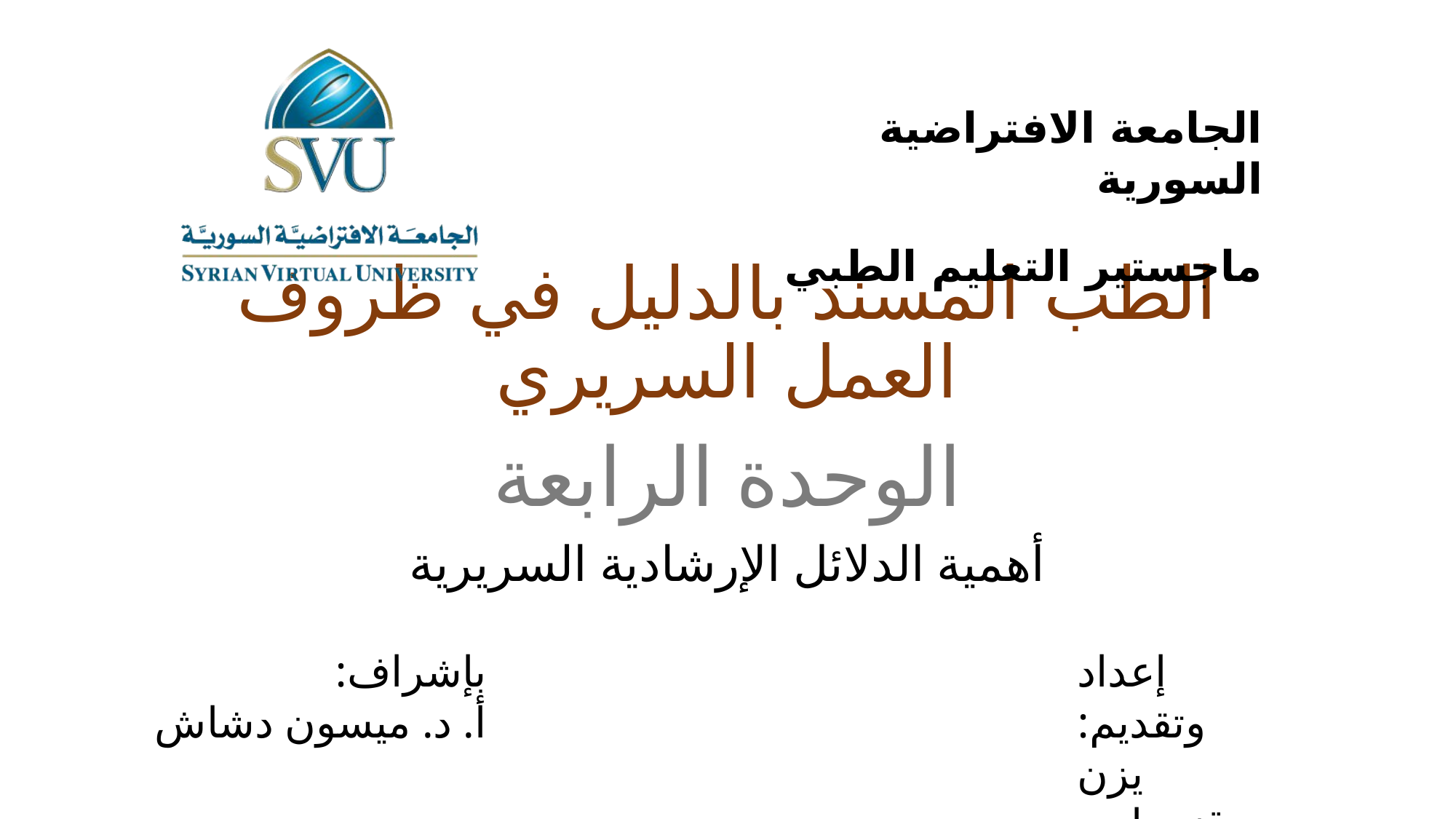

الجامعة الافتراضية السورية
ماجستير التعليم الطبي
# الطب المسند بالدليل في ظروف العمل السريري
الوحدة الرابعة
أهمية الدلائل الإرشادية السريرية
إعداد وتقديم:
يزن قنجراوي
بإشراف:
أ. د. ميسون دشاش

## Slide 2
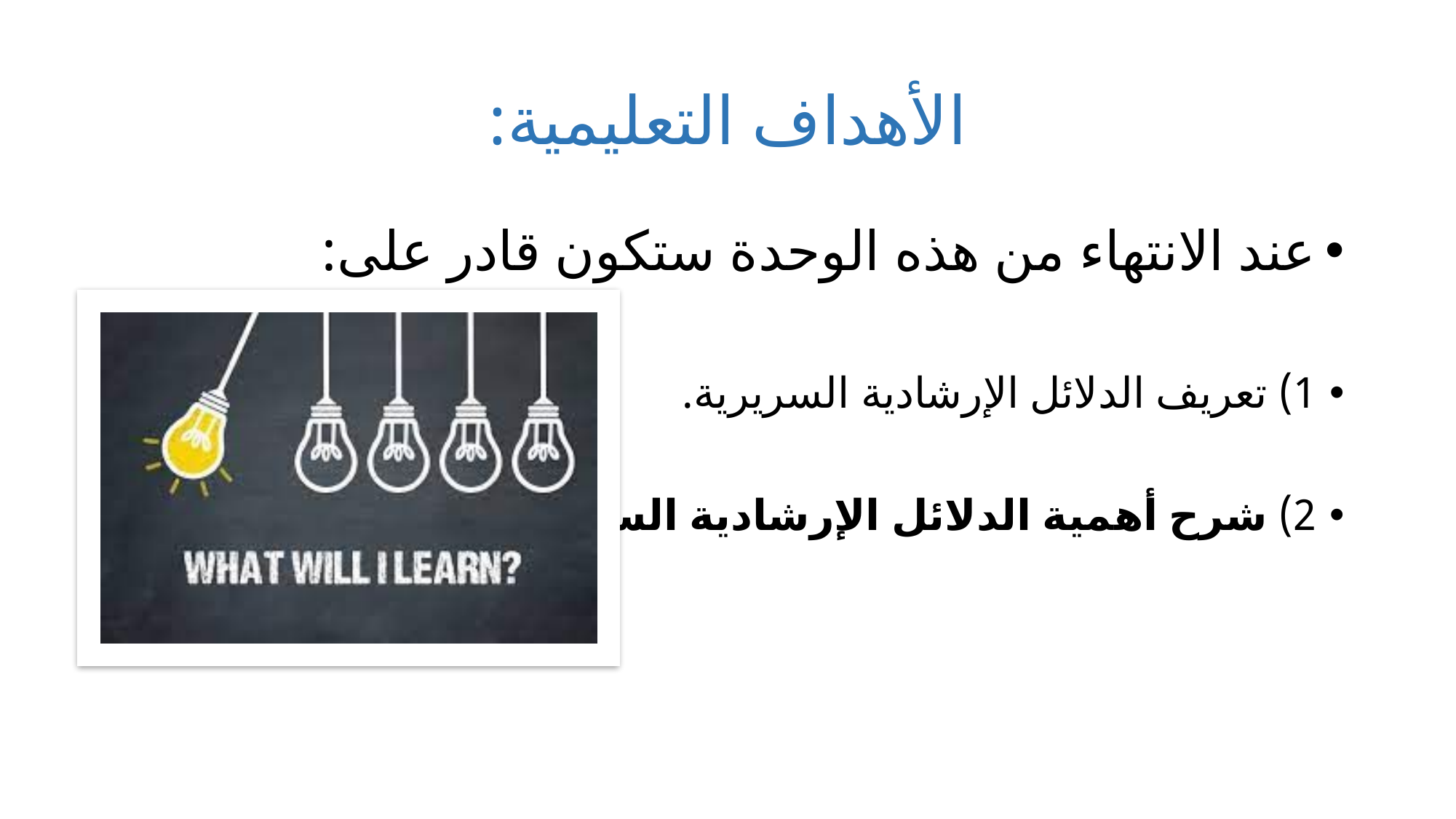

# الأهداف التعليمية:
عند الانتهاء من هذه الوحدة ستكون قادر على:
1) تعريف الدلائل الإرشادية السريرية.
2) شرح أهمية الدلائل الإرشادية السريرية.

## Slide 3
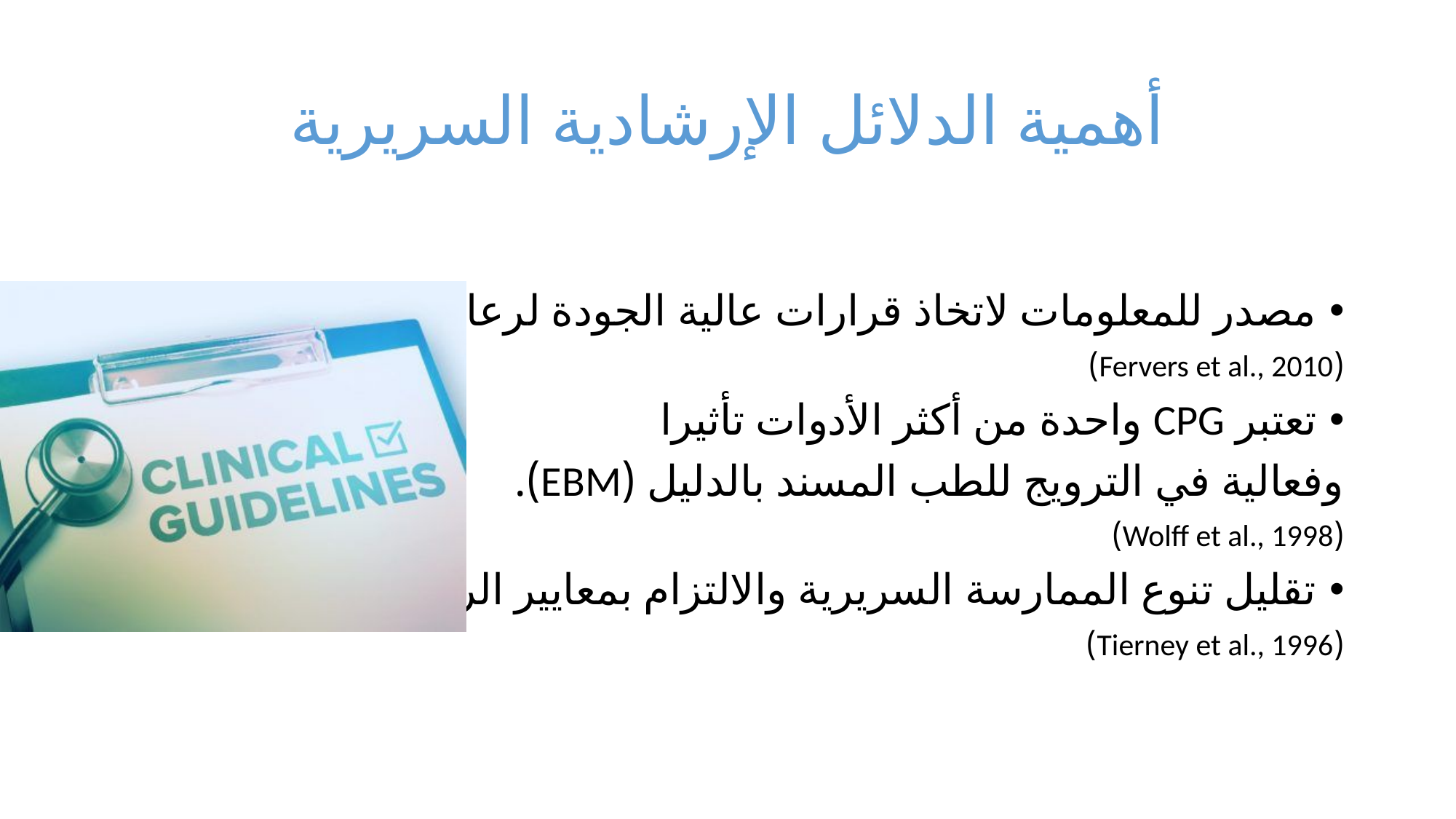

# أهمية الدلائل الإرشادية السريرية
مصدر للمعلومات لاتخاذ قرارات عالية الجودة لرعاية مرضانا.
(Fervers et al., 2010)
تعتبر CPG واحدة من أكثر الأدوات تأثيرا
وفعالية في الترويج للطب المسند بالدليل (EBM).
(Wolff et al., 1998)
تقليل تنوع الممارسة السريرية والالتزام بمعايير الرعاية الجيدة.
(Tierney et al., 1996)

## Slide 4
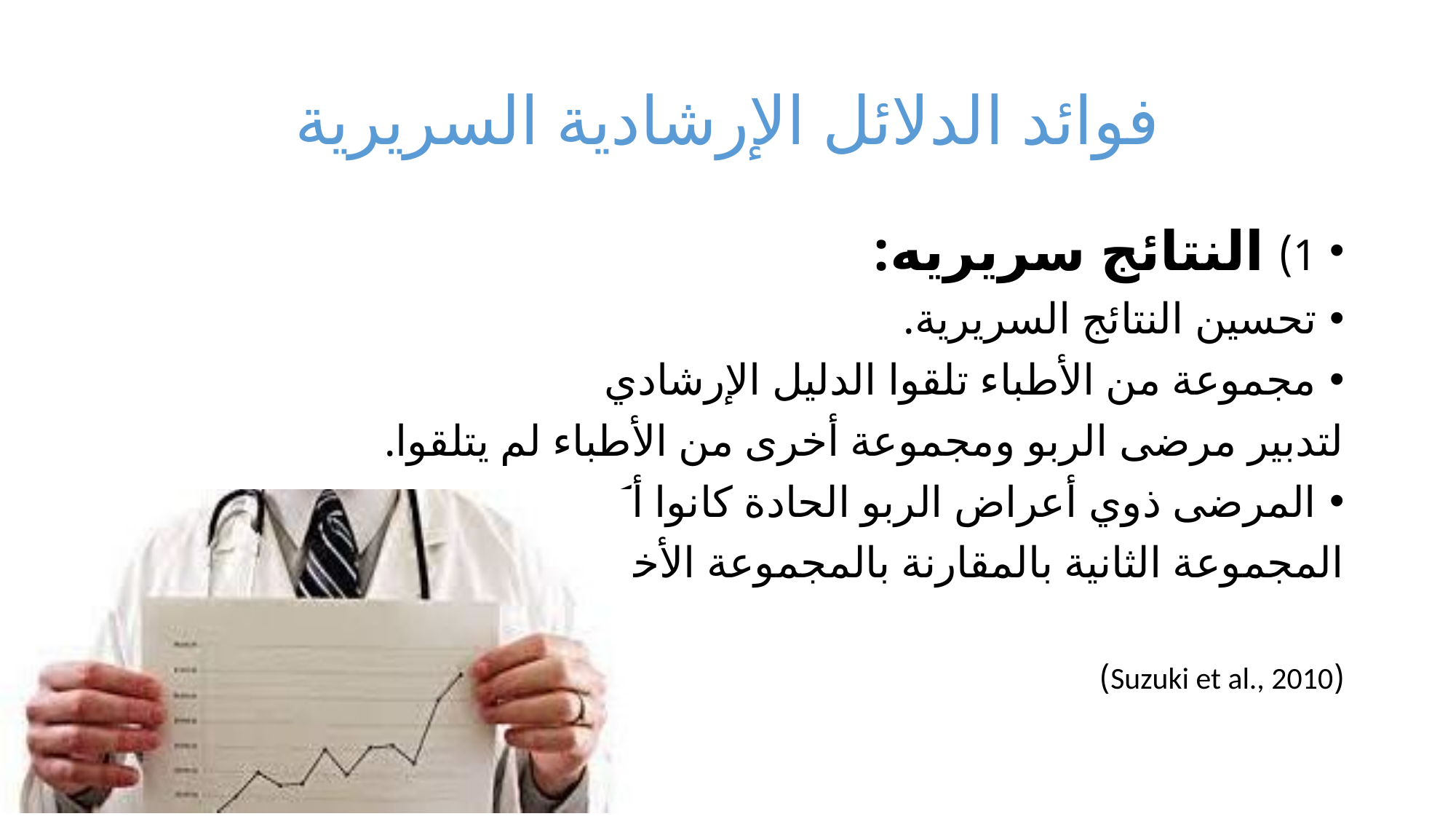

# فوائد الدلائل الإرشادية السريرية
1) النتائج سريريه:
تحسين النتائج السريرية.
مجموعة من الأطباء تلقوا الدليل الإرشادي
لتدبير مرضى الربو ومجموعة أخرى من الأطباء لم يتلقوا.
المرضى ذوي أعراض الربو الحادة كانوا أكثر في
المجموعة الثانية بالمقارنة بالمجموعة الأخرى.
(Suzuki et al., 2010)

## Slide 5
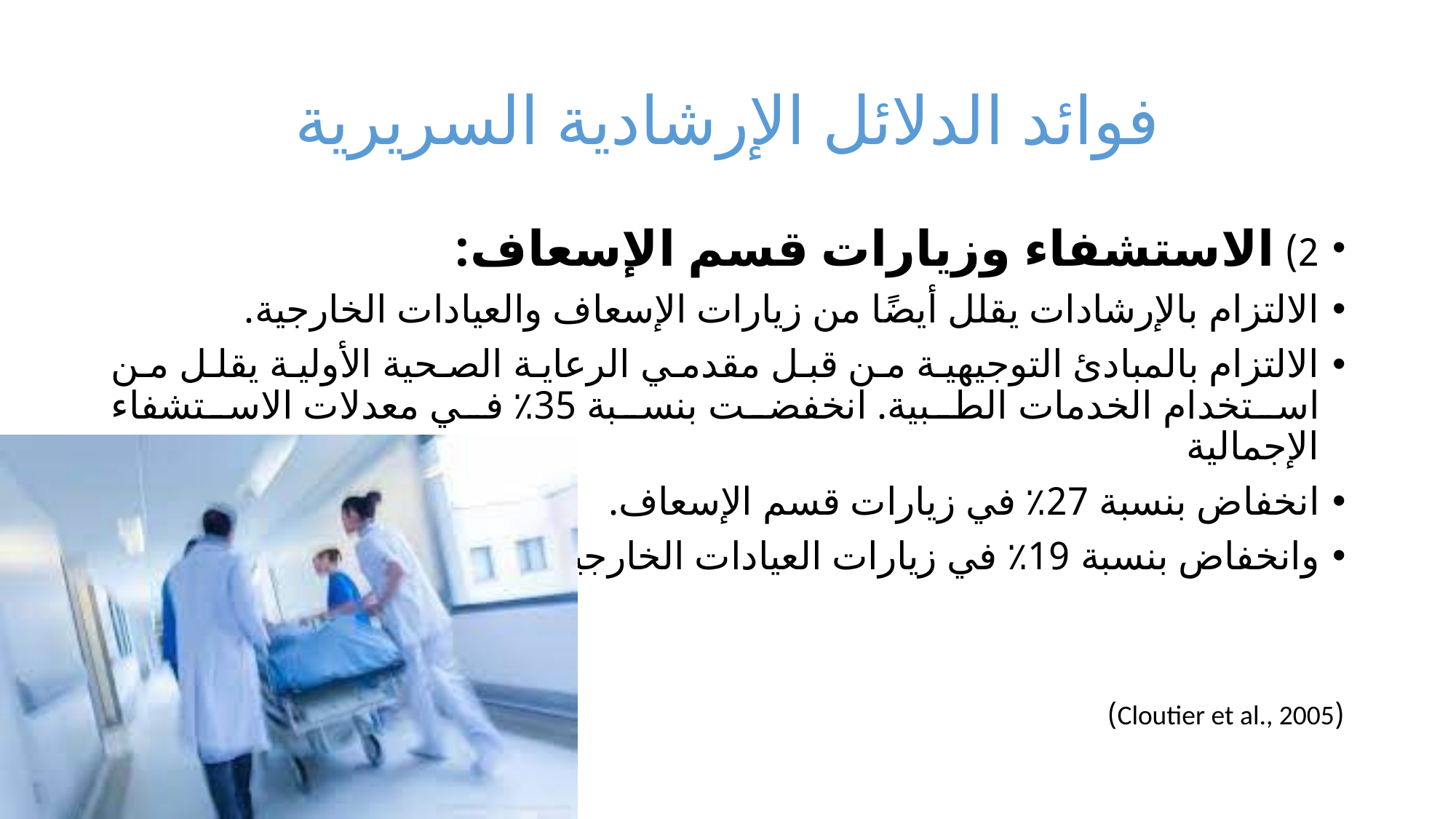

# فوائد الدلائل الإرشادية السريرية
2) الاستشفاء وزيارات قسم الإسعاف:
الالتزام بالإرشادات يقلل أيضًا من زيارات الإسعاف والعيادات الخارجية.
الالتزام بالمبادئ التوجيهية من قبل مقدمي الرعاية الصحية الأولية يقلل من استخدام الخدمات الطبية. انخفضت بنسبة 35٪ في معدلات الاستشفاء الإجمالية
انخفاض بنسبة 27٪ في زيارات قسم الإسعاف.
وانخفاض بنسبة 19٪ في زيارات العيادات الخارجية.
(Cloutier et al., 2005)

## Slide 6
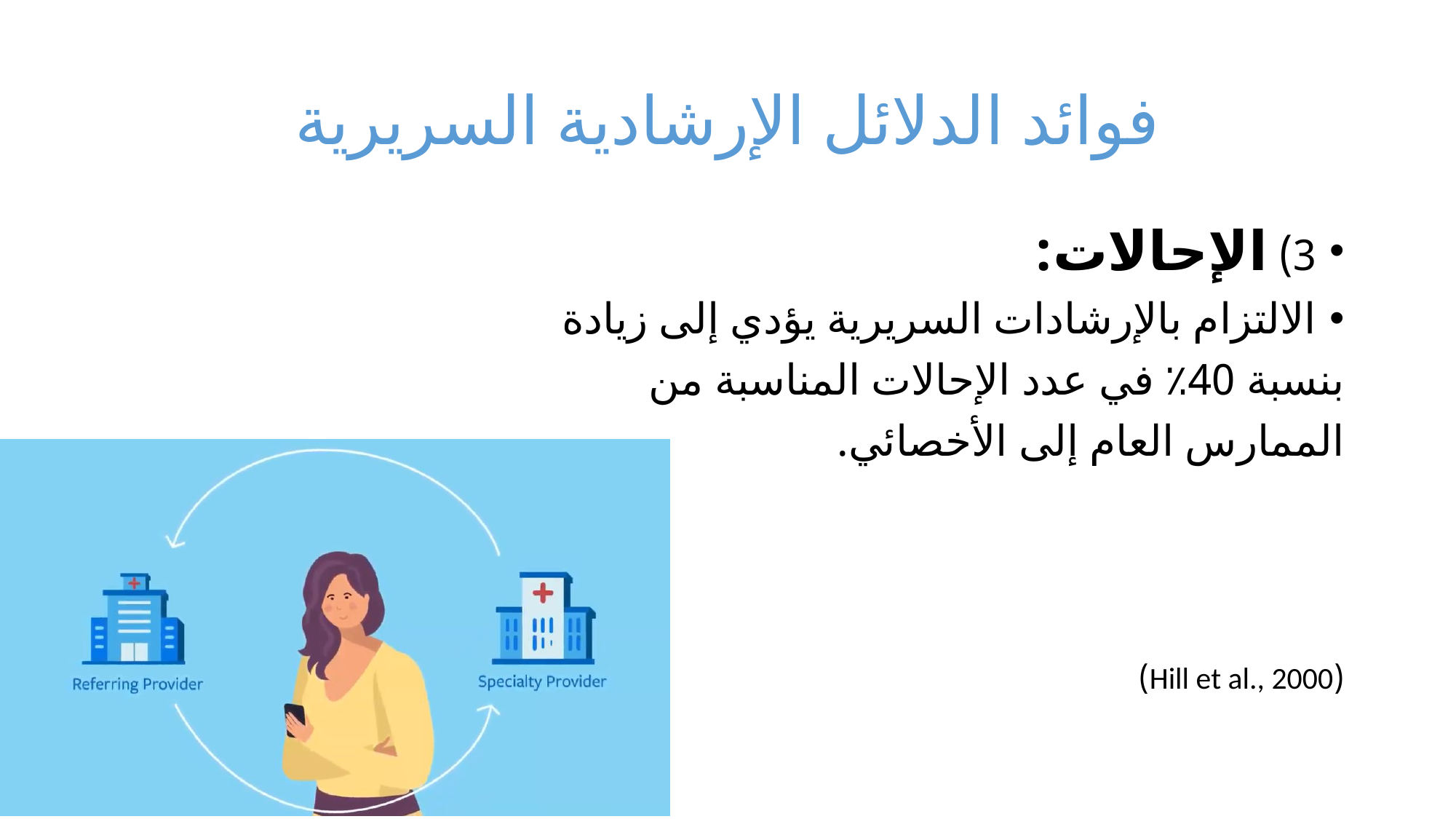

# فوائد الدلائل الإرشادية السريرية
3) الإحالات:
الالتزام بالإرشادات السريرية يؤدي إلى زيادة
بنسبة 40٪ في عدد الإحالات المناسبة من
الممارس العام إلى الأخصائي.
(Hill et al., 2000)

## Slide 7
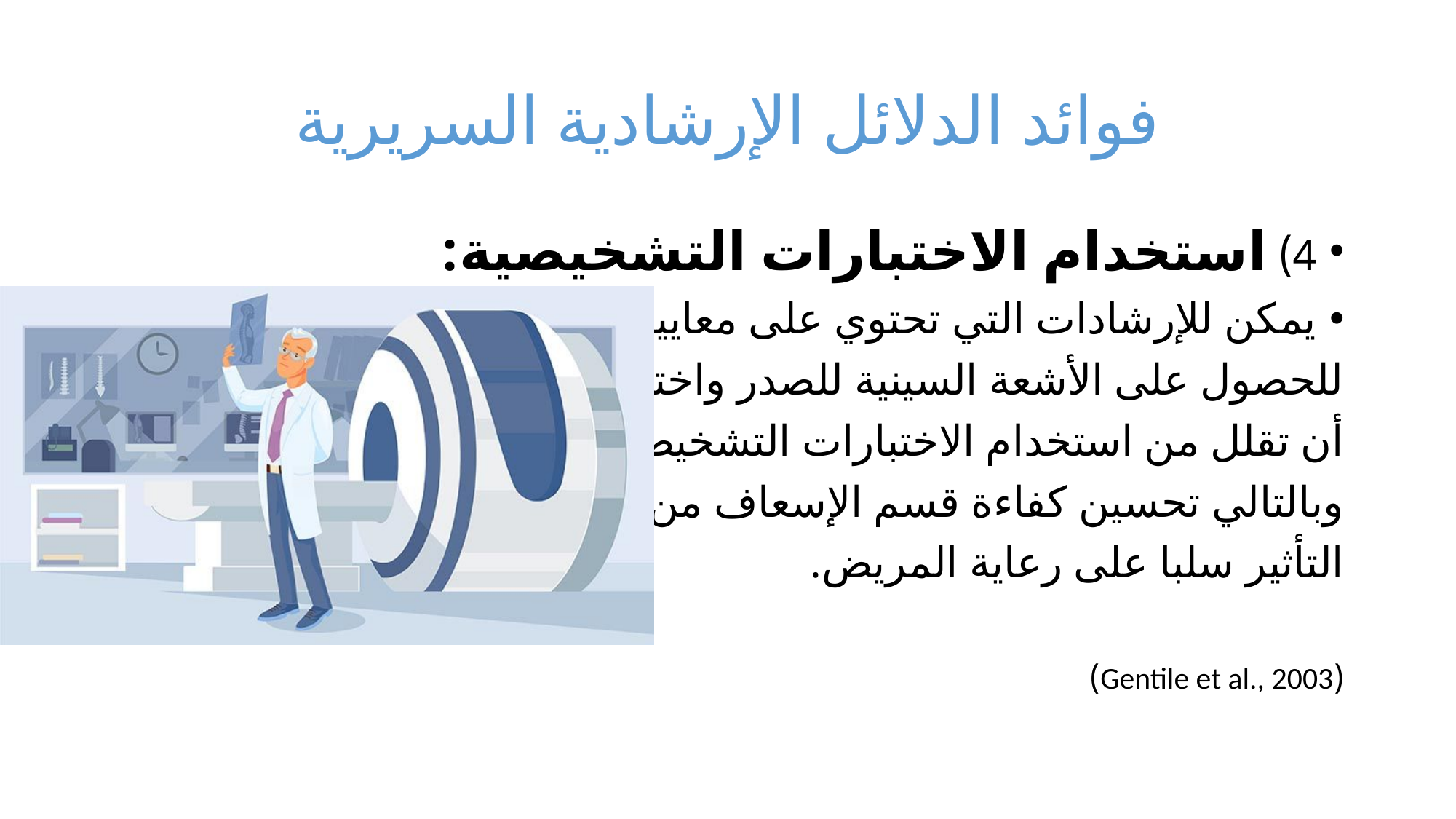

# فوائد الدلائل الإرشادية السريرية
4) استخدام الاختبارات التشخيصية:
يمكن للإرشادات التي تحتوي على معايير
للحصول على الأشعة السينية للصدر واختبارات الدم
أن تقلل من استخدام الاختبارات التشخيصية،
وبالتالي تحسين كفاءة قسم الإسعاف من دون
التأثير سلبا على رعاية المريض.
(Gentile et al., 2003)

## Slide 8
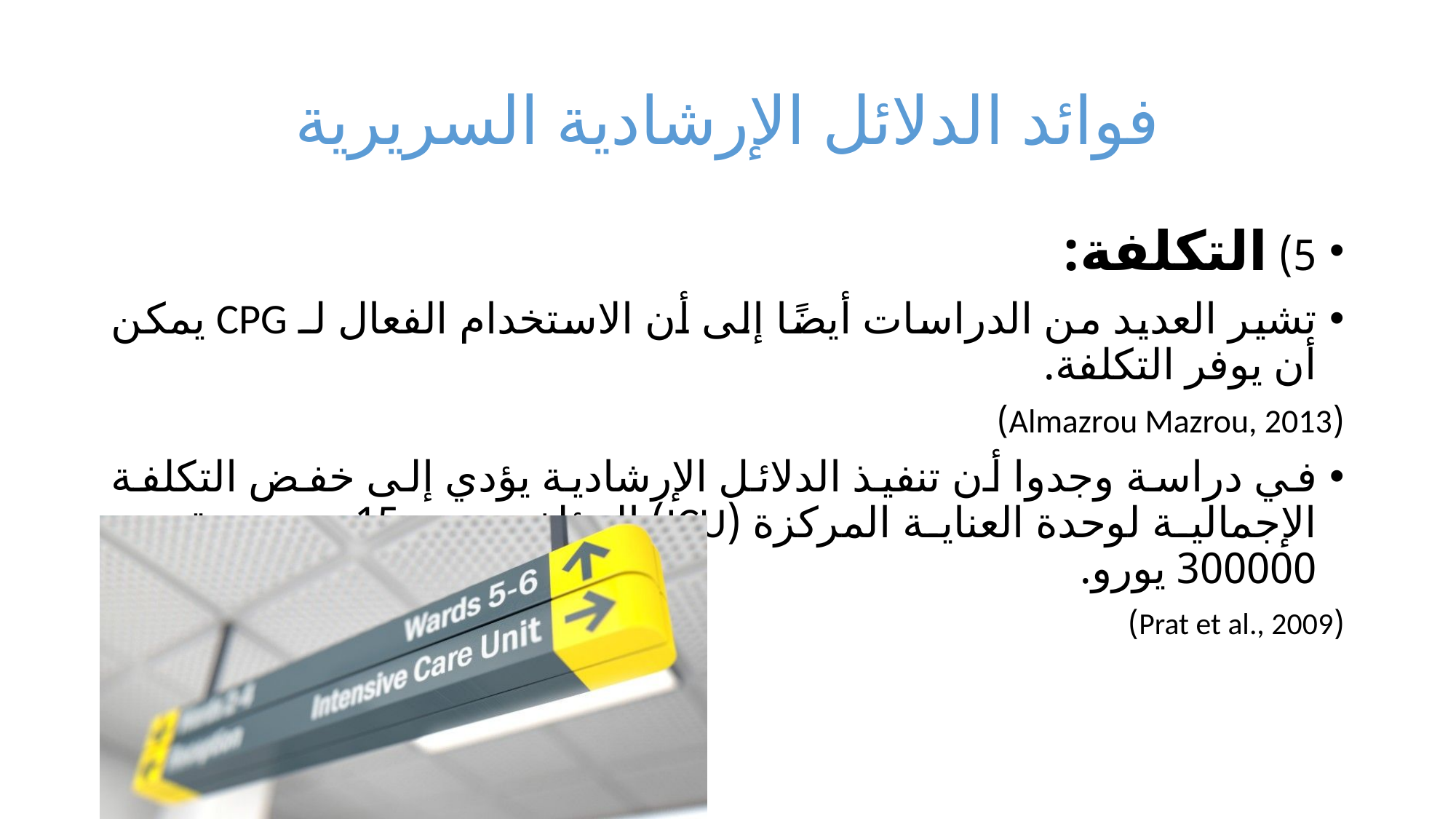

# فوائد الدلائل الإرشادية السريرية
5) التكلفة:
تشير العديد من الدراسات أيضًا إلى أن الاستخدام الفعال لـ CPG يمكن أن يوفر التكلفة.
(Almazrou Mazrou, 2013)
في دراسة وجدوا أن تنفيذ الدلائل الإرشادية يؤدي إلى خفض التكلفة الإجمالية لوحدة العناية المركزة (ICU) المؤلفة من 15 سرير بقيمة 300000 يورو.
(Prat et al., 2009)

## Slide 9
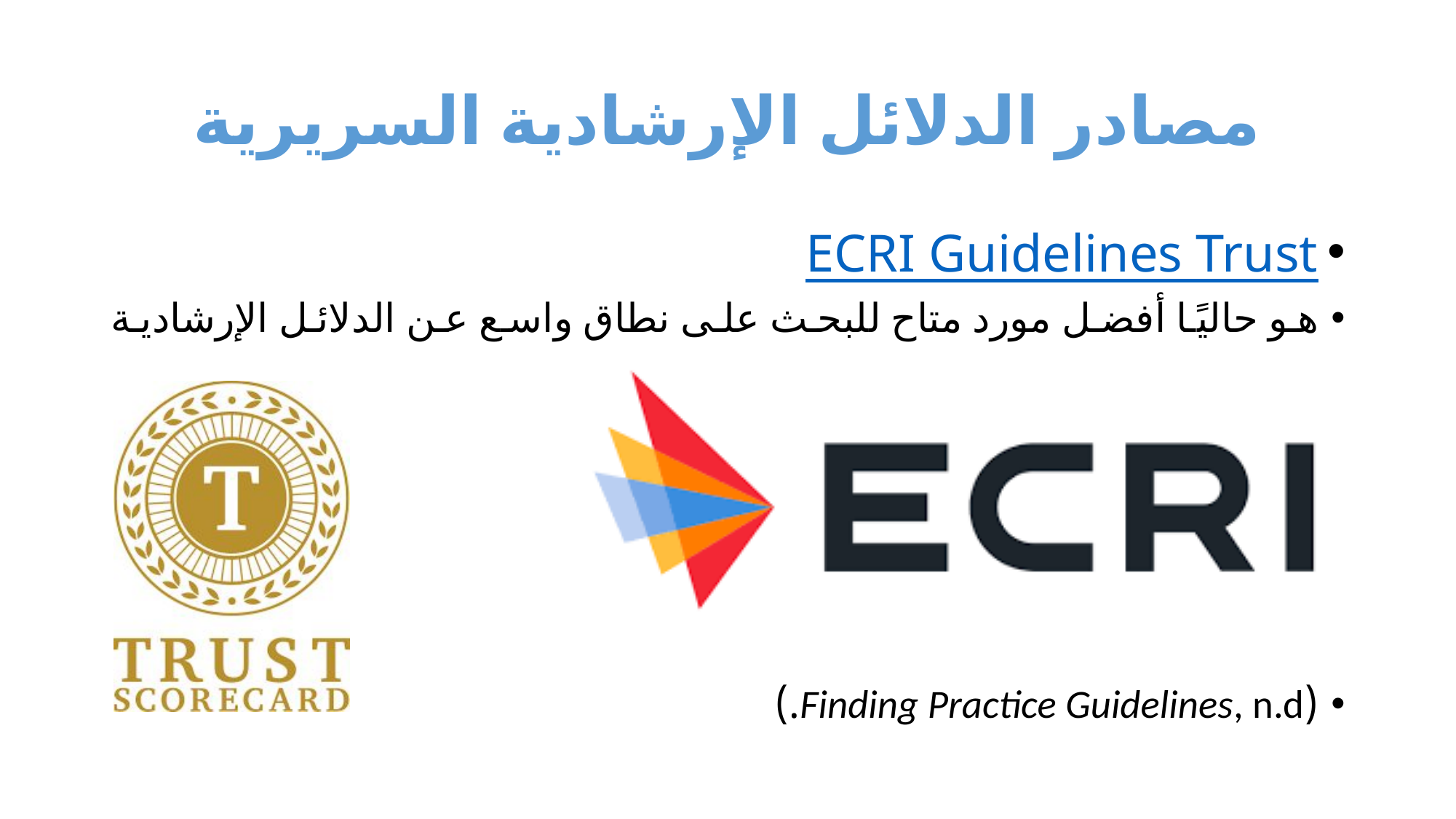

# مصادر الدلائل الإرشادية السريرية
ECRI Guidelines Trust
هو حاليًا أفضل مورد متاح للبحث على نطاق واسع عن الدلائل الإرشادية السريرية.
(Finding Practice Guidelines, n.d.)

## Slide 10
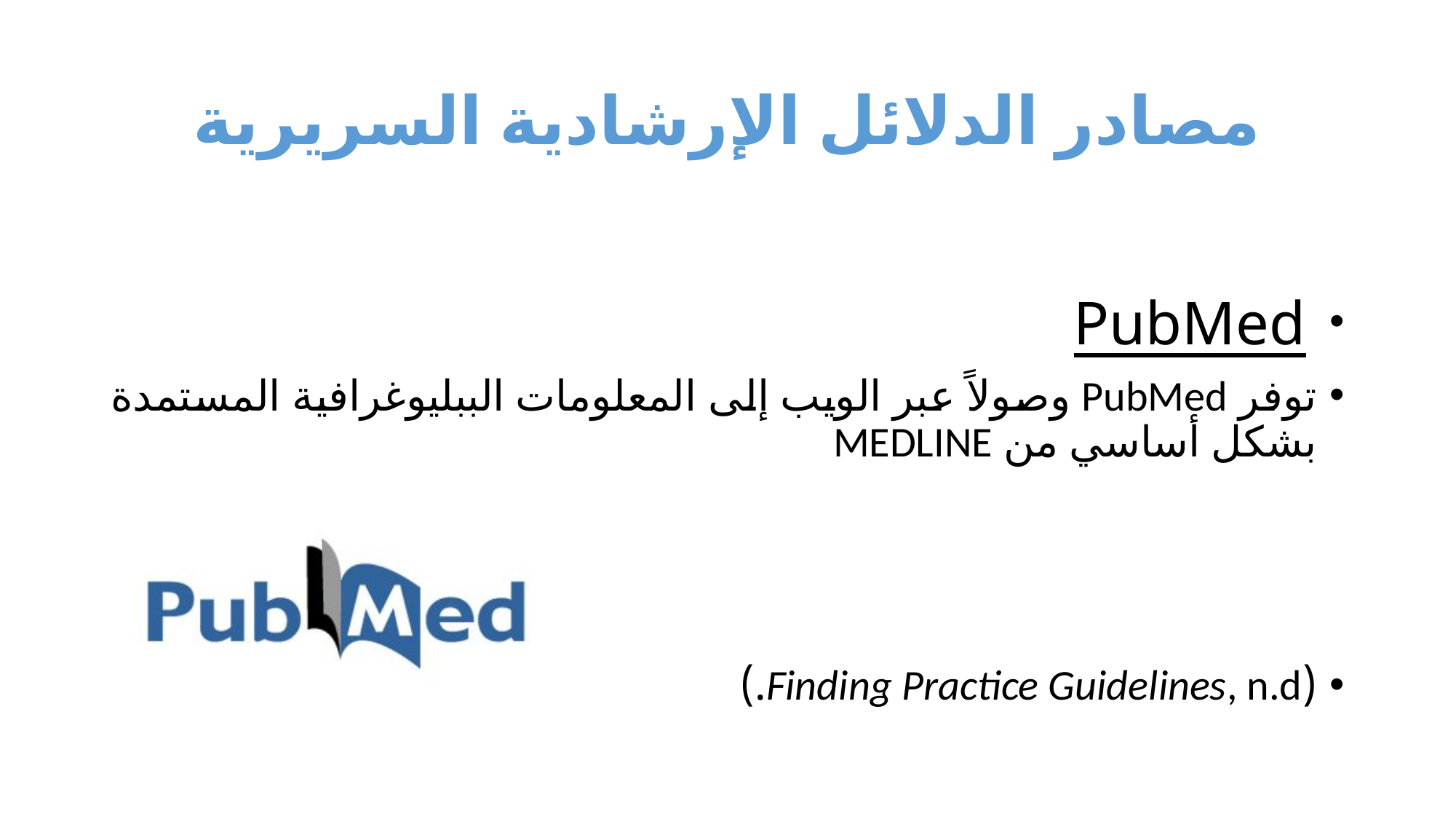

# مصادر الدلائل الإرشادية السريرية
 PubMed
توفر PubMed وصولاً عبر الويب إلى المعلومات الببليوغرافية المستمدة بشكل أساسي من MEDLINE
(Finding Practice Guidelines, n.d.)

## Slide 11
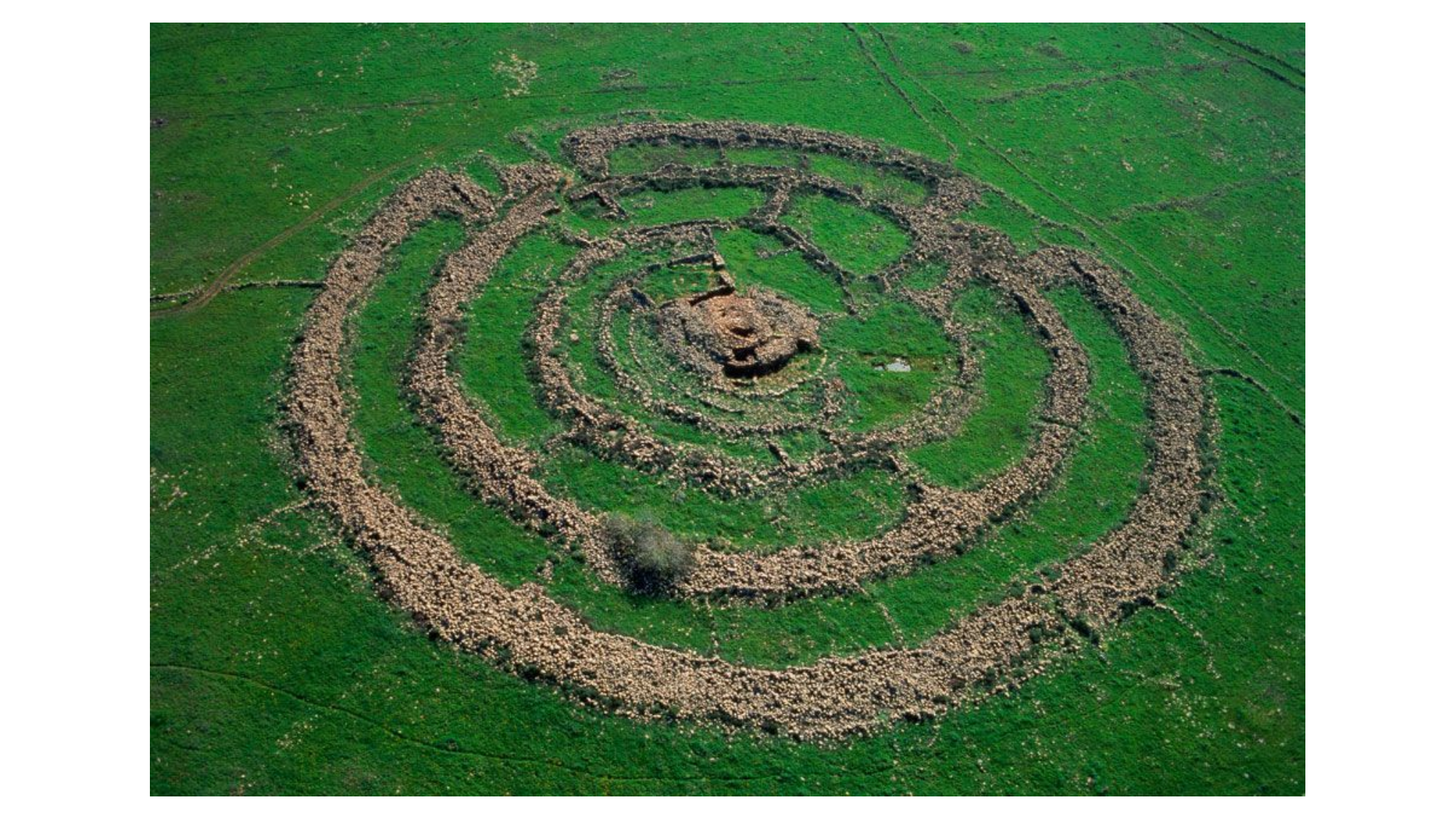

## Slide 12
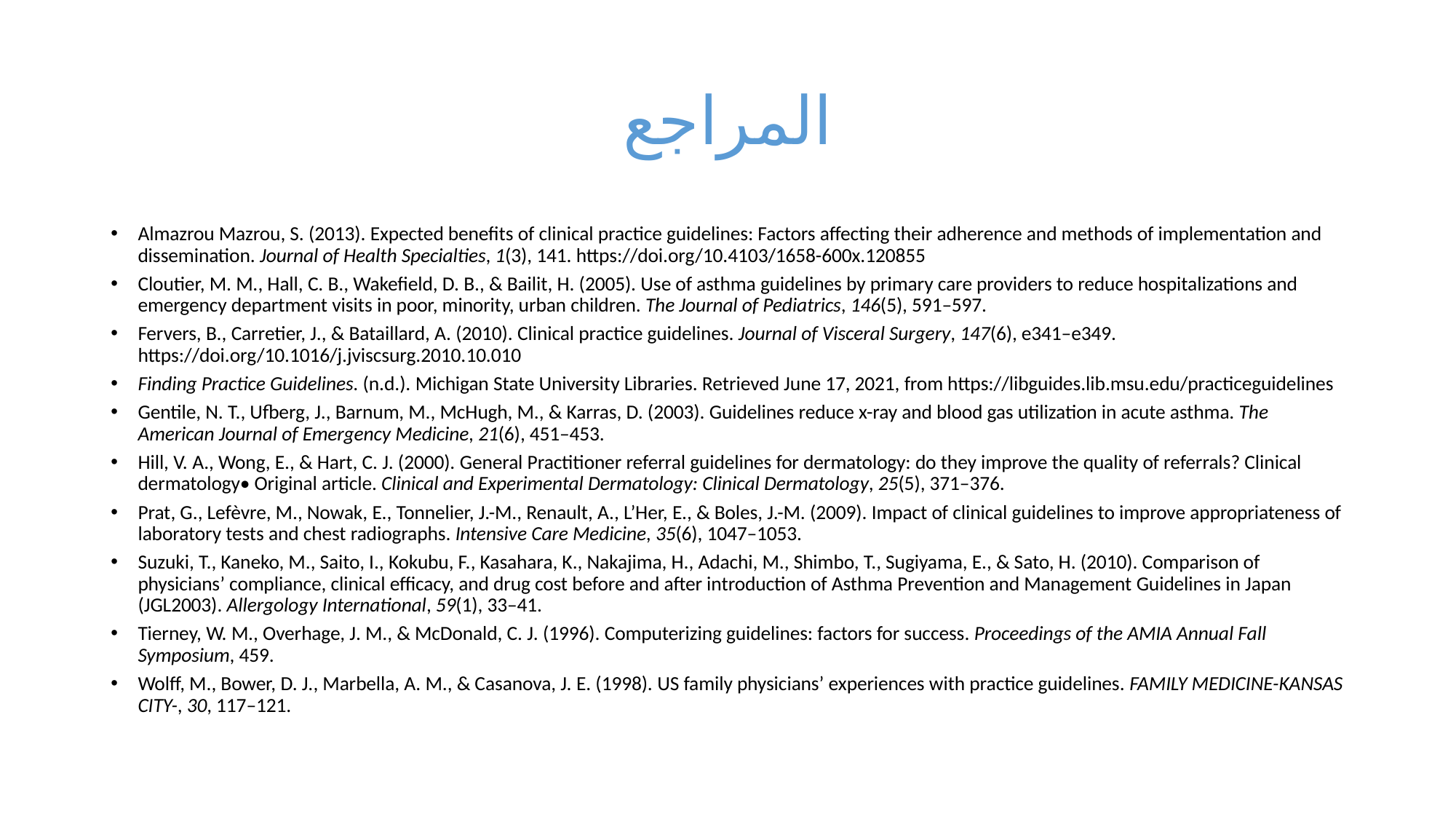

# المراجع
Almazrou Mazrou, S. (2013). Expected benefits of clinical practice guidelines: Factors affecting their adherence and methods of implementation and dissemination. Journal of Health Specialties, 1(3), 141. https://doi.org/10.4103/1658-600x.120855
Cloutier, M. M., Hall, C. B., Wakefield, D. B., & Bailit, H. (2005). Use of asthma guidelines by primary care providers to reduce hospitalizations and emergency department visits in poor, minority, urban children. The Journal of Pediatrics, 146(5), 591–597.
Fervers, B., Carretier, J., & Bataillard, A. (2010). Clinical practice guidelines. Journal of Visceral Surgery, 147(6), e341–e349. https://doi.org/10.1016/j.jviscsurg.2010.10.010
Finding Practice Guidelines. (n.d.). Michigan State University Libraries. Retrieved June 17, 2021, from https://libguides.lib.msu.edu/practiceguidelines
Gentile, N. T., Ufberg, J., Barnum, M., McHugh, M., & Karras, D. (2003). Guidelines reduce x-ray and blood gas utilization in acute asthma. The American Journal of Emergency Medicine, 21(6), 451–453.
Hill, V. A., Wong, E., & Hart, C. J. (2000). General Practitioner referral guidelines for dermatology: do they improve the quality of referrals? Clinical dermatology• Original article. Clinical and Experimental Dermatology: Clinical Dermatology, 25(5), 371–376.
Prat, G., Lefèvre, M., Nowak, E., Tonnelier, J.-M., Renault, A., L’Her, E., & Boles, J.-M. (2009). Impact of clinical guidelines to improve appropriateness of laboratory tests and chest radiographs. Intensive Care Medicine, 35(6), 1047–1053.
Suzuki, T., Kaneko, M., Saito, I., Kokubu, F., Kasahara, K., Nakajima, H., Adachi, M., Shimbo, T., Sugiyama, E., & Sato, H. (2010). Comparison of physicians’ compliance, clinical efficacy, and drug cost before and after introduction of Asthma Prevention and Management Guidelines in Japan (JGL2003). Allergology International, 59(1), 33–41.
Tierney, W. M., Overhage, J. M., & McDonald, C. J. (1996). Computerizing guidelines: factors for success. Proceedings of the AMIA Annual Fall Symposium, 459.
Wolff, M., Bower, D. J., Marbella, A. M., & Casanova, J. E. (1998). US family physicians’ experiences with practice guidelines. FAMILY MEDICINE-KANSAS CITY-, 30, 117–121.
